# Specimen-level phylogenetic analysis and taxonomic revision of North American metoposaurids: a case for monospecific genera in vertebrate paleontology

**DOI:** 10.7717/peerj.21720

**Published:** 2026-09-23

**Authors:** Aaron M. Kufner

**Affiliations:** 1 Department of Geoscience, University of Wisconsin-Madison, Madison, Wisconsin, United States; 2 UW Geology Museum, University of Wisconsin-Madison, Madison, Wisconsin, United States

**Keywords:** Metoposauridae, Triassic, Specimen-level analysis, Amphibians, Temnospondyli, Phylogeny

## Abstract

The Metoposauridae were a widespread family of aquatic Late Triassic temnospondyl amphibians known from numerous localities in non-marine strata of the Northern Hemisphere, India, and Madagascar. Recent descriptions of intraspecific variation in skulls from hyper accumulations suggest some characters used in species diagnoses may be variable within hypothesized populations. This realization necessitates the reevaluation of taxonomic diagnoses for previously established species. Multiple monospecific hyper accumulations of different metoposaurid taxa are known in North America providing a relatively large sample to assess the validity of the North American taxa: *Anaschisma browni*, *Buettnererpeton bakeri*, and *Apachesaurus gregorii*. The purported ‘small-bodied’ metoposaurid *A. gregorii* has recently been suggested to be predominantly represented by juvenile material; whether this material represents a juvenile of a previously described large-bodied metoposaurid or is indeed a distinct taxon has not been tested. A specimen-level character matrix incorporating scores from all name-bearing specimens diagnostic to the species level as well as numerous referred specimens was constructed, and the interpretations are validated by the inclusion of photographs and interpretive drawings (where necessary for clarity) of specimens included in the matrix. The synonymy of specimens from Texas, New Mexico, and Arizona referred to *An. browni* was not supported, and to avoid exacerbating taxonomic instability of the Metoposauridae, two novel genera are proposed to encompass two previously described species from Texas (*Paranaschisma* gen. nov. *howardensis* and *Tecovaserpeton* gen. nov. *perfectum*). A putative adult specimen of *A. gregorii* was identified, and ontogenetic variation in *B. bakeri* and *A. gregorii* was compared to propose evolutionary scenarios related to heterochronic shifts in metoposaurids that could be tested in future studies. Reconciling outstanding challenges with the assessment of ontogenetic variation in metoposaurids will rely on the recovery, description, and phylogenetic analysis of specimens that occupy the size gap between the large-bodied and small-bodied Late Triassic metoposaurids in the western USA in this specimen-level framework.

## Introduction

An outstanding and long recognized challenge in vertebrate paleontology is limited sampling that cannot capture the range of intraspecific variation present in modern vertebrates (*e.g*., Darda & Wake, 2015; Ivanović et al., 2023; Papežíková et al., 2024; Stilson, Bell & Mead, 2017). Many morphologically novel extinct taxa are known solely from partial remains of one or a handful of individuals, precluding direct observation of population dynamics (*e.g*., age distributions, ontogenetic variation, morphological plasticity, *etc*.). Sample size can be improved with additional specimens, but new specimens must overlap with existing specimens in taxonomically informative morphology which is likely to change as new taxa are discovered or more complete specimens of existing taxa are recovered (*e.g*., Fitch et al., 2023; Hunt, 1993; Kammerer, Angielczyk & Fröbisch, 2011). Ideally, numerous individuals of different ages could be examined to determine whether morphological variation could be due to ontogeny, sexual dimorphism, or standing anatomical variation, but preservational biases in the non-marine vertebrate fossil record limit these cases to rare exceptions to the rule (*e.g*., Gee, Haridy & Reisz, 2020; Schoch & Mujal, 2022; Sulej, 2007; Witzmann, 2005).

Unique taphonomic instances of hyperaccumulation such as mass mortality assemblages (*sensu* Rogers, Eberth & Fiorillo, 2007) provide a unique opportunity to observe variation otherwise obscured by preservational circumstances. For example, some temnospondyl amphibians are represented by numerous nearly-complete elements in varying states of articulation (Dutuit, 1976b; Liu, 2016; So et al., 2024; Sulej, 2007). Among temnospondyls, metoposaurid stereospondyls are globally distributed as is their presence in hyperaccumulations in non-marine Upper Triassic deposits (Kufner et al., 2025). This has led to several studies documenting morphological variation (Gee & Kufner, 2022; Lucas et al., 2016; Rinehart, Lucas & Heckert, 2024; Sulej, 2007) and histological variation of metoposaurids (Konietzko-Meier, Bodzioch & Sander, 2012; Konietzko-Meier & Klein, 2013; Konietzko-Meier & Sander, 2013; Steyer et al., 2004). Their near global presence in the Upper Triassic has also been suggested have some utility to help constrain the ages of geographically disparate strata provided diagnostic specimens referrable to the same genus or species are present (*e.g*., Lovelace et al., 2024; Lucas, 2020).

Sulej (2007) was the first to explicitly document intraspecific variation in commonly employed diagnostic features in a population of a metoposaurid—*Metoposaurus krasiejowensis* Sulej, 2002 (*sensu* Brusatte et al., 2015). The vast majority of the polymorphic features of *M. krasiejowensis* identified by Sulej (2007) are found in the skull roof, including both the presence and absence of a lacrimal contribution to the orbital margin. The lacrimal contribution to the orbital margin is a longstanding character used to differentiate metoposaurid taxa, especially the three most commonly recognized species from North America—*Anaschisma browni* Branson, 1905 (*sensu* Gee, Parker & Marsh, 2020), *Buettnererpeton bakeri* Case, 1931 (*sensu* Gee & Kufner, 2022), and *Apachesaurus gregorii* Hunt, 1993—as well as historically the European metoposaurid *Metoposaurus diagnosticus* Meyer, 1842 (Lydekker, 1890) (but see Sulej, 2002). However, only a single described specimen of *M. krasiejowensis* has a lacrimal excluded from the orbital margin (ZPAL AbIII/681, fig. 12: Sulej, 2007). A recent redescription and taxonomic revision of *Buettnererpeton bakeri* also highlighted intraspecific variation in the skull roof of specimens from the Elkins Place bonebed (the type locality of *B. bakeri*), but the lacrimal was invariably excluded from the orbital margin (Gee & Kufner, 2022). Similarly, recent descriptions of hypothesized metoposaurid populations referred to “*Koskinonodon perfectum*” have reported variation in some features of the skull roof, but the lacrimal contribution to the orbital margin is ubiquitous (Lucas et al., 2016; Rinehart, Lucas & Heckert, 2024).

Although some authors maintain the exclusion of the lacrimal from the orbital margin of *Metoposaurus* spp. (Buffa, Jalil & Steyer, 2019; Rinehart, Lucas & Heckert, 2024), other authors have used the majority condition for the lacrimal contribution to the orbital margin of *M. krasiejowensis* in phylogenetic inference of metoposaurids (Chakravorti & Sengupta, 2019; Gee, Parker & Marsh, 2020) or treated this character as polymorphic (Gee, Parker & Marsh, 2020; Gee & Kufner, 2022). The most recent published revision to a referred specimen of *M. diagnosticus* suggests a lacrimal contribution to the orbital margin (Sulej, 2002). The broad consensus in the literature of the assignment of *Metoposaurus krasiejowensis* to the genus *Metoposaurus* persists while most of the specimens have a lacrimal contribution to the orbital margin (Brusatte et al., 2015; Gee & Kufner, 2022; Lucas et al., 2016; Rinehart, Lucas & Heckert, 2024; Sulej, 2007). One specimen of *M*. *krasiejowensis* does lack a lacrimal-orbital margin contact (fig. 12: Sulej, 2007), but it is a singleton within this larger sample in which the lacrimal contribution to the orbital margin is otherwise ubiquitous. The holotype of *Apachesaurus gregorii* (UCMP 63845) and a referred specimen (UCMP 171591) also lack a lacrimal contribution to the orbital margin, but a referred large-bodied specimen (UCMP 27051; this study) has this contribution suggesting variability or even ontogenetic influence in the states of this character within *A. gregorii* (see below).

**Figure 1:**
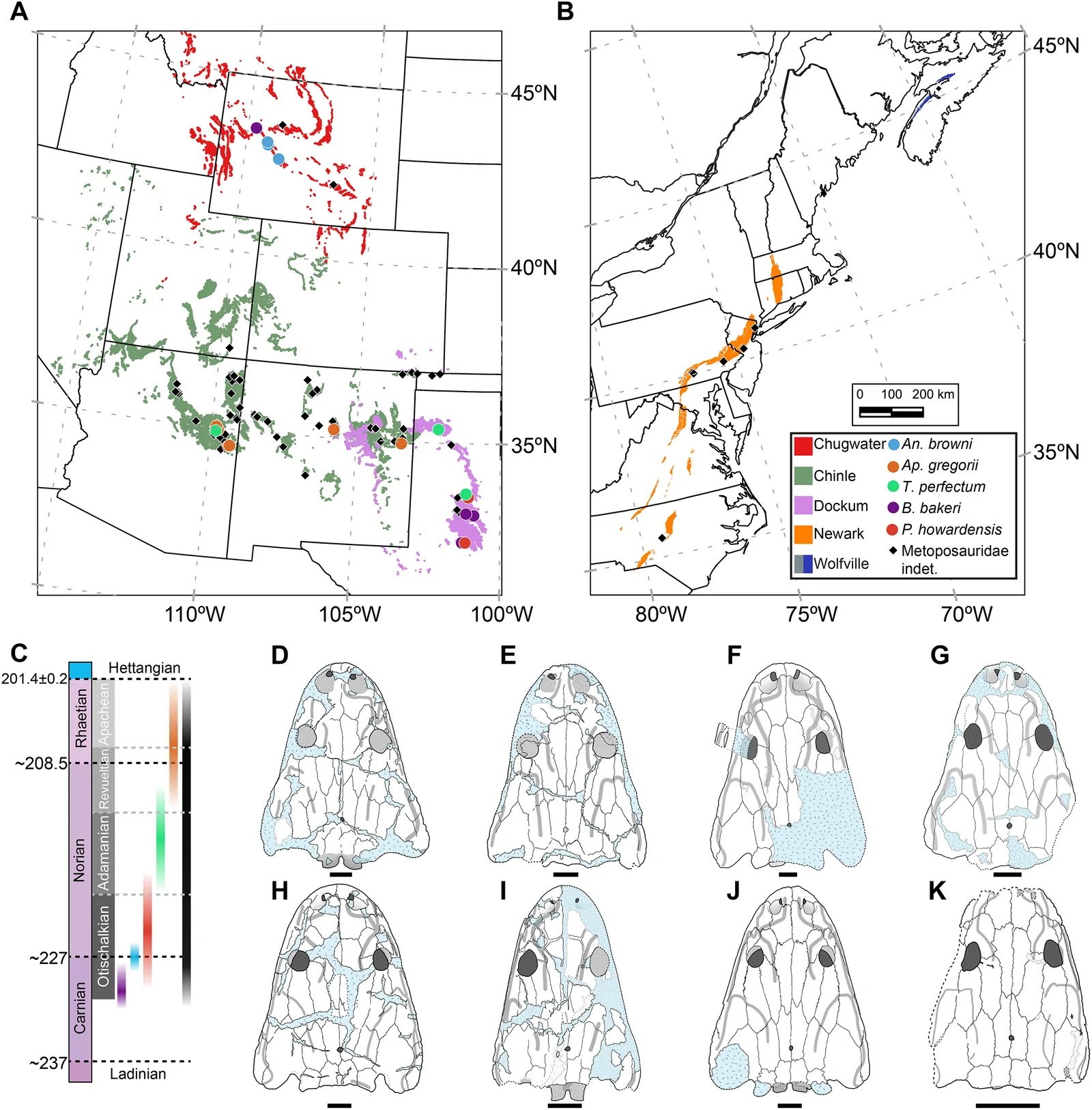
The geographic and stratigraphic distribution of metoposaurids in North America and name-bearing, diagnostic metoposaurid specimens. (A) Map of Upper Triassic outcrops in the western United States with metoposaurid occurrences; (B) map of map of Upper Triassic outcrops in the eastern United States and Canada with metoposaurid occurrences; (C) simplified chronostratigraphic distribution of valid metoposaurid taxa; (D) FMNH UC 447 (*Anaschisma browni*); (E) FMNH UC 448 (“*Anaschisma brachygnatha*”); (F) MU 537 (“*Koskinonodon princeps*”); (G) MU 517 (“*Borborophagus wyomingensis*”); (H) TMM 31100-30 (*Paranaschisma* gen. nov. *howardensis*); (I) UMMP 13055 (*Buettnererpeton bakeri*); (J) UMMP 7475 (*Tecovaserpeton* gen. nov. *perfectum*); (K) UCMP 63845 (*Apachesaurus gregorii*). See legend in (B) for map details and colors used in (A–C). For (D–K), blue hatching represents reconstruction. Scale bars equal 5 cm and are located under each corresponding subfigure in (D–K). Species referrals for a given locality are based on the results of this study. (F) and (G) are modified after (Kufner & Gee, 2021); (I) is modified after (Gee & Kufner, 2022). Outcrop area in (A) and (B) retrieved from https://macrostrat.org/ in March, 2020 (Peters, Husson & Czaplewski, 2018) and modified from Lovelace et al. (2024).

In their phylogenetic analyses of the Metoposauridae, Gee & Kufner (2022) scored species-level operational taxonomic units (OTUs) with all described polymorphic states, ultimately weighting all polymorphisms of an OTU equally even if a majority condition could be identified. Building upon the inclusion of polymorphic states in character matrices, specimen-level analyses can test whether previously described taxa form natural groupings or whether intraspecific variation present in hypothesized populations is sufficient to destabilize phylogenetic relationships and reduce our ability to diagnose species. Periodic revision of taxa is necessary to ensure their stability through time as new specimens reveal new information or new techniques or interpretations reveal previously undescribed or previously unsupported patterns in the evolutionary history of a clade. Of 15 named metoposaurid taxa from North America, eight are typically recognized as diagnostic or potentially diagnostic: four from the Popo Agie Formation of western Wyoming and four from the Dockum Group of west Texas and east-central New Mexico (Fig. 1). Recent advances in radioisotopic dating of non-marine Triassic strata of the western USA and improved lithostratigraphic and biostratigraphic correlations have helped to refine the occurrences of the eight specimens upon which these metoposaurid taxa are defined as well as numerous other metoposaurid occurrences (Lovelace et al., 2024, 2025; Martz & Parker, 2017; Ramezani, Fastovsky & Bowring, 2014; Rasmussen et al., 2021).

Despite the commonality of metoposaurids in Upper Triassic non-marine sediments, little is understood of their ontogenetic sequence(s) or heterochronic shifts through their evolutionary history. For example, *Apachesaurus gregorii* has been the subject of some recent studies attempting to characterize morphological variation of the taxon and, by extension, ontogenetic variation (Gee & Parker, 2020; Rinehart & Lucas, 2018; Spielmann & Lucas, 2012). An undeveloped tabular horn and otic notch are commonly cited as diagnostic characters of *A. gregorii* (*e.g*., Hunt, 1993; Rinehart & Lucas, 2018; Spielmann & Lucas, 2012), but morphometric analyses of a population of metoposaurids from the Lamy amphibian quarry suggest that relative lengthening of the tabular horn and/or deepening of the otic notch would be expected in metoposaurid ontogeny (Rinehart, Lucas & Heckert, 2024). Indeed, elongate intercentra—considered characteristic of *Apachesaurus*—exhibit histological and morphological features indicative of an earlier ontogenetic stage including a higher abundance of calcified cartilage and an open notochordal canal that, in some specimens, shows the onset of closure (Gee, Parker & Marsh, 2017; Gee & Parker, 2020). Taken together, these data suggest that *Apachesaurus* may be entirely represented by juvenile specimens, and this raises the question of whether *A. gregorii* is simply a juvenile of a pre-existing large-bodied metoposaurid taxon, a juvenile of a separate taxon, or if indeed all referred material of *Apachesaurus* is diagnostic to the level of genus or species. Thus far, no cranial specimens larger than YPM 4201 from the type locality have been referred to *Apachesaurus*. In addition, identification, description, and incorporation of juvenile specimens of other metoposaurid species may elucidate potential ontogenetically variable characters that could be shared with *Apachesaurus* further clarifying ontogenetic sequences in metoposaurids and adding to the body of literature on Triassic stereospondyls.

In the present study, a specimen-level phylogenetic and descriptive analysis of more than 50 North American metoposaurid crania including all name-bearing specimens diagnostic to the level of species helps to reveal the validity of metoposaurid taxa and assign referred specimens with a more rigorous taxonomic framework. These results suggest that the longstanding synonymy of specimens referred to *Anaschisma browni* (or junior synonyms therein) should not be maintained. Two novel genera are proposed to encompass the species *Paranaschisma* gen. nov. *howardensis* and *Tecovaserpeton* gen. nov. *perfectum* that can be differentiated from *Anaschisma browni* by several apomorphies and a unique combination of characters, respectively.

## Materials and Methods

### Examined specimens, photography, and image processing

Metoposaurid specimens examined in this study were observed firsthand except for the following: specimens of *Metoposaurus krasiejowensis* (ZPAL) and YPM VPPU 021742 but a cast of the latter was observed (TMM 41742.1). All specimens included in the phylogenetic analyses are listed in Table 1. Following best taxonomic practices, photographs of most specimens are provided, and if photographs are not provided here, specimen numbers and/or direct reference to previously published figures are provided (Bianchi & Gonçalves, 2021). Most specimens were photographed with a Nikon D3500 DSLR camera (Nikon, Tokyo, Japan), but some UWGM specimens were photographed with a Canon EOS 6D (Canon Inc., Tokyo, Japan). Color correction was performed in Adobe Lightroom. For tall specimens which required multiple focal depths to capture the full specimen, photographs were focus stacked in Adobe Photoshop (Adobe Corp., San Jose, VA, USA) using default scripts. Figures were compiled in Adobe Illustrator (Adobe Corp., San Jose, VA, USA).

**Table 1:** All specimens included in the phylogenetic analyses with type status, locality, stratigraphic provenance, and reference if not observed firsthand.

Specimen Number	Taxon	Type Status (if applicable)	Locality	Group	Formation	Member	Reference (if applicable)
UC 447	*Anaschisma browni*	Holotype	Willow Creek	Chugwater	Popo Agie	“ochre unit”	
UC 448	“*Anaschisma brachygnatha*” (=*Anaschisma browni*)	Holotype	Willow Creek	Chugwater	Popo Agie	“ochre unit”	
MU 517	“*Boborophagus wyomingensis*” (=*Anaschisma browni*)	Holotype	Sage Creek	Chugwater	Popo Agie	“ochre unit”	
MU 537	“*Koskinonodon princeps*” (=*Anaschisma browni*)	Holotype	Bull Lake Creek	Chugwater	Popo Agie	“purple-ochre transition”?	
MU 527	“*Koskinonodon princeps*” (=*Anaschisma browni*)	Paratype	Bull Lake Creek	Chugwater	Popo Agie	“purple-ochre transition”?	
UCMP 66991	*Paranaschisma howardensis*	N/A	Otis Chalk Quarry 3	Dockum	Colorado City		
TMM 31100-19	*Paranaschisma howardensis*	N/A	Otis Chalk Quarry 3	Dockum	Colorado City		
TMM 31100-30	*Paranaschisma howardensis*	Lectotype	Otis Chalk Quarry 3	Dockum	Colorado City		
TMM 31100-42	*Paranaschisma howardensis*	Syntype	Otis Chalk Quarry 3	Dockum	Colorado City		
TMM 31100-122	*Paranaschisma howardensis*	Syntype	Otis Chalk Quarry 3	Dockum	Colorado City		
TMM 31100-124	*Paranaschisma howardensis*	Syntype	Otis Chalk Quarry 3	Dockum	Colorado City		
TMM 31100-431	*Paranaschisma howardensis*	Syntype	Otis Chalk Quarry 3	Dockum	Colorado City		
UMMP 21326	*cf. Paranaschisma howardensis*	N/A	Cerita de la Cruz Creek	Dockum	Tecovas		
TTU-P09044	“*Buettneria calgariensis*” (=*Tecovaserpeton perfectum*)	Holotype	MOTT VPL 0696	Dockum	Tecovas		
TTU-P09045	*Tecovaserpeton perfectum*	N/A	MOTT VPL 0000 (Unknown)	Dockum	Unknown		
UCMP 113554	*Tecovaserpeton perfectum*	N/A	UCMP V6333	Dockum	N/A	N/A	
UMMP 7475	*Tecovaserpeton perfectum*	Holotype	Sand Creek Breaks	Dockum	Tecovas	N/A	
PPHM 1 (WT 3114)	*Tecovaserpeton perfectum*	N/A	Rotten Hill	Dockum	Tecovas	N/A	
PPHM 4	*Tecovaserpeton perfectum*	N/A	Rotten Hill	Dockum	Tecovas	N/A	
PPHM 6	*Tecovaserpeton perfectum*	N/A	Rotten Hill	Dockum	Tecovas	N/A	
PPHM 9	*Tecovaserpeton perfectum*	N/A	Rotten Hill	Dockum	Tecovas	N/A	
WT 3011	*Tecovaserpeton perfectum*	N/A	Rotten Hill	Dockum	Tecovas	N/A	
WT 3011-1	*Tecovaserpeton perfectum*	N/A	Rotten Hill	Dockum	Tecovas	N/A	
WT 3011-3	*Tecovaserpeton perfectum*	N/A	Rotten Hill	Dockum	Tecovas	N/A	
WT 3055	*Tecovaserpeton perfectum*	N/A	Rotten Hill	Dockum	Tecovas	N/A	
WT 3057	*Tecovaserpeton perfectum*	N/A	Rotten Hill	Dockum	Tecovas	N/A	
WT 3067-1	*Tecovaserpeton perfectum*	N/A	Rotten Hill	Dockum	Tecovas	N/A	
WT 3068-2	*Tecovaserpeton perfectum*	N/A	Rotten Hill	Dockum	Tecovas	N/A	
WT 3166-1	*Tecovaserpeton perfectum*	N/A	Rotten Hill	Dockum	Tecovas	N/A	
UCMP 27103	*cf. Tecovaserpeton perfectum*	N/A	PF 124 (UCMP 7038 on collection tag)	N/A	Chinle	lower Petrified Forest	
UMMP 8854	*cf. Tecovaserpeton perfectum*	N/A	Head of Holmes Creek	Dockum	Tecovas?		
YPM 4201	*Apachesaurus gregorii*	N/A	YPM 6649	Dockum	Redonda		
UCMP 63845	*Apachesaurus gregorii*	Holotype	UCMP V6148	Dockum	Redonda		
UCMP 63846	*Apachesaurus gregorii*	Paratype	UCMP V6148	Dockum	Redonda		
UCMP 171591	*Apachesaurus gregorii*	N/A	UCMP V82250	N/A	Chinle	lower Petrified Forest	
UCMP 27051	*cf. Apachesaurus gregorii*	N/A	UCMP 7307	N/A	Chinle	lower Petrified Forest	
UMMP 13055	*Buettnererpeton bakeri*	Holotype	Elkins Place	Dockum	Camp Springs Conglomerate		
UMMP 13820	*Buettnererpeton bakeri*	Paratype	Elkins Place	Dockum	Camp Springs Conglomerate		
MCZ 1054 (formerly UMMP 13821)	*Buettnererpeton bakeri*	Plesiotype	Elkins Place	Dockum	Camp Springs Conglomerate		
UMMP 13822	*Buettnererpeton bakeri*	Paratype	Elkins Place	Dockum	Camp Springs Conglomerate		
UMMP 13823	*Buettnererpeton bakeri*	Paratype	Elkins Place	Dockum	Camp Springs Conglomerate		
UWGM 7211	*Buettnererpeton bakeri*	N/A	Nobby Knob	Chugwater	Popo Agie	“purple unit”	
UWGM 7212	*Buettnererpeton bakeri*	N/A	Nobby Knob	Chugwater	Popo Agie	“purple unit”	
UWGM 7275	*Buettnererpeton bakeri*	N/A	Nobby Knob	Chugwater	Popo Agie	“purple unit”	
UWGM 7336	*Buettnererpeton bakeri*	N/A	Nobby Knob	Chugwater	Popo Agie	“purple unit”	
TTU-P10530	*Buettnererpeton bakeri*	N/A	Boren Ranch	Dockum	lower Cooper Canyon	overlying Boren Ranch Sandstone	
TTU-P11047	*Buettnererpeton bakeri*	N/A	Boren Ranch	Dockum	lower Cooper Canyon	overlying Boren Ranch Sandstone	
TMM 31099-12	*Buettnererpeton bakeri*	N/A	Otis Chalk Quarry 2	Dockum	Colorado City		
TMM 31099-128	*Buettnererpeton bakeri*	N/A	Otis Chalk Quarry 2	Dockum	Colorado City		
ZPAL AbIII/3	*Metoposaurus krasiejowensis*	N/A	Krasiejów		Drawno beds		Sulej (2007)
ZPAL AbIII/358	*Metoposaurus krasiejowensis*	Holotype	Krasiejów		Drawno beds		Sulej (2007)
ZPAL AbIII/682	*Metoposaurus krasiejowensis*	N/A	Krasiejów		Drawno beds		Sulej (2007)
ZPAL AbIII/683	*Metoposaurus krasiejowensis*	N/A	Krasiejów		Drawno beds		Sulej (2007)
ZPAL AbIII/894	*Metoposaurus krasiejowensis*	N/A	Krasiejów		Drawno beds		Sulej (2007)
ZPAL AbIII/1192	*Metoposaurus krasiejowensis*	N/A	Krasiejów		Drawno beds		Sulej (2007)
ZPAL AbIII/1683	*Metoposaurus krasiejowensis*	N/A	Krasiejów		Drawno beds		Sulej (2007)
UCMP 31800	Metoposauridae indet.	N/A	UCMP 7307	N/A	Chinle	lower Petrified Forest	
UCMP 35006	Metoposauridae indet.	N/A	UCMP 7307	N/A	Chinle	lower Petrified Forest	
UCMP 27024	Metoposauridae indet.	N/A	UCMP 7046A	N/A	Chinle	lower Petrified Forest	
UCMP 27039	Metoposauridae indet.	N/A	UCMP 7307	N/A	Chinle	lower Petrified Forest	
TMM 31220-1	Metoposauridae indet.	N/A	Otis Chalk Quarry 4	Dockum	Colorado City		
YPM VPPU 021742 (cast observed TMM 41742.1)	Metoposauridae indet.	N/A	Noel Head	Newark (Supergroup)	Wolfville	Evangeline	

### Phylogenetic analysis

Phylogenetic inference was performed using a modified version of the matrix of Gee & Kufner (2022). Seven non-metoposaurid stereospondylomorphs were added to the matrix to improve outgroup sampling (*Archegosaurus decheni*, *Mastodonsaurus cappelensis*, *Ninumbeehan dookoodukah*, *Rhineceps karibaensis*, *Thoosuchus yakovlevi*, and *Wetlugasaurus angustifrons*). Two of these species (*M. cappelensis* and *R. karibaensis*) were included as they have been recently considered congeneric with species already included in the character matrix and allow for a comparison with potentially congeneric metoposaurids (Schoch et al., 2023; Steyer & Sidor, 2025). Scores for mandibles and postcrania were included for each specimen based on the aggregate conditions found in each locality if the elements could be reasonably assumed to be from a similar sized individual (*e.g*., if a skull from a large individual and only small postcrania were recovered, the postcranial characters were considered unknown). *Sclerocephalus haeuseri* was designated as the outgroup in all analyses.

During the study, characters 95, 114, and 124 were recognized as problematic and consequently removed as they could not be readily modified without significant revision beyond the scope of this study. The rationale for excluding each of the problematic characters is further elaborated in the Discussion. Characters 46, 50, 86, 127, and 165 were likely ontogenetically variable and were removed from some analyses to determine if they affected the groupings of small- and large-bodied metoposaurid specimens. Characters 10, 16, 19, 20, 27, 46, 59, 66, 75, 84, 91, 93, 98, 106, 118, 120, and 136 were modified for this study. Five states were added to preexisting characters: 52:2, 75:2, 91:1, 118:1, and 119:2. Twenty-nine new characters were appended to the character list and matrix as either novel characters, splitting of compound characters, or characters from other studies. Multi-state characters that could be reasonably inferred to exist along a morphocline were ordered in all analyses following a previous analysis with this matrix (Gee & Kufner, 2022). Comparisons of taxonomically relevant character states are provided in Table 2. Character and character-score modifications with rationale are recorded in File S1.

**Table 2:** Character-state comparisons between North American and Moroccan metoposaurids.

	*Dutuitosaurus ouazzoui*	*Arganasaurus lyazidi*	*Buettnererpeton bakeri*	*Anaschisma browni*	*Paranaschisma howardensis*	*Tecovaserpeton perfectum*	*Apachesaurus gregorii*
Lacrimal contact with orbital margin	Absent*	Absent*	Absent*	Present**	Present**	Present**	Absent in juveniles, present in adults (?)
Lacrimal length	Elongate*	Elongate*	Elongate*	Elongate*	Elongate (some short)	Short**	Short**
Prefrontal, anterior end	Pointed*	Pointed*	Pointed*	Blunt (in large individuals) and pointed (in the smallest specimen)	Blunt (with a few pointed on one side of the skull)	Pointed and Blunt (no clear size-related differences)	Blunt (in small specimens) and pointed (in large referred skull)
Prefrontal, anterior end (angle between medial and lateral margins)	Narrow and wide*	Narrow and wide*	Narrow and wide*	Narrow (in large holotype specimen) and wide (in the smallest specimen); variable in the largest referred specimen	Wide**	Wide**	Wide**
Orbit position	Anterior orbit margin well posterior to anterior margin of interpterygoid vacuity*	Anterior orbit margin well posterior to anterior margin of interpterygoid vacuity*	Anterior orbit margin well posterior to anterior margin of interpterygoid vacuity*	Anterior orbit margin at or slightly posterior to anterior margin of interpterygoid vacuity**	Anterior orbit margin at or slightly posterior to anterior margin of interpterygoid vacuity**	Anterior orbit margin at or anterior to the anterior margin of interpterygoid vacuity	Anterior orbit margin well posterior to anterior margin of interpterygoid vacuity in juveniles, more anterior in adults (?)
Squamosal (lateral margin)	Stepped (pentagonal in outline)*	Gentle curve (subtriangular in outline)**	Gentle curve (subtriangular in outline)**	Stepped (pentagonal in outline)*	Stepped (pentagonal in outline)*	Stepped (pentagonal in outline)*	Stepped (pentagonal in outline)*
Tabular shape	Square, with anterior point between the squamosal and the supratemporal*	Short, subrectangular tabular lacking an anterior point between the squamosal and the supratemporal**	Square, with anterior point between the squamosal and the supratemporal (one short and subrectangular)*	Square, with anterior point between the squamosal and the supratemporal**	Square, with anterior point between the squamosal and the supratemporal**	Square, with anterior point between the squamosal and the supratemporal**	Square, with anterior point between the squamosal and the supratemporal**
Tabular horn	Present*	Present*	Present*	Present*	Present*	Present*	Absent in juveniles, present in adults(?)
Otic notch	Deep and narrow*	Relatively shallow and wide	Deep and narrow*	Deep and narrow*	Deep and narrow (wide in some specimens)	Deep and narrow*	Shallow and wide in juveniles
Quadrate process of pterygoid	Relatively flat*	Relatively flat*	Relatively flat*	Relatively flat*	Relatively flat*	Relatively flat*	Bent at 90°
Lamellose process	Present, offset from occipital pillar	Unknown	Present, offset from occipital pillar (confluent in the smallest specimens)	Present, as raised ridge confluent with occipital pillar	Present, offset from occipital pillar, counterparts nearly contact at the midline	Present, as raised ridge confluent with occipital pillar, and offset from occipital pillar (no clear size differences)	Present, as raised ridge confluent with occipital pillar
Contribution of ectopterygoid to palatal ramus	Absent*	Absent*	Absent*	Absent*	Present**	Absent*	Present**
Cultriform process (width)	Broader than the palatal ramus of the pterygoid*	Broader than the palatal ramus of the pterygoid*	Subequal to the palatal ramus of the pterygoid, broader in one small specimen, and narrower in the largest specimens	Both subequal to and broader than the palatal ramus of the pterygoid	Broader than the palatal ramus of the pterygoid*	Both narrower and subequal to the palatal ramus of the pterygoid	Both narrower (adults) and subequal to (juveniles) the palatal ramus of the pterygoid
Cultriform process (ornamentation)	Absent*	Absent*	Present, but faint**	Present, but faint**	Present in at least some specimens, and pronounced when present	Present and absent with no clear size related pattern	Unknown
Supraorbital sulcus-lacrimal contact	Present*	Present*	Present and absent (always present in the type locality and primarily absent outside of type locality)	Present and absent (primarily absent)**	Present and absent (primarily absent)**	Present and absent (primarily present)	Absent
Transverse occipital sulcus	Absent*	Absent*	Absent (or incipient)*	Absent*	Present**	Present**	Absent in juveniles, present in adults (?)
Accessory jugal sulcus	Absent*	Absent*	Present**	Absent*	Present**	Present**	Absent in juveniles, present in adults (?)
Presacral trunk intercentra (proportions)	Not anteroposteriorly shortened*	Unknown	Anteroposteriorly shortened**	Anteroposteriorly shortened**	Anteroposteriorly shortened**	Anteroposteriorly shortened in adult intercentra	Not anteroposteriorly shortened*
Notochordal canal or pit	Dorsal to center of intercentra*	Unknown	Dorsal to center of intercentra*	Dorsal to center of intercentra*	Dorsal to center of intercentra*	At center of intercentrum**	At center of intercentrum**
Clavicle ornamentation	Small region of reticulate ornamentation*	Large region of reticulate ornamentation**	Large region of reticulate ornamentation**	Large region of reticulate ornamentation**	Large region of reticulate ornamentation**	Large region of reticulate ornamentation**	Large region of reticulate ornamentation**
Sensory sulcus on posterolateral margin of the clavicle	Present*	Present*	Present*	Absent**	Present and absent	Absent**	Absent**
Apomorphies (within Metoposauridae)	Ossified sphenethmoid and ossified trunk pleurocentra	Narrow, widely spaced occipital condyles; short, subrectangular tabular lacking an anterior point between the squamosal and the supratemporal	N/A	N/A	Long *insula jugalis* occupying more than half of the transverse width of the palatal ramus of the pterygoid; very pronounced lamellose processes almost contacting at the midline	Dorsal shaft of ilium without anterior convexity or flexure (straight in lateral view)	Temporal sulcus terminates on the squamosal (in part)

**Note:**

The plesiomorphic condition assuming *Dutuitosaurus* is the earliest diverging metoposaurid is marked by a single asterisk (*), a shared derived condition is marked by two asterisks (**), and states without an asterisk are unique with no clear correspondence across taxa.

Phylogenetic analyses were conducted with maximum parsimony in TNT v. 1.6 (Goloboff & Morales, 2023) and with Bayesian inference in MrBayes v. 3.2.7 (Ronquist et al., 2012). Parsimony searches conducted in TNT were done using equal weights according to the tree search methods outlined by Hartman et al. (2019) and Smith (2019). Bremer decay indices were calculated for each strict (Nelson) consensus tree using the “BREMER.RUN” script provided with TNT v. 1.6, and retention and consistency indices were calculated using the “STATS.RUN” script. Priors in the MrBayes runs were set to a General Time Reversible (GTR) substitution model (nst = 6) with a gamma distribution of rates with default coding parameters (*i.e*., no constant character states) over 100,000,000 iterations and 25% of trees (25,000,000) discarded as burn-in. A summary tree was computed with consensus type set to “halfcompat” collapsing nodes with less than 0.5 posterior probability with these nodes considered unresolved following a recent study demonstrating the efficacy of majority-rule consensus trees in recovering correct nodes with simulated data (O’Reilly et al., 2016; O’Reilly & Donoghue, 2018).

### Nomenclatural acts

The electronic version of this article in Portable Document Format (PDF) will represent a published work according to the International Commission on Zoological Nomenclature (ICZN), and hence the new names contained in the electronic version are effectively published under that Code from the electronic edition alone. This published work and the nomenclatural acts it contains have been registered in ZooBank, the online registration system for the ICZN. The ZooBank Life Science Identifiers (LSIDs) can be resolved and the associated information viewed through any standard web browser by appending the LSID to the prefix http://zoobank.org/. The LSID for this publication is: urn:lsid:zoobank.org:pub:CF860616-7B8B-4815-90E7-1973FC686972. The online version of this work is archived and available from the following digital repositories: PeerJ, PubMed Central SCIE, and CLOCKSS.

**Systematic Paleontology and Description**

TEMNOSPONDYLI Zittel, 1887–1890 *sensu* Schoch, 2013

STEREOSPONDYLI Zittel, 1887–1890 *sensu* Yates & Warren, 2000

TREMATOSAURIA Yates & Warren, 2000

METOPOSAURIDAE Watson, 1919 *sensu* Buffa, Jalil & Steyer, 2019

*Arganasaurus* Hunt, 1993

Diagnosis—. As for species by monotypy.

*Arganasaurus lyazidi* Dutuit, 1976b *sensu* Hunt, 1993

Figure 2

**Figure 2:**
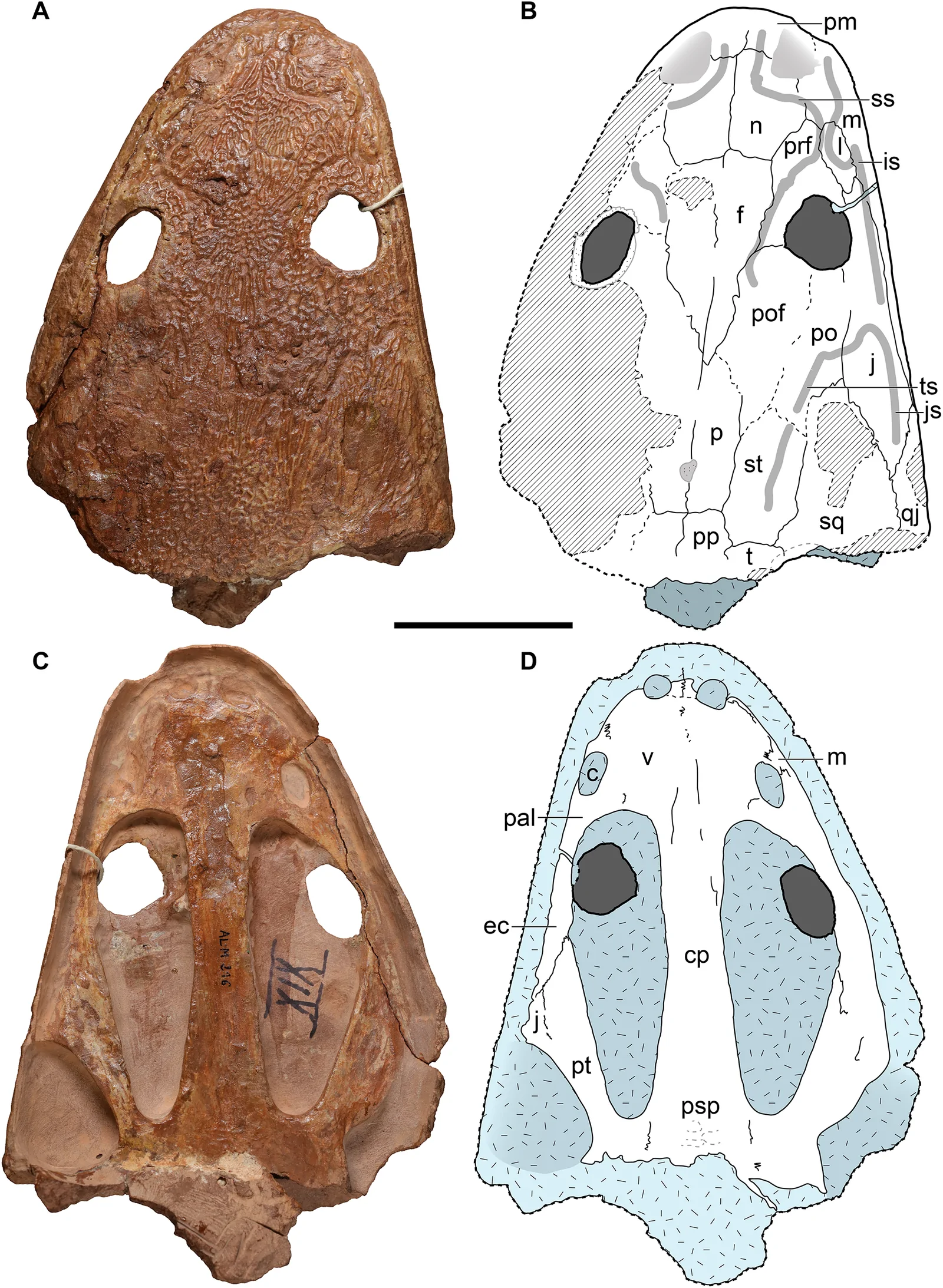
Representative partial cranium of *Arganasaurus lyazidi*, MNHN-F-ALM216. (A) Photograph in dorsal view; (B) interpretive drawing of the same; (C) photograph in ventral view; (D) interpretive drawing of the same. Blue hatching represents reconstruction; diagonal lines represent broken surfaces; fine dashed gray lines represent topographic details like ridges. Abbreviations: c, choana; cp, cultriform process; ec, ectopterygoid; f, frontal; is, infraorbital sulcus; j, jugal; js, jugal sulcus; l, lacrimal; m, maxilla; n, nasal; p, parietal; pal, palatine; pm, premaxilla; po, postorbital; pof, postfrontal; pp, postparietal; prf, prefrontal; psp, parasphenoid; pt, pterygoid; qj, quadratojugal; sq, squamosal; ss, supraorbital sulcus; st, supratemporal; t, tabular; ts, temporal sulcus; v, vomer. Scale bar equals 5 cm.

Synonyms—. *Metoposaurus lyazidi* Dutuit, 1976b

Emended Diagnosis—. Metoposaurid tentatively differentiated from all other metoposaurids by narrow occipital condyles that are widely spaced (*i.e*., the basal plate of the parasphenoid is less completely ossified) and a short, subrectangular tabular lacking an anterior point between the squamosal and the supratemporal (shared with some specimens of *Buettnererpeton bakeri*). Further differentiated from all other metoposaurids except *Apachesaurus gregorii* (in part), *Dutuitosaurus ouazzoui*, and *Buettnererpeton bakeri* by a lacrimal that does not enter the orbital margin. Tentatively differentiated from *B. bakeri* by the absence of an accessory jugal sulcus. Further differentiated from *D. ouazzoui* by the absence of an ossified sphenethmoid. Further differentiated from *A. gregorii* by a broad cultriform process, a temporal sulcus that terminates on the supratemporal, a dorsally positioned notochordal canal throughout the axial column, and the presence of a sensory sulcus on the posterolateral margin of the clavicle.

Remarks—. Without a doubt the specimens of *Ar. lyazidi* were plastically deformed, but a consistent pattern is present between all the specimens: the narrow occipital condyles with a wide space between them (see pls. xlix–l: Dutuit, 1976b). The spacing between the occipital condyles may be ontogenetically influenced based on other small-bodied metoposaurid specimens with less ossification of the basal plate between the exoccipitals. However, the narrow occipital condyles are not seen in other purported juvenile specimens (see below) and may only be present due to overpreparation in one large North American specimen (UCMP 27024: Metoposauridae indet.). *Arganasaurus lyazidi* had previously been differentiated from all other metoposaurids by an apomorphic lacrimal contribution to the margin of the external naris (Hunt, 1993). This condition could not be corroborated in any of the specimens the present author could observe, and indeed, a previous composite reconstruction of *Ar. lyazidi* (fig. 73: Sulej, 2007) is consistent with the present interpretation of a referred specimen: MNHN-F-ALM216 (Fig. 2). *Buettnererpeton bakeri*, *Dutuitosaurus ouazzoui*, and *Ar. lyazidi* are the only metoposaurid taxa that lack a lacrimal contribution to the orbital margin in total. Buffa, Jalil & Steyer (2019) united *Ar. lyazidi* and “*Arganasaurus*” *azerouali* with an emended diagnosis of: a posteroventrally angled occipital surface, a shallow otic notch with a “bulge-like” tabular horn, and a sub-triangular squamosal. A posteroventrally sloping occiput that is partially exposed in dorsal view is relatively commonplace among metoposaurids, but several North American specimens have a nearly vertical occiput including the holotypes of *A. gregorii* and *Anaschisma browni* and most specimens previously referred to *An. browni* (but see below). The shallow otic notch of *Ar. lyazidi* is more incised than in similarly-sized specimens of *A. gregorii* (pls. xlviii–xlix: Dutuit, 1976b), and at least one specimen of *Ar. lyazidi* (pl. xlix: Dutuit, 1976b) has a tabular horn that projects out more so than the “bulge-like” appearance seen in “*Ar*.” *azerouali* (fig. 1: Buffa, Jalil & Steyer, 2019). There may be allometry to consider in the relative proportions of the otic notch and tabular horn. A sub-triangular squamosal is shared between *Ar. lyazidi*, *B. bakeri*, and “*Ar*.” *azerouali*, but a lack of many of the other shared features and instability in the phylogenetic position of “*Ar*.” *azerouali* led to uncertainty in the generic assignment of this species in the present study. A redescription of *Ar. lyazidi* could recover further differential or apomorphic features of the species, but that is beyond the scope of this study.

*Buettnererpeton* Gee & Kufner, 2022

Diagnosis—. As for species by monotypy.

*Buettnererpeton bakeri* Case, 1931 *sensu* Gee & Kufner, 2022

Synonyms—. *Buettneria bakeri* Case, 1931

*Eupelor fraasi jonesi* (in part) Colbert & Imbrie, 1956

*Metoposaurus fraasi jonesi* (in part) RoyChowdhury, 1965

*Metoposaurus bakeri* Baird & Olsen, 1983; Hopson, 1984

*Metoposaurus fraasi* (in part) Murry, 1986

*Metoposaurus* (“*Buettneria*”) *howardensis* (in part) Murry, 1989

*Koskinonodon bakeri* Brusatte et al., 2015

Figures 3–19

**Figure 3:**
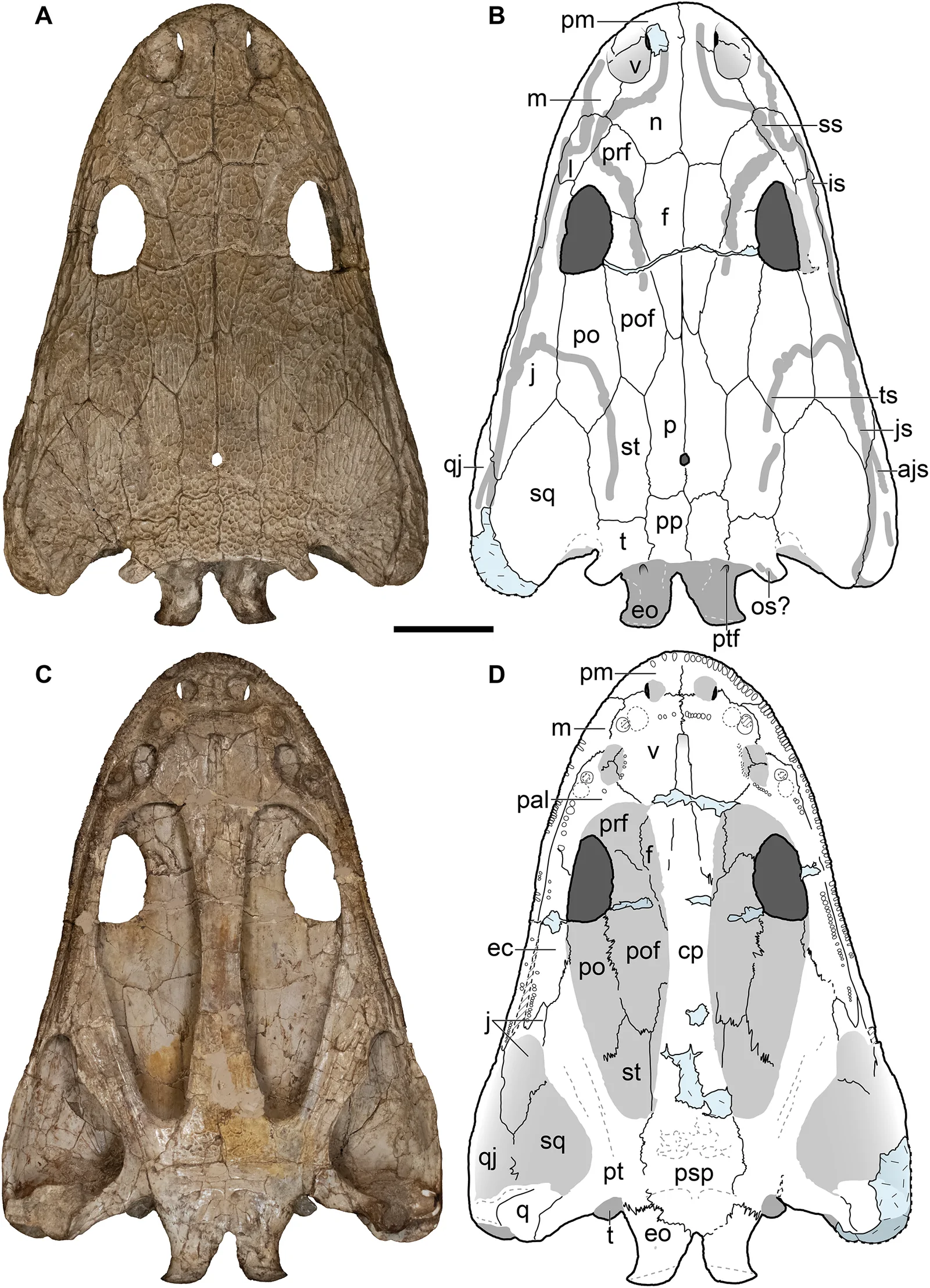
Plesiotype cranium of *Buettnererpeton bakeri*, MCZ 1054, from Elkins Place in the Camp Springs Conglomerate of Snyder County, TX. (A) Photograph in dorsal view; (B) interpretive drawing of the same; (C) photograph in ventral view; (D) interpretive drawing of the same. Blue hatching represents reconstruction; diagonal lines represent broken surfaces; fine dashed gray lines represent topographic details like ridges. Abbreviations: ajs, accessory jugal sulcus; cp, cultriform process; ec, ectopterygoid; eo, exoccipital; f, frontal; is, infraorbital sulcus; j, jugal; js, jugal sulcus; l, lacrimal; m, maxilla; n, nasal; os?, transverse occipital sulcus?; p, parietal; pal, palatine; pm, premaxilla; po, postorbital; pof, postfrontal; pp, postparietal; prf, prefrontal; psp, parasphenoid; pt, pterygoid; ptf, posttemporal fenestra; q, quadrate; qj, quadratojugal; sq, squamosal; ss, supraorbital sulcus; st, supratemporal; t, tabular; ts, temporal sulcus; v, vomer. Scale bar equals 5 cm.

**Figure 4:**
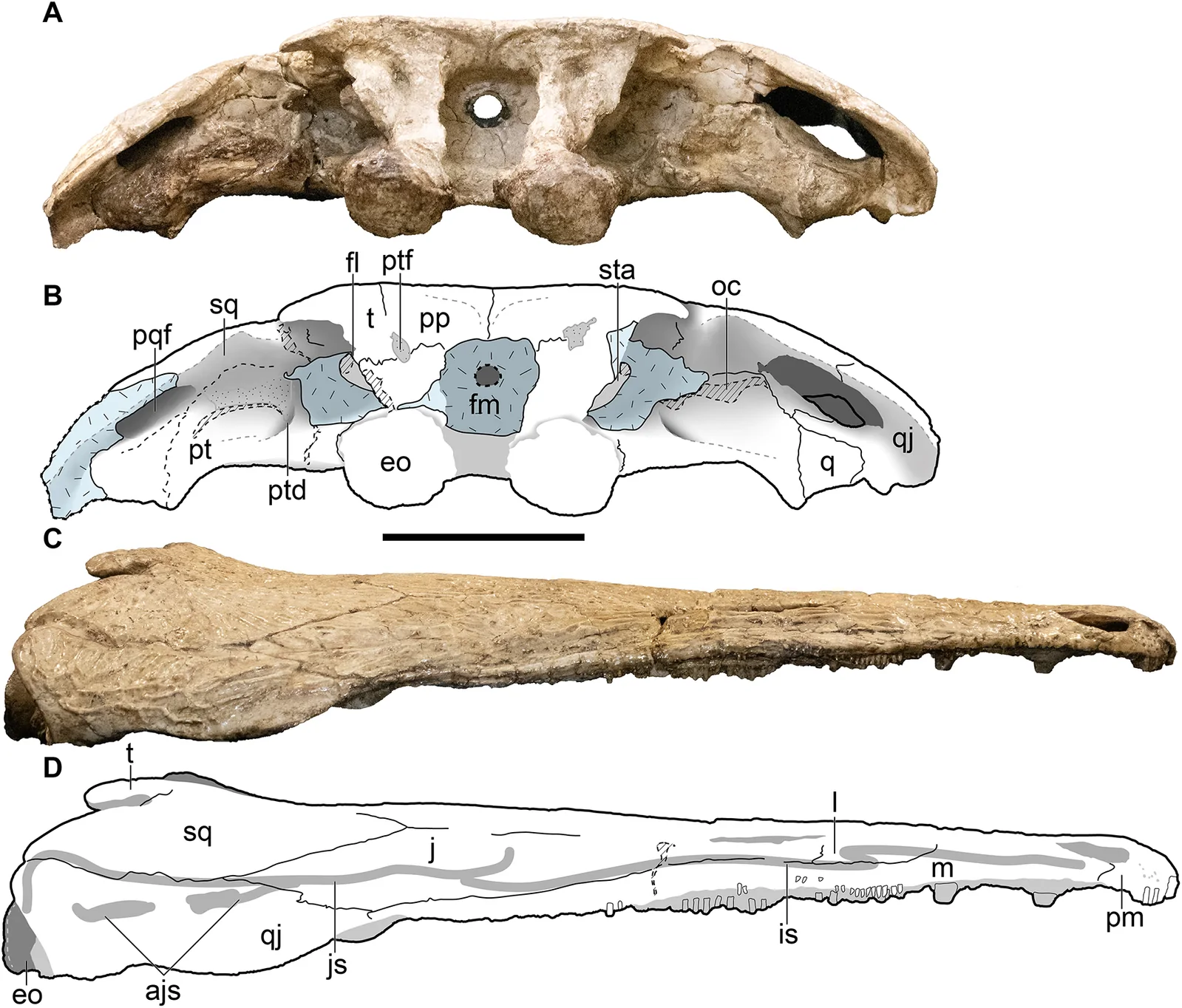
Plesiotype cranium of *Buettnererpeton bakeri*, MCZ 1054, from Elkins Place in the Camp Springs Conglomerate of Snyder County, TX. (A) Photograph in occipital view; (B) interpretive drawing of the same; (C) photograph in right lateral view; (D) interpretive drawing of the same. Blue hatching represents reconstruction; diagonal lines represent broken surfaces; fine dashed gray lines represent topographic details like ridges. Abbreviations: ajs, accessory jugal sulcus; eo, exoccipital; fl, flange on the parotic process of the tabular; fm, foramen magnum; is, infraorbital sulcus; j, jugal; js, jugal sulcus; l, lacrimal; m, maxilla; oc, oblique crest of the pterygoid (broken); pm, premaxilla; pp, postparietal; pqf, paraquadrate foramen; pt, pterygoid; ptd, pterygoid depression; ptf, posttemporal fenestra; q, quadrate; qj, quadratojugal; sq, squamosal; sta, stapes; t, tabular. Scale bar equals 5 cm.

**Figure 5:**
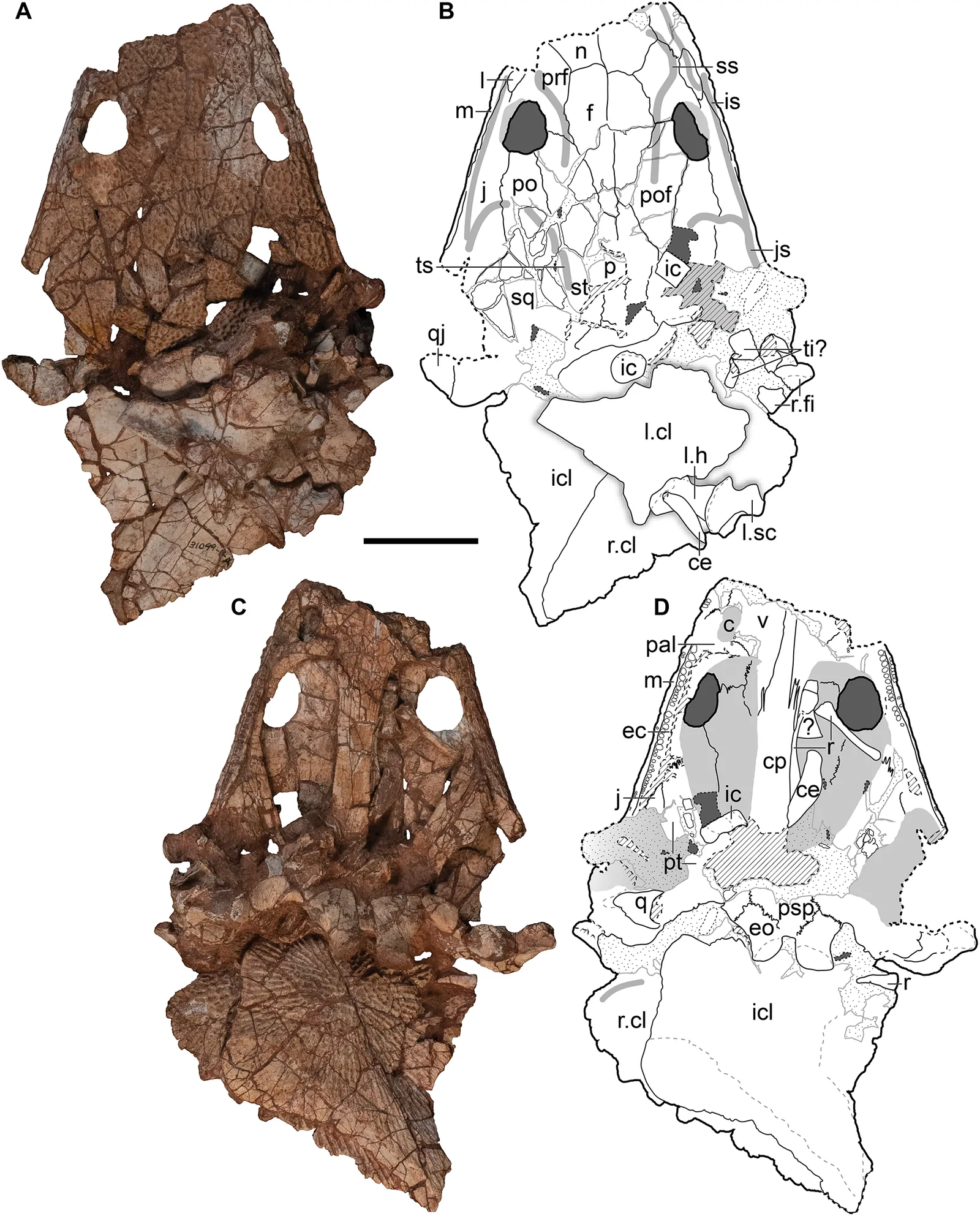
Referred partial skeleton of *Buettnererpeton bakeri*, TMM 31099-12, from Otis Chalk Quarry 2 in Howard County, TX in anatomical profiles relative to the cranium. (A) Photograph in dorsal view; (B) interpretive drawing of the same; (C) photograph in ventral view; (D) interpretive drawing of the same. Hatching represents reconstruction; stippling represents residual matrix; diagonal lines represent broken surfaces; fine dashed gray lines represent topographic details like ridges. Abbreviations: c, choana (internal naris); ce, cleithrum; cp, cultriform process; ec, ectopterygoid; eo, exoccipital; f, frontal; ic, intercentrum; icl, interclavicle; is, infraorbital sulcus; j, jugal; js, jugal sulcus; l, lacrimal; l.cl, left clavicle; l.h, left humerus; l.sc, left scapula; m, maxilla; n, nasal; p, parietal; pal, palatine; po, postorbital; pof, postfrontal; prf, prefrontal; psp, parasphenoid; pt, pterygoid; q, quadrate; qj, quadratojugal; r, rib; r.cl, right clavicle; r.fi, right fibula; sq, squamosal; ss, supraorbital sulcus; st, supratemporal; ti?, tibia?; ts, temporal sulcus; v, vomer; ?, unknown element. Scale bar equals 5 cm.

**Figure 6:**
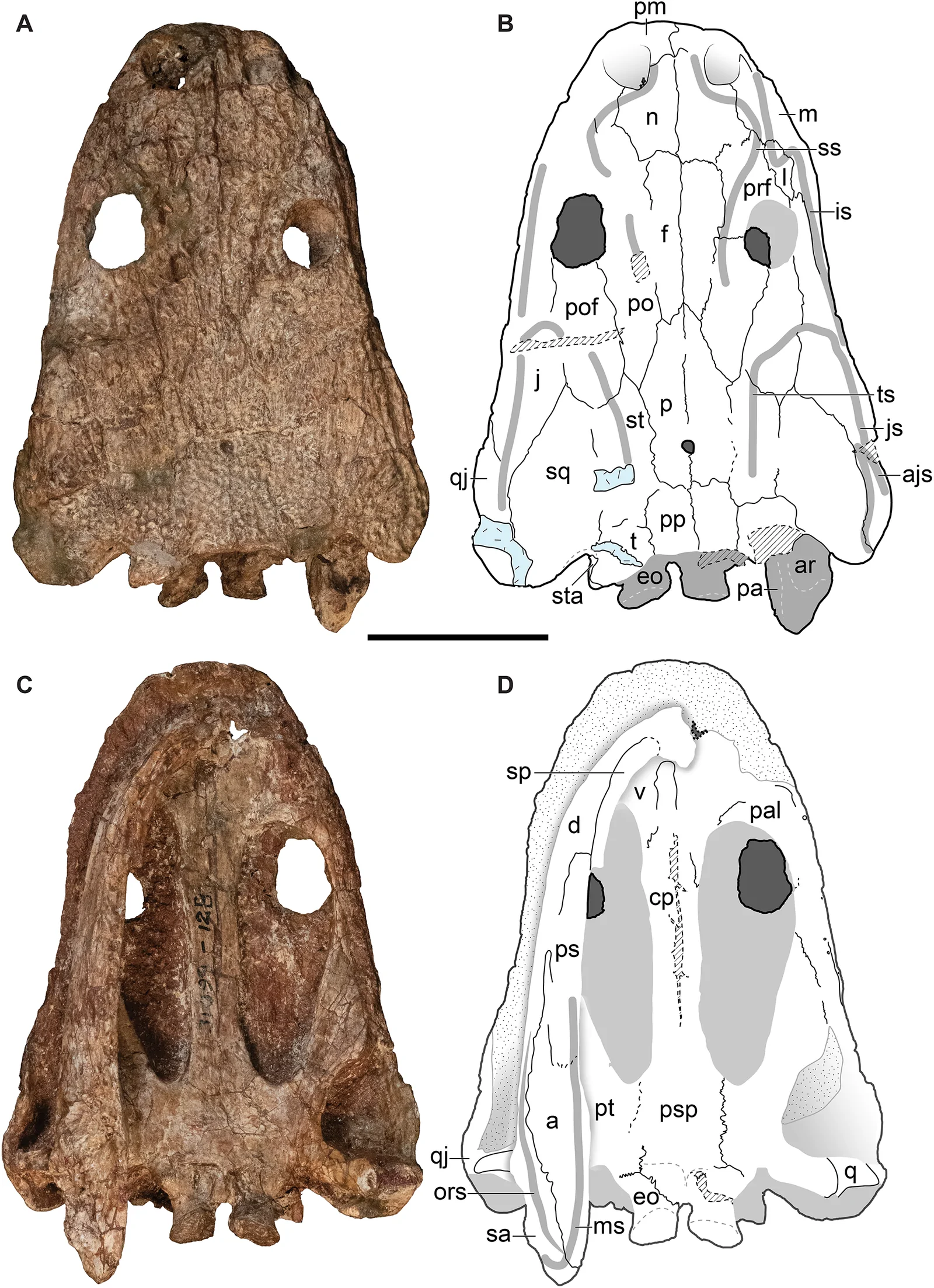
Referred cranium and right mandible of *Buettnererpeton bakeri*, TMM 31099-128, from Otis Chalk Quarry 2 in Howard County, TX. (A) Photograph in dorsal view; (B) interpretive drawing of the same; (C) photograph in ventral view; (D) interpretive drawing of the same. Blue hatching represents reconstruction; stippling represents residual matrix; diagonal lines represent broken surfaces; fine dashed gray lines represent topographic details like ridges. Abbreviations: a, angular; ajs, accessory jugal sulcus; ar, articular; cp, cultriform process; d, dentary; eo, exoccipital; f, frontal; is, infraorbital sulcus; j, jugal; js, jugal sulcus; l, lacrimal; m, maxilla; ms, mandibular sulcus; n, nasal; ors, oral sulcus; p, parietal; pa, prearticular; pal, palatine; pm, premaxilla; po, postorbital; pof, postfrontal; pp, postparietal; prf, prefrontal; ps, postpslenial; psp, parasphenoid; pt, pterygoid; q, quadrate; qj, quadratojugal; sa, surangular; sp, splenial; sq, squamosal; ss, supraorbital sulcus; st, supratemporal; sta, stapes; t, tabular; ts, temporal sulcus; v, vomer. Scale bar equals 5 cm.

**Figure 7:**
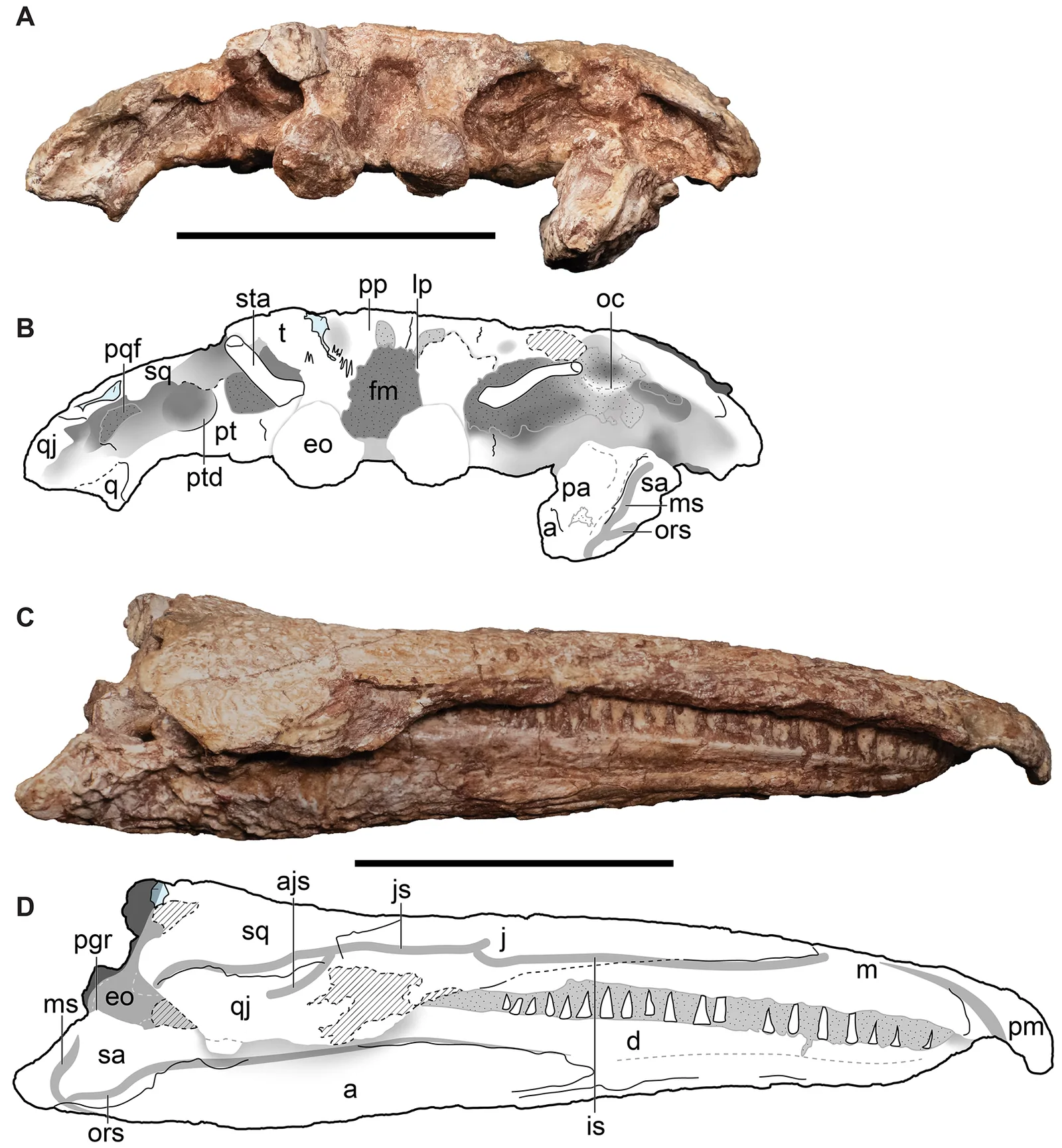
Referred cranium and right mandible of *Buettnererpeton bakeri*, TMM 31099-128, from Otis Chalk Quarry 2 in Howard County, TX. (A) Photograph in occipital view; (B) interpretive drawing of the same; (C) photograph in right lateral view; (D) interpretive drawing of the same. Hatching represents reconstruction; stippling represents residual matrix; diagonal lines represent broken surfaces; fine dashed gray lines represent topographic details like ridges. Abbreviations: a, angular; ajs, accessory jugal sulcus; d, dentary; eo, exoccipital; fm, foramen magnum; is, infraorbital sulcus; j, jugal; js, jugal sulcus; lp, lamellose process; m, maxilla; ms, mandibular sulcus; oc, oblique crest of the pterygoid; ors, oral sulcus; pa, prearticular; pgr, postglenoid ridge; pm, premaxilla; pp, postparietal; pqf, paraquadrate foramen; pt, pterygoid; ptd, pterygoid depression; q, quadrate; qj, quadratojugal; sa, surangular; sq, squamosal; sta, stapes; t, tabular. Scale bar equals 5 cm.

**Figure 8:**
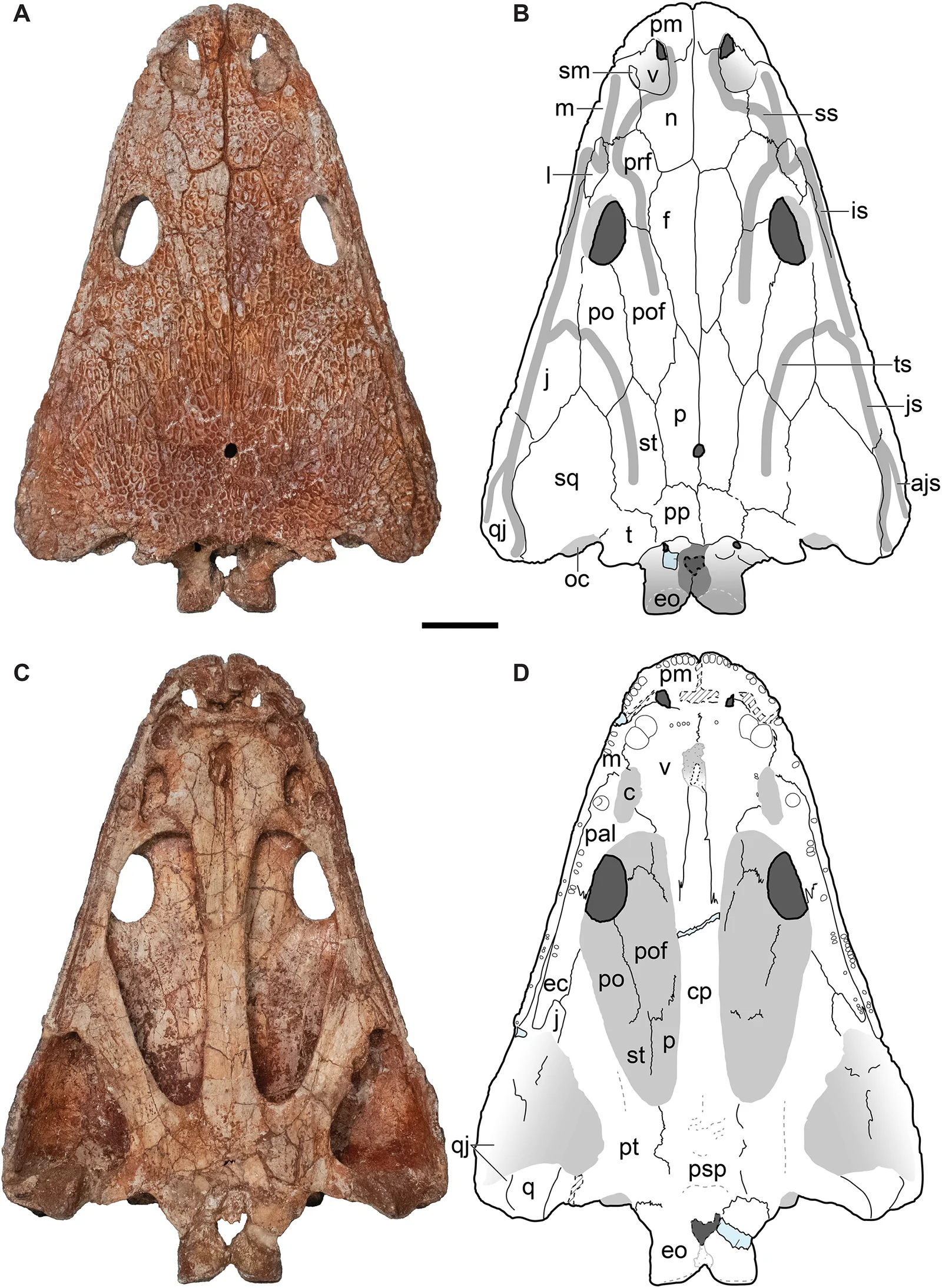
Referred cranium of *Buettnererpeton bakeri*, TTU-P10530, from the MOTT VPL 3869 (Boren Quarry) in Garza County, TX. (A) Photograph in dorsal view; (B) interpretive drawing of the same; (C) photograph in ventral view; (D) interpretive drawing of the same. Blue hatching represents reconstruction; stippling represents residual matrix; diagonal lines represent broken surfaces; fine dashed gray lines represent topographic details like ridges. Abbreviations: ajs, accessory jugal sulcus; c, choana (internal naris); cp, cultriform process; ec, ectopterygoid; eo, exoccipital; f, frontal; is, infraorbital sulcus; j, jugal; js, jugal sulcus; l, lacrimal; m, maxilla; n, nasal; oc, oblique crest of the pterygoid; p, parietal; pal, palatine; pm, premaxilla; po, postorbital; pof, postfrontal; pp, postparietal; prf, prefrontal; psp, parasphenoid; pt, pterygoid; q, quadrate; qj, quadratojugal; sm, septomaxilla; sq, squamosal; ss, supraorbital sulcus; st, supratemporal; t, tabular; ts, temporal sulcus; v, vomer. Scale bar equals 5 cm.

**Figure 9:**
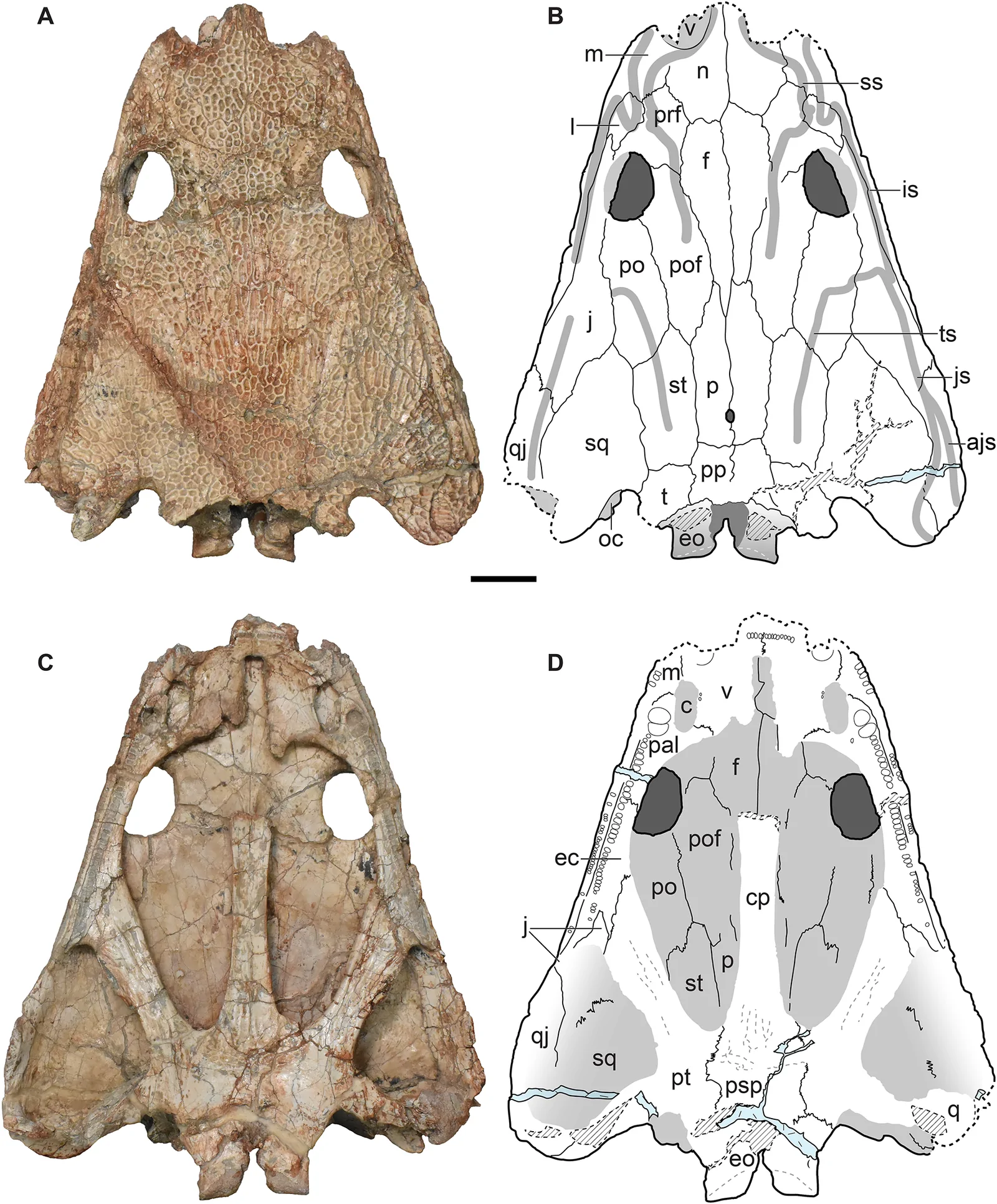
Referred cranium of *Buettnererpeton bakeri*, TTU-P11046, from MOTT VPL 3869 (Boren Quarry) in Garza County, TX. (A) Photograph in dorsal view; (B) interpretive drawing of the same; (C) photograph in ventral view; (D) interpretive drawing of the same. Blue hatching represents reconstruction; stippling represents residual matrix; diagonal lines represent broken surfaces; fine dashed gray lines represent topographic details like ridges. Abbreviations: ajs, accessory jugal sulcus; c, choana (internal naris); cp, cultriform process; ec, ectopterygoid; eo, exoccipital; f, frontal; is, infraorbital sulcus; j, jugal; js, jugal sulcus; l, lacrimal; m, maxilla; n, nasal; oc, oblique crest of the pterygoid; p, parietal; pal, palatine; po, postorbital; pof, postfrontal; pp, postparietal; prf, prefrontal; psp, parasphenoid; pt, pterygoid; q, quadrate; qj, quadratojugal; sq, squamosal; ss, supraorbital sulcus; st, supratemporal; t, tabular; ts, temporal sulcus; v, vomer. Scale bar equals 5 cm.

**Figure 10:**
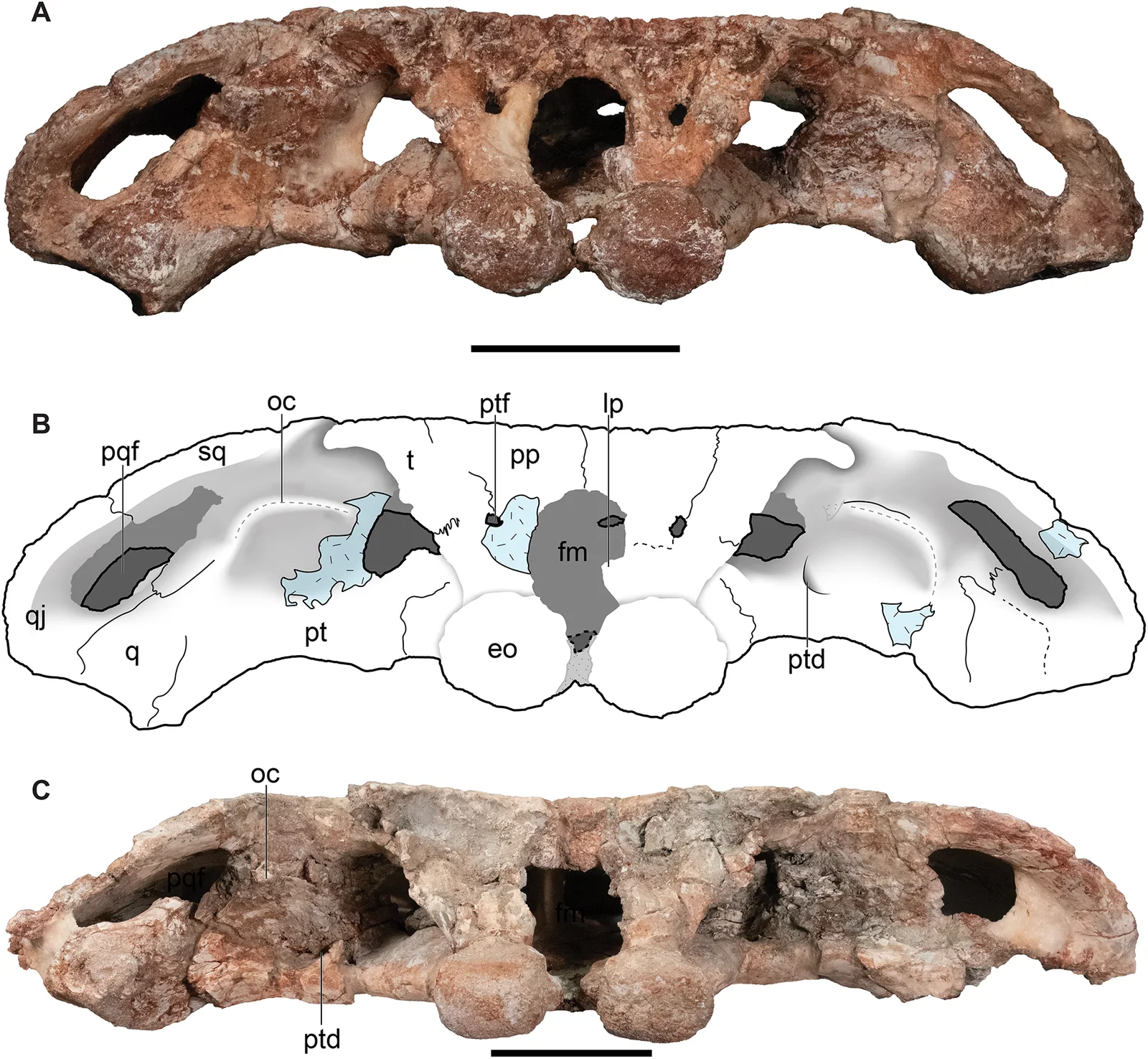
Referred crania of *Buettnererpeton bakeri*, TTU-P10530 (A, B) and TTU-P11046 (C), from the Boren Quarry in Garza County, TX in occipital view. (A) Photograph of TTU-P10530; (B) interpretive drawing of the same; (C) photograph of TTU-P11046. Hatching represents reconstruction; stippling represents residual matrix; fine dashed gray lines represent topographic details like ridges. Abbreviations: eo, exoccipital; fm, foramen magnum; lp, lamellose process; oc, oblique crest of the pterygoid; pp, postparietal; pqf, paraquadrate foramen; pt, pterygoid; ptd, pterygoid depression; ptf, posttemporal fenestra; q, quadrate; qj, quadratojugal; sq, squamosal; t, tabular. Upper scale bar for A and B; lower scale bar for C. Scale bars equal 5 cm.

**Figure 11:**
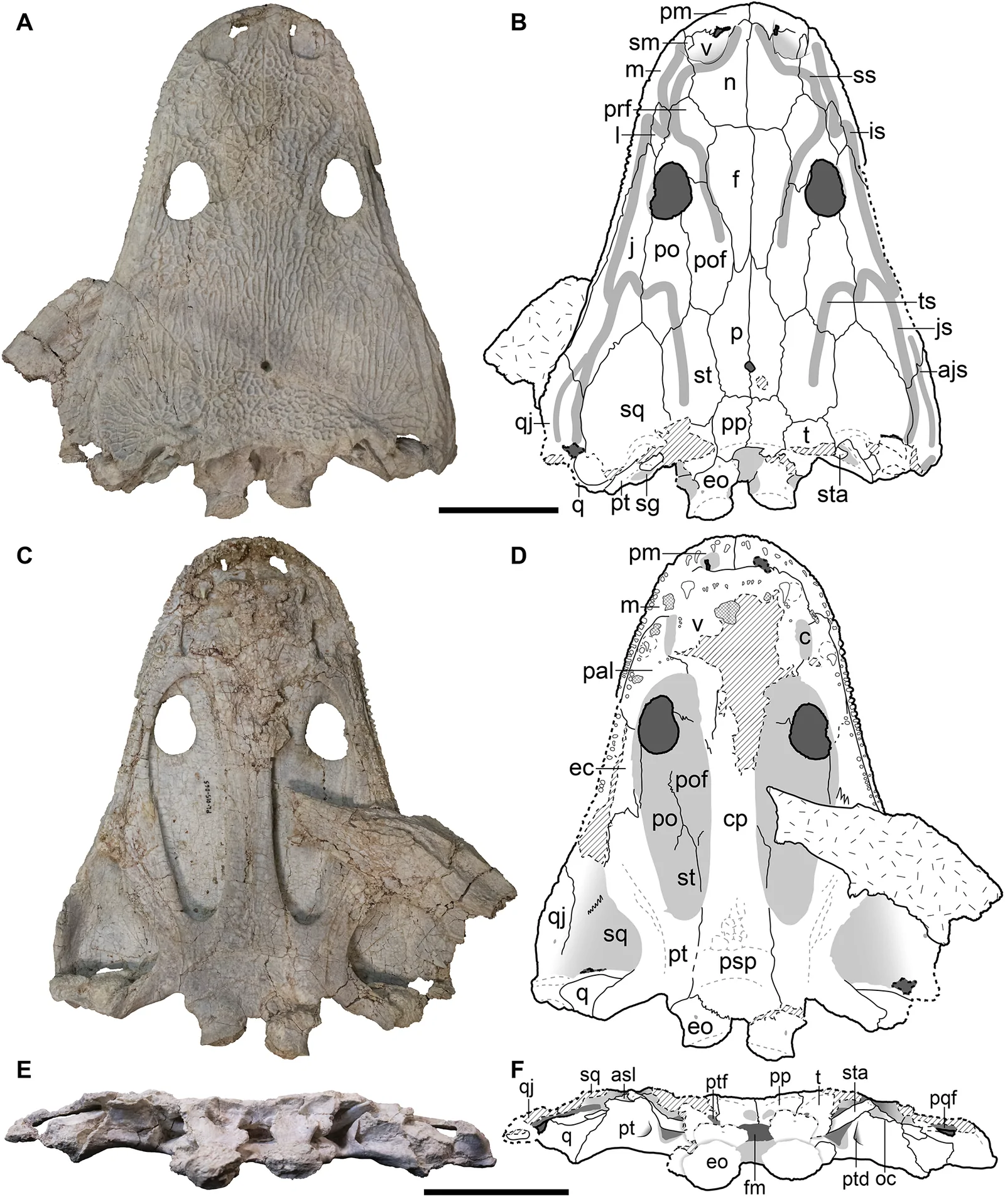
Referred cranium of *Buettnererpeton bakeri*, UWGM 7336, from Nobby Knob in Fremont County, WY. (A) Photograph in dorsal view; (B) interpretive drawing of the same; (C) photograph in ventral view; (D) interpretive drawing of the same; (E) photograph in occipital view; (F) interpretive drawing of the same. Stippling represents residual matrix; fine dashed gray lines represent topographic details like ridges; diagonal lines represent broken surfaces; diamonds represent patches of denticulate palatal plates; hatching represents a mandibular fragment. Abbreviations: ajs, accessory jugal sulcus; asl, ascending lamina of the pterygoid; c, choana (internal naris); cp, cultriform process; ec, ectopterygoid; eo, exoccipital; f, frontal; fm, foramen magnum; is, infraorbital sulcus; j, jugal; js, jugal sulcus; l, lacrimal; m, maxilla; n, nasal; oc, oblique crest of the pterygoid; p, parietal; pal, palatine; pm, premaxilla; po, postorbital; pof, postfrontal; pp, postparietal; pqf, paraquadrate foramen; prf, prefrontal; psp, parasphenoid; pt, pterygoid; ptd, pterygoid depression; ptf, posttemporal fenestra; q, quadrate; qj, quadratojugal; sg, stapedial groove; sm, septomaxilla; sq, squamosal; ss, supraorbital sulcus; st, supratemporal; sta, stapes; t, tabular; ts, temporal sulcus; v, vomer. Scale bar equals 5 cm.

**Figure 12:**
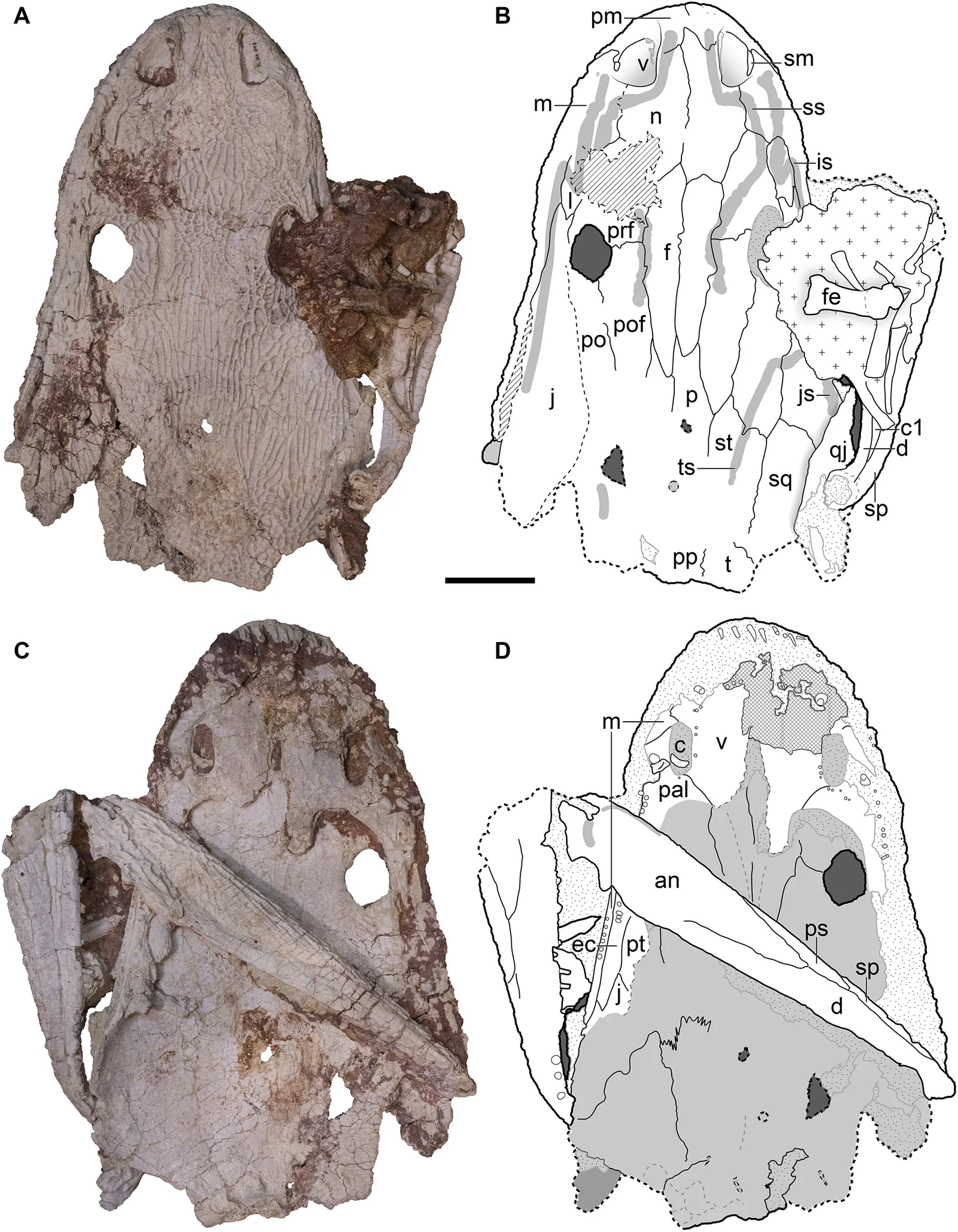
Referred partial cranium of *Buettnererpeton bakeri*, UWGM 7212, from Nobby Knob in Fremont County, WY. (A) Photograph in dorsal view; (B) interpretive drawing of the same; (C) photograph in ventral view; (D) interpretive drawing of the same. Stippling represents residual matrix; fine dashed gray lines represent topographic details like ridges; diagonal lines represent broken surfaces; diamonds represent patches of denticulate palatal plates; crosses represent an accumulation of bones possibly from the sacral region. Abbreviations: an, angular; c, choana (internal naris); c1, coronoid 1 (anterior coronoid/precoronoid); d, dentary; ec, ectopterygoid; f, frontal; fe, femur; is, infraorbital sulcus; j, jugal; js, jugal sulcus; l, lacrimal; m, maxilla; n, nasal; p, parietal; pal, palatine; pm, premaxilla; po, postorbital; pof, postfrontal; pp, postparietal; prf, prefrontal; ps, postsplenial; pt, pterygoid; qj, quadratojugal; sm, septomaxilla; sp, splenial; sq, squamosal; ss, supraorbital sulcus; st, supratemporal; t, tabular; ts, temporal sulcus; v, vomer. Scale bar equals 5 cm.

**Figure 13:**
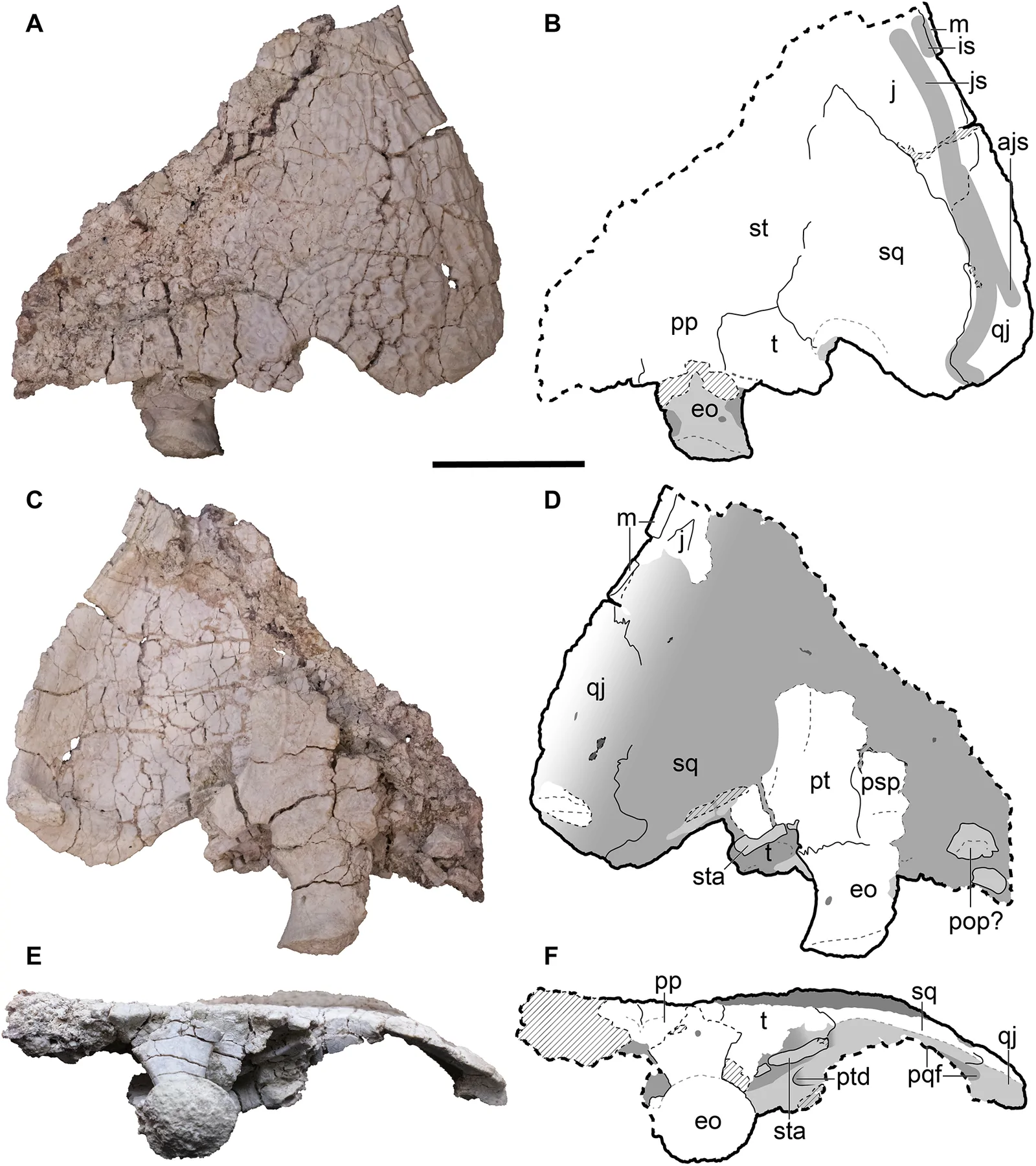
Referred partial cranium of *Buettnererpeton bakeri*, UWGM 7275, from Nobby Knob in Fremont County, WY. (A) Photograph in dorsal view; (B) interpretive drawing of the same; (C) photograph in ventral view; (D) interpretive drawing of the same; (E) photograph in occipital view; (F) interpretive drawing of the same. Stippling represents residual matrix; fine dashed gray lines represent topographic details like ridges; diagonal lines represent broken surfaces. Abbreviations: ajs, accessory jugal sulcus; eo, exoccipital; is, infraorbital sulcus; j, jugal; js, jugal sulcus; m, maxilla; pop?, fragment of left parotic process?; pp, postparietal; pqf, paraquadrate foramen; psp, parasphenoid; pt, pterygoid; ptd, pterygoid depression; qj, quadratojugal; sq, squamosal; st, supratemporal; sta, stapes; t, tabular. Scale bar equals 5 cm.

**Figure 14:**
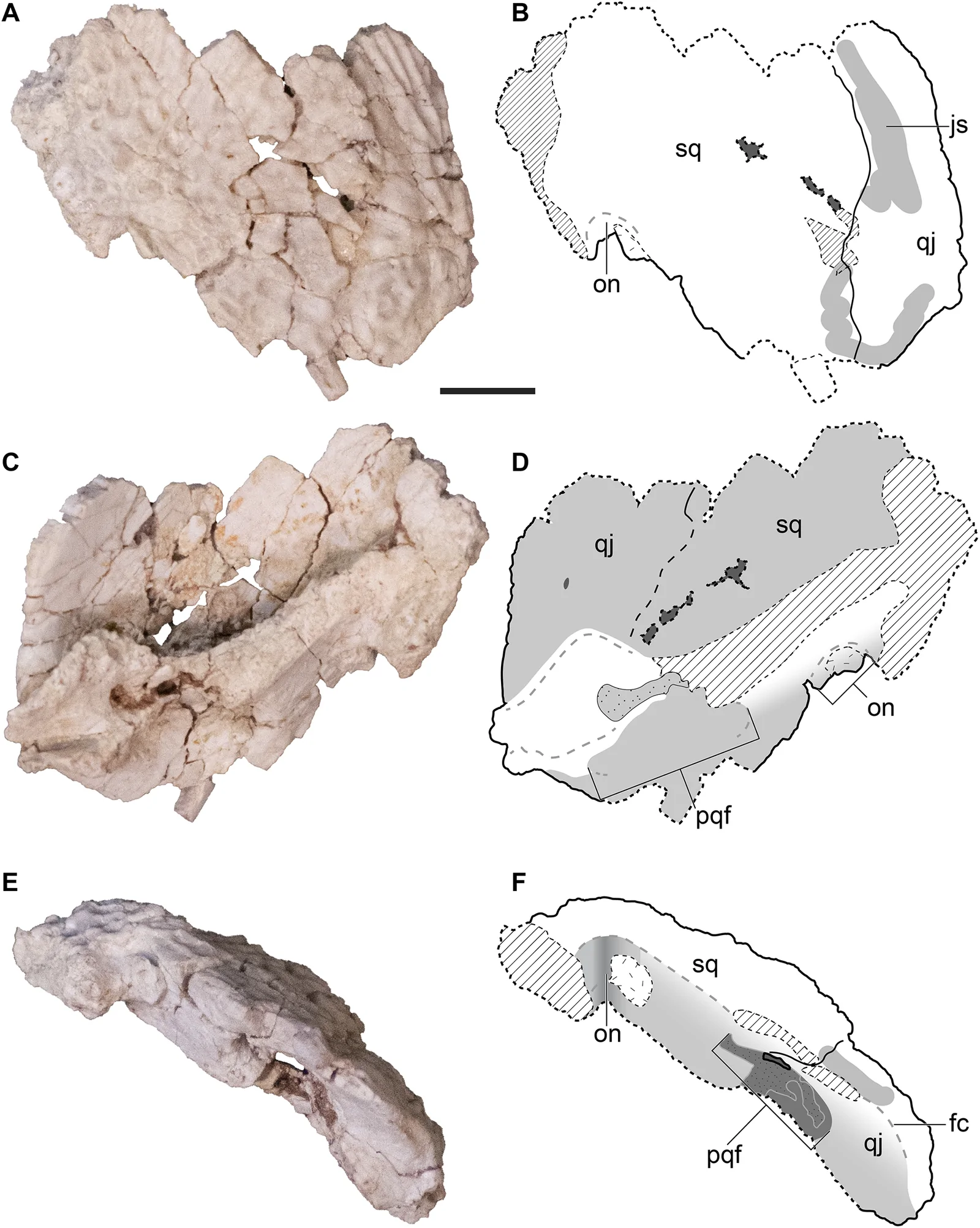
Referred partial cranium of *Buettnererpeton bakeri*, UWGM 8279, from Nobby Knob in Fremont County, WY. (A) Photograph in dorsal view; (B) interpretive drawing of the same; (C) photograph in ventral view; (D) interpretive drawing of the same; (E) photograph in occipital view; (F) interpretive drawing of the same. Stippling represents residual matrix; fine dashed gray lines represent topographic details like ridges; diagonal lines represent broken surfaces; hatching represents an unknown fragment of bone. Abbreviations: fc, falciform crest; js, jugal sulcus; on, otic notch; pqf, paraquadrate foramen; qj, quadratojugal; sq, squamosal. Scale bar equals 1 cm.

**Figure 15:**
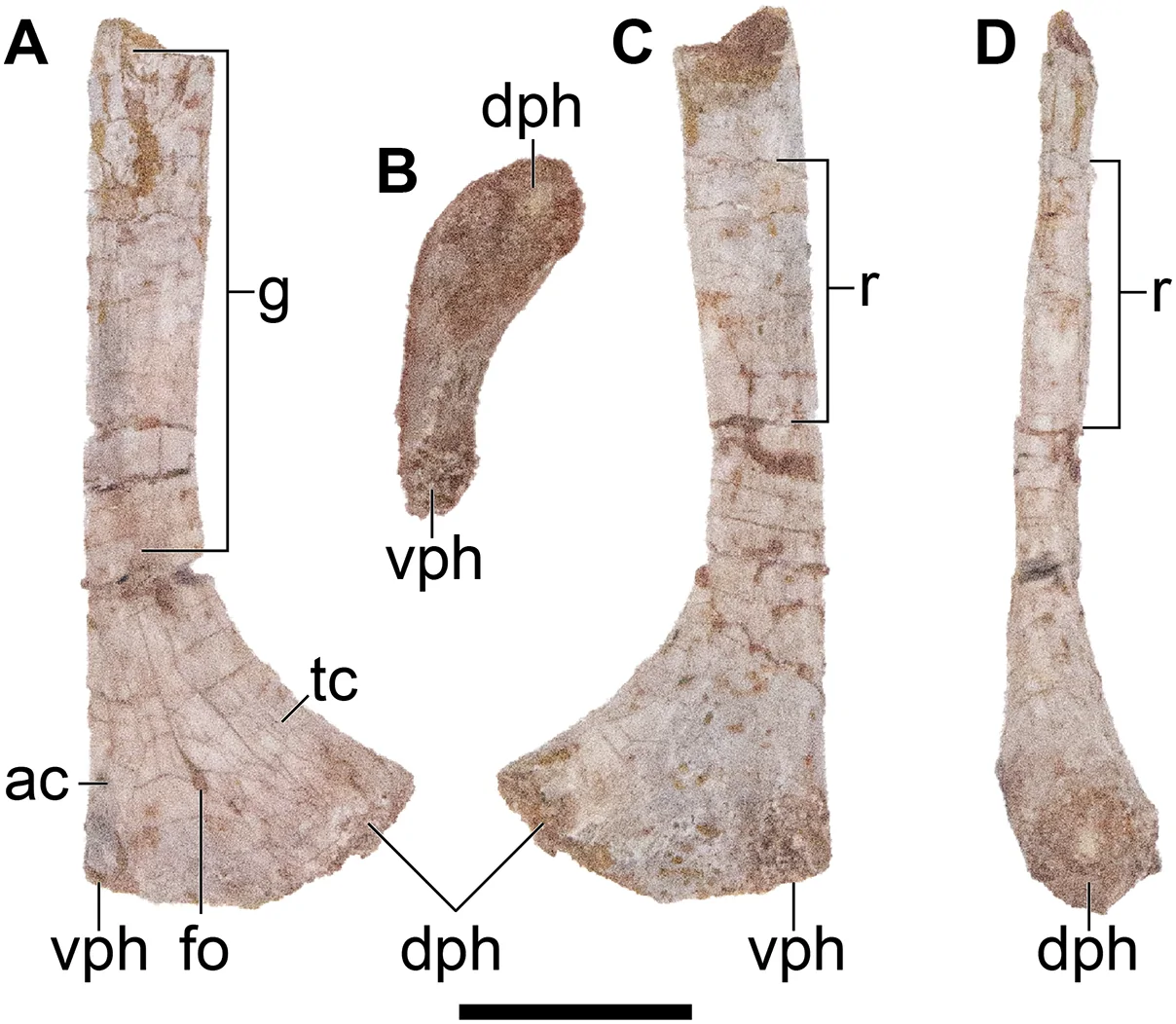
Photographs of a referred left stapes of *Buettnererpeton bakeri*, UWGM 8271, from Nobby Knob in Fremont County, WY. (A) Posteroventral view; (B) proximal view; (C) anteromedial view; (D) posterodorsal view. Abbreviations: ac, anterior crest; dph, dorsal proximal head; fo, foramen; g, groove; r, ridge; tc, transverse crest; vph, ventral proximal head. Scale bar equals 1 cm.

**Figure 16:**
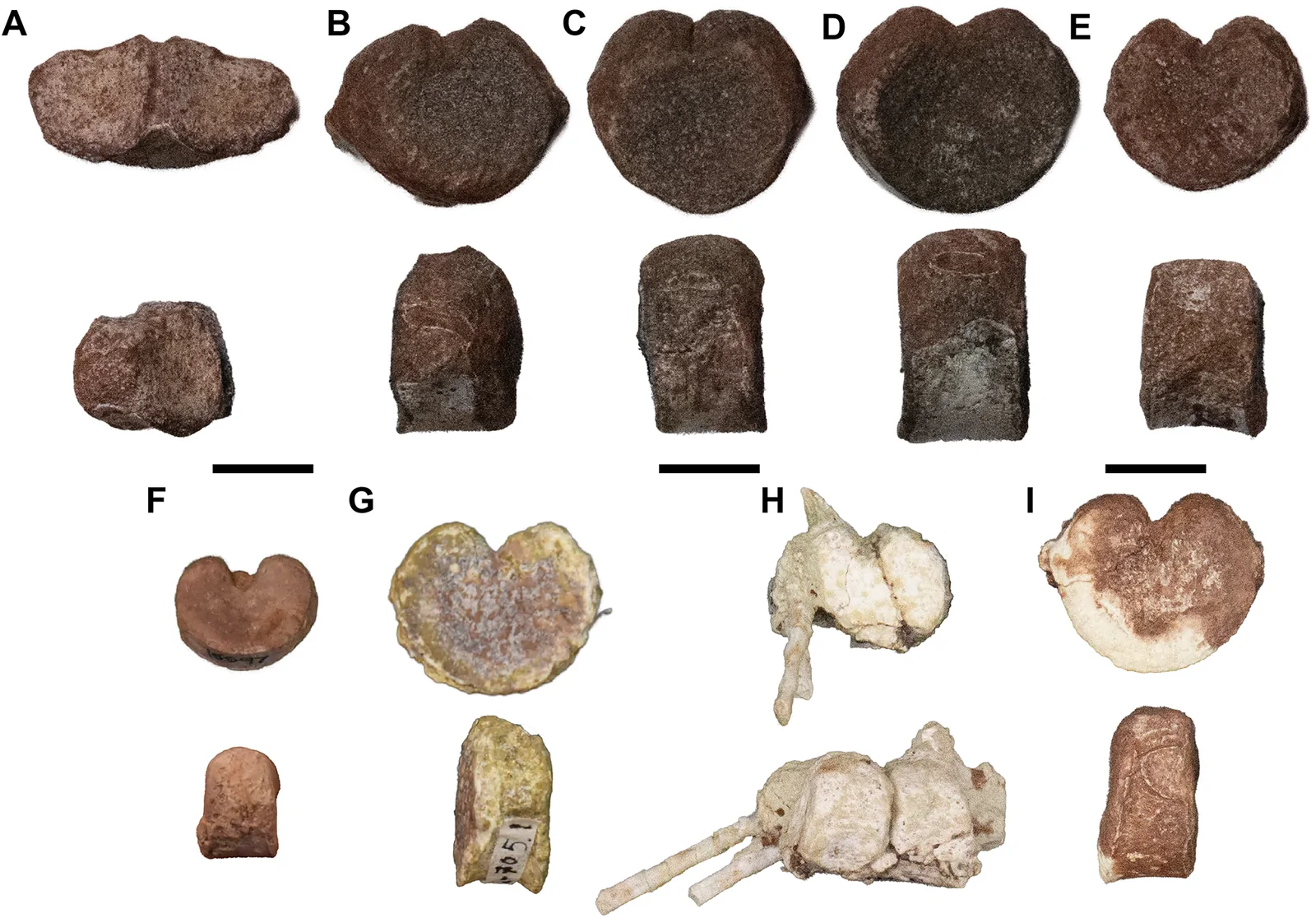
Photographs of small referred intercentra of *Buettnererpeton bakeri*. Upper image is anterior (posterior in H), lower image is either right lateral (A–E, H) or left lateral (F, G, I). (A–E) are all housed under catalog number TMM 31099-123 from Otis Chalk Quarry 2, TX; (F) is from MOTT VPL 3869 (Boren Quarry), TX; (G–I) are from Nobby Knob, WY. (A) Atlas; (B) anterior dorsal; (C) indeterminate trunk; (D) indeterminate trunk; (E) perisacral; (F) indeterminate trunk, possibly postsacral, TTU-P15597; (G) presacral, UWGM 7054; (H) two articulated presacral intercentra with left side ribs, UWGM 7048; (I) anterior dorsal, UWGM 7330. Scale bars equal 1 cm; all elements are at the same scale.

**Figure 17:**
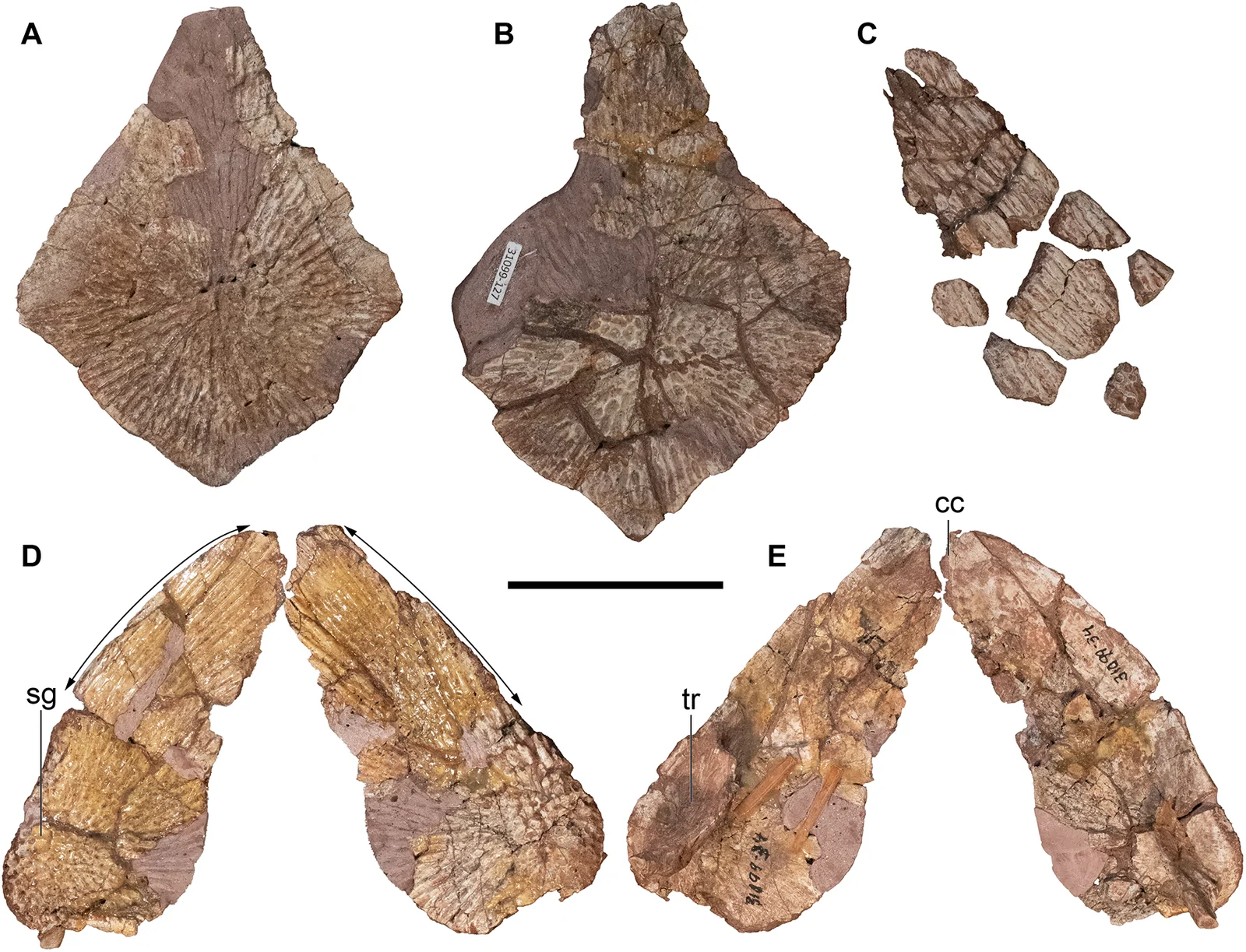
Photographs of pectoral girdle elements referred to *Buettnererpeton bakeri* from Otis Chalk Quarry 2 in Howard County, TX. (A) TMM 31099-126, interclavicle in ventral view; (B) TMM 31099-127 (in part), interclavicle in ventral view; (C) TMM 31099-127 (in part), fragments of left clavicle in ventral view; (D) TMM 31099-34, right and left clavicle in ventral view; (E) the same in dorsal view. Abbreviations: cc, clavicular contact; sg, sensory groove; tr, trough. Anterior is up. Arrows in (D) indicate the curvature of the lateral margins of each clavicle. Scale bar equals 5 cm.

**Figure 18:**
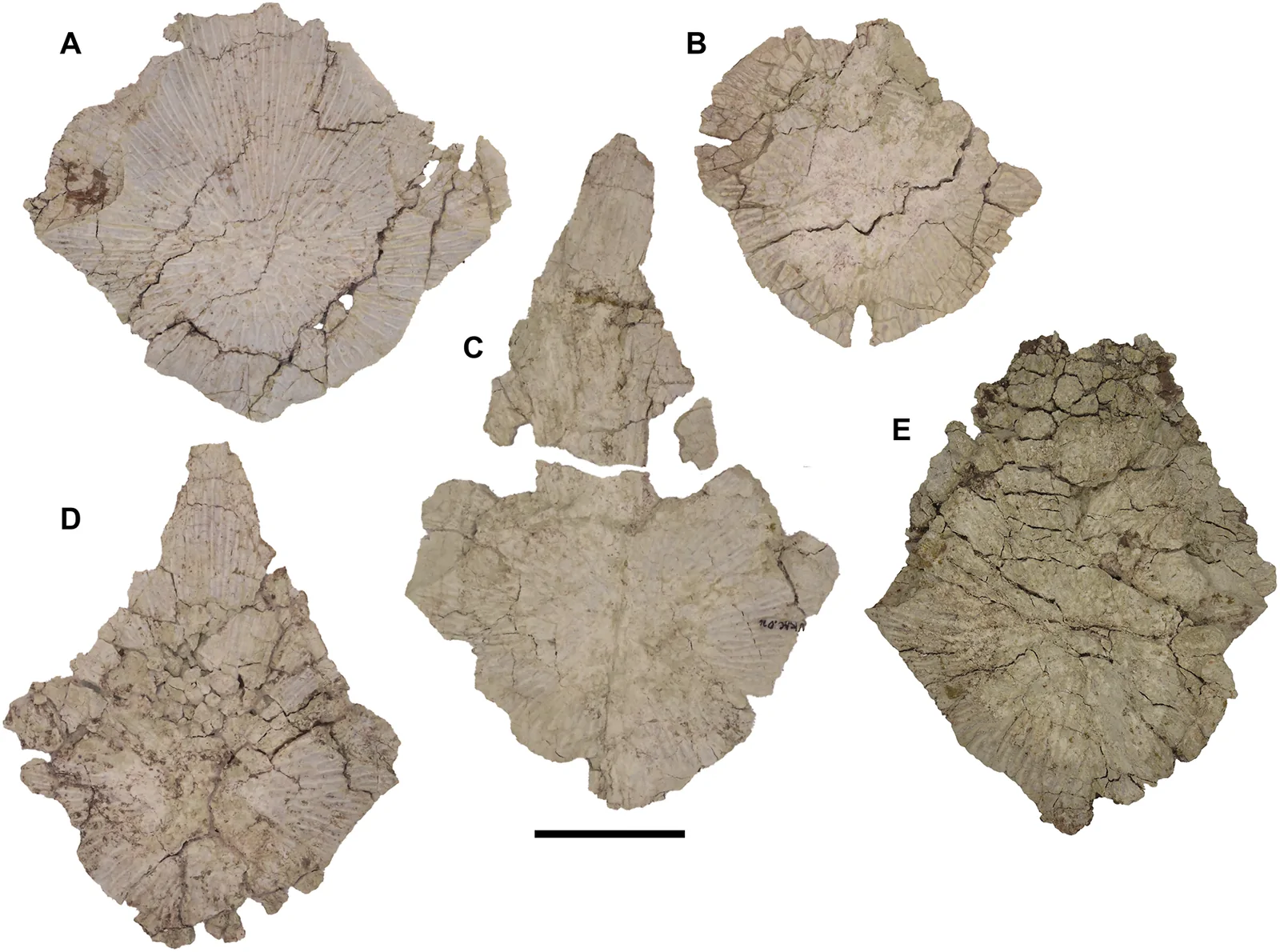
Photographs of select interclavicles referred to *Buettnererpeton bakeri* from Nobby Knob in Fremont County, WY in ventral view. (A) UWGM 8290; (B) UWGM 8272; (C) UWGM 8273; (D) UWGM 8319; (E) UWGM 6782. Anterior is up. Scale bar equals 5 cm.

**Figure 19:**
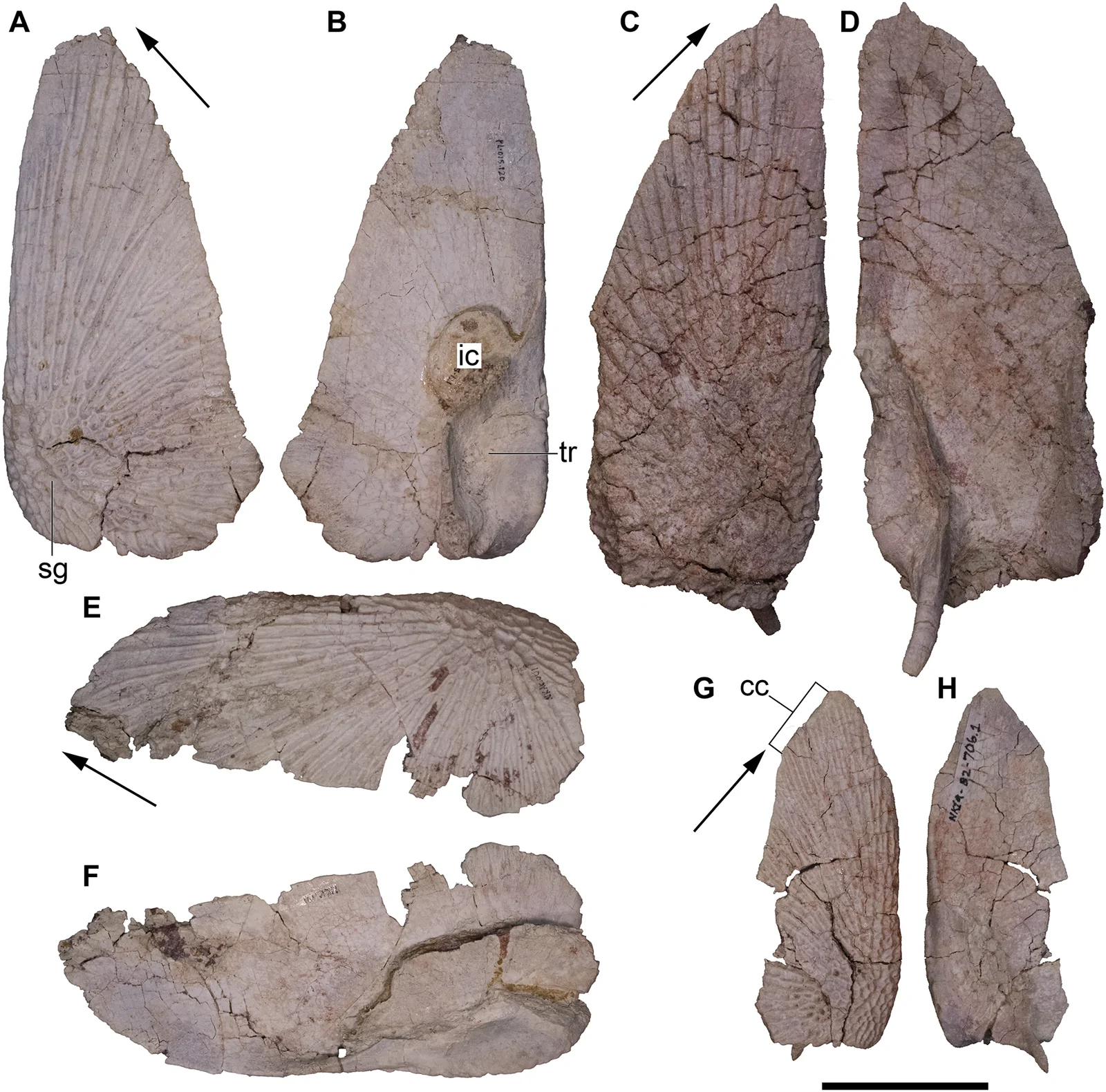
Photographs of select clavicles referred to *Buettnererpeton bakeri* from Nobby Knob in Fremont County, WY. (A) UWGM 6781, right clavicle in ventral view; (B) the same in dorsal view; (C) UWGM 7296 (in part), left clavicle in ventral view; (D) the same in dorsal view; (E) UWGM 8275, left clavicle in ventral view; (F) the same in dorsal view; (G) UWGM 8274, left clavicle in ventral view; (H) the same in dorsal view. Arrows indicate anterior direction on the ventral profiles. Abbreviations: cc, clavicular contact; ic, intercentrum; sg, sensory groove; tr, trough. Scale bar equals 5 cm.

Holotype—. UMMP 13055, an essentially complete skull.

Referred specimens—. All other metoposaurid material from the Elkins Place bonebed (see Gee & Kufner, 2022) including: MCZ 1054, complete cranium; Otis Chalk Quarry 2 metoposaurid material: TMM 31099-12, partial cranium and associated postcrania; TMM 31099-34, pair of clavicles; TMM 31099-123, intercentra; TMM 31099-126, interclavicle; TMM 31099-127, interclavicle and associated fragmentary clavicle; TMM 31099-128, partial cranium and articulated mandible; metoposaurid crania from MOTT VPL 3869: TTU-P10530, complete cranium; TTU-P11046, nearly complete cranium; all metoposaurid material from the Nobby Knob (NK) locality (see Kufner et al., 2025) including: UWGM 7211, skull roof; UWGM 7212, skull roof; UWGM 7275, partial posterior cranium; UWGM 7336, nearly complete cranium; UWGM 8271, left stapes; UWGM 8279, partial posterior cranium.

Type locality and horizon—. The Elkins Place bonebed in the Camp Springs Conglomerate of Snyder County, TX.

Emended diagnosis—. Differentiated from all other metoposaurids except *Apachesaurus gregorii* (in part), *Dutuitosaurus ouazzoui*, and *Arganasaurus lyazidi* by exclusion of the lacrimal from the orbital margin. Differentiated from *A. gregorii* by an elongate lacrimal that occupies 50% or more of the distance between the orbit and the external naris, a gently curving quadrate process of the pterygoid in occipital view, a relatively wide cultriform process, a temporal sulcus that terminates on the supratemporal, a dorsally offset notochordal pit/canal, post-axial trunk intercentra with length shorter than width throughout ontogeny, and a sensory groove along the posterolateral margin of the clavicle. Differentiated from *D. ouazzoui* by shortened intercentra that form dorsally closed cylinders in adults and a large region of reticulate ornamentation on the clavicle. Differentiated from *Ar. lyazidi* by a more developed tabular horn (at similar size), a tabular with an anterolateral corner extending between the squamosal and the supratemporal, a deeper and narrower otic notch (at similar size), and by occipital condyles that are wider and more closely spaced bilaterally.

Remarks—. Gee & Kufner (2022) included a less developed alary process of the premaxilla in their diagnosis of *Buettnererpeton bakeri*. However, the intranarial portion of the premaxilla is only well preserved in one specimen from the type locality of *B. bakeri* in their redescription (UMMP 13820; fig. 9: Gee & Kufner, 2022) and exhibits a slight extension along the medial margin of the external naris. Other specimens referable to *B. bakeri* possess a relatively well-developed alary process of the premaxilla along the medial narial margin, the development of which seems to be decoupled from body size (see below).

**Description**

Elkins Place—. A recent redescription of specimens from the type locality of *Buettnererpeton bakeri* (Gee & Kufner, 2022) provides a detailed comparison of all cranial specimens except MCZ 1054 (formerly UMMP 13821) that was sent to the Museum of Comparative Zoology around the time of Case’s original descriptions (Case, 1931, 1932). MCZ 1054 is slightly smaller than the other complete crania from Elkins Place, and it is nearly identical in most morphological details to previously described specimens. The orbits are slightly more elongate than any other specimens from Elkins Place despite being smaller, possibly indicating an earlier ontogenetic stage. Other possible indicators of an earlier ontogenetic stage for MCZ 1054 are a discontinuous right temporal sulcus also figured by Case (1932; fig. 4) and a discontinuous right accessory jugal sulcus (Figs. 3A, 3B). The temporal and jugal sulci are joined into a single U-shaped sulcus posterior to the orbit often referred to as the postorbital sulcus (*sensu* Stensiö, 1947), and some authors only use the term postorbital sulcus or canal when referring to these even if they are not connected. Two sets of what appear to be a pair of joined pits or a single elongated pit on the right tabular horn are similar to the transverse occipital lateral line sulcus described here for many of the large-bodied metoposaurids from the south and southwest USA, however, this sulcus is not evident on the left side of the skull and is thus considered uncertain in this specimen. A similar condition is also found in another specimen from Elkins Place (fig. 9a: Gee & Kufner, 2022), but the elongate or joined pits were not recognized by the authors as obviously lateral line sulci. Regardless, these features are clearly distinct from the series of 4–5+ joined pits forming the partial transverse occipital sulcus in other North American metoposaurids. MCZ 1054 also has a nasal-lacrimal contact previously only known from UMMP 13822 from the Elkins Place bonebed (Gee & Kufner, 2022). Contrary to a previous interpretation of the Elkins Place specimens, the infraorbital sulcus does not continue posteriorly along the lateral skull roof alongside the jugal sulcus (Gee & Kufner, 2022). Instead, the sulcus deviating from the jugal sulcus along the dorsolateral surface of the quadratojugal is here considered an accessory jugal sulcus arising from the jugal sulcus (Figs. 3A, 3B, 4A, 4B). The accessory jugal sulcus appears to be widely present among North American metoposaurids with the exceptions of *Anaschisma browni sensu stricto* and juvenile specimens of *Apachesaurus gregorii*. The accessory jugal sulcus was noted as a bifurcated postorbital canal on the quadratojugal which joined on the jugal in a large specimen of *Metoposaurus algarvensis* (fig. 3: Brusatte et al., 2015) and may be present in some specimens of *Metoposaurus krasiejowensis* (figs 8, 11: Sulej, 2007).

The palate does not differ significantly from other Elkins Place specimens or other metoposaurids, and the only notable distortion is the slight separation of the posterior maxillae from their contact with the jugal giving the *insula jugalis* a ‘forked’ appearance (Figs. 3C, 3D). In lateral profile, the premaxillae are relatively flat which is similarly the case in other Elkins Place specimens but not previously noted. The dentition of MCZ 1054 is preserved nearly in its entirety, but much of it is incompletely prepared from the matrix to provide structural support for the delicate teeth (Figs. 3C, 3D, 4C, 4D). The dentition of MCZ 1054 does not vary significantly from other metoposaurids with 15 premaxillary teeth, >50 maxillary teeth, and an indeterminate number of teeth in the palatal rows. Pairs of fang (‘tusk’) attachments are present on the vomer and the palatine, but only one fang is present for each pair of fang attachments. The occiput is very well preserved with the only notable damage that the oblique crest of the pterygoid is broken at its origin on both sides of MCZ 1054, and the margins of the foramen magnum are damaged inhibiting identification of a lamellose process of the exoccipital (Figs. 4A, 4B). Unlike other specimens from the Elkins Place bonebed (figs. 11, 14: Gee & Kufner, 2022), the squamosal does not contact the quadrate due to a long medial lamina of the quadratojugal. The dorsal shaft of both stapedes are similarly broken, but they are preserved in articulation as in some other specimens from the Elkins Place bonebed (figs. 11, 18, 23: Gee & Kufner, 2022).

Otis Chalk Quarry 2—. Metoposaurids from the Otis Chalk quarries are commonly considered synonymous with *Anaschisma browni* (or previous synonyms therein, see below), but there are at least two species present: one from Quarry 2 and one from Quarry 3 with additional indeterminate metoposaurid material from quarries 1 and 4.

TMM 31099-12 (formerly TMM 31099-12A of Sawin, 1944) is a crushed, fragmentary skull with associated clavicles and an interclavicle (Fig. 5). Several additional postcranial elements are included under TMM 31099-12; these were used for the basis of character-state scores where relevant, but a complete osteological description of the Otis Chalk Quarry 2 metoposaurid specimens is underway. The skull is missing portions of the rostrum including the premaxillae in their entirety and much of the maxilla and the anterior nasal and vomer. The sutures surrounding the lacrimal are well preserved and demonstrate a complete lack of a contribution to the orbital margin (Figs. 5A, 5B). While the posterior skull roof is relatively more distorted, the parietal-supratemporal suture exhibits a wide angle like that of *Metoposaurus krasiejowensis* (Sulej, 2002, 2007). The supraorbital sulcus passes medial to the lacrimal, although it is much closer to the lacrimal than that of *Apachesaurus gregorii*. There is a Z-shaped flexure of the infraorbital sulcus on the right lacrimal. The cultriform process is relatively wide, likely subequal in width to the minimal width of the palatal ramus of the pterygoid although the latter is fragmented (Figs. 5C, 5D). A short, though nearly 50% of the width of the palatal ramus, *insula jugalis* could be identified on the left side of TMM 31099-12. The postcrania of TMM 31099-12 are consistent with previous descriptions of *B. bakeri* including features such as a sensory sulcus visible along the posteroventral margin of the right clavicle, wide regions of reticulate ornamentation on both the clavicle and the interclavicle, and cylindrical trunk intercentra in adults (Fig. 5). Additional postcranial elements that are fully prepared from Otis Chalk Quarry 2 are described below.

TMM 31099-128 (formerly TMM 31099-12b (Sawin, 1944); also listed as TMM 31099-34 (Mueller, 2016; Stocker, 2013) and TMM 31099-12B (Davidow-Henry, 1989; Gee & Parker, 2020; Hunt, 1993)) includes a skull, semi-articulated right hemimandible, and an associated left hemimandible (Figs. 6, 7). Previous inclusion of this specimen under TMM 31099-34 (an associated pair of clavicles) could not be confirmed due to the presence of at least three individuals of similar size from Otis Chalk Quarry 2 determined by the presence of three small interclavicles (TMM 31099-12, TMM 31099-126, and TMM 31099-127). Taxonomic referrals of TMM 31099-128 have been inconsistent with some previous authors considering it a juvenile of *Anaschisma browni* or previous subjective synonyms (Davidow-Henry, 1989; Gee, Parker & Marsh, 2020; Gee & Kufner, 2022; Gee & Parker, 2020; Hunt, 1993) and still others as a juvenile of *Buettnererpeton bakeri* or synonyms therein (this study; Mueller, 2016; Sawin, 1944; Stocker, 2013). The referral of this specimen to *B. bakeri* is confirmed here by: a lacrimal that does not contribute to the orbital margin, a sub-triangular squamosal, a wide cultriform process, a quadrate ramus of the pterygoid with a gradual slope as opposed to a right-angle inflection (Figs. 6, 7), and associated intercentra that are anteroposteriorly shorter than their width and with a dorsal incisure for the notochordal canal (see below).

TMM 31099-128 is remarkably similar in cranial proportions with larger specimens of *B. bakeri*. There is minimal taphonomic distortion with a notable exception being slight crushing of the right side of the skull presumably due to conformation around the semi-articulated right hemimandible. The right hemimandible is slightly posteriorly displaced such that the glenoid fossa is mostly visible in dorsal view (Figs. 6A, 6B). The arrangement of the skull roof and palate are nearly identical with that of other specimens of *B. bakeri*. A notable exception is the supraorbital sulcus that passes just medial to the lacrimal, shared with TMM 31099-12 and specimens from the Boren Quarry (TTU-P10530 and TTU-P11046) as well as several specimens referred to other taxa. The premaxillae are distinctly downturned in lateral view (Figs. 7C, 7D) unlike specimens of *B. bakeri* from the type locality (Fig. 3; Gee & Kufner, 2022) and the Popo Agie Formation (see below). The septomaxillae are missing, presumably due to pre- or syn-depositional loss because there are faint striae along the lateral side of the external naris where the septomaxilla would have contacted the maxilla and the premaxilla (Figs. 6A, 6B). It is not clear if the epipterygoids, the sphenethmoid, or any otic bones are present/ossified because matrix is covering the areas these elements would occupy. No details of the marginal or palatal dentition can be determined because it is relatively unprepared due to the delicate nature of the teeth and relative hardness of the matrix (Figs. 6C, 6D). A small lamellose process is present on the right exoccipital barely projecting into the foramen magnum (Figs. 7A, 7B), like that of the holotype of *A. gregorii* (see below). The posttemporal fenestrae are not visible, but it is not clear if this is due entirely to distortion (*e.g*., the vertical process of the right exoccipital overlaps with the descending processes of the postparietal and the tabular), true absence, or diminutive size. The width of the paraquadrate foramen is slightly smaller than the widths of both the occipital condyle and the foramen magnum.

Boren Quarry—. Two relatively complete metoposaurid crania were excavated from the Boren Quarry in strata overlying the Boren Ranch sandstone in Garza County, TX (Martz, 2008; Mueller, 2016). These specimens and numerous metoposaurid postcrania were the subject of an ongoing study by W. D. Mueller prior to his passing in 2019. TTU-P10530 (Fig. 8) and TTU-P11046 (Fig. 9) were previously included in a conference abstract (Houle & Mueller, 2004), Mueller’s dissertation (Mueller, 2016), and Martz’s dissertation (Martz, 2008) demonstrating referral to *Buettnererpeton bakeri*. The skull roof and palate of TTU-P10530 are very well preserved and prepared, but the occipital surface has some damage and residual matrix hindering morphological interpretations (Figs. 10A, 10B).

The anatomy of TTU-P10530 is broadly consistent with specimens from the type locality (Elkins Place) of *B. bakeri* apart from its larger size, and only differences are noted here. The premaxillae appear elongate in dorsal view due to taphonomic deformation confirmed by breakage along the premaxilla-vomer contact in palatal view and additional splitting along the midline suture between the premaxillae (Fig. 8D). If this deformation was corrected, the premaxillae would most likely exhibit the slight ventral downturn present in some less distorted skulls (*e.g*., Figs. 7C, 7D). There is a bulge formed by the postparietals along the posterior skull roof margin (Figs. 8A, 8B) unlike most metoposaurids save for a minority of specimens (*e.g*., UMMP 13820, fig. 9: Gee & Kufner, 2022; see below). The lateral line sulci are expressed very clearly across the skull roof and are typical for *Buettnererpeton bakeri* (Figs. 8A, 8B). The occiput is less dorsoventrally crushed than in most examples from Elkins Place (Fig. 10), and the oblique crest of the pterygoid is essentially complete and visible in dorsal view (Fig. 8). The posttemporal fenestrae are visible, small and rounded, and slightly recessed in a large but shallow triangular depression (Figs. 10A, 10B). The paraquadrate foramen is wide, much wider than both the foramen magnum and the occipital condyle (Figs. 10A, 10B).

The larger skull from the Boren Quarry, TTU-P11046, is less complete than TTU-P10530, but it is the largest known articulated skull of *Buettnererpeton bakeri* with a midline length of at least 39 cm. TTU-P11046 is missing the premaxillae, the right quadrate, and the anterior portion of the cultriform process (Fig. 9). On the right side of the skull roof, the lateral line sulci are very well defined and demonstrate the presence of an accessory jugal sulcus on the quadratojugal. On the left side of the skull roof, the supraorbital sulcus passes medially to the lacrimal, but on the right side, it passes onto the lacrimal (Figs. 9A, 9B). The cultriform process in both specimens from Boren Quarry is proportionally narrow compared to that of other referred specimens of *B. bakeri*, narrower than the minimal width of the palatal ramus of the pterygoid (Figs. 8C, 8D, 9C, 9D). The right maxilla appears to be complete posteriorly (Fig. 9D), but it is separated from the quadratojugal by the jugal unlike other specimens of *B. bakeri* (*e.g*., UMMP 13823, fig. 16: Gee & Kufner, 2022) for which this can be evaluated. The occiput is less well preserved than that of TTU-P10530, but in the morphology that is evident, these specimens are indistinguishable (Fig. 10C). No stapes was identified from MOTT VPL 3869.

NK—. The NK locality comprises a monodominant bonebed with >20 minimum number of individuals of *Buettnererpeton bakeri* from the Carnian-aged purple unit of the Popo Agie Formation in Fremont County, WY (Kufner et al., 2025). Preparation of material from NK is ongoing, but nearly every element of a metoposaurid skeleton is represented including several complete crania and some articulated elements (partial anterior skeletons and limbs). Most of the specimens are crushed, but otherwise surface details are well preserved. Small, fragmentary metoposaurid material has been found at the site alongside typical large-bodied specimens comparable in size to those from localities like Elkins Place though smaller than those from the Boren Quarry. This small metoposaurid material is considered to represent one or more juveniles of *B. bakeri* due to similarities in postcranial morphology with referred specimens of *B. bakeri* (see below), and a relatively wide range in ontogenetic stages informed by preliminary osteohistology of ribs from NK (see Fig. S1 and accompanying description in File S1). A more thorough description is provided for these previously unpublished specimens.

UWGM 7336 was the most complete cranial specimen from NK available at the time of study (Fig. 11). UWGM 7336 is nearly complete except for loss of the tabular horns and damage to the surrounding areas, and it is significantly flattened which is most obvious in the postorbital cranium. A fracture between the otic notches of UWGM 7336 extends anteriorly to nearly the pineal foramen, slightly distorting the appearance of the posterior parietals, and portions of the basicranium have been posteriorly and dorsally displaced relative to the skull roof. UWGM 7336 bears a prominent alary process of the premaxilla that extends along the medial narial margin (Figs. 11A, 11B). The septomaxilla is preserved in the lateral external naris covering the juncture of the premaxilla, the maxilla, and the vomer. It is poorly integrated with the surrounding elements and readily dislodged by pre- or post-burial events (see left septomaxilla that was partially lost). The anterior process of the prefrontal is pointed and the angle between the medial and lateral margins is relatively narrow compared with most other North American metoposaurids not assigned to *B. bakeri*. The lacrimal is excluded from the orbital margin, a condition found in all other metoposaurid crania from NK for which this feature can be evaluated. Like most other examples of *B. bakeri*, the lacrimal is relatively short and does not contact the nasal anteriorly. Unlike most specimens of *B. bakeri* in which the posterior margin of the lacrimal is level or posterior to the orbital margin, the lacrimal of UWGM 7336 is situated entirely anterior to the orbital margin. UWGM 7336 exhibits a unique condition of the parietal-supratemporal suture where the right suture is subparallel to the midline, but the left suture is oblique to the midline (Figs. 11A, 11B). In all remaining NK specimens preserving this region, the parietal-supratemporal suture is subparallel to the midline. Like other specimens of *B. bakeri*, the squamosal has a smooth, curving lateral margin resulting in a subtriangular profile. All cranial lateral line sulci are present in UWGM 7336 except for the transverse occipital sulcus, but the tabular is incomplete. Due to posterior displacement of the basicranium relative to the skull roof, the stylus columellae of both stapedes in the stapedial groove are visible in dorsal view.

In ventral view, a significant portion of the anterior palate of UWGM 7336 is damaged or obscured by matrix (Figs. 11C, 11D). The premaxilla-maxilla suture could not be identified, and the maxilla on the left side does not reach the choana and is instead restricted by an anterior contact of the palatine and the vomer. There is a small foramen on the internal surface of the quadratojugal. The cultriform process of the parasphenoid is subequal in width with the minimal width of the palatal ramus of the pterygoid. The ectopterygoid has a posteromedial extension on the right side, but on the left side it appears to be absent, but a fragment of a mandible obscures much of the left palatal ramus. The exoccipital and the basal plate of the parasphenoid form a more transversely oriented suture resulting in a basal plate that appears rounded rather than tapering or ‘pointed’ along its posterior margin, a condition found in some other specimens of *B. bakeri* (Figs. 3, 6; figs. 10, 13, 22: Gee & Kufner, 2022). The muscular crest on the basal plate is formed by a single transverse crest rather than a pair of L-shaped crests (Figs. 11C, 11D). Reticulate ornamentation on the basal plate of the parasphenoid radiates toward but does not extend onto the cultriform process, and between the region of reticulate ornament and the muscular crest on the basal plate, there is a smooth, slightly depressed surface.

While all of the tooth rows of the typical metoposaurid palate can be identified in UWGM 7336, they are all too incomplete or unprepared to provide tooth counts for any elements. This is similarly the case for all NK specimens at present. Of the teeth that are available for study, the left vomerine fang of UWGM 7336 is well preserved revealing a short keel at the apex of the tooth extending about a third of the way to the fang base. The palatal teeth of UWGM 7336 do not appear to have significantly greater diameters than the adjacent marginal teeth. Isolated patches of denticulate palatal plates are present on the vomer and the palatine.

The degree of crushing to the posterior cranium is most evident in occipital view (Figs. 11E, 11F). The posteriorly and dorsally displaced exoccipitals partially obscure the view of the small, oval posttemporal fenestrae. The medial margin of the vertical process of the exoccipital is slightly angled medially, here interpreted as a low lamellose process, but this process is not distinctly offset from the occipital pillar as in most of the large-bodied specimens described here. A small foramen is present just below the contact of the vertical process of the exoccipital with the descending processes of the postparietal and the tabular. The oblique crest of the pterygoid is tall, and the dorsal margin of the left oblique crest is folded posteriorly likely due to compaction. The pterygoid depression is prominent formed by an L-shaped ridge. The squamosal and the quadratojugal fully envelop the paraquadrate foramen *via* the ornamented roofing elements and ventral processes, but the precise dimensions of the paraquadrate foramen are unclear due to damage. The ventral process of the squamosal forms a broad contact with the quadrate and excludes the quadrate from participating in the margin of the paraquadrate foramen. Both stapedes of UWGM 7336 are in approximate life position, and a more complete description of an isolated stapes from NK is provided below.

UWGM 7212 is a partial skull roof with portions of the anterior palate, and several additional mandibular and postcranial elements are attached to the cranium (Fig. 12). Damage to UWGM 7212 is predominantly in the postorbital skull with the entire basicranium missing, much of the posterior skull margin missing, the left ‘cheek’ broken and slightly displaced medially, and the right ‘cheek’ broken and folded over such that the ventral surface of the quadratojugal is visible in dorsal view (Figs. 12A, 12B). The preorbital cranium is proportionally long with the orbits placed well posterior to the anterior margin of the interpterygoid vacuity (Fig. 12). UWGM 7212 has a prominent alary process of the premaxilla that is separated from the narial margin similar to the condition in a previously published NK specimen, UWGM 7211 (fig. 5: Kufner et al., 2025). A septomaxilla is also present in lateral external naris of UWGM 7212. The lacrimal of UWGM 7212 is more elongate than that of UWGM 7336, occupying about 50% of the orbit-narial distance, but otherwise very similar in its contacts and overall shape (Figs. 12A, 12B). The lacrimal of the previously published specimen UWGM 7211 is elongate and forms a broad contact with the nasal (fig. 5: Kufner et al., 2025), leading to similar variation as that of the Elkins Place bonebed (Fig. 3; Gee & Kufner, 2022). The frontal appears relatively narrow, but it is of equal or lesser width than the parietal at its greatest width just anterior to the orbits (Figs. 12A, 12B). The cranial lateral line sulci do not differ significantly from those of UWGM 7336 (Figs. 11A, 11B), and the transverse occipital sulcus could not be identified, although the tabular horn is missing in the preserved part of the tabular of UWGM 7212 (Figs. 12A, 12B). Minimal morphological information is revealed by the ventral view of UWGM 7212 (Figs. 12C, 12D). Unlike UWGM 7336, the maxilla forms a restricted contribution to the lateral margin of the choana as is typical for metoposaurids. The ectopterygoid does not form a posteromedial projection extending onto the palatal ramus, and the *insula jugalis* is present and short. Elongate premaxillary teeth are well preserved but incompletely revealed due to under preparation (intended to preserve the delicate dentition). The anterior part of the left vomer is almost entirely obscured by a region of matrix interspersed with disarticulated denticulate palatal plates.

UWGM 7275 is a partial posterior cranium encompassing the right ‘cheek’ and part of the right basicranium (Fig. 13). The pattern of skull roof bones is indistinguishable from previously described NK specimens, but the tabular horn is preserved. The end of the tabular horn is ‘pronged’ with a shallow emargination between short anterior and posterior projections. This is similarly the case with UWGM 7211 (figs. 5a–b: Kufner et al., 2025), but the margin of the tabular horn is rounded in some incompletely prepared specimens from NK (not figured). A short segment of the infraorbital sulcus, the posterior extent of the jugal sulcus, and the full extent of the accessory jugal sulcus are present in UWGM 7275. The transverse occipital sulcus is absent along the posterior skull margin. The ventral view of UWGM 7275 confirms the presence of a short *insula jugalis* (Figs. 13C, 13D). The posterior extent of the maxilla is fragmentary but preserved, and it reaches the quadratojugal in a point contact. The quadrate is entirely missing, but the ventral process that would partially envelop the quadrate is present. The descending lamina of the squamosal is present, in part, and a finished margin interpreted as the medial margin of the paraquadrate foramen suggests a relatively wide paraquadrate foramen as in most large-bodied metoposaurids. In ventral view, the quadratojugal-squamosal suture is more sinuous than that expressed dorsally. A small foramen also identified in UWGM 7336 (Figs. 11C, 11D) is present on the internal surface of the quadratojugal (Figs. 13C, 13D).

In occipital view, the dorsoventral flattening of UWGM 7275 is apparent (Figs. 13E, 13F). Like many other metoposaurid specimens, the exoccipital is dorsally and posteriorly displaced relative to the rest of the cranium obscuring the position of the posttemporal fenestra and artificially lengthening the exposure of the occiput. Indeed, the broken surfaces of the vertical process of the exoccipital are visible in dorsal view. Three foramina could be identified on the exoccipital of UWGM 7275, one on the dorsolateral aspect just anterior to the occipital condyle, one on the ventrolateral aspect at about the midlength of the body of the exoccipital, and one on the posterior aspect of the vertical process just ventral to the contact with the postparietal and the tabular. The identities of these foramina are unclear, and variation in the positions and number of foramina in the exoccipital of *B. bakeri* has been previously documented (Gee & Kufner, 2022).

An isolated right ‘cheek’ from one of the smallest metoposaurids from NK, UWGM 8271, is made up of the squamosal and the quadratojugal (Fig. 14). A clear and relatively narrow and deep otic notch is present on the squamosal with an unornamented “gutter” surrounding it. A small fragment of bone is present in the otic notch, but it is not clear if this is a fragment of the stapes or another element. The ornament on the squamosal of UWGM 8271 is distinct in being almost entirely reticulate and lacking any elongate ridges and grooves typically found along the lateral margin of the squamosal (*e.g*., Figs. 3, 8, 9, 11). The jugal sulcus is preserved and continuous, but an accessory jugal sulcus could not be distinguished from elongate grooves on the surface of the quadratojugal. A slight evulsion of the jugal sulcus could represent a portion of the accessory jugal sulcus. The jugal sulcus approximates the course of the squamosal-quadratojugal suture until it reaches the posterior skull margin where is curves laterally along the margin. A small foramen of unknown affinity can be seen on the internal surface of the quadratojugal (Figs. 14C, 14D) as in some other specimens from NK (Figs. 11C, 11D, 13C, 13D). The dorsal margin of the paraquadrate foramen is preserved, and it is comparable in proportional width to TMM 31099-128 (Figs. 7A, 7B), being about the same width as the quadratojugal in occipital view (Figs. 14E, 14F).

An isolated stapes from NK provides another point of comparison with specimens of *Buettnererpeton bakeri* from the type locality and other North American metoposaurids (Fig. 15). The dorsal and ventral proximal heads of the stapes are unfinished, and a sharp convex ridge is present between the proximal heads of UWGM 8271. Compared to other specimens of *B. bakeri* (figs. 18b, 31: Gee & Kufner, 2022) and other stereospondyls (*e.g*., Schoch, 2000), the underdeveloped proximal heads may indicate an earlier ontogenetic stage. The anterior crest and transverse crest are weakly developed in UWGM 8271 unlike stapedes from the type locality of *Buettnererpeton* which have pronounced crests just distal to the proximal expansion for the two heads (figs. 18b, 31: Gee & Kufner, 2022). A small probable nutrient foramen is present on the posteroventral surface between the anterior and transverse crests, but no perforating stapedial foramen is present. The stylus columella is elongate and forms an elongate oval in cross-section. A groove extends along the entire posterior face of the stylus columella (Fig. 15A) which could be due to crushing as described in *Mastodonsaurus giganteus* (Schoch, 2000). A short but sharp ridge is present on the anteromedial surface of the distal half of the stylus columella (Figs. 15C, 15D).

Intercentra—. Here “trunk” is used to refer to vertebrae anterior to the sacrum and posterior to the third or fourth vertebra (first or second posterior to the axis). The term “presacral” has been used to refer to the five vertebrae preceding the sacrum in *Dutuitosaurus* (Dutuit, 1976b; Sulej, 2007) and as such this term has been applied to other metoposaurids (*e.g*., Gee & Kufner, 2022; Konietzko-Meier, Bodzioch & Sander, 2012). Isolated presacral intercentra can be readily identified by the rounded, intersegmental parapophyses located at about the mid-height of the intercentrum and by an acoelous to weakly amphicoelous body shape. Trunk intercentra anterior to the presacral intercentra typically possess a single parapophysis on the posterior aspect of the lateral surface that is positioned either at the mid-height (mid-dorsal) or higher (postcervical and anterior dorsal). The anterior trunk intercentra are typically opisthocoelous and gradually become acoelous posteriorly along the axial column.

Most intercentra from the type locality of *Buettnererpeton bakeri* (Elkins Place) are typical of large-bodied metoposaurids forming dorsally closed discs with a notochordal pit positioned dorsal to the center of the vertebral body (Gee & Kufner, 2022). One small trunk intercentrum from Elkins Place (fig. 42g: Gee & Kufner, 2022) exhibits unique features among metoposaurids including: (1) length shorter than both height and width (proportions more similar to large-bodied metoposaurids than to stratigraphically higher occurrences of small-bodied metoposaurids), (2) a dorsally open notochordal canal which forms an incised, subcircular profile in transverse section, (3) lack of a notochordal pit due to this open canal. Small dorsally open intercentra are also known from three other localities with *B. bakeri* (Otis Chalk Quarry 2: Figs. 16A–16E; Boren Quarry: Fig. 16F; NK: Figs. 16G–16I). Two of these localities also have large, dorsally closed trunk intercentra with a notochordal pit above the center of the vertebral body (not figured: Boren Quarry; NK). Some intercentra from Otis Chalk Quarry 2 exhibit partial closure of the notochordal canal (*e.g*., Fig. 16C). These characteristics are likely the result of osteological immaturity in the axial skeleton further confirmed by an atlas intercentrum from Otis Chalk Quarry 2 that lacks a fused neural arch (Fig. 16A) like a juvenile specimen (UCMP 171591) referred to *Apachesaurus gregorii* (fig. 5a: Rinehart & Lucas, 2018). The Otis Chalk Quarry 2 intercentra differ in proportions from the remaining small *B. bakeri* intercentra being slightly longer compared to their height and width than the similarly sized but foreshortened examples from Elkins Place and NK (Fig. 16; fig. 42g: Gee & Kufner, 2022).

Interclavicles and clavicles—. Several dermal elements of the pectoral girdle from Otis Chalk Quarry 2 and NK were previously unfigured or described that are associated with TMM 31099-128. These elements are briefly described here due to their frequent inclusion in metoposaurid species diagnoses (Gee & Kufner, 2022; Lucas et al., 2016). Two interclavicles (TMM 31099-126 and TMM 31099-127 (in part)) and three clavicles (TMM 31099-127 (in part) and TMM 31099-34) were identified from Otis Chalk Quarry 2 and are likely from a similar sized individual as TMM 31099-128. The interclavicles form the typical rhomboidal shape but with a slightly offset posterior process previously proposed to indicate juvenile morphology in *Apachesaurus gregorii* (Rinehart & Lucas, 2018), but similar interclavicles are present in the Elkins Place (EP) bonebed from much larger individuals with significant variation in the shape of the posterior margin from similarly sized individuals (figs. 55–56: Gee & Kufner, 2022).

The clavicle of TMM 31099-127 is highly fragmented and of limited utility for a description of the morphology (Fig. 17C), but the clavicle pair of TMM 31099-34 is well preserved (Figs. 17D, 17E). The right clavicle is broken at its midlength, but a slight curvature is present along the lateral margin of the unbroken segment. The lateral margin of the left clavicle is nearly straight. The anteromedial margins of the right and left clavicle meet at prolonged contact indicated by the parasagittally oriented margin diverging from the angled medial margin of most of the ventral blade of the clavicle (“cc” in Fig. 17E). As in all other North American metoposaurids, the posterolateral aspect of the ventral surface bears a wide region of reticulate ornamentation (Fig. 17D). This region of reticulate ornamentation is interrupted by a faint sensory groove along the posterolateral margin of the clavicle. Notably, this groove is not well developed with some reduced ridges from the reticulate ornament still traversing the bottom of the sulcus. The dorsal surface of the clavicle has the typical ‘trough’ lateral to the dorsal lamina and some minor remnants of the ascending process extending posteriorly and dorsally from this lamina (Fig. 17E).

Pectoral girdle elements from the NK quarry are relatively abundant, and a select few are figured here to demonstrate the rationale for character-state scores and provide a point of comparison with other small-bodied metoposaurid specimens. A complete osteology of the metoposaurids of the NK quarry awaits further preparation. The interclavicles from NK are typical for metoposaurids and, like other North American metoposaurids, have a wide region of reticulate ornamentation (Fig. 18). A slight offset posterior process is present in one specimen (Fig. 18E) like that present in Otis Chalk Quarry 2 (Figs. 17A, 17B) and other specimens of *B. bakeri* (figs. 55–56: Gee & Kufner, 2022). An unusual observation from the interclavicles at NK is that most specimens have a region of reticulate ornamentation that is softer than the surrounding element resulting in poor preservation of the ornament at the center of the interclavicle. Differences in histological character or preservation of the interclavicles could be determined in thin section.

The largest clavicles from NK figured here (Figs. 19A–19F) are indistinguishable from previously described specimens of *B. bakeri* (figs. 51–52: Gee & Kufner, 2022), but the smallest clavicle exhibits a marked and prolonged anteromedial contact (Figs. 19G, 19H) much like the small clavicles from Otis Chalk Quarry 2 (Figs. 17D, 17E) and a small juvenile specimen of *Apachesaurus gregorii* (figs. 7a, c: Rinehart & Lucas, 2018). The clavicles from NK are very similar to clavicles from EP in having both a wide region of reticulate ornamentation along the posterolateral margin of the ventral surface and a sensory groove interrupting this reticulate ornament (Fig. 19). The sensory groove in the smallest clavicle (Fig. 19G) also exhibits some low ridges within the sulcus, but this is not as pronounced as that observed in TMM 31099-34 (Fig. 17D). In dorsal view, all NK clavicles have the typical ‘trough’ alongside the dorsal lamina (Figs. 19B, 19D, 19F, 19H), and the ascending process of the clavicle is well preserved in two NK clavicles (Figs. 19D, 19H). In addition to the pectoral girdle and axial elements of the postcranial skeleton, photographs of select ilia from NK are also provided in File S1, but these are indistinguishable from ilia of EP (Fig. S2).

*Anaschisma* Branson, 1905 *sensu* Gee, Parker & Marsh, 2020

Diagnosis—. As for species by monotypy.

*Anaschisma browni* Branson, 1905 *sensu* Gee, Parker & Marsh, 2020

Synonyms—. *Anaschisma brachygnatha* Branson, 1905

*Koskinonodon princeps* Branson & Mehl, 1929

*Borborophagus wyomingensis* Branson & Mehl, 1929

*Eupelor browni* Colbert & Imbrie, 1956

*Metoposaurus browni* RoyChowdhury, 1965

*Buettneria perfecta* (in part) Hunt, 1993

*Koskinonodon perfectus* (in part) Mueller, 2007

*Koskinonodon perfectum* (in part) Spielmann & Lucas, 2012

*Koskinonodon perfecta* (in part) Brusatte et al., 2015

Figures 20–23

**Figure 20:**
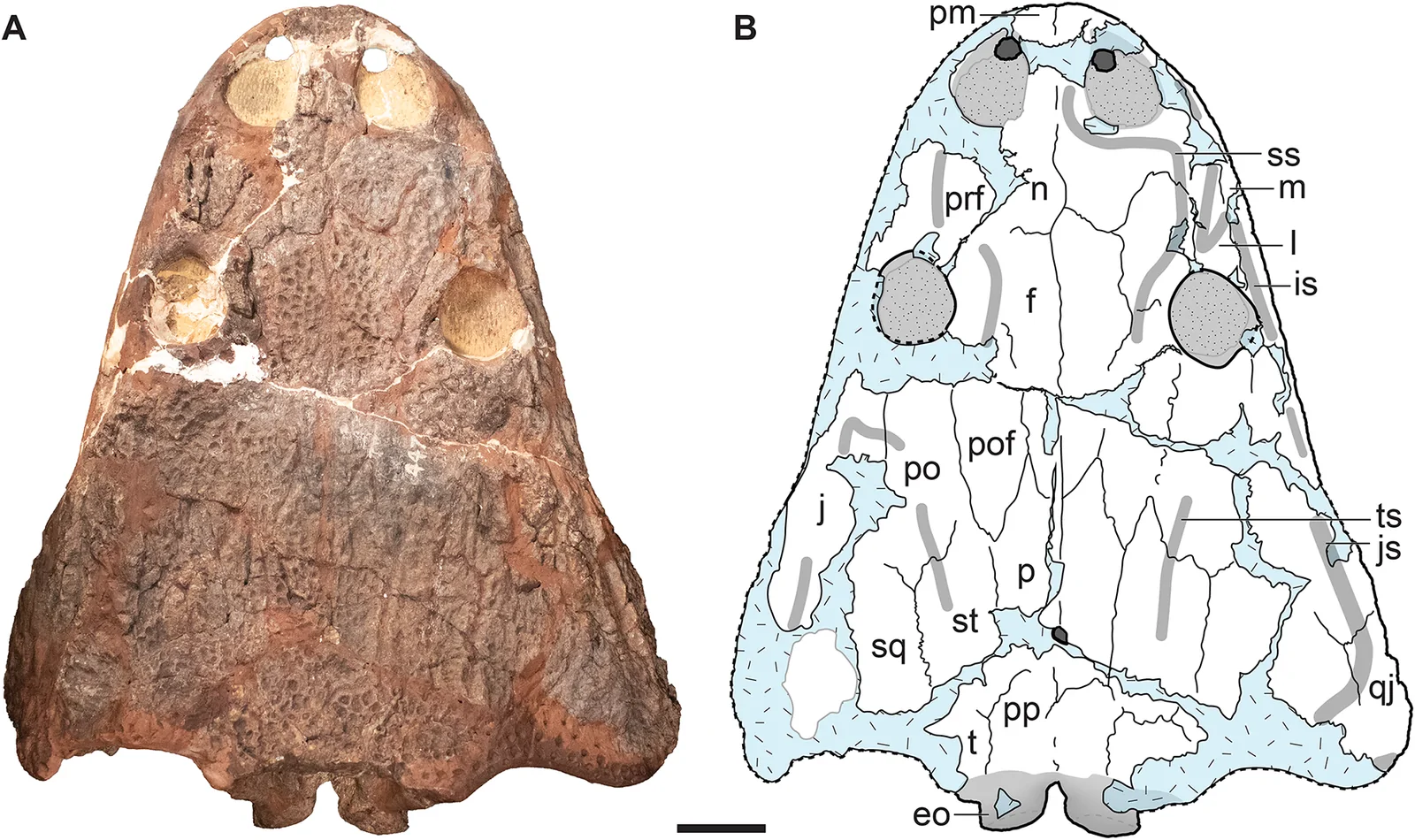
Holotype cranium of *Anaschisma browni*, FMNH UC 447, from Willow Creek in Fremont County, WY in dorsal view. (A) Photograph; (B) interpretive drawing. Blue hatching represents reconstruction; stippling represents residual matrix; fine dashed gray lines represent topographic details like ridges. Abbreviations: eo, exoccipital; f, frontal; is, infraorbital sulcus; j, jugal; js, jugal sulcus; l, lacrimal; m, maxilla; n, nasal; p, parietal; pm, premaxilla; po, postorbital; pof, postfrontal; pp, postparietal; prf, prefrontal; qj, quadratojugal; sq, squamosal; ss, supraorbital sulcus; st, supratemporal; t, tabular; ts, temporal sulcus. Scale bar equals 5 cm.

**Figure 21:**
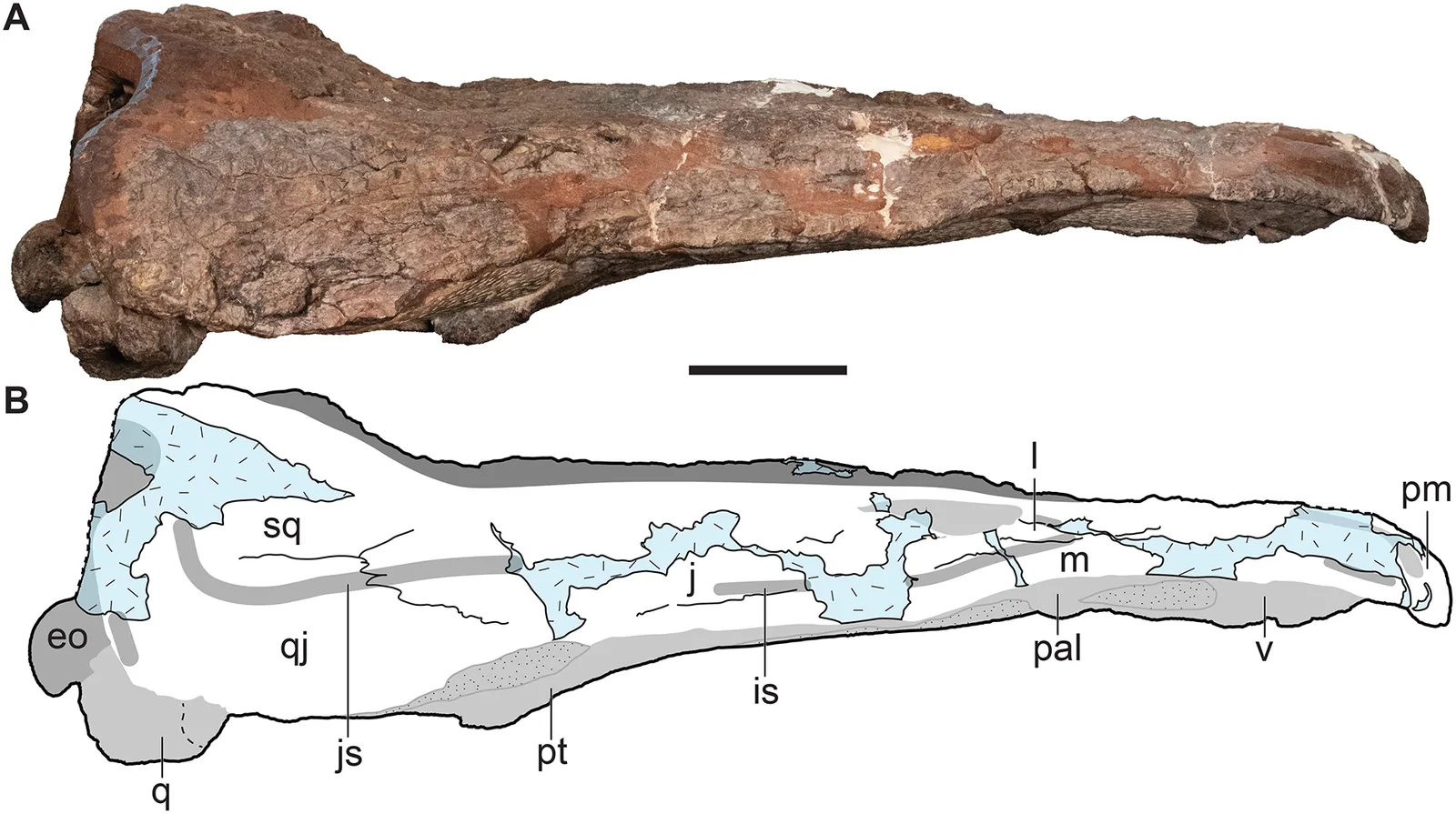
Holotype cranium of *Anaschisma browni*, FMNH UC 447, from Willow Creek in Fremont County, WY in right lateral view. (A) Photograph; (B) interpretive drawing. Blue hatching represents reconstruction; stippling represents residual matrix. Abbreviations: eo, exoccipital; is, infraorbital sulcus; j, jugal; js, jugal sulcus; l, lacrimal; m, maxilla; pal, palatine; pm, premaxilla; pt, pterygoid; q, quadrate; qj, quadratojugal; sq, squamosal; v, vomer. Scale bar equals 5 cm.

**Figure 22:**
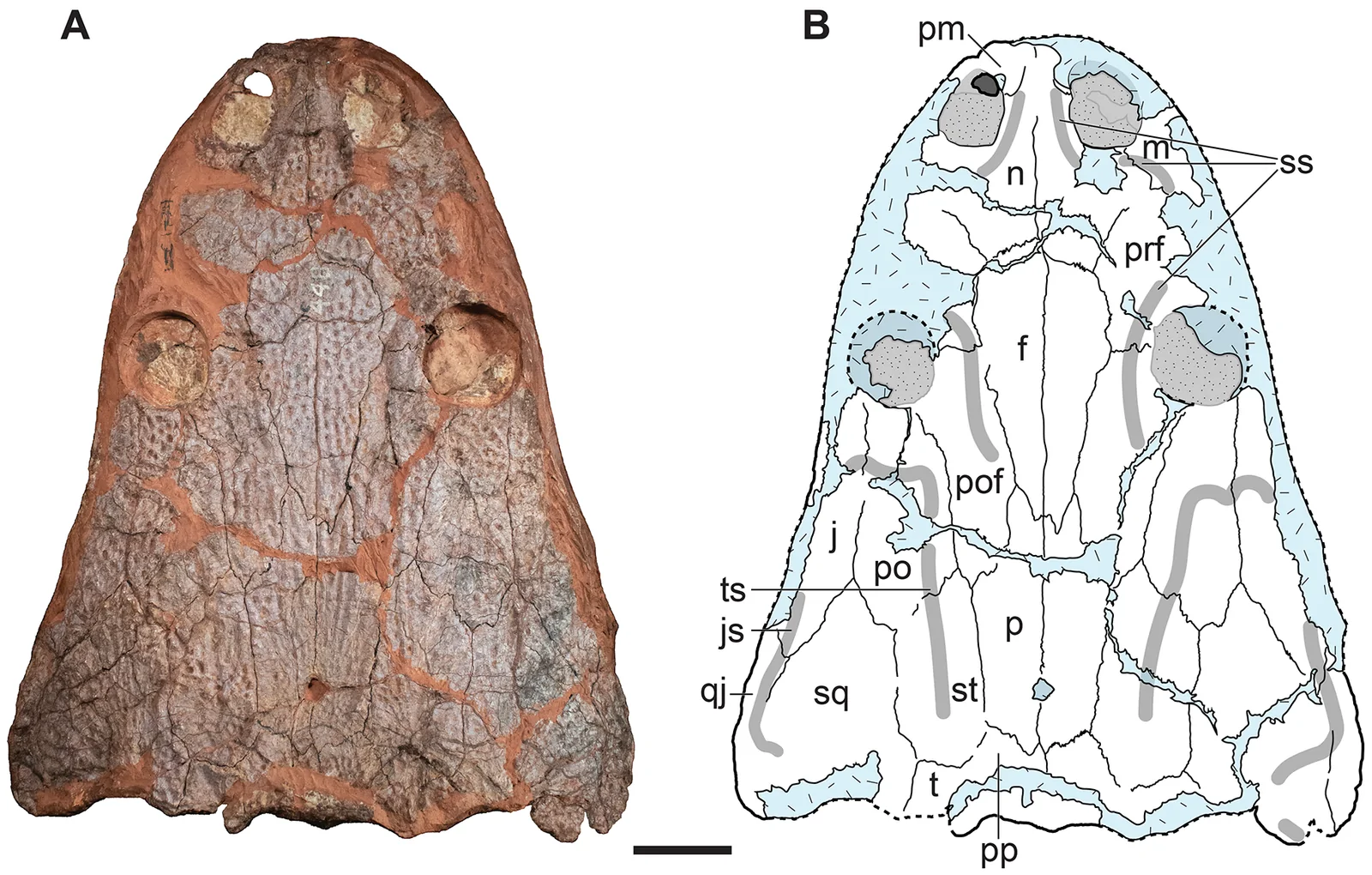
Holotype cranium of “*Anaschisma brachygnatha*” (=*Anaschisma browni*), FMNH UC 448, from Willow Creek in Fremont County, WY in dorsal view. (A) Photograph; (B) interpretive drawing. Blue hatching represents reconstruction; stippling represents residual matrix. Abbreviations: f, frontal; j, jugal; js, jugal sulcus; m, maxilla; n, nasal; p, parietal; pm, premaxilla; po, postorbital; pof, postfrontal; pp, postparietal; prf, prefrontal; qj, quadratojugal; sq, squamosal; ss, supraorbital sulcus; st, supratemporal; t, tabular; ts, temporal sulcus. Scale bar equals 5 cm.

**Figure 23:**
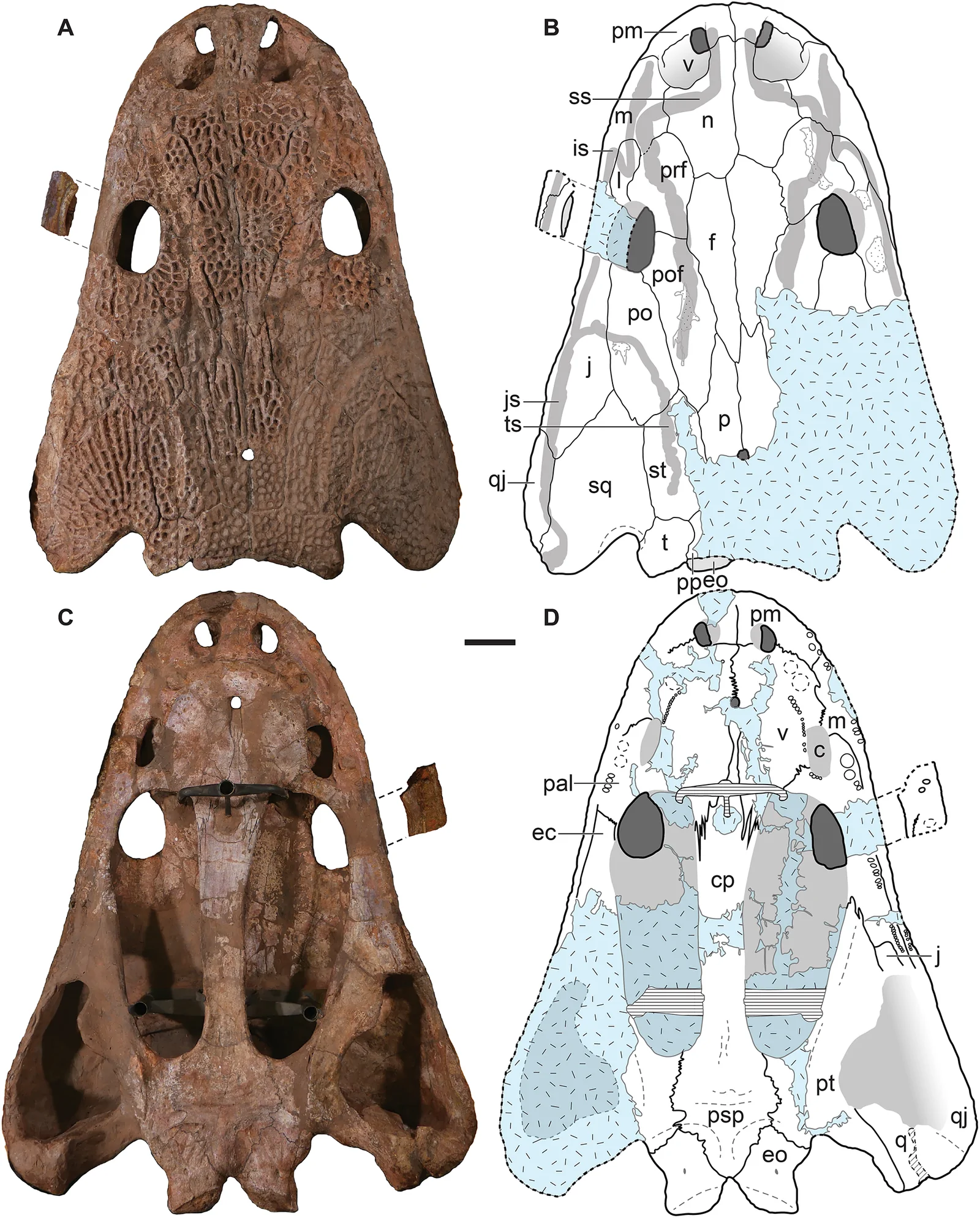
Holotype cranium of “*Koskinonodon princeps*” (=*Anaschisma browni*), MU 537, from Bull Lake Creek in Fremont County, WY. (A) Photograph in dorsal view; (B) interpretive drawing of the same; (C) photograph in ventral view; (D) interpretive drawing of the same. Blue hatching represents reconstruction; stippling represents residual matrix; fine dashed gray lines represent topographic details like ridges; horizontal lines represent mounting armature. Abbreviations: c, choana (internal naris); cp, cultriform process; ec, ectopterygoid; eo, exoccipital; f, frontal; is, infraorbital sulcus; j, jugal; js, jugal sulcus; l, lacrimal; m, maxilla; n, nasal; p, parietal; pal, palatine; pm, premaxilla; po, postorbital; pof, postfrontal; pp, postparietal; prf, prefrontal; psp, parasphenoid; pt, pterygoid; q, quadrate; qj, quadratojugal; sq, squamosal; ss, supraorbital sulcus; st, supratemporal; t, tabular; ts, temporal sulcus; v, vomer. Scale bar equals 5 cm.

Holotype—. FMNH UC 447, an essentially complete skull missing much of the posterior skull roof margin.

Referred specimens—. MU 517, holotype cranium of *Borborophagus wyomingensis*; MU 537, holotype cranium of *Koskinonodon princeps*; MU 504, nearly complete cranium covered in plaster; MU 527, partial left cranium; MU 539, partial right hemimandible; MU 567, right hemimandible; MU 568, left hemimandible and associated fang; MU 578, partial trunk intercentrum; MU 583, three intercentra: anterior dorsal, presacral, and postsacral; MU 506, MU 519, MU 521, MU 522, and MU 542, interclavicles; MU 513, associated interclavicle and left and right clavicles found with the holotype cranium of *B. wyomingensis*; MU 508, MU 510, and MU 512, left clavicles; MU 556, right ilium; FMNH UC 448, holotype cranium of *Anaschisma brachygnatha* and an associated right hemimandible.

Emended diagnosis—. Metoposaurid differentiated from all other metoposaurids except *Buettnererpeton bakeri*, *Apachesaurus gregorii*, *Tecovaserpeton perfectum*, *Paranaschisma howardensis*, *Panthasaurus maleriensis*, and “*Arganasaurus*” *azerouali* by a wide region of reticulate ornamentation on the posterolateral side of the clavicle. Differentiated from *B. bakeri*, “*Ar*.” *azerouali*, *Pan. maleriensis*, and *Par. howardensis* (in part) by the absence of a sensory groove on the posterolateral margin of the clavicle. Further differentiated from *B. bakeri* by a lacrimal that enters the orbital margin, anterior orbital margin positioned at or slightly posterior to the anterior margin of the interpterygoid vacuity, and the absence of an accessory jugal sulcus. Further differentiated from “*Ar*.” *azerouali* by a lack of a commissure between the supraorbital and postorbital (=temporal sulcus + jugal sulcus) lateral line sulci. Differentiated from *A. gregorii* (in part), *Par. howardensis* (in part), and *T. perfectum* by the absence of a transverse occipital sulcus in adults and a relatively narrow anterior end of the prefrontal in adults. Further differentiated from *Par. howardensis* by the absence of a posteromedial extension of the ectopterygoid and by an *insula jugalis* that occupies less than half of the transverse width of the palatal ramus of pterygoid. Further differentiated from *Pan*. *maleriensis* by a maxilla-prefrontal contact, a ‘Z-shaped’ flexure of the infraorbital sulcus on the lacrimal, and a parasagittally oriented parasphenoid-pterygoid suture (not oblique to the midline). Differentiated from *T. perfectum* and *A. gregorii* by a dorsally positioned notochordal canal. Further differentiated from *A. gregorii* by a temporal sulcus that terminates on the supratemporal and a relatively wide cultriform process.

Type locality and horizon—. The Willow Creek locality in the “ochre unit” of the Popo Agie Formation of Fremont County, WY.

Remarks—. Recent redescriptions of the holotype of *Anaschisma browni* and junior subjective synonyms (Gee, Parker & Marsh, 2020; Kufner & Gee, 2021) have served to clarify the anatomy of this species although some authors maintain the status of *A. browni* as a *nomen dubium* (Lucas, 2020; Rinehart, Lucas & Heckert, 2024). The diagnosis of *A. browni* is further emended here to demonstrate both the validity of the species and to differentiate it from previously synonymized species.

**Description**

Due to the recent redescriptions of the holotype and referred specimens of *Anaschisma browni*, the descriptions here are kept brief, and the reader is referred to those previous studies (Gee, Parker & Marsh, 2020; Kufner & Gee, 2021). My firsthand study of FMNH UC 447 confirms the “bone map” proposed by Gee, Parker & Marsh (2020; *contra* Lucas, 2020) including the position of the lacrimal relative to the orbital margin which can be discerned in the right antorbital region. The only appreciable difference from the redescription of FMNH UC 447 by Gee, Parker & Marsh (2020) presented here is the further identification of lateral line sulci. The infraorbital sulcus was previously considered entirely reconstructed, but a Z-shaped flexure of the infraorbital sulcus was identified across the right lacrimal (Fig. 20; *contra* Gee, Parker & Marsh, 2020). The infraorbital sulcus follows its typical pathway lateral to the orbit following the lacrimal-maxilla suture for a short distance then following the presumed position of the maxilla-jugal suture. Only a short segment of the infraorbital sulcus is evident posterior to the orbital margin. The infraorbital sulcus is more clearly visible in lateral view following the maxilla-jugal suture, but damage in this region prevents identification of a commissure between the infraorbital and jugal sulci (Fig. 21). The jugal sulcus reaches the posterior skull roof margin (*contra* Gee, Parker & Marsh, 2020) with a medially inflected curve that reaches the reconstructed part of the posterior skull roof, but the continuation of the jugal sulcus can be seen along the posterior margin of the squamosal or the quadratojugal (Figs. 20, 21). No accessory jugal sulcus is evident on the quadratojugal (Fig. 21). In lateral view, the premaxilla is slightly ventrally deflected (Fig. 21) as noted for some specimens of *B. bakeri* (Figs. 7, 8). This ventral deflection of the premaxilla is also present in MU 517 and MU 537 the holotypes of “*Borborophagus wyomingensis*” (=*A. browni*) and “*Koskinonodon princeps*” (=*A. browni*), respectively (A. Kufner, 2019, personal observations).

Along with FMNH UC 447, Gee, Parker & Marsh (2020) provided an updated description of FMNH UC 448—the holotype of “*Anaschisma brachygnatha*”— and similarly only differences in interpretation are noted. The authors suggested the presence of a parietal-postorbital contact on the right side of this specimen, however, the “suture” used to infer this contact is instead a crack through the posterior process of the postfrontal that continues transversely across the posterior process of the postorbital (Fig. 22; fig. 3: Gee, Parker & Marsh, 2020). There is significant reconstruction in this region, and the fracture through the postfrontal is consistent with damage seen elsewhere on the specimen but inconsistent with nearby sutures (*i.e*., hairline, irregular, no interdigitation). Some metoposaurid specimens have a parietal-postorbital contact, but this is the result of a wide parietal with a straight lateral margin that is more steeply angled away from the midline (Buffa, Jalil & Steyer, 2019; Chakravorti & Sengupta, 2019; Sulej, 2007) and not the step-like lateral margin suggested by Gee, Parker & Marsh (2020) which is unknown among metoposaurids with a parietal-postorbital contact and indeed in all described metoposaurid specimens. However, the parietal-supratemporal suture is more steeply angled away from the midline in FMNH UC 448 than FMNH UC 447. The only other appreciable difference of the skull roof is the sinusoidal rather than straight morphology of the commissure between the temporal and jugal sulci (Fig. 22; fig. 3: Gee, Parker & Marsh, 2020), which is more typical of that seen widely among metoposaurids. Like FMNH UC 447, the jugal sulcus exhibits a medial inflection at its posterior extent and a deeper groove along the posterior margin of the skull roof appears to be the continuation of the jugal sulcus although surficial damage in this area inhibits confident identification. In addition to these figured differences, faint ornamentation can be seen on the ventral surface of both the basal plate of the parasphenoid and the cultriform process with a raking light.

A recent redescription by Kufner & Gee (2021) provided the first thorough description the anatomy of MU 537, the holotype of “*Koskinonodon princeps*,” since the initial description (Branson & Mehl, 1929) and showed extensive reconstruction of the right posterolateral skull roof. An updated interpretation of the skull roof and palate of MU 537 is provided here differing only in the inclusion of sutures within the external naris, slight correction of the lateral line sulci, and some cosmetic differences (Fig. 23). Like FMNH UC 447, the lacrimal of MU 537 is elongate and narrow with a posterior curve toward the orbit differing from most stratigraphically higher metoposaurids primarily from Texas, Arizona, and New Mexico previously referred to *Anaschisma browni* (see below). The supraorbital sulcus is wider than depicted by Kufner & Gee (2021), but regardless, it does not contact the lacrimal (Figs. 23A, 23B). Notably, for comparison with other previously synonymized species, MU 537 entirely lacks a transverse occipital sulcus and confirms the absence of an accessory jugal sulcus in *A. browni* like in FMNH UC 447 and FMNH UC 448.

*Paranaschisma* gen. nov.

Diagnosis—. As for species by monotypy.

Etymology—. From the Ancient Greek “*para*-” meaning “near” or “alongside” and “*Anaschisma*” a genus of metoposaurid for which an etymology was never rendered (Branson, 1905; Gee, Parker & Marsh, 2020). The name refers to the possible contemporaneity of the two genera and previously proposed synonymy of the species *Anaschisma browni* and “*Buettneria*” *howardensis*.

*Paranaschisma howardensis* Sawin, 1944

Synonyms—. *Buettneria howardensis* Sawin, 1944

*Eupelor fraasi jonesi* (in part) Colbert & Imbrie, 1956

*Metoposaurus fraasi jonesi* (in part) RoyChowdhury, 1965

*Metoposaurus fraasi* (in part) Murry, 1986

*Metoposaurus* (“*Buettneria*”) *howardensis* (in part) Murry, 1989

*Koskinonodon perfectus* (in part) Mueller, 2007

*Koskinonodon perfectum* (in part) Spielmann & Lucas, 2012

*Koskinonodon perfecta* (in part) Brusatte et al., 2015

*Koskinonodon howardensis* Chakravorti & Sengupta, 2019

*Anaschisma browni* (in part) Gee, Parker & Marsh, 2020

Figures 24–33

**Figure 24:**
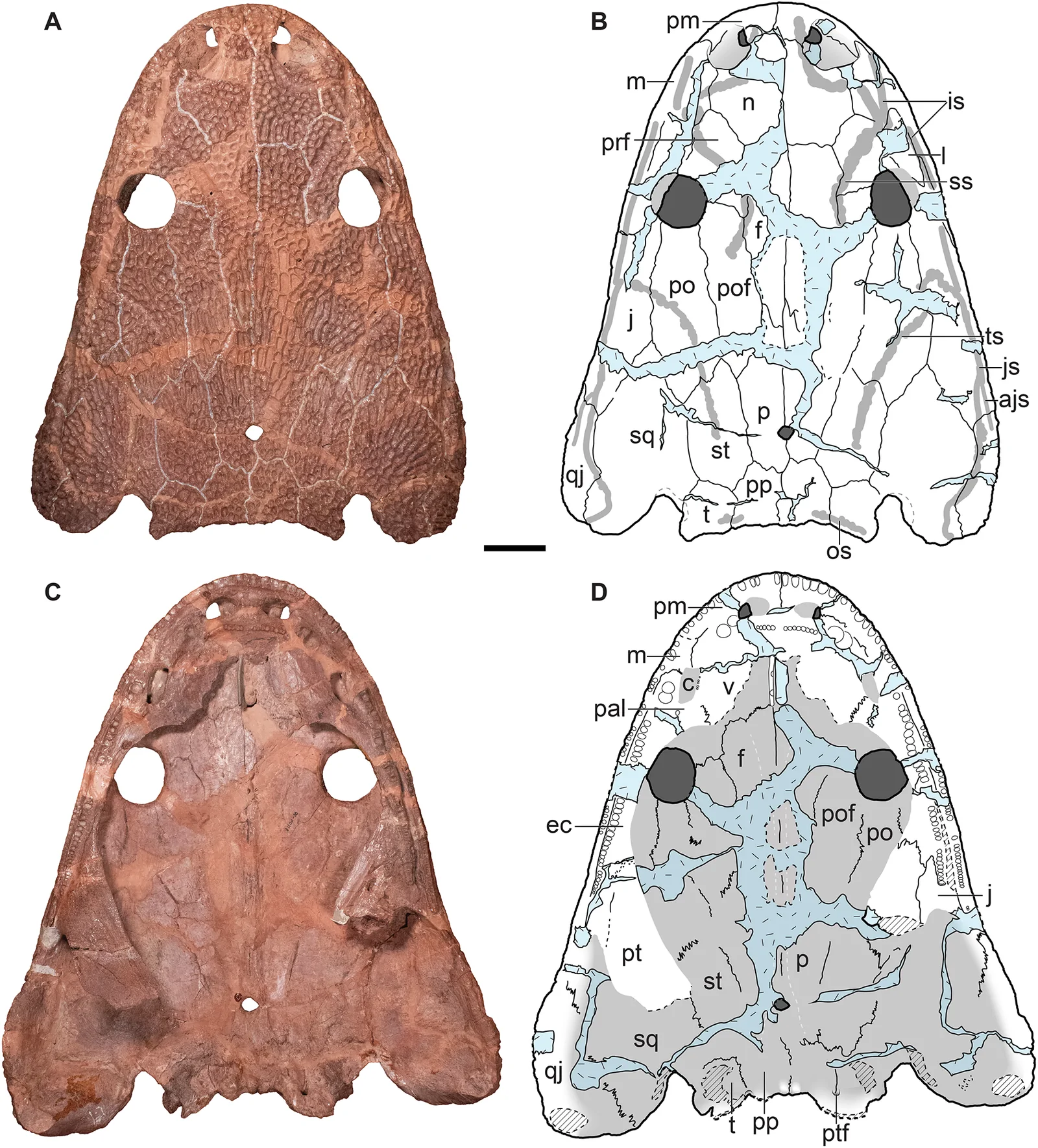
Lectotype partial cranium of *Paranaschisma howardensis*, TMM 31100-30, from Otis Chalk Quarry 3 in Howard County, TX. (A) Photograph in dorsal view; (B) interpretive drawing of the same; (C) photograph in ventral view; (D) interpretive drawing of the same. Blue hatching represents reconstruction; fine dashed gray lines represent topographic details like ridges; diagonal lines represent broken surfaces; open circles represent a metal rod for support. Abbreviations: ajs, accessory jugal sulcus; c, choana (internal naris); ec, ectopterygoid; f, frontal; is, infraorbital sulcus; j, jugal; js, jugal sulcus; l, lacrimal; m, maxilla; n, nasal; os, transverse occipital sulcus; p, parietal; pal, palatine; pm, premaxilla; po, postorbital; pof, postfrontal; pp, postparietal; prf, prefrontal; pt, pterygoid; ptf, posttemporal fenestra; qj, quadratojugal; sq, squamosal; ss, supraorbital sulcus; st, supratemporal; t, tabular; ts, temporal sulcus; v, vomer. Scale bar equals 5 cm.

**Figure 25:**
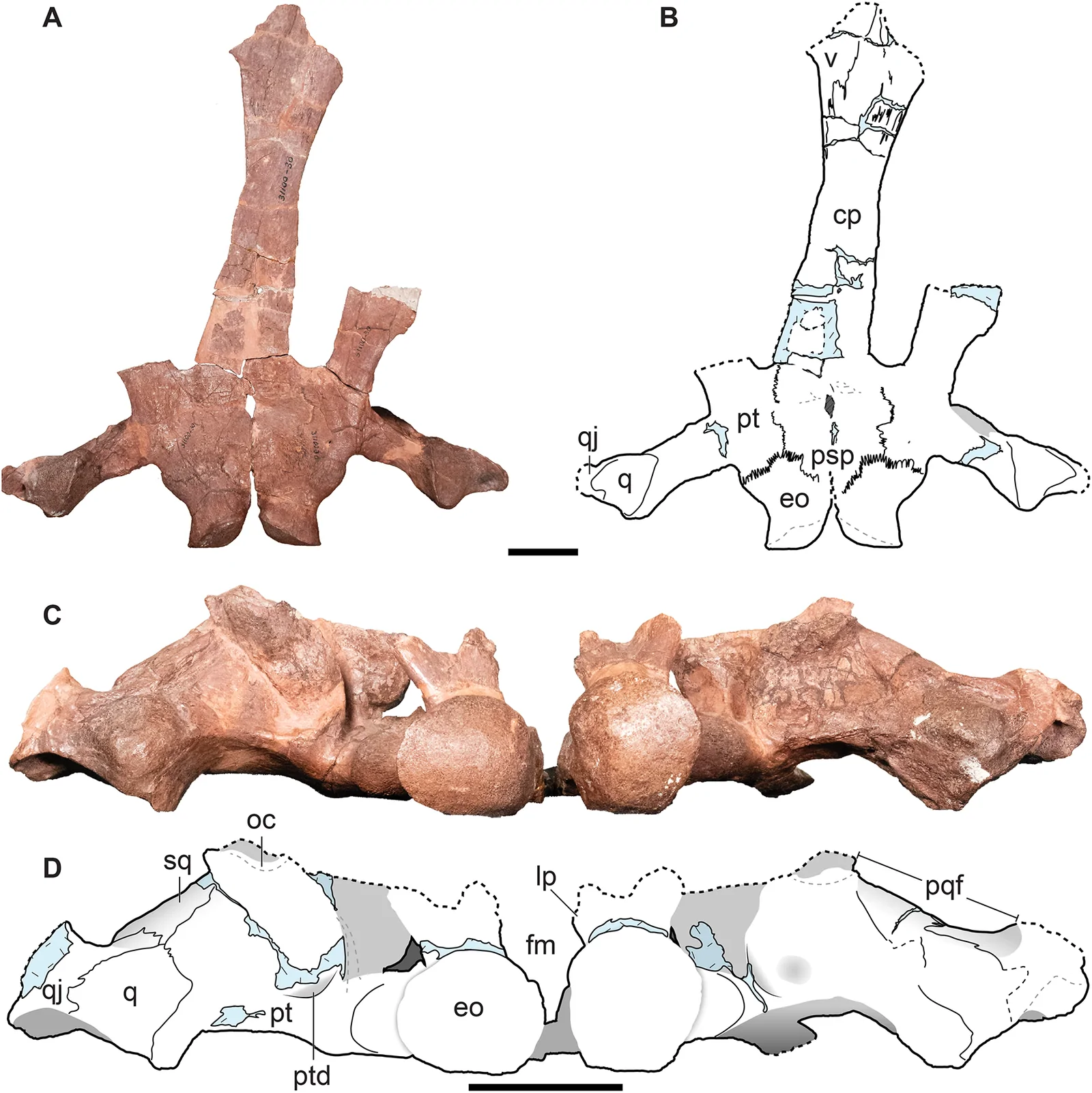
Lectotype “suspensorium” and basicranium of *Paranaschisma howardensis*, TMM 31100-30, from Otis Chalk Quarry 3 in Howard County, TX. Blue hatching represents reconstruction; fine dashed gray lines represent topographic details like ridges. Abbreviations: cp, cultriform process; eo, exoccipital; fm, foramen magnum; lp, lamellose process; oc, oblique crest of the pterygoid; pqf, paraquadrate foramen; psp, parasphenoid; pt, pterygoid; ptd, pterygoid depression; q, quadrate; qj, quadratojugal; v, vomer. Upper scale bar for (A) and (B); lower scale bar for (C) and (D). Scale bars equal 5 cm.

**Figure 26:**
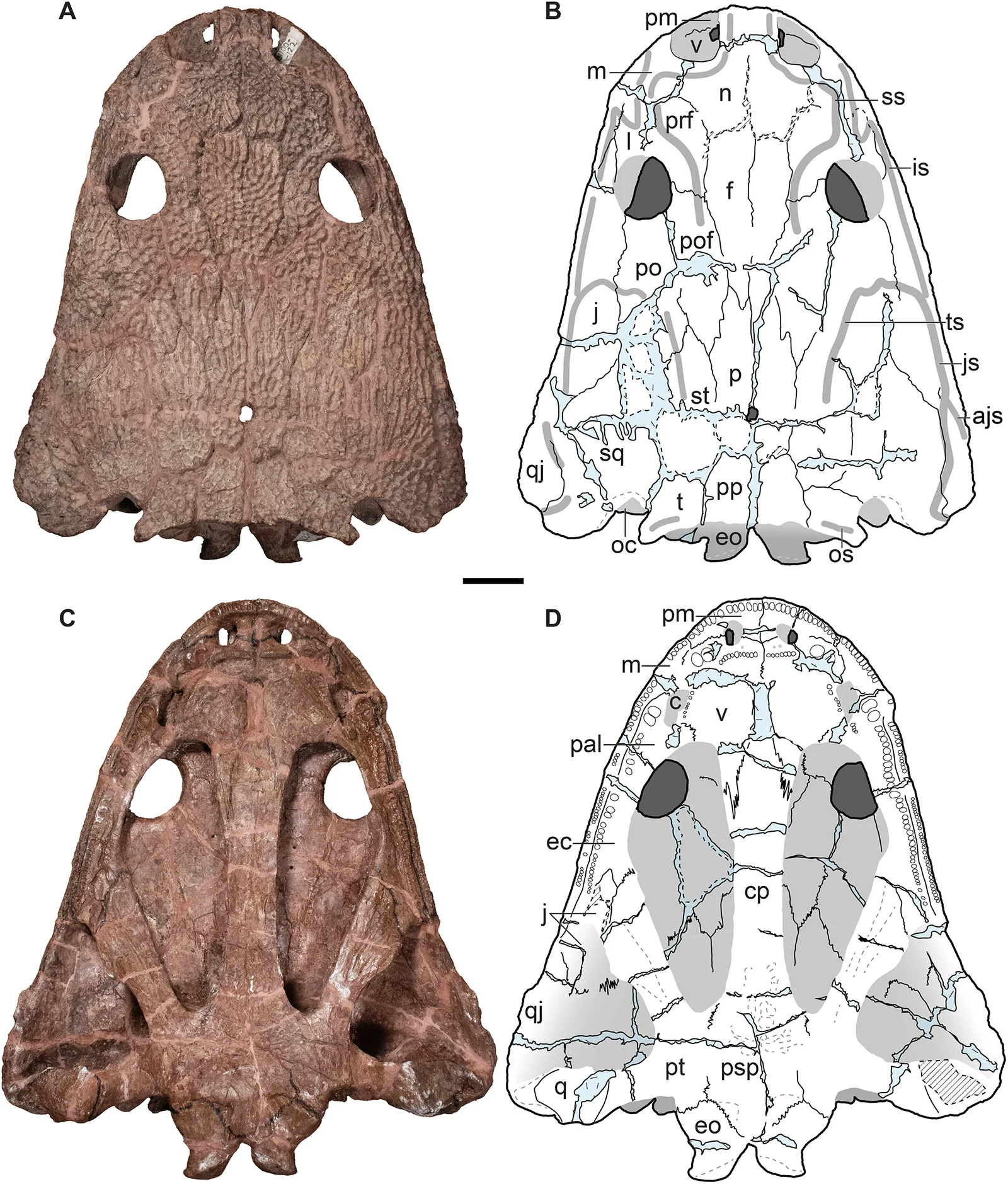
Syntype cranium of *Paranaschisma howardensis*, TMM 31100-122, from Otis Chalk Quarry 3 in Howard County, TX. (A) Photograph in dorsal view; (B) interpretive drawing of the same; (C) photograph in ventral view; (D) interpretive drawing of the same. Blue hatching represents reconstruction; fine dashed gray lines represent topographic details like ridges; diagonal lines represent broken surfaces. Abbreviations: ajs, accessory jugal sulcus; c, choana (internal naris); cp, cultriform process; ec, ectopterygoid; eo, exoccipital; f, frontal; is, infraorbital sulcus; j, jugal; js, jugal sulcus; l, lacrimal; m, maxilla; n, nasal; oc, oblique crest of the pterygoid; os, transverse occipital sulcus; p, parietal; pal, palatine; pm, premaxilla; po, postorbital; pof, postfrontal; pp, postparietal; prf, prefrontal; psp, parasphenoid; pt, pterygoid; q, quadrate; qj, quadratojugal; sq, squamosal; ss, supraorbital sulcus; st, supratemporal; t, tabular; ts, temporal sulcus; v, vomer. Scale bar equals 5 cm.

**Figure 27:**
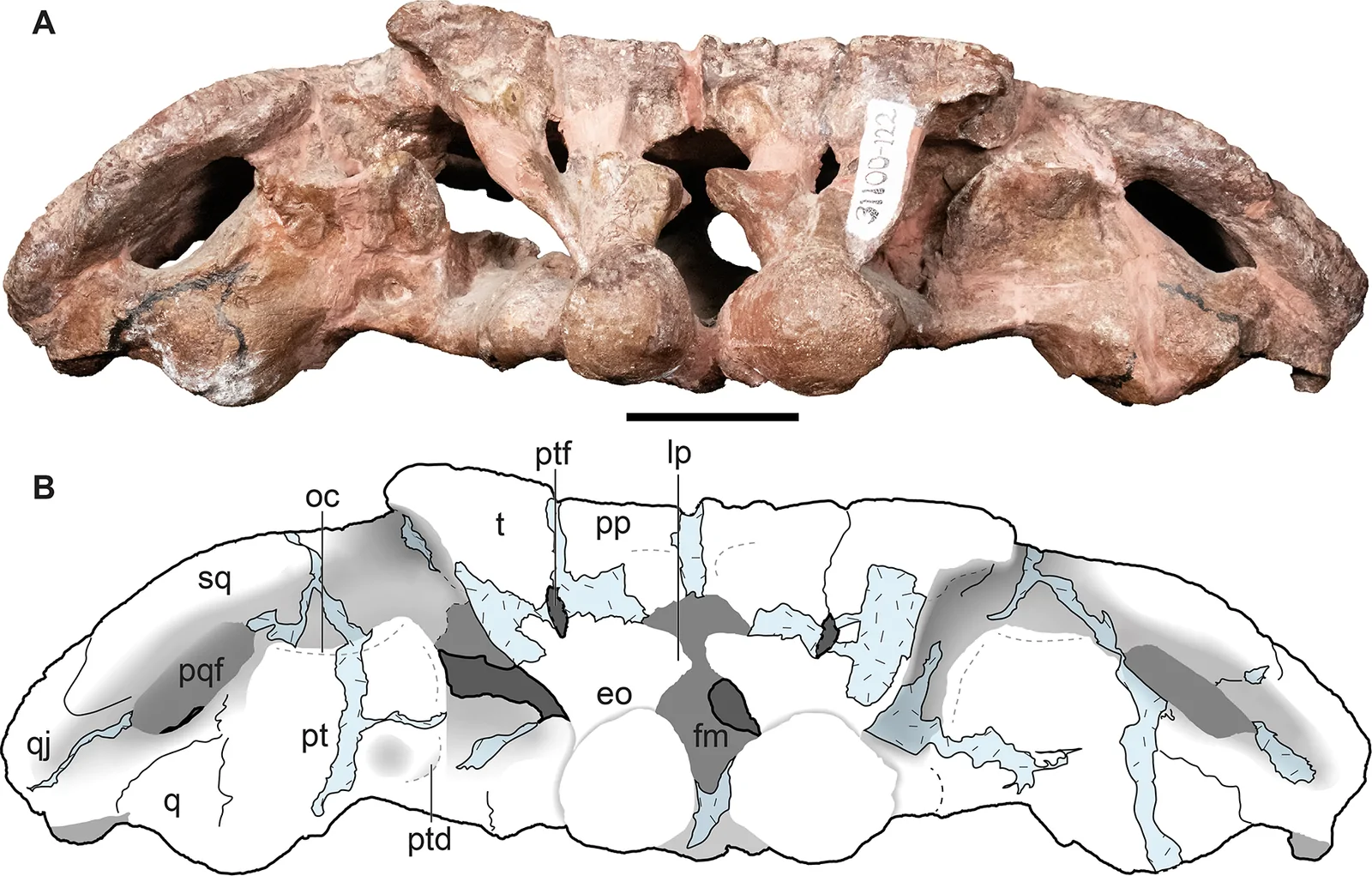
Syntype cranium of *Paranaschisma howardensis*, TMM 31100-122, from Otis Chalk Quarry 3 in Howard County, TX in occipital view. (A) Photograph; (B) interpretive drawing of the same. Blue hatching represents reconstruction; fine dashed gray lines represent topographic details like ridges. Abbreviations: eo, exoccipital; fm, foramen magnum; lp, lamellose process; oc, oblique crest of the pterygoid; pp, postparietal; pqf, paraquadrate foramen; pt, pterygoid; ptd, pterygoid depression; ptf, posttemporal fenestra; q, quadrate; qj, quadratojugal; sq, squamosal; t, tabular. Scale bar equals 5 cm.

**Figure 28:**
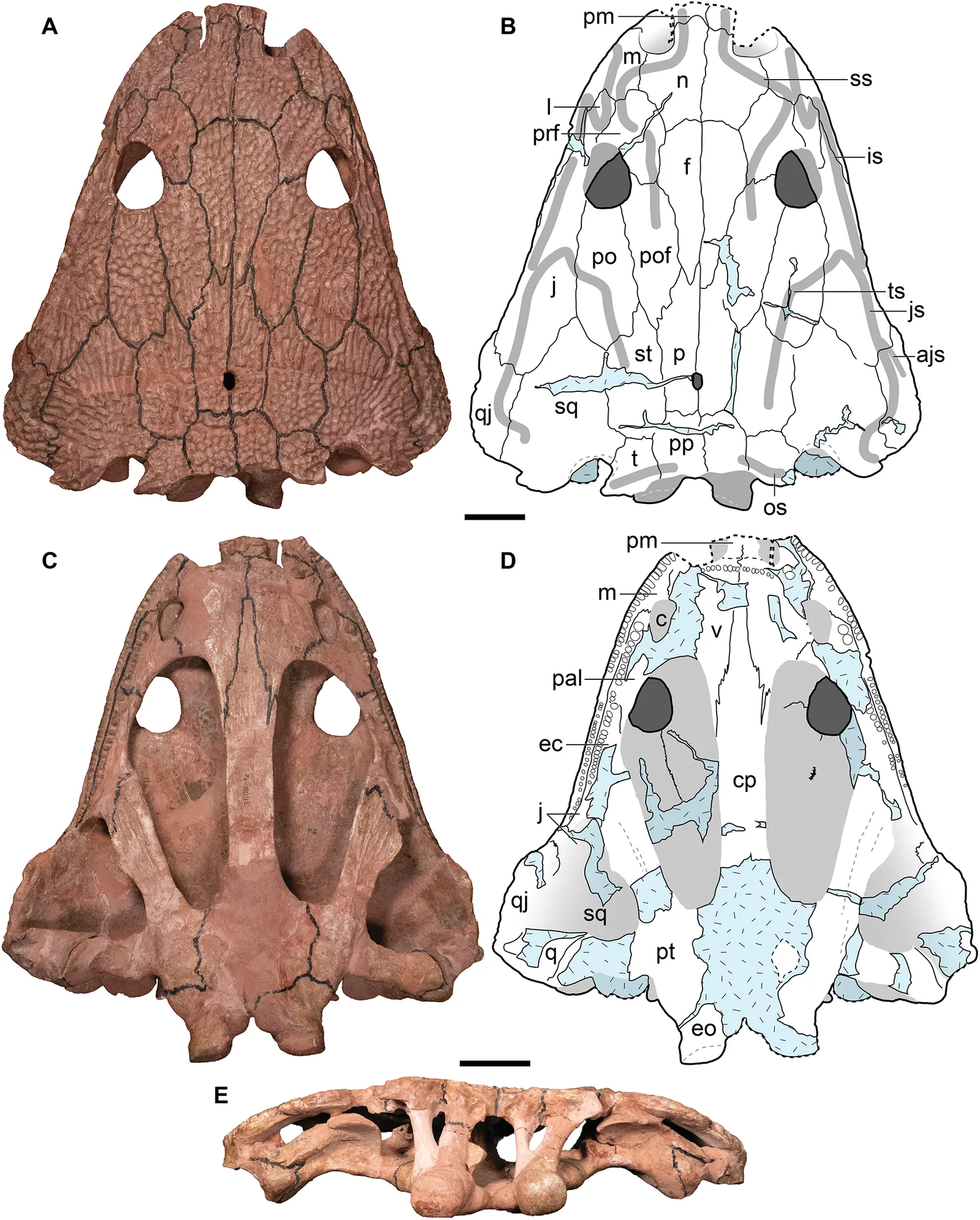
Syntype cranium of *Paranaschisma howardensis*, TMM 31100-42, from Otis Chalk Quarry 3 in Howard County, TX. (A) Photograph in dorsal view; (B) interpretive drawing of the same; (C) photograph in ventral view; (D) interpretive drawing of the same; (E) photograph in occipital view. Blue hatching represents reconstruction; fine dashed gray lines represent topographic details like ridges. Abbreviations: ajs, accessory jugal sulcus; c, choana (internal naris); cp, cultriform process of the parasphenoid; ec, ectopterygoid; eo, exoccipital; f, frontal; is, infraorbital sulcus; j, jugal; js, jugal sulcus; l, lacrimal; m, maxilla; n, nasal; os, transverse occipital sulcus; p, parietal; pal, palatine; pm, premaxilla; po, postorbital; pof, postfrontal; pp, postparietal; prf, prefrontal; pt, pterygoid; q, quadrate; qj, quadratojugal; sq, squamosal; ss, supraorbital sulcus; st, supratemporal; t, tabular; ts, temporal sulcus; v, vomer. Upper scale bar for (A–D); lower scale bar for (E). Scale bars equal 5 cm.

**Figure 29:**
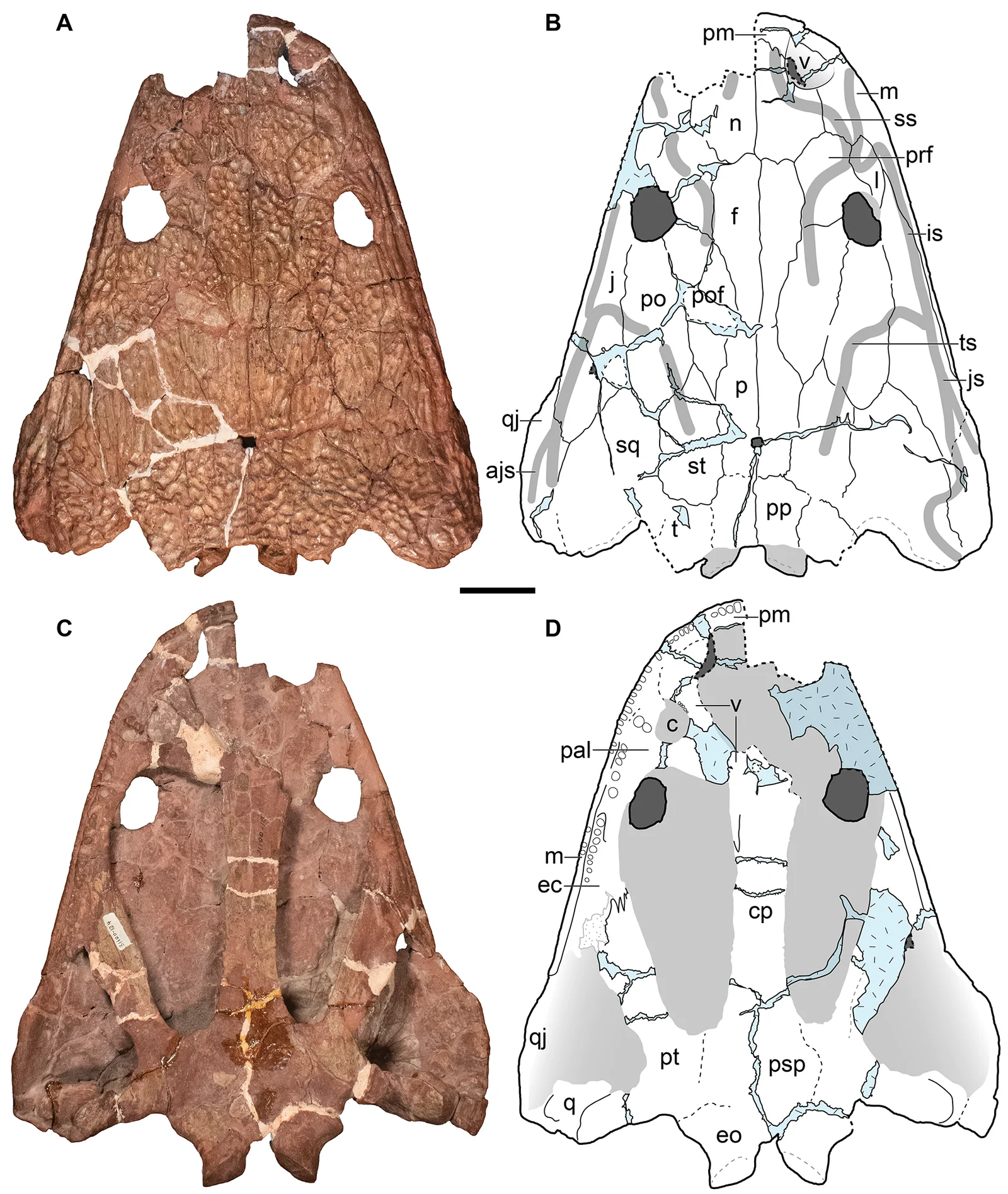
Syntype cranium of *Paranaschisma howardensis*, TMM 31100-124, from Otis Chalk Quarry 3 in Howard County, TX. (A) Photograph in dorsal view; (B) interpretive drawing of the same; (C) photograph in ventral view; (D) interpretive drawing of the same. Blue hatching represents reconstruction; fine dashed gray lines represent topographic details like ridges; diagonal lines represent broken surfaces. Abbreviations: ajs, accessory jugal sulcus; c, choana (internal naris); cp, cultriform process; ec, ectopterygoid; eo, exoccipital; f, frontal; is, infraorbital sulcus; j, jugal; js, jugal sulcus; l, lacrimal; m, maxilla; n, nasal; p, parietal; pal, palatine; pm, premaxilla; po, postorbital; pof, postfrontal; pp, postparietal; prf, prefrontal; psp, parasphenoid; pt, pterygoid; q, quadrate; qj, quadratojugal; sq, squamosal; ss, supraorbital sulcus; st, supratemporal; t, tabular; ts, temporal sulcus; v, vomer. Scale bar equals 5 cm.

**Figure 30:**
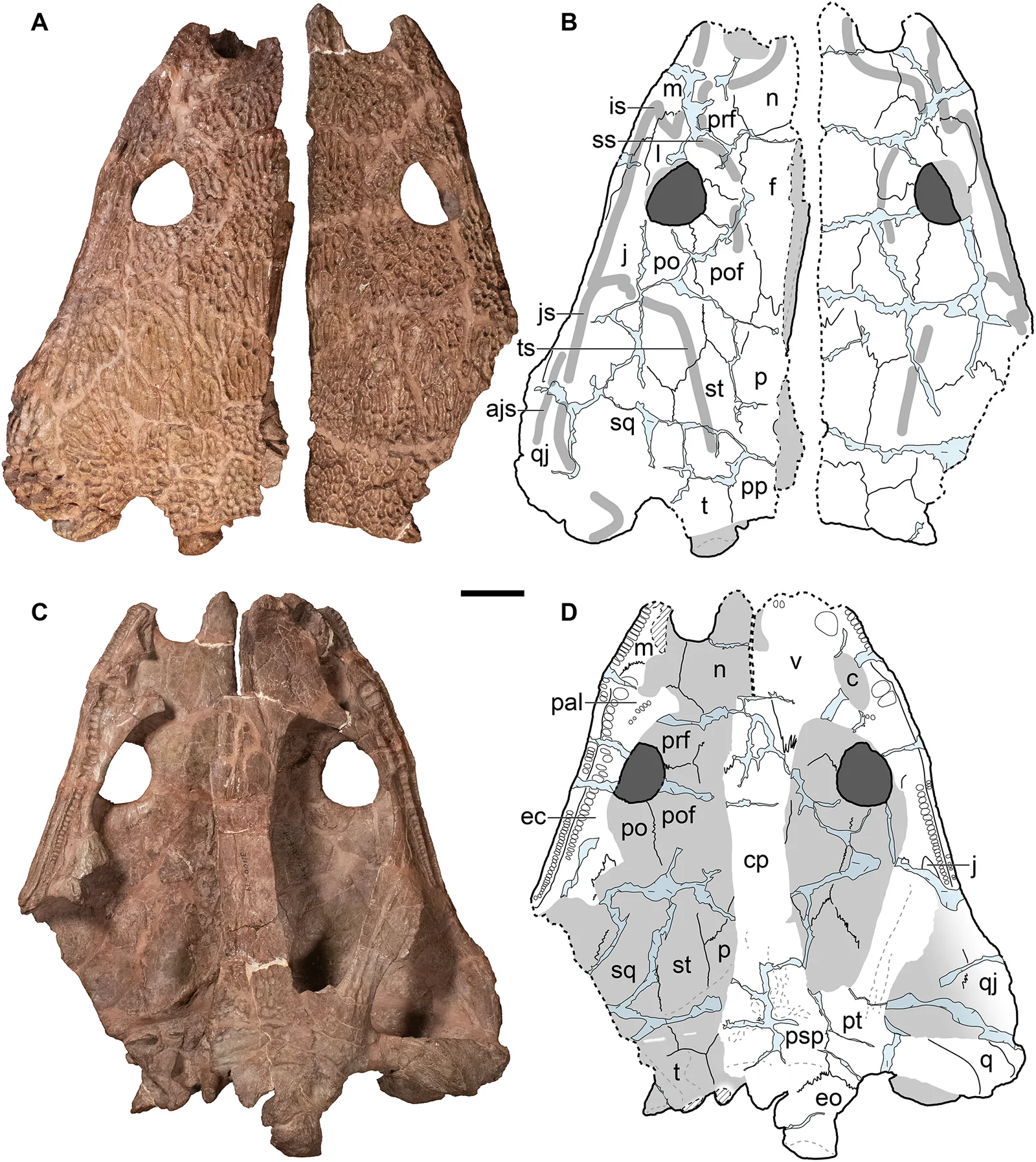
Referred cranium of *Paranaschisma howardensis*, TMM 31100-19, from Otis Chalk Quarry 3 in Howard County, TX. (A) Photograph in dorsal view; (B) interpretive drawing of the same; (C) photograph in ventral view; (D) interpretive drawing of the same. Blue hatching represents reconstruction; fine dashed gray lines represent topographic details like ridges; diagonal lines represent broken surfaces. Abbreviations: ajs, accessory jugal sulcus; c, choana (internal naris); cp, cultriform process; ec, ectopterygoid; eo, exoccipital; f, frontal; is, infraorbital sulcus; j, jugal; js, jugal sulcus; l, lacrimal; m, maxilla; n, nasal; p, parietal; pal, palatine; po, postorbital; pof, postfrontal; pp, postparietal; prf, prefrontal; psp, parasphenoid; pt, pterygoid; q, quadrate; qj, quadratojugal; sq, squamosal; ss, supraorbital sulcus; st, supratemporal; t, tabular; ts, temporal sulcus; v, vomer. Scale bar equals 5 cm.

**Figure 31:**
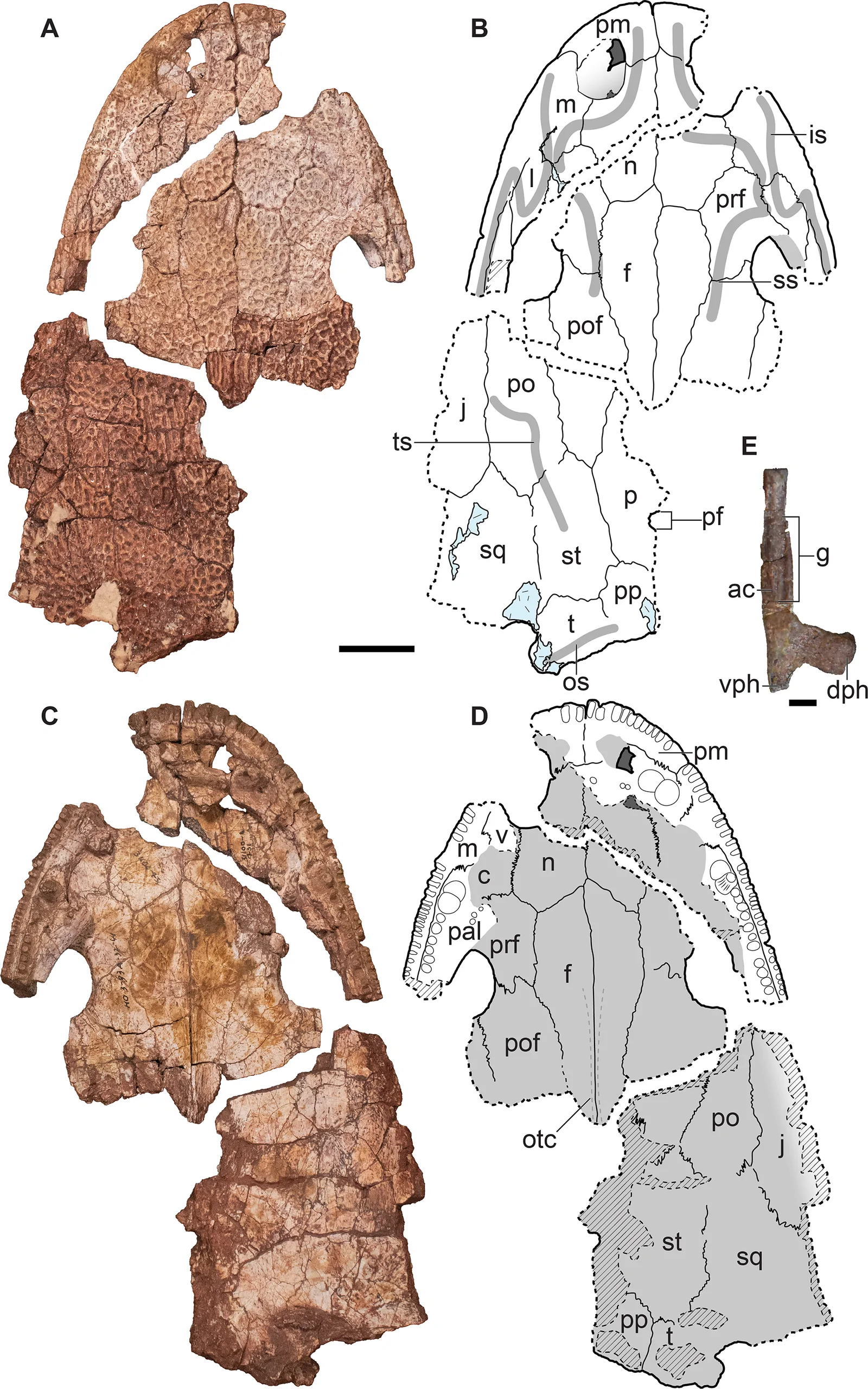
Referred skull roof, partial palate, and left stapes of *Paranaschisma howardensis*, TMM 31100-431, from Otis Chalk Quarry 3 in Howard County, TX. (A) Photograph in dorsal view; (B) interpretive drawing of the same; (C) photograph in ventral view; (D) interpretive drawing of the same; (E) photograph in posteroventral view. Blue hatching represents reconstruction; fine dashed gray lines represent topographic details like ridges; diagonal lines represent broken surfaces. Abbreviations: ac, anterior crest; c, choana (internal naris); dph, dorsal proximal head; f, frontal; g, groove; is, infraorbital sulcus; j, jugal; l, lacrimal; m, maxilla; n, nasal; otc, orbitotemporal crest; p, parietal; pal, palatine; pf, pineal (parietal) foramen; pm, premaxilla; po, postorbital; pof, postfrontal; pp, postparietal; prf, prefrontal; sq, squamosal; ss, supraorbital sulcus; st, supratemporal; t, tabular; ts, temporal sulcus; v, vomer; vph, ventral proximal head. Left scale bar for (A–D) equals 5 cm; right scale bar for (E) equals 1 cm.

**Figure 32:**
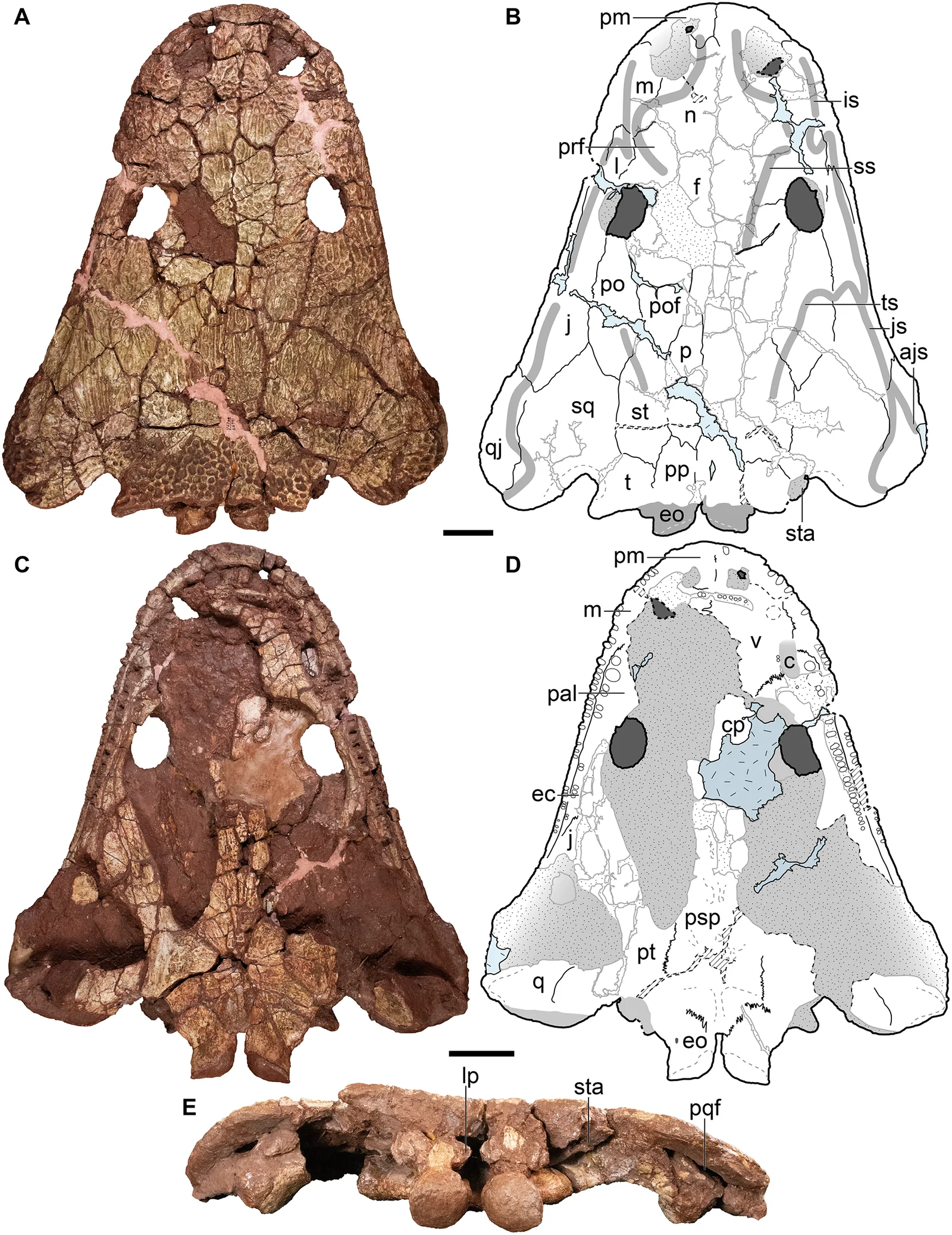
Referred cranium of *Paranaschisma howardensis*, UCMP 66991, from Otis Chalk Quarry 3 in Howard County, TX. (A) Photograph in dorsal view; (B) interpretive drawing of the same; (C) photograph in ventral view; (D) interpretive drawing of the same; (E) photograph in occipital view. Blue hatching represents reconstruction; fine dashed gray lines represent topographic details like ridges; stippling represents residual matrix; diagonal lines represent broken surfaces. Abbreviations: ajs, accessory jugal sulcus; c, choana (internal naris); cp, cultriform process; ec, ectopterygoid; eo, exoccipital; f, frontal; is, infraorbital sulcus; j, jugal; l, lacrimal; lp, lamellose process; m, maxilla; n, nasal; p, parietal; pal, palatine; pm, premaxilla; po, postorbital; pof, postfrontal; pp, postparietal; pqf, paraquadrate foramen; prf, prefrontal; psp, parasphenoid; sq, squamosal; ss, supraorbital sulcus; st, supratemporal; sta, stapes; t, tabular; ts, temporal sulcus; v, vomer. Upper scale bar for (A–D); lower scale bar for (E). Scale bars equal 5 cm.

**Figure 33:**
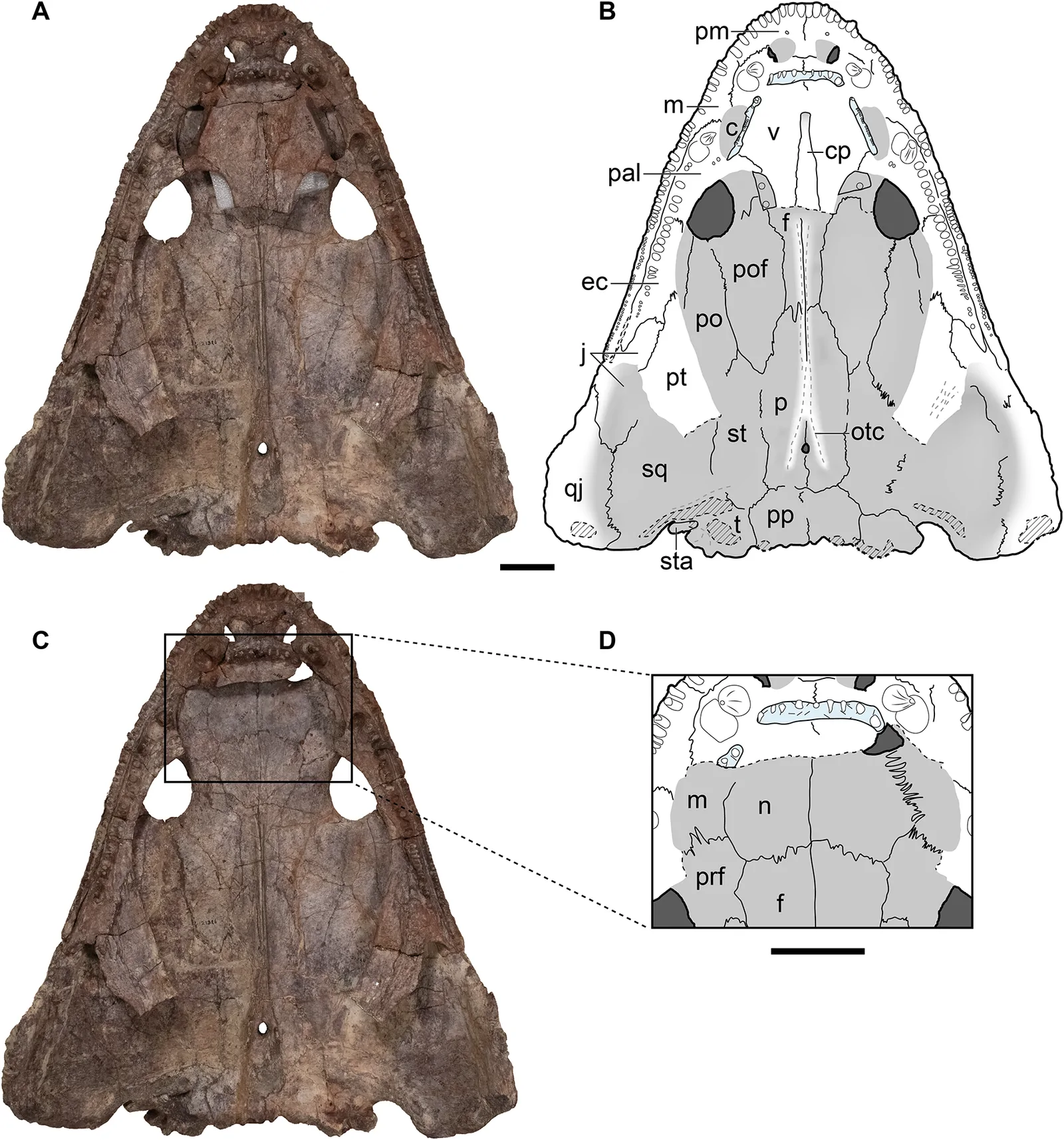
Partial cranium of a metoposaurid cf. *Paranaschisma howardensis*, UMMP 21326, from Cerita de la Cruz Creek in Crosby County, TX in ventral view. (A) Photograph; (B) interpretive drawing; (C) photograph with portion of palate removed; (D) zoomed in interpretive drawing of the skull roof. Blue hatching represents reconstruction; fine dashed gray lines represent topographic details like ridges; open circles represent a block of supporting foam. Abbreviations: c, choana (internal naris); cp, cultriform process of the parasphenoid; ec, ectopterygoid; f, frontal; j, jugal; m, maxilla; n, nasal; otc, orbitotemporal crest; p, parietal; pal, palatine; pm, premaxilla; po, postorbital; pof, postfrontal; pp, postparietal; prf, prefrontal; pt, pterygoid; qj, quadratojugal; sq, squamosal; st, supratemporal; sta, stapes; t, tabular; v, vomer. Upper scale bar for (A–C); lower scale bar for (D). Scale bars equal 5 cm.

Lectotype—. TMM 31100-30, an essentially complete cranium in two pieces.

Syntype specimens—. TMM 31100-42, cranium; TMM 31100-122, cranium; TMM 31100-124, cranium; TMM 31100-161, cranium.

Referred specimens—. TMM 31100-19, nearly complete cranium in two parts; TMM 31100-47, cranium; TMM 31100-431, skull roof and stapes; UCMP 66991, a nearly complete cranium with associated pectoral girdle including interclavicle, both clavicles, scapulocoracoid, humerus, radius, and ulna; UMMP 21326, partial cranium missing basicranium and posterior palate. Numerous metoposaurid postcrania are present in the collection at TMM from Otis Chalk Quarry 3, and these are all likely referable to *Paranaschisma howardensis*. However, many of these remain uncatalogued, and as a result, a list of these postcranial specimens is excluded here and would be better served in a full osteology of the taxon.

Diagnosis—. Metoposaurid differentiated from all other metoposaurids by an extensive *insula jugalis* occupying at least half of the width of the palatal ramus of the pterygoid. Tentatively differentiated from all other metoposaurids except *Panthasaurus maleriensis* by a co-ossified scapulocoracoid in adults. Further differentiated from *P. maleriensis* by a ‘Z-shaped’ flexure of the infraorbital sulcus on the lacrimal and by a parasagittally oriented parasphenoid-pterygoid suture (not oblique to the midline). Further differentiated from all other metoposaurids except *Anaschisma browni*, *Buettnererpeton bakeri*, *Apachesaurus gregorii*, *Tecovaserpeton perfectum*, and “*Arganasaurus*” *azerouali* by a wide region of reticulate ornamentation on the posterolateral side of the clavicle. Differentiated from all other metoposaurids except “*Ar*.” *azerouali* by ornamentation on the palate that extends onto the posterior half of the cultriform process of the parasphenoid in some specimens of the type series. Further differentiated from “*Ar*.” *azerouali* by a lack of a commissure between the supraorbital and postorbital (=temporal sulcus + jugal sulcus) lateral line sulci. Differentiated from all other metoposaurids except possibly large adults of *B. bakeri* (see Figs. 10A, 10B) by a very pronounced lamellose process of the exoccipital, that nearly meets its counterpart at the midline. Further differentiated from *B. bakeri* by a lacrimal that enters the orbital margin, anterior orbital margin positioned at or slightly posterior to the anterior margin of the interpterygoid vacuity, and the presence of a partial transverse occipital sulcus. Further differentiated from *Anaschisma browni* by the presence of an accessory jugal sulcus and a partial transverse occipital sulcus. Differentiated from all other metoposaurids except *Apachesaurus gregorii* by a medial extension of the ectopterygoid onto the palatal ramus. Further differentiated from *Apachesaurus gregorii* by a more dorsally positioned notochordal canal or pit.

Type locality and horizon—. Otis Chalk Quarry 3 in the Colorado City Member of the Dockum Group of Howard County, TX.

Remarks—. In his description of “*Buettneria*” (=*Paranaschisma* gen. nov.) *howardensis*, Sawin (1944) did not designate a holotype but based most of his description of the cranial osteology on TMM 31100-30. However, Sawin did not provide any figures of TMM 31100-30 but instead produced composite figures based on specimens TMM 31100-47, TMM 31100-122, and TMM 31220-1 (figs. 3–4: Sawin, 1944). Colbert & Imbrie (1956) considered specimens TMM 31100-30, 31100-42, 31100-122, 31100-124, 31100-161 from Otis Chalk Quarry 3 and TMM 31220-1 from Quarry 4 (see below) to be “cotypes” (=syntypes) of “*B*.” *howardensis* (see table i–a: Sawin, 1944), a framework that has remained in use (Elder, 1978; Schoch & Milner, 2000; Stocker, 2013). Colbert & Imbrie (1956) treated “*Buettneria perfecta*” (=*Tecovaserpeton perfectum*), “*Buettneria*” (=*Buettnererpeton*) *bakeri*, and “*B*.” *howardensis* as synonyms under the subspecies “*Eupelor fraasi jonesi*” based on postcranial characters now known to be variable within populations or simply non-diagnostic to the level of species in the current taxonomic framework (Gee & Kufner, 2022; Lucas et al., 2016; Sulej, 2007). Elder (1978) suggested TMM 31100-30 be used as the lectotype of “*B*.” *howardensis* in an unpublished thesis in keeping with historical trends of discussion of the taxon, and this designation has largely been maintained (Long & Murry, 1995; Stocker, 2013). Hunt (1993) considered “*B*.” *howardensis* to be synonymous with “*B. perfecta*” and did not provide a designation for any of the Howard County specimens. Long & Murry (1995) considered TMM 31100-30 and 31100-42 to be paratypes of “*B*.” *howardensis* but upheld Hunt’s referral of the Howard County specimens to “*B. perfecta*.” Specimens TMM 31100-47 and TMM 31100-161 could not be accessed for this study, but the remaining crania of the type series are scored and figured here. TMM 31220-1 is excluded from the type series here due to ambiguity in stratigraphic correlation between Otis Chalk quarries 3 and 4 and the indeterminate nature of TMM 31220-1 (see below).

**Description**

Otis Chalk Quarry 3—. A brief description of the lectotype cranium (TMM 31100-30) is provided here followed by additional information or variation provided by the syntypes and referred specimens from Otis Chalk Quarry 3. TMM 31100-30 is separated into two main parts: the skull roof and partial palate (Fig. 24) and the basicranium and remainder of the palate (Fig. 25). The skull roof is about 43 cm in length along the midline. The preorbital region of TMM 31100-30 is very broad with relatively small orbits. Other proportional differences in features of the skull roof such as the large external nares of *An. browni sensu stricto* have been noted by previous authors (*e.g*., Kufner & Gee, 2021) but will be treated separately in a study on the morphometrics of the skull roof in North American metoposaurids. Due the conserved arrangement of the bones of the skull roof, only features that are known to vary among metoposaurids are noted here. The septomaxilla was not identified in any of the specimens of *Paranaschisma howardensis*. The lacrimal is incomplete on both the right and left side, but its margins are sufficiently well preserved to show that it forms just under ¼ of the anterolateral orbital margin with its posterior end slotting into the anterior process of the jugal (Figs. 24A, 24B). The lateral part of the anterior process of the jugal extends just anterior to the orbit. The parietal-supratemporal suture is oblique to the midline, but the parietal does not contact the postorbital. The squamosal is approximately pentagonal in outline with a sharper inflection of the squamosal-quadratojugal suture than that seen in *Arganasaurus* and *Buettnererpeton* and more like *Anaschisma* and *Apachesaurus*. The otic notch is deep and opens almost directly posteriorly, framed by a short posterolaterally directed tabular horn. The infraorbital, supraorbital, temporal, jugal, accessory jugal, and partial transverse occipital sulci are present and follow their typical course. The antorbital flexure of the infraorbital sulcus could not be confirmed in TMM 31100-30 due to reconstruction in this area.

Much like the arrangement of the skull roof bones, the elements of the palate are highly conserved. The choanae of TMM 31100-30 are proportionally small compared with most other metoposaurids (Figs. 24C, 24D), but this does not appear to be a widespread condition among other specimens of *P*. *howardensis*. The sutures of the posterior palate encompassing the ectopterygoid, the jugal, and the palatine ramus of the pterygoid are more firmly connected than surrounding elements possibly indicating a higher degree of ossification consistent with the large size of TMM 31100-30. The ectopterygoid on the left side of the palate extends medially to join with the palatine ramus of the pterygoid similar to the condition found in *Apachesaurus gregorii*. The *insula jugalis* is large on the left side of the palate, framing the anterior margin of the subtemporal fenestra and forming over half the width of the palatal ramus. The cultriform process is very wide, only comparable to “*Arganasaurus*” *azerouali* among metoposaurids (Figs. 25A, 25B; fig. 3: Buffa, Jalil & Steyer, 2019). The space between the cultriform process of the parasphenoid and the palatal ramus of the pterygoid that forms the posterior margin of the interpterygoid vacuity appears very narrow, but this is likely due to displacement and reconstruction of the cultriform process where it arises from the basal plate. Ornamentation is not well preserved on the palate, but some faint ridges are present on the basal plate of the parasphenoid.

The squamosal has a narrow contact with the quadrate most evident on the left side of TMM 31100-30 in occipital view (Figs. 25C, 25D). The borders of the paraquadrate foramina indicate a width about twice the maximum diameter of the foramen magnum. The lamellose processes are mostly broken, but the right lamellose process projects slightly into the foramen magnum, though the lamellose processes are much better preserved in other specimens of *P. howardensis* (see below). The dorsal surface of the exoccipital is notched forming the ventral border of the posttemporal fenestra which is entirely enclosed between the descending processes of the tabular and the postparietal which connect to the exoccipital *via* a broad overlap (Figs. 24C, 24D). A small depression of unknown origin is present lateral to the pterygoid depression on the right side of TMM 31100-30 (Figs. 25C, 25D). All of the typical tooth rows and palatal fangs of the metoposaurid palate are present although determining tooth counts from TMM 31100-30 is complicated by extensive reconstruction along the margins of the skull that obscures both teeth and sutures.

The remaining crania of Otis Chalk Quarry 3 are very similar to TMM 31100-30 but reveal details obscured or damaged in the lectotype as well as some variation of the skull roof. TMM 31100-122 served as the basis for much of Sawin’s (1944) initial description likely due to its excellent preservation. Much like TMM 31100-30, the snout is broad and blunt, but the orbits are proportionally larger despite being nearly the same size as the lectotype with a midline length of about 44 cm (Fig. 26). The angle of the parietal-supratemporal suture is more shallow, subparallel to the midline. The Z-shaped flexure of the infraorbital sulcus anterior to the orbit is confirmed here despite poor preservation of the medial margins of each lacrimal.

Details of the surface of the palate are well preserved in TMM 31100-122 including reticulate ornamentation on the basal plate of the parasphenoid that extends onto the cultriform process. The dentition is most completely preserved in TMM 31100-122 along with clear delineations between the tooth-bearing elements of the palate. The labial marginal tooth row spans the premaxilla and the maxilla. The right premaxilla bears 13 tooth bases with space for 14 teeth, and the left premaxilla bears 15 tooth bases. The left maxilla bears at least 44 tooth bases with space for many more comparable to many other stereospondyls with high tooth counts. The lingual marginal tooth row posterior to the palatine fangs is made up of at least 34 teeth that span the palatine and the ectopterygoid. The teeth of the lingual marginal tooth row are larger at their bases than the teeth of the labial marginal row and progressively decrease in size posteriorly. The palatal tooth rows are incomplete in all specimens considered here but are made up teeth with smaller bases than either of the marginal rows. A pair of fang attachments is present on the vomer and on the palatine, but the fangs are entirely missing.

The occiput of TMM 31100-122 is the best preserved of any of the Otis Chalk Quarry 3 specimens (Fig. 27). The most notable departure from other metoposaurids is the medially extensive lamellose process forming an offset projection that nearly meets its counterpart at the midline of the foramen magnum. A similar condition is present in *Metoposaurus algarvensis* (figs. 6a–b: Brusatte et al., 2015), but *M. krasiejowensis* exhibits the more typical broad triangular lamellose process (fig. 16: Sulej, 2007). A slight depression on the left pterygoid is similar to that observed on the right pterygoid of TMM 31100-30 lateral to the pterygoid depression (Figs. 25C, 25D, 26).

TMM 31100-42 has a very well-preserved skull roof with pen markings delineating sutures or approximate suture positions, and the palate and the occiput are heavily reconstructed (Fig. 28). No significant deviations of the suture configuration of the skull roof could be noted. Along the posterior margin of the squamosal, the falciform crest forms a projection that is unique in forming laterally facing notches (Figs. 28A, 28B). A less developed form of this is present in other crania of *Paranaschisma howardensis* (*e.g*., Figs. 24A, 24B, 26A, 26B), but these much smaller notches coincide with the squamosal-quadratojugal suture. The lateral line sulci are clear in TMM 31100-42 clarifying the supraorbital sulcus which does not contact the lacrimal through its antorbital flexure (Figs. 28A, 28B). The posterior flexure of the jugal sulcus turns dramatically toward the midline before abruptly turning toward the lateral side of the skull to contact and traverse the posterior skull roof margin (Figs. 28A, 28B). In palatal view, only the anterior end of the very wide cultriform process is preserved with the basal plate and posterior portion of the cultriform process of the parasphenoid that would bear ornament missing. The margins of the choanae are reconstructed, in part (Figs. 28C, 28D). The palatine-ectopterygoid suture is identified on the right side of the TMM 31100-42 as a slight separation between the elements, and it is ‘kinked’ (*sensu* Gee & Kufner, 2022) deviating significantly from the transverse pen marking on the specimen (Figs. 28C, 28D). None of the dentition-bearing elements preserves the dentition in its entirety. In occipital view, little additional information can be discerned. The occipital pillars (*sensu* Brusatte et al., 2015) and much of the posterior face of the pterygoids are almost entirely reconstructed (Fig. 28E). The postparietals form a ventrally projecting blade as in all other metoposaurids. The sutures underlying the pen markings delimiting the quadrate are entirely unclear, and only the medial and lateral borders of the left paraquadrate foramen can be estimated.

TMM 31100-124 is the smallest nearly complete cranium from Otis Chalk Quarry 3 with a midline length of 35.5 cm. The sutures of the skull roof are well defined, but the palate is damaged and partially reconstructed with either a putty or residual matrix obscuring large portions of the posterior palate and ventral occiput so that the sutural contacts are unclear (Fig. 29). Despite its smaller size, the orbits appear relatively small. Surface details of the antorbital region on the right side of TMM 31100-124 are well preserved demonstrating an elongate lacrimal that contributes to the orbital margin (Figs. 29A, 29B). The sutures of the squamosal are most completely preserved on the right side and are subtriangular in outline without a distinct inflection at the jugal-squamosal-quadratojugal juncture. The supraorbital sulcus on the right side appears to merge with the infraorbital sulcus, and it slightly protrudes onto the lacrimal. An accessory jugal sulcus deviates from the jugal sulcus at about the level of the jugal-quadratojugal suture. A transverse occipital sulcus could not be identified likely due to the incompleteness of the tabulars which are both missing the horn. Little of the sutural morphology of the palate remains intact or identifiable with only general morphology such as the wide, ventrally flat cultriform process being immediately evident (Figs. 29C, 29D). The right pterygoid-ectopterygoid suture is visible indicating a posteromedial extension of the ectopterygoid to form part of the palatal ramus along with the pterygoid. The remaining details of the palate are consistent with metoposaurids in general.

TMM 31100-19 is larger than the preceding crania from Otis Chalk Quarry 3, but due to missing premaxillae, the midline length could not be determined but is ~41 cm without the premaxilla. TMM 31100-19 was excluded from the syntype series of “*Buettneria*” (=*Paranaschisma*) *howardensis* and here is referred on the basis of a very wide cultriform process of the parasphenoid, extensive ornamentation of the basal plate of the parasphenoid extending onto the cultriform process, and a posteromedial extension of the ectopterygoid forming part of the palatal ramus with the pterygoid (Fig. 30). The skull roof appears to have separated along many of the sutures, especially on the left side, resulting in infilling of plaster to stabilize TMM 31100-19 (Figs. 30A, 30B). The sutures of the skull roof are consistent with other specimens from Otis Chalk Quarry 3 with no notable variation. The lateral line sulci of the skull roof are mostly conserved with the only notable difference being the lack of a transverse occipital sulcus. The palate is similarly conserved, and the ventral surface of the basal plate and cultriform process of the parasphenoid is well preserved (Figs. 30C, 30D). Reticulate ornamentation on the basal plate radiates anteriorly onto the cultriform process as a set of elongate ridges. A row of at least six teeth is present posterior to the right choana.

TMM 31100-431 is a partial skull roof with an associated stapes from Otis Chalk Quarry 3 (Fig. 31). Most of the details of the dorsal surface of the skull roof are consistent with other specimens of *Paranaschisma howardensis*. Like the lectotype and a referred specimen of *P. howardensis* (Figs. 24, 30), TMM 31100-431 has an angled parietal-supratemporal suture (Figs. 31A, 31B). On the right side of TMM 31100-431, the supraorbital sulcus slightly overlaps onto the lacrimal, and on the left side, the supraorbital sulcus appears to be excluded from contacting the lacrimal by the infraorbital sulcus, but there is significant damage in this area. The transverse occipital sulcus extends across the entire posterior margin of the tabular and probably partially onto the postparietal. In ventral view, little of the palate remains and the ventral expression of the skull roof sutures approximately mirrors that of the dorsal surface (Figs. 31C, 31D). The lateral margin of the left choana is preserved and suggests a much larger opening than that seen in some other specimens of *P. howardensis* (*e.g*., Figs. 24, 26, 29). The left premaxilla is preserved in nearly its entirety with 11 tooth bases and space for as many as 16 (Figs. 31C, 31D). A left stapes numbered TMM 31100-431 was previously described by Sawin (Fig. 31E; fig. 5: 1945). The dorsal and ventral proximal heads are well defined with a concave notch between them, and no foramina were observed on the proximal end of the stapes. The dorsal proximal head is rugose as in other metoposaurids for a presumed cartilaginous cap. The transverse and anterior crests, extending distally from dorsal and ventral proximal heads, respectively, are not well defined on the proximal end. A groove extends along the length of the posteroventral surface of the stylus columella with the most well defined portion of the anterior crest framing it, but it is unclear if this groove is present due to crushing as has been described in some specimens of *Mastodonsaurus giganteus* (Schoch, 2000).

UCMP 66991 is the largest cranium from Otis Chalk Quarry 3 accessed in this study with a midline length of ~49.6 cm. The dorsal surface of the skull roof is well preserved with minimal reconstruction, and the sutural pattern is consistent with that observed in the type series of *Paranaschisma howardensis* (Figs. 32A, 32B). Most of the sutures were split and separated by a thin layer of matrix, but some of the matrix-filled features are fractures. Like some other specimens of *P. howardensis* (Figs. 24, 26, 28, 30), the supraorbital sulcus does not reach the lacrimal on the left side; the right side is too damaged to determine. There is no evidence of a transverse occipital sulcus on the tabular with exclusively reticulate ornamentation extending onto the tabular horn. The palate is poorly preserved with extensive fracturing and significant missing portions (Figs. 32C, 32D). The cultriform process of the parasphenoid while broken is clearly wider at its minimum width that the palatal ramus of the pterygoid. The *insula jugalis* is visible on the right side of UCMP 66991, and it extends well anteriorly. Additional features of the lateral margins of the palate are difficult to discern due to damage. The dentition is incompletely preserved throughout but does not differ significantly from other metoposaurids.

Cerita de la Cruz Creek—. UMMP 21326 is a partial cranium with the dorsal surface entirely embedded in plaster (Fig. 33). The ventral surface of the skull roof and the palate are well-preserved and prepared allowing for a general understanding of the arrangement of the skull roof bones and detailed understanding of the preserved parts of the palate, especially the dentition. The arrangement of the palate and skull roof bones in ventral view is similar to other examples of *Paranaschisma howardensis*, and only differences are noted here. The shape of the rostrum is not as blunt and rounded as in other specimens of *P. howardensis*. The premaxilla bears a small depression just anterior to the anterior palatal fenestra which is interpreted here as a mark left by the dentary fang (Figs. 33A, 33B). A ‘kinked’ palatine-ectopterygoid suture could be readily identified in UMMP 21326 due to the excellent preservation. The orbitotemporal crest on the ventral surface of the parietal is ventrally extended as a flange with an irregular ventral margin flanking the pineal foramen. Posterior to this flange, the orbitotemporal crest rapidly reduces in height. This flange likely either contacted one or more cartilaginous elements of the neurocranium such as the sphenethmoid or could be a partially ossified neurocranial element, but fractures at its base could not be confidently identified as sutures. The *insula jugalis* of UMMP 21326 is extensive occupying more than half the width of the palatal ramus like in other examples of *P. howardensis* (Figs. 24, 26, 30). A portion of the vomer and the parasphenoid is broken and could be removed to reveal the anterior extent of the frontal and relative length of the nasal (Figs. 33C, 33D).

Many of the teeth of UMMP 21326 are still in place and supported by plaster or matrix, but this does block the view of some teeth in ventral view (Figs. 33C, 33D), so full counts are provided here. The marginal tooth rows are almost completely preserved on the premaxillae and partially preserved on the maxillae due to damage. There are nine teeth and three tooth bases on the right premaxilla, and there are at least nine teeth on the left premaxilla with space for more. There are at least 60 teeth on the right maxilla with space for more There are five teeth in the transverse vomerine tooth row on the right vomer and four teeth on the left with space for more on both elements. Based on other metoposaurid specimens, this would suggest that approximately every other tooth base of the transverse vomerine tooth row bore a tooth at the time of deposition of UMMP 21326. There are 18 teeth in the left parachoanal tooth row, and a fracture through the tooth row on the right vomer precludes a full count on the left side, but at least 13 teeth are present. A pair of teeth is present just posterior to the choana on the right and the left palatine. The palatal row of the left palatine has six teeth, and the left palatine has space for as many as seven teeth with two smaller teeth just posterior to the fangs. A total of 17 teeth could be identified on the left ectopterygoid with space for 5–8 more, and 16 teeth were identified on the right ectopterygoid with space for a similar number of teeth as the left side. The diameters of the palatal teeth are greater than the adjacent teeth of the marginal row. The typical paired fangs of the vomer and the palatine are present with a fang present in the anterior positions of the vomerine fangs and in the anterior position on the right palatine and the posterior position on the left palatine. All the preserved teeth form simple cones that are recurved in the anterior positions and more or less straight or slightly lingually inclined further posteriorly.

*Tecovaserpeton* gen. nov.

Diagnosis—. As for species by monotypy.

Etymology—. The name is derived from the Tecovas Formation from which the holotype was collected, and numerous referred specimens are known, combined with “-*herpeton*” a neuter Greek suffix commonly applied to “reptiles” and “amphibians.” The word “Tecovas” is a corruption of the Spanish “techados” meaning “roofs” or “tents” referring to the common encampments along the nominal creek and nearby springs in Potter County, TX (Anonymous, 1995).

*Tecovaserpeton perfectum* Case, 1922

Synonyms—. *Buettneria perfecta* Case, 1922

*Buettneria calgariensis* Green, 1954

*Eupelor fraasi jonesi* (in part) Colbert & Imbrie, 1956

*Metoposaurus fraasi jonesi* (in part) RoyChowdhury, 1965

*Metoposaurus fraasi* (in part) Murry, 1986

*Koskinonodon perfectus* (in part) Mueller, 2007

*Koskinonodon perfectum* (in part) Spielmann & Lucas, 2012

*Koskinonodon perfecta* (in part) Brusatte et al., 2015

*Anaschisma browni* (in part) Gee, Parker & Marsh, 2020

Figures 34–45

**Figure 34:**
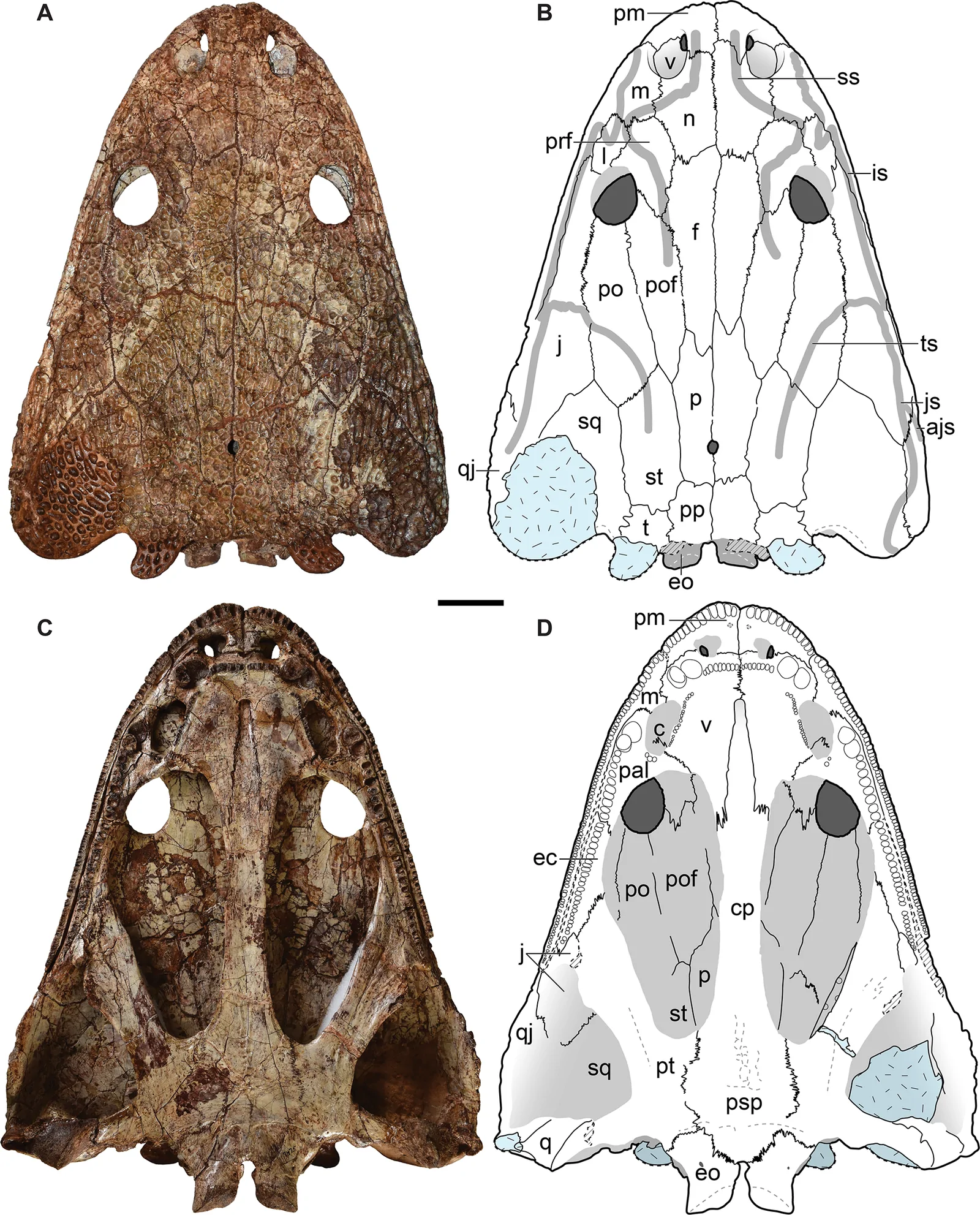
Holotype cranium of *Tecovaserpeton perfectum*, UMMP 7475, from the Sand Creek breaks in Crosby County, TX. (A) Photograph in dorsal view; (B) interpretive drawing of the same; (C) photograph in ventral view; (D) interpretive drawing of the same. Blue hatching represents reconstruction; diagonal lines represent broken surfaces; fine dashed gray lines represent topographic details like ridges; open circles represent a block of supporting foam. Abbreviations: ajs, accessory jugal sulcus; c, choana (internal naris); cp, cultriform process; ec, ectopterygoid; eo, exoccipital; f, frontal; is, infraorbital sulcus; j, jugal; js, jugal sulcus; l, lacrimal; m, maxilla; n, nasal; p, parietal; pal, palatine; pm, premaxilla; po, postorbital; pof, postfrontal; pp, postparietal; prf, prefrontal; psp, parasphenoid; pt, pterygoid; q, quadrate; qj, quadratojugal; sq, squamosal; ss, supraorbital sulcus; st, supratemporal; t, tabular; ts, temporal sulcus; v, vomer. Scale bar equals 5 cm.

**Figure 35:**
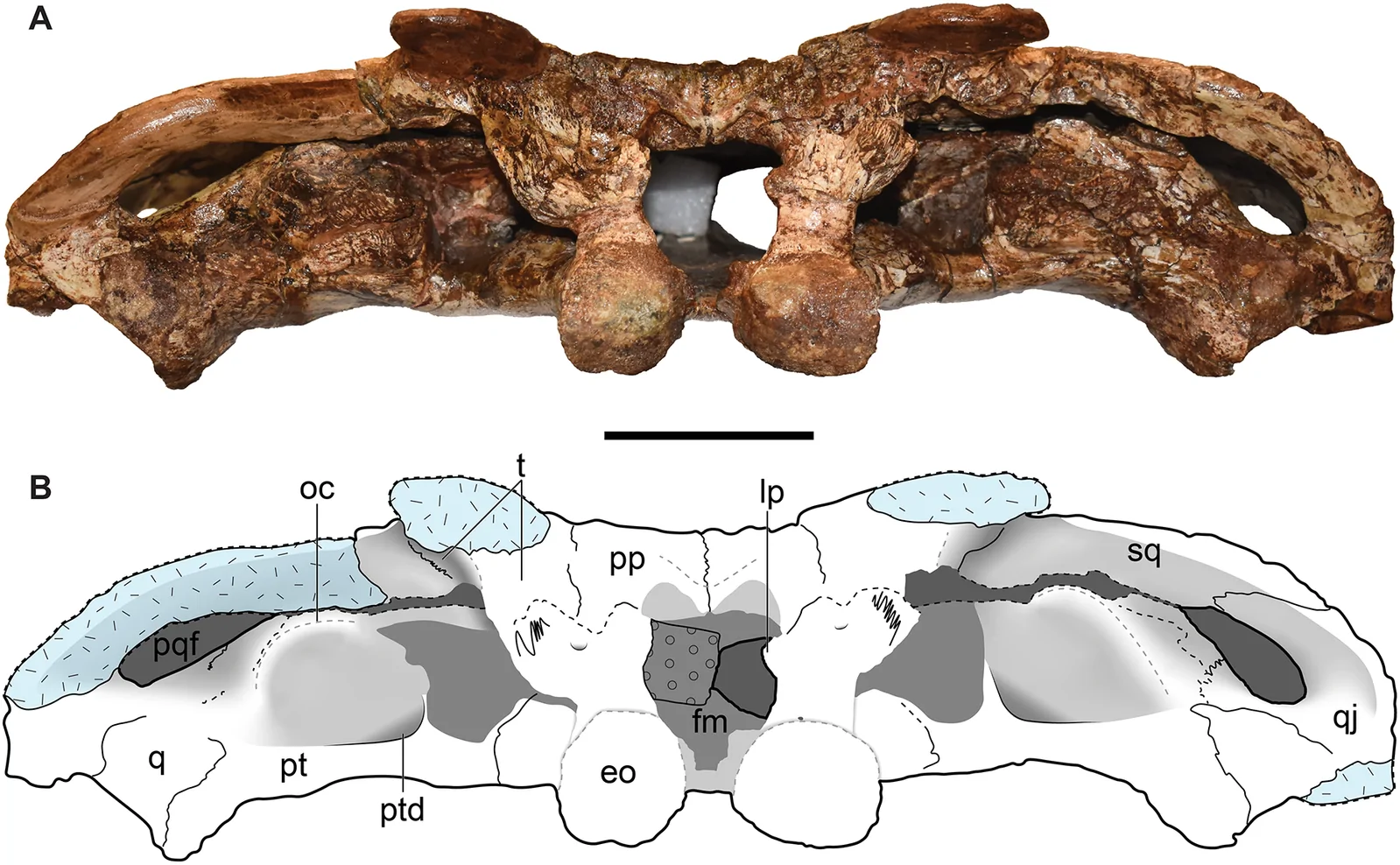
Holotype cranium of *Tecovaserpeton perfectum*, UMMP 7475, from the Sand Creek breaks in Crosby County, TX in occipital view. (A) Photograph; (B) interpretive drawing. Blue hatching represents reconstruction; fine dashed gray lines represent topographic details like ridges; open circles represent a block of supporting foam. Abbreviations: eo, exoccipital; fm, foramen magnum; lp, lamellose process; oc, oblique crest of the pterygoid; pp, postparietal; pqf, paraquadrate foramen; pt, pterygoid; ptd, pterygoid depression; q, quadrate; qj, quadratojugal; sq, squamosal; t, tabular. Scale bar equals 5 cm.

**Figure 36:**
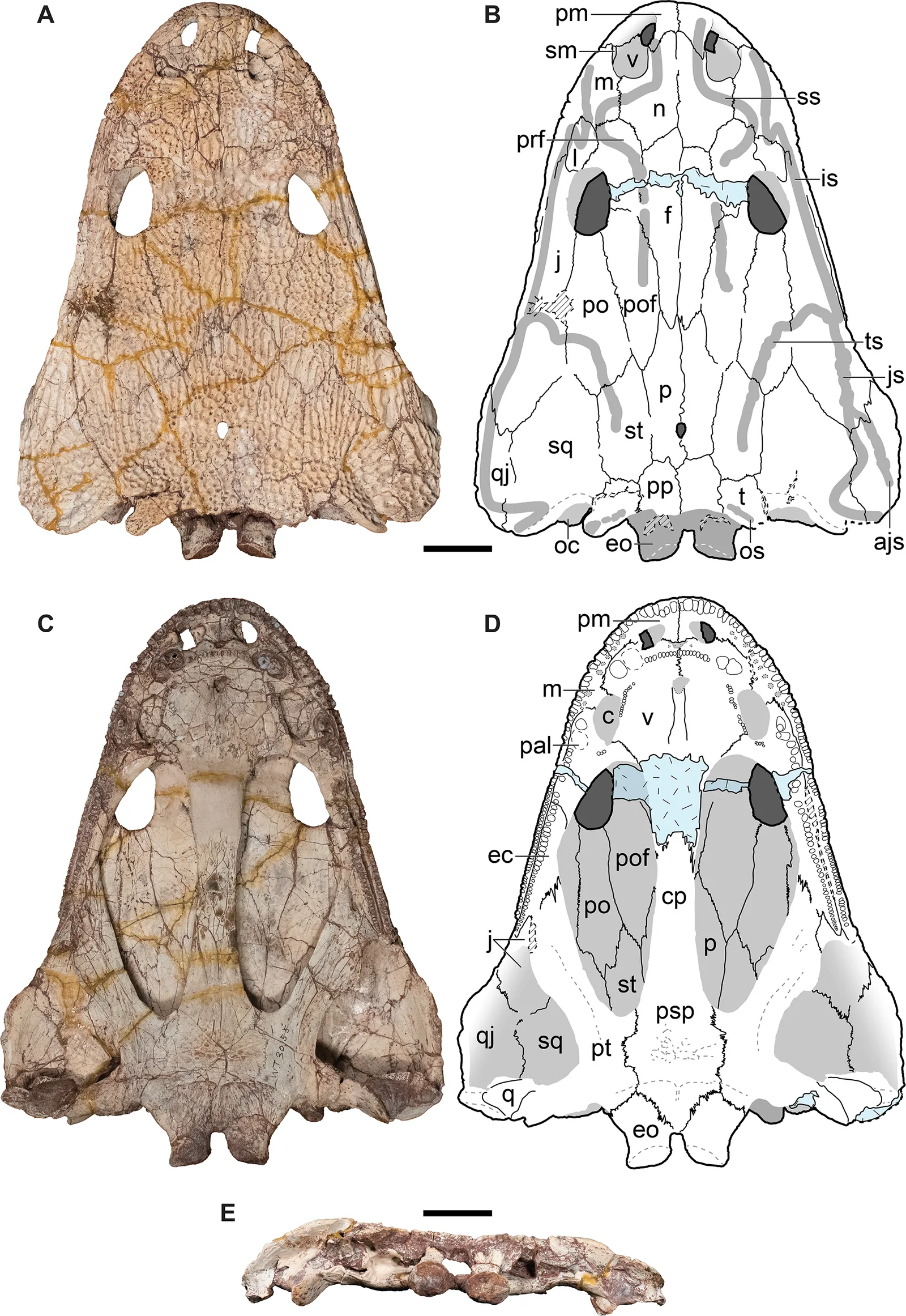
Referred cranium of *Tecovaserpeton perfectum*, WT 3055, from the Rotten Hill bonebed in Potter County, TX. (A) Photograph in dorsal view; (B) interpretive drawing of the same; (C) photograph in ventral view; (D) interpretive drawing of the same; (E) photograph in occipital view. Blue hatching represents reconstruction; diagonal lines represent broken surfaces; fine dashed gray lines represent topographic details like ridges; dashed outlines of the palatal fangs indicate a tooth attachment site without a tooth present; dashed outlines infilled with gray indicate depressions made by the dentary tooth row. Abbreviations: ajs, accessory jugal sulcus; c, choana (internal naris); cp, cultriform process; ec, ectopterygoid; eo, exoccipital; f, frontal; is, infraorbital sulcus; j, jugal; js, jugal sulcus; l, lacrimal; m, maxilla; n, nasal; oc, oblique crest of the pterygoid; os, transverse occipital sulcus; p, parietal; pal, palatine; pm, premaxilla; po, postorbital; pof, postfrontal; pp, postparietal; prf, prefrontal; psp, parasphenoid; pt, pterygoid; q, quadrate; qj, quadratojugal; sm, septomaxilla; sq, squamosal; ss, supraorbital sulcus; st, supratemporal; t, tabular; ts, temporal sulcus; v, vomer. Upper scale bar for (A–D); lower scale bar for (E). Scale bars equal 5 cm.

**Figure 37:**
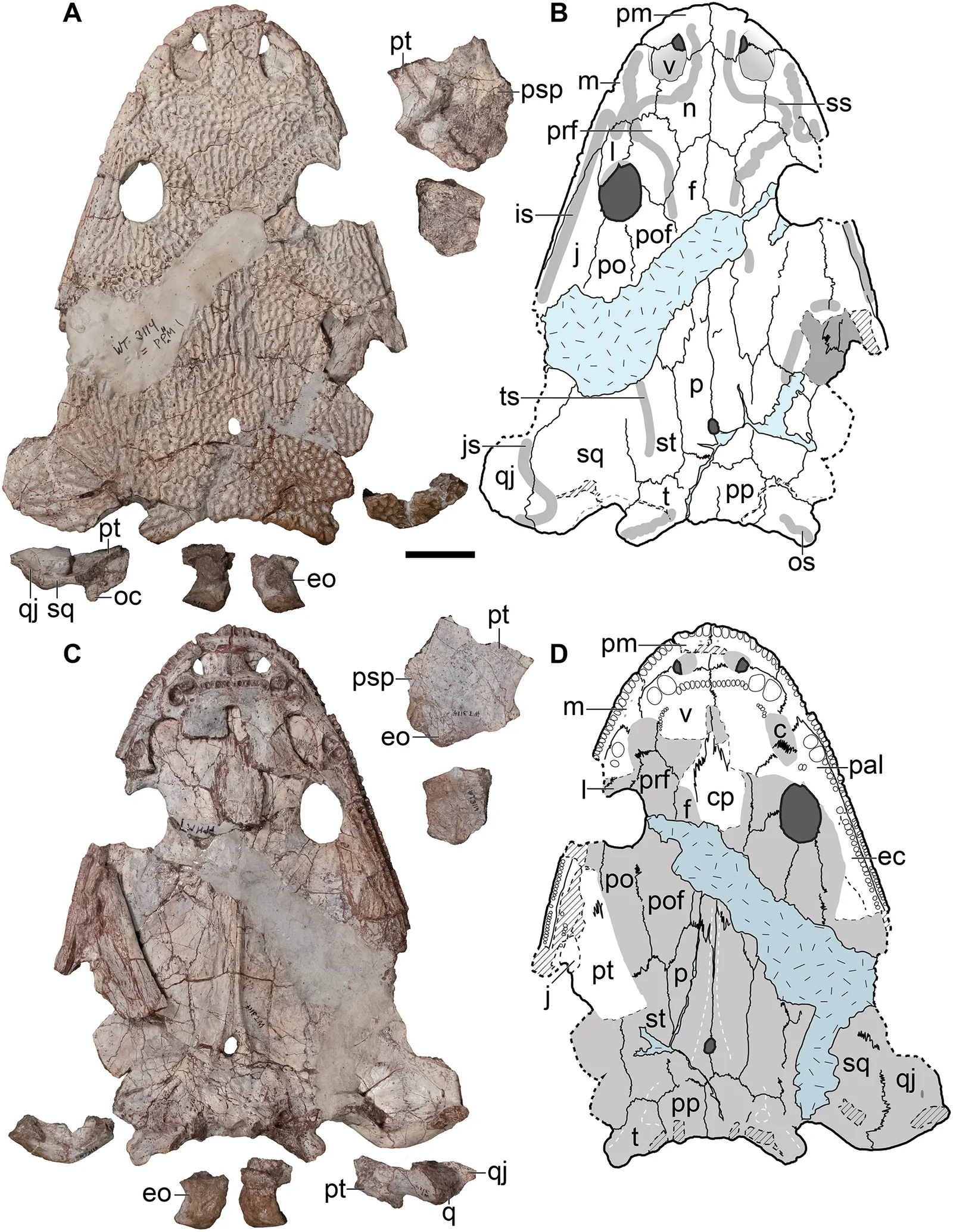
Referred skull roof and associated fragments of the palate and occiput of *Tecovaserpeton perfectum*, WT 3114 (PPHM 1), from the Rotten Hill bonebed in Potter County, TX. (A) Photograph in dorsal view; (B) interpretive drawing of the skull roof in dorsal view; (C) photograph in ventral view; (D) interpretive drawing of the skull roof in ventral view. Blue hatching represents reconstruction and regions covered with a thin layer of plaster; fine dashed gray lines represent topographic details like ridges; diagonal lines represent broken surfaces. Abbreviations: c, choana (internal naris); cp, cultriform process; ec, ectopterygoid; eo, exoccipital; f, frontal; is, infraorbital sulcus; j, jugal; js, jugal sulcus; l, lacrimal; m, maxilla; n, nasal; oc, oblique crest of the pterygoid; os, transverse occipital sulcus; p, parietal; pal, palatine; pm, premaxilla; po, postorbital; pof, postfrontal; pp, postparietal; prf, prefrontal; psp, parasphenoid; pt, pterygoid; q, quadrate; qj, quadratojugal; sq, squamosal; ss, supraorbital sulcus; st, supratemporal; t, tabular; ts, temporal sulcus; v, vomer. Scale bar equals 5 cm.

**Figure 38:**
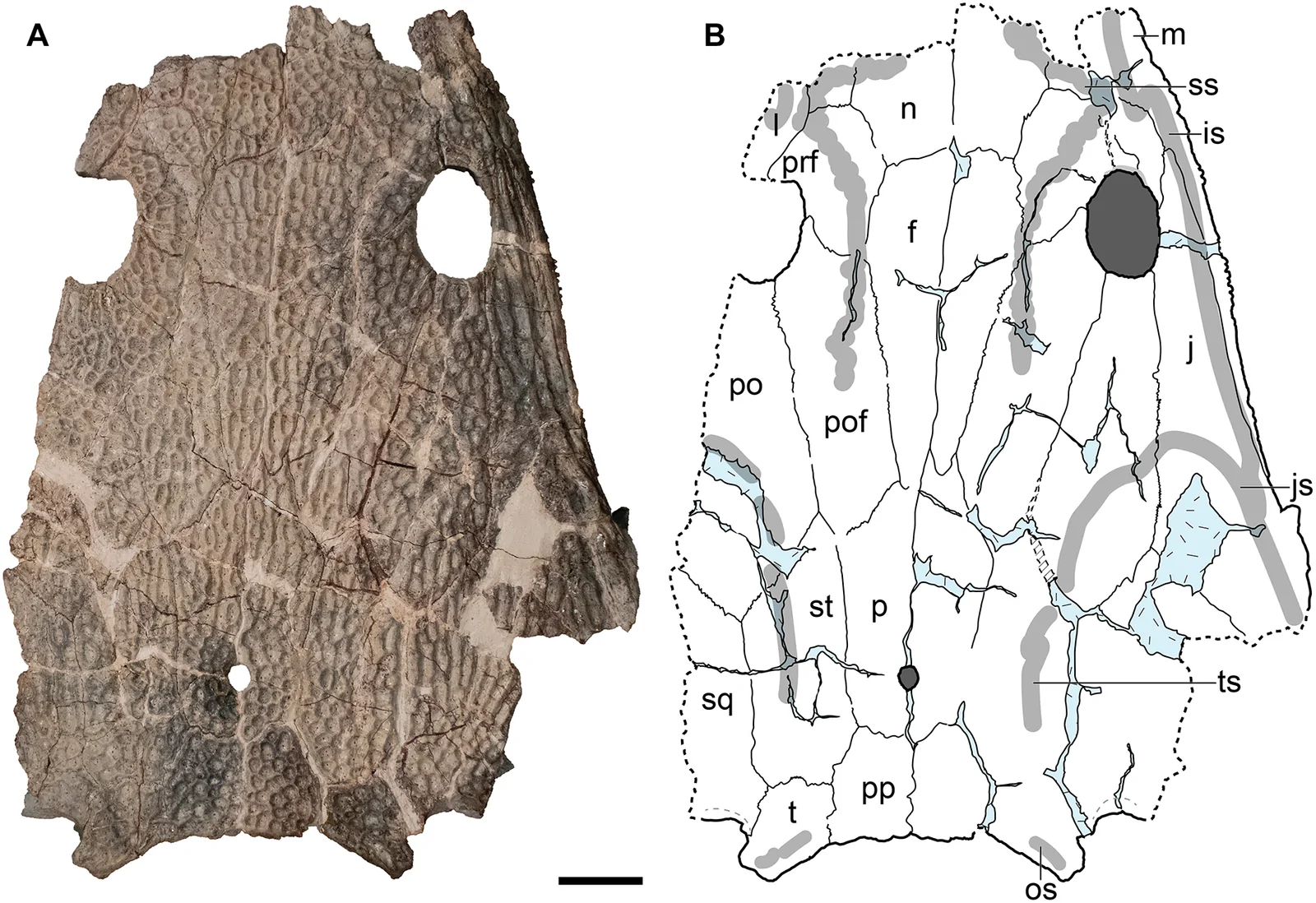
Referred skull roof of *Tecovaserpeton perfectum*, WT 3003, from the Rotten Hill bonebed in Potter County, TX in dorsal view. (A) Photograph; (B) interpretive drawing. Blue hatching represents reconstruction and regions covered with a thin layer of plaster; fine dashed gray lines represent topographic details like ridges. Abbreviations: f, frontal; is, infraorbital sulcus; j, jugal; js, jugal sulcus; l, lacrimal; m, maxilla; n, nasal; os, transverse occipital sulcus; p, parietal; po, postorbital; pof, postfrontal; pp, postparietal; prf, prefrontal; sq, squamosal; ss, supraorbital sulcus; st, supratemporal; t, tabular; ts, temporal sulcus. Scale bar equals 5 cm.

**Figure 39:**
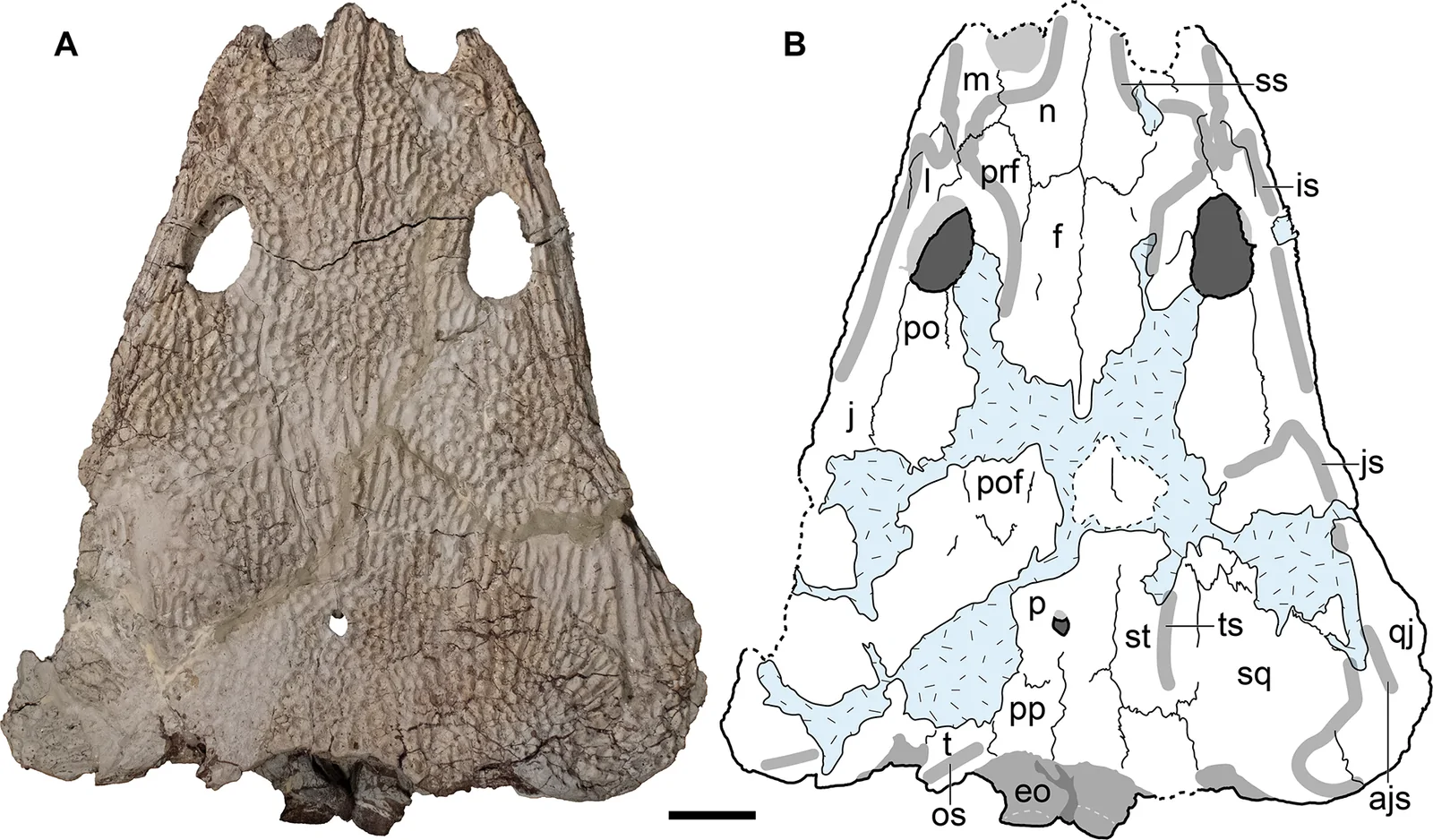
Referred cranium of *Tecovaserpeton perfectum*, WT 3011-1, from the Rotten Hill bonebed in Potter County, TX in dorsal view. (A) Photograph; (B) interpretive drawing. Blue hatching represents reconstruction and regions covered with a thin layer of plaster; fine dashed gray lines represent topographic details like ridges. Abbreviations: ajs, accessory jugal sulcus; eo, exoccipital; f, frontal; is, infraorbital sulcus; j, jugal; js, jugal sulcus; l, lacrimal; m, maxilla; n, nasal; os, transverse occipital sulcus; p, parietal; po, postorbital; pof, postfrontal; pp, postparietal; prf, prefrontal; qj, quadratojugal; sq, squamosal; ss, supraorbital sulcus; st, supratemporal; t, tabular; ts, temporal sulcus. Scale bar equals 5 cm.

**Figure 40:**
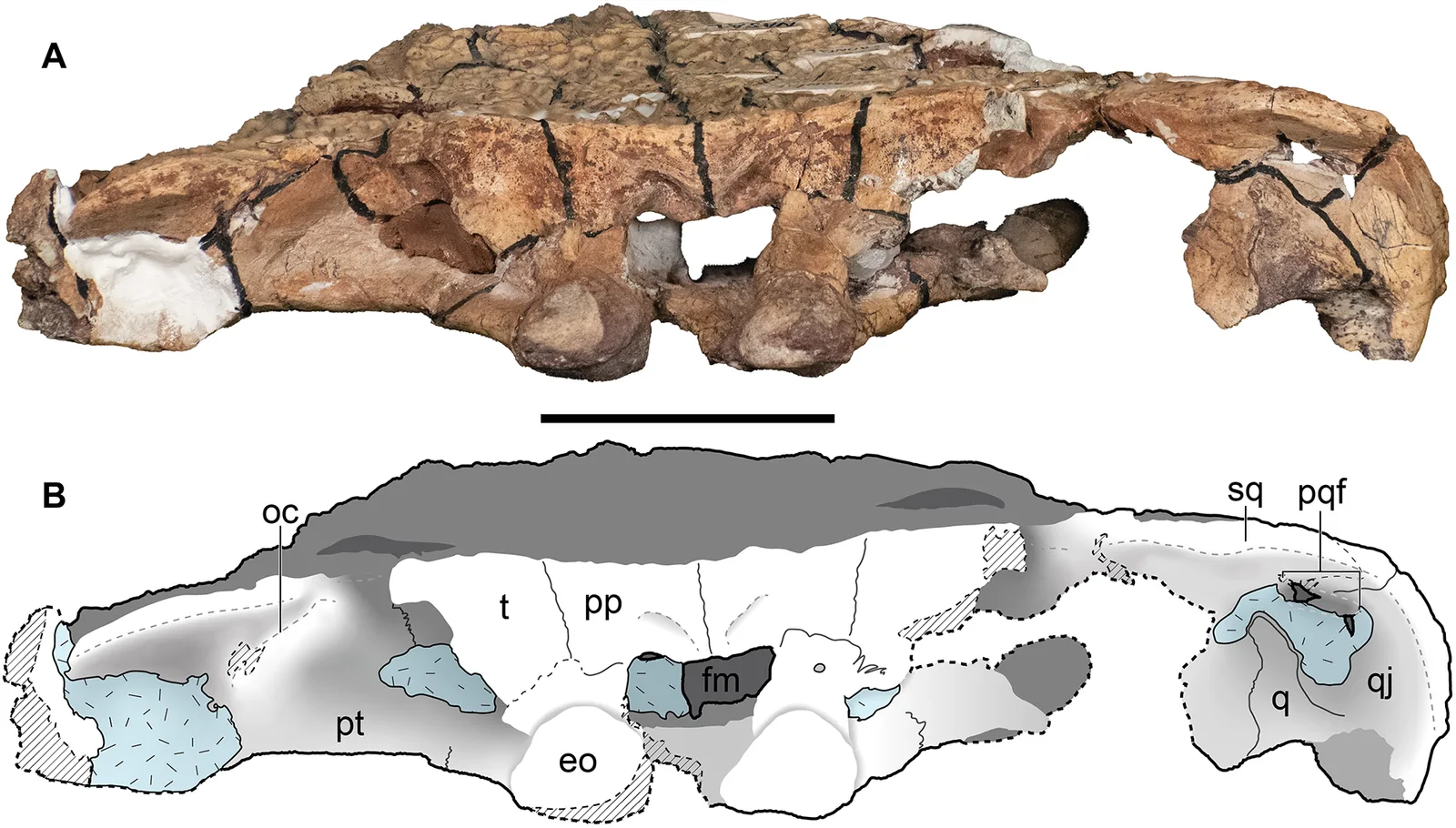
Referred cranium of *Tecovaserpeton perfectum*, WT 3011, from the Rotten Hill bonebed in Potter County, TX in occipital view. (A) Photograph; (B) interpretive drawing. Blue hatching represents reconstruction; fine dashed gray lines represent topographic details like ridges; diagonal lines represent broken surfaces. Abbreviations: eo, exoccipital; fm, foramen magnum; oc, oblique crest of the pterygoid; pp, postparietal; pqf, paraquadrate foramen; pt, pterygoid; q, quadrate; qj, quadratojugal; sq, squamosal; t, tabular. Scale bar equals 5 cm.

**Figure 41:**
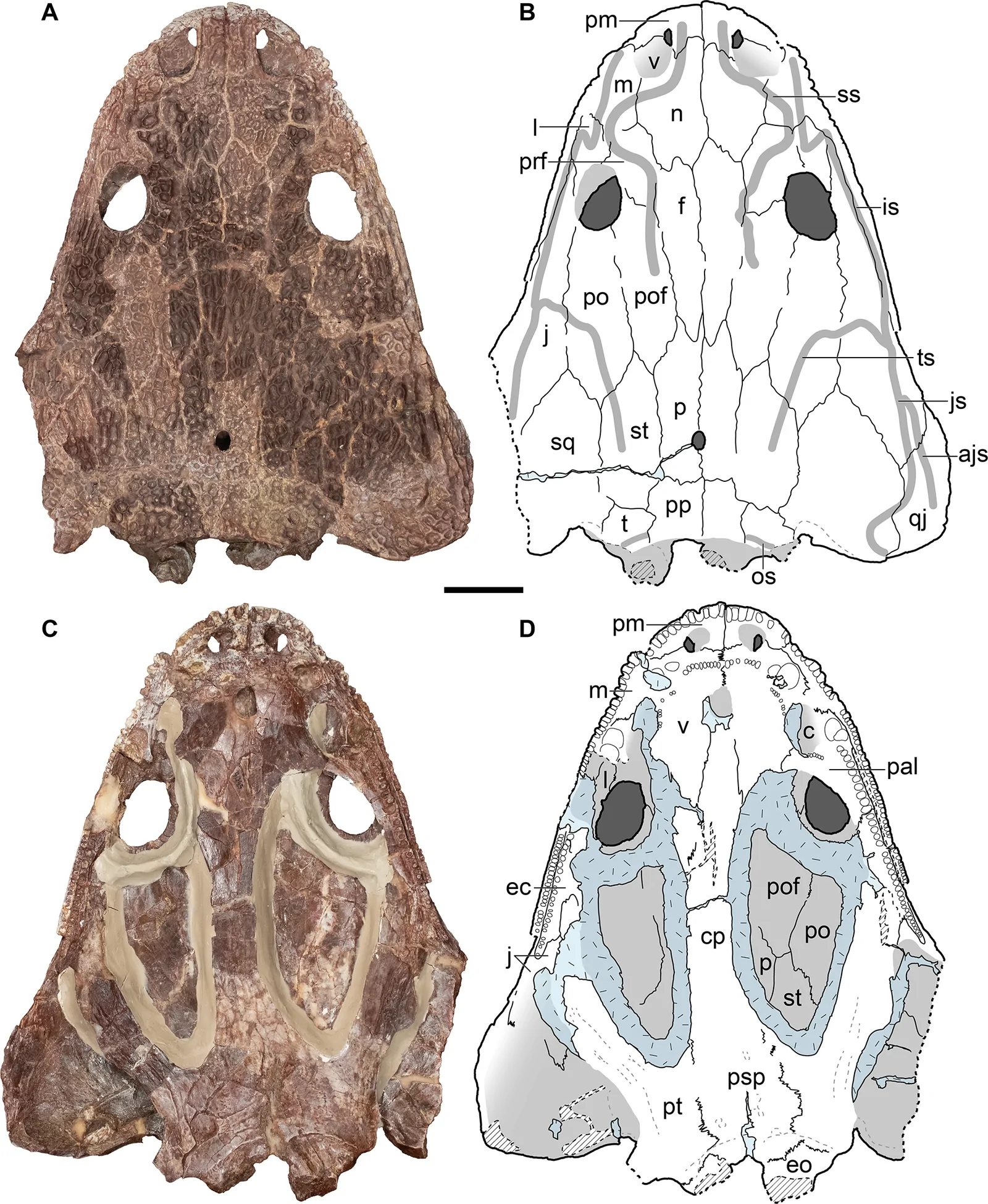
Partial cranium referred to *Tecovaserpeton perfectum*, TTU-P09045, from an unknown locality in the Dockum Group of TX. (A) Photograph in dorsal view; (B) interpretive drawing of the same; (C) photograph in ventral view; (D) interpretive drawing of the same. Blue hatching represents reconstruction; diagonal lines represent broken surfaces; fine dashed gray lines represent topographic details like ridges. Abbreviations: ajs, accessory jugal sulcus; c, choana (internal naris); cp, cultriform process; ec, ectopterygoid; eo, exoccipital; f, frontal; is, infraorbital sulcus; j, jugal; js, jugal sulcus; l, lacrimal; m, maxilla; n, nasal; os, transverse occipital sulcus; p, parietal; pal, palatine; pm, premaxilla; po, postorbital; pof, postfrontal; pp, postparietal; prf, prefrontal; psp, parasphenoid; pt, pterygoid; qj, quadratojugal; sq, squamosal; ss, supraorbital sulcus; st, supratemporal; t, tabular; ts, temporal sulcus; v, vomer. Scale bars equal 5 cm.

**Figure 42:**
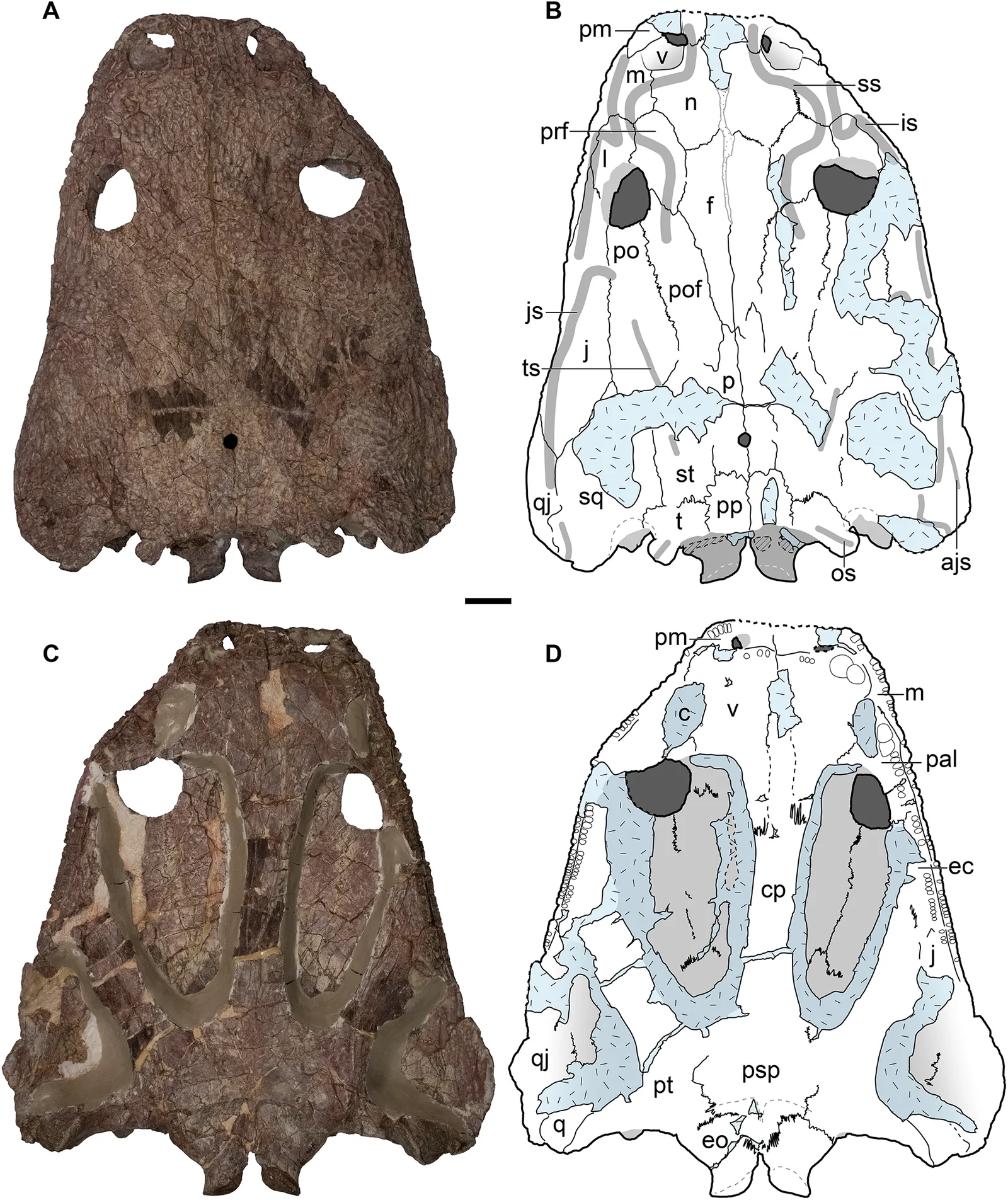
Holotype cranium of “*Buettneria calgariensis*” (=*Tecovaserpeton perfectum*), TTU-P09044, from MOTT VPL 0696 in Crosby County, TX. (A) Photograph in dorsal view; (B) interpretive drawing of the same; (C) photograph in ventral view; (D) interpretive drawing of the same. Blue hatching represents reconstruction or supporting putty; fine dashed gray lines represent topographic details like ridges; diagonal lines represent broken surfaces. Abbreviations: ajs, accessory jugal sulcus; c, choana (internal naris); cp, cultriform process; ec, ectopterygoid; eo, exoccipital; f, frontal; is, infraorbital sulcus; j, jugal; js, jugal sulcus; l, lacrimal; m, maxilla; n, nasal; os, transverse occipital sulcus; p, parietal; pal, palatine; pm, premaxilla; po, postorbital; pof, postfrontal; pp, postparietal; prf, prefrontal; psp, parasphenoid; pt, pterygoid; q, quadrate; qj, quadratojugal; sq, squamosal; ss, supraorbital sulcus; st, supratemporal; t, tabular; ts, temporal sulcus; v, vomer. Scale bar equals 5 cm.

**Figure 43:**
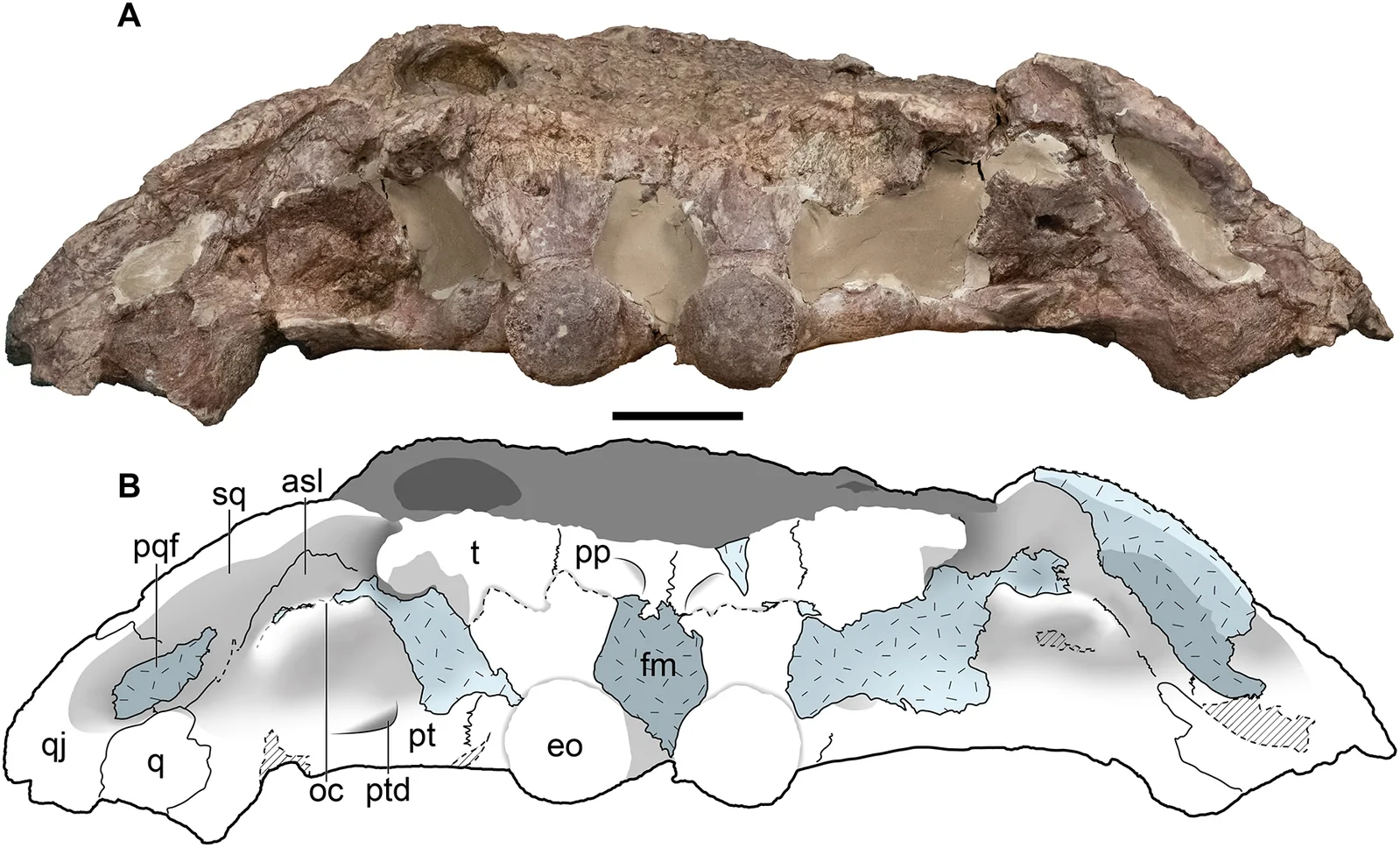
Holotype cranium of “*Buettneria calgariensis*” (=*Tecovaserpeton perfectum*), TTU-P09044, from MOTT VPL 0696 in Crosby County, TX. in occipital view. (A) Photograph; (B) interpretive drawing. Blue hatching represents reconstruction or supporting putty; fine dashed gray lines represent topographic details like ridges; diagonal lines represent broken surfaces. Abbreviations: asl, ascending lamina of the pterygoid; eo, exoccipital; fm, foramen magnum; oc, oblique crest of the pterygoid; pp, postparietal; pqf, paraquadrate foramen; pt, pterygoid; ptd, pterygoid depression; q, quadrate; qj, quadratojugal; sq, squamosal; t, tabular. Scale bar equals 5 cm.

**Figure 44:**
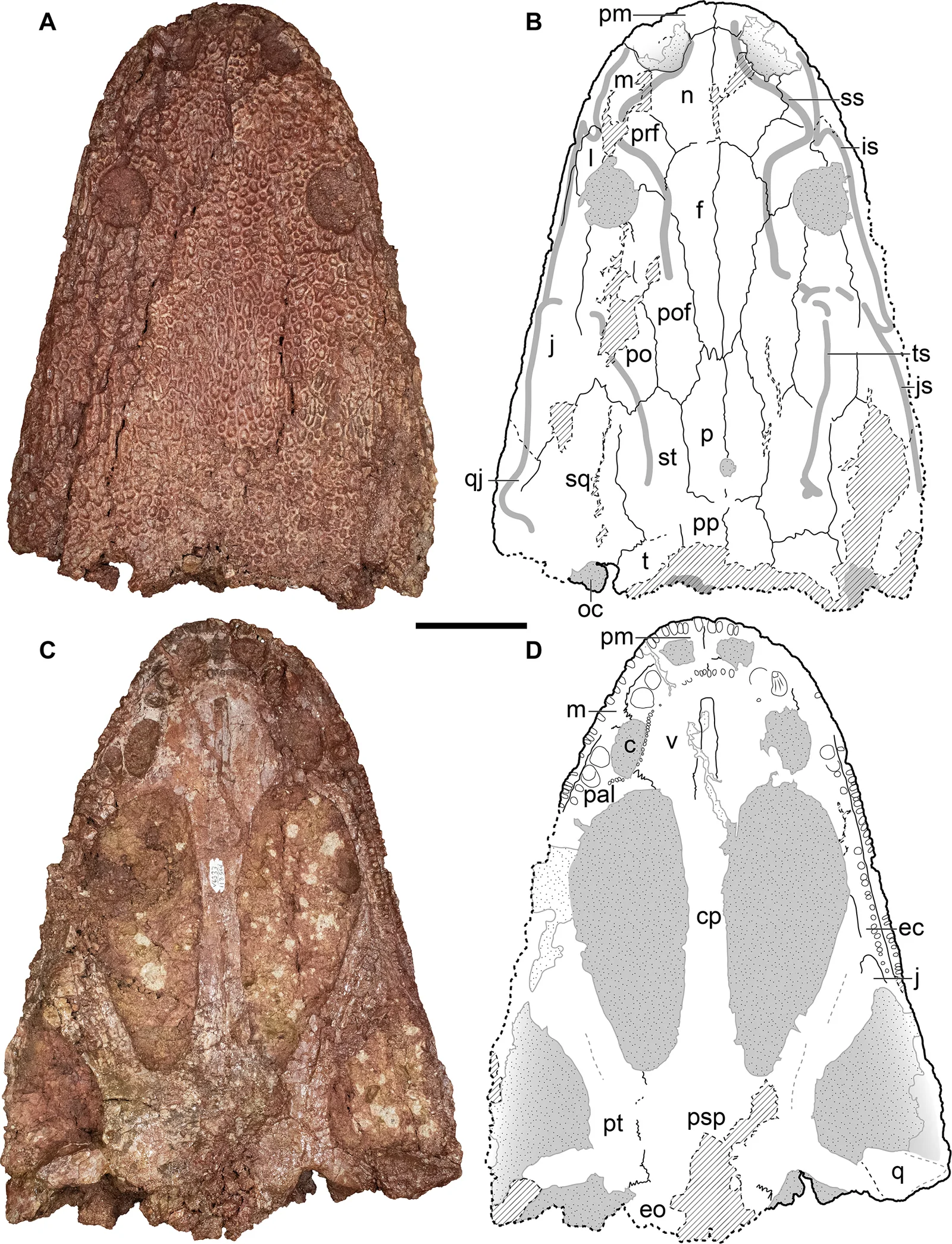
Cranium of a metoposaurid cf. *Tecovaserpeton perfectum*, UCMP 113554, from UCMP V6333 (Davidson Creek) in Crosby County, TX. (A) Photograph in dorsal view; (B) interpretive drawing of the same; (C) photograph in ventral view; (D) interpretive drawing of the same. Stippling represents residual matrix; diagonal lines represent broken surfaces; fine dashed gray lines represent topographic details like ridges. Abbreviations: c, choana (internal naris); cp, cultriform process; ec, ectopterygoid; eo, exoccipital; f, frontal; is, infraorbital sulcus; j, jugal; js, jugal sulcus; l, lacrimal; m, maxilla; n, nasal; oc, oblique crest of the pterygoid; p, parietal; pal, palatine; pm, premaxilla; po, postorbital; pof, postfrontal; pp, postparietal; prf, prefrontal; psp, parasphenoid; pt, pterygoid; q, quadrate; qj, quadratojugal; sq, squamosal; ss, supraorbital sulcus; st, supratemporal; t, tabular; ts, temporal sulcus; v, vomer. Scale bar equals 5 cm.

**Figure 45:**
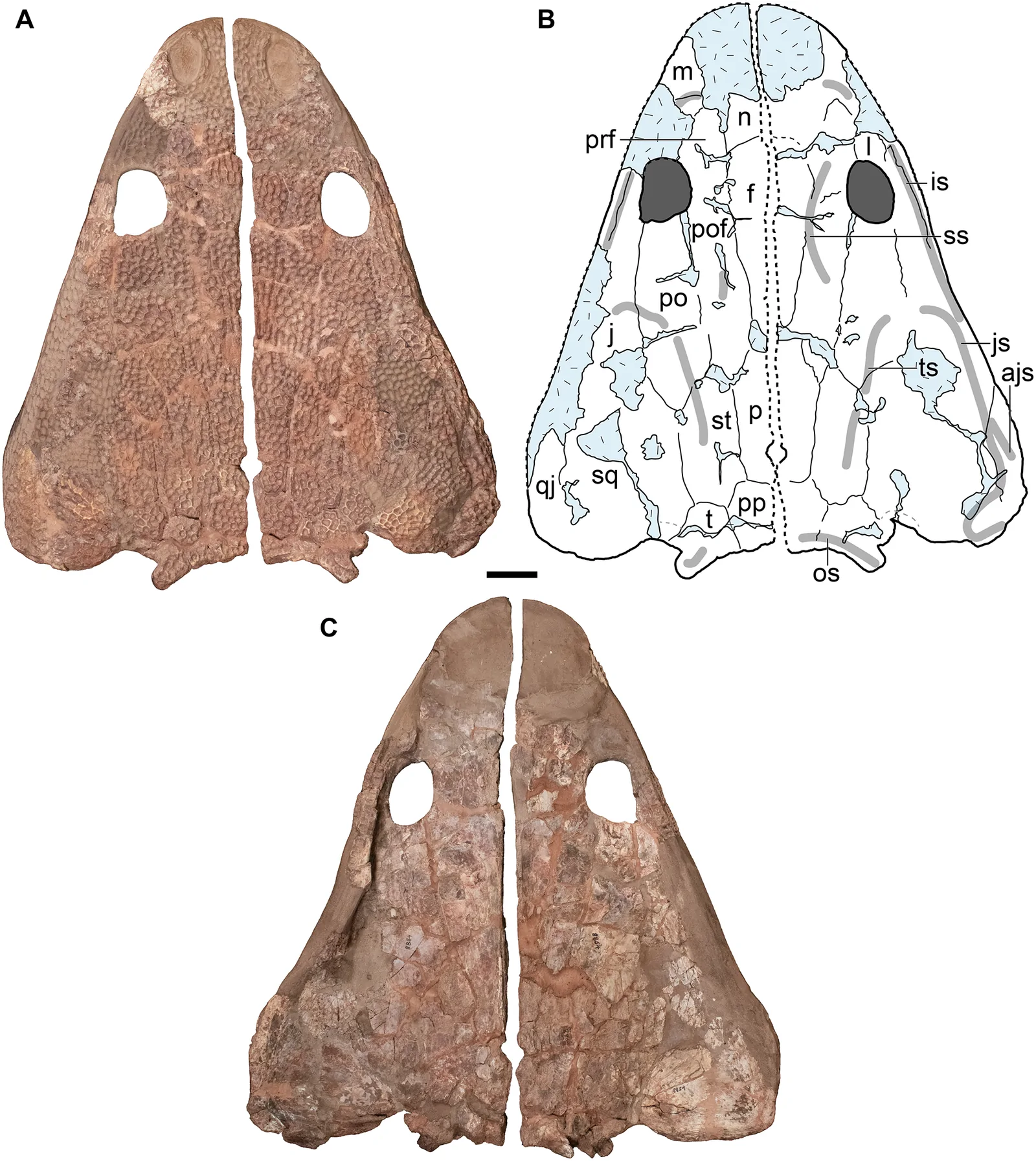
Skull roof of a metoposaurid cf. *Tecovaserpeton perfectum*, UMMP 8854, from Head of Holmes Creek (sometimes spelled Home Creek) in Crosby County, TX. (A) Photograph in dorsal view; (B) interpretive drawing of the same; (C) photograph in ventral view. Blue hatching represents reconstruction; fine dashed gray lines represent topographic details like ridges. Abbreviations: ajs, accessory jugal sulcus; f, frontal; is, infraorbital sulcus; j, jugal; js, jugal sulcus; l, lacrimal; m, maxilla; n, nasal; os, transverse occipital sulcus; p, parietal; po, postorbital; pof, postfrontal; pp, postparietal; prf, prefrontal; qj, quadratojugal; sq, squamosal; ss, supraorbital sulcus; st, supratemporal; t, tabular; ts, temporal sulcus. Scale bar equals 5 cm.

Holotype—. UMMP 7475, an essentially complete skull.

Paratype—. UMMP 7469, a complete left hemimandible.

Etymology—. The specific epithet is amended to the neuter form of the Latin “*perfectum*” corresponding to the neuter form of the Ancient Greek “*herpeton*” used in “*Tecovaserpeton*.”

Type locality and horizon—. Sand Creek breaks south of Cedar Mountain in the Tecovas Formation of Crosby County, TX.

Referred material—. All metoposaurid material from the Rotten Hill locality previously referred to “*Koskinonodon perfectum*” (see Lucas et al., 2016); TTU-P09044, cranium; TTU-P09045, cranium; UCMP 113554, nearly complete cranium; UMMP 8854, skull roof; UCMP 27103, nearly complete skull roof from Crocodile Hill (PF 124) in the lower Petrified Forest Member of the Chinle Formation (fig. 10a: Long & Murry, 1995; fig. 107: Rinehart, Lucas & Heckert, 2024).

Diagnosis—. Metoposaurid differentiated from all other metoposaurids with referred material by a straight dorsal shaft of the ilium. Further differentiated from all other metoposaurids except *Metoposaurus* spp., *Panthasaurus maleriensis*, “*Arganasaurus*” *azerouali*, *Paranaschisma howardensis*, and *Apachesaurus gregorii* (in part) by a lacrimal that enters the orbital margin. Further differentiated from *Metoposaurus* spp. by a large region of reticulate ornamentation on the interclavicle and the clavicle. Further differentiated from “*Ar*.” *azerouali* by a wide and pentagonal squamosal, narrower cultriform process lacking reticulate ornamentation, and a lack of a commissure between the supraorbital and postorbital (=temporal sulcus + jugal sulcus) lateral line sulci. Further differentiated from *Pan. maleriensis* by a wide and blunt anterior end of the prefrontal, a maxilla-prefrontal contact, a marked flexure of the infraorbital sulcus on the lacrimal (either stepped or ‘Z-shaped’), and a parasagittally oriented parasphenoid-pterygoid suture (not oblique to the midline). Further differentiated from *Par. howardensis* by the absence of an extension of the ectopterygoid onto the palatal ramus and a cultriform process of the parasphenoid subequal to or narrower than the palatal ramus of the pterygoid. Differentiated from *Apachesaurus gregorii* by a temporal sulcus with a straight posterior terminus that does not pass onto or curve toward the squamosal. Further differentiated from *Pan. maleriensis*, “*Ar*.” *azerouali*, and *Par. howardensis* (in part) with referred material by the absence of a sensory groove on the clavicle. Further differentiated from all other metoposaurids except *Apachesaurus gregorii* with referred material by a centrally placed notochordal canal throughout the trunk intercentra. Further differentiated from all other metoposaurids except *Par. howardensis* and *A. gregorii* (in part) with referred material by the presence of a discontinuous transverse occipital sulcus.

Remarks—. “*Buettneria*” *perfecta* (=*Tecovaserpeton* gen. nov. *perfectum*) was a longstanding and widespread metoposaurid species from North America primarily defined by the lacrimal contribution to the orbital margin (Hunt, 1993). The taxonomic history of “*B*.” *perfecta* is complex and has been discussed in detail in several prior publications (Gee, Parker & Marsh, 2020; Hunt, 1993; Kufner & Gee, 2021; Mueller, 2007). Just over 30 years after the initial publication (Case, 1922), “*B*.” *perfecta* was synonymized with “*Buettneria*” *bakeri* (=*Buettnererpeton bakeri*), “*Buettneria*” *howardensis* (=*Paranaschisma* gen. nov. *howardensis*), and “*Metoposaurus jonesi*” under the subspecies “*Eupelor fraasi jonesi*” (Colbert & Imbrie, 1956) notably excluding “*Eupelor*” *browni* (=*Anaschisma browni*) known exclusively from the Popo Agie Formation of WY. Later (RoyChowdhury, 1965) proposed categorizing all metoposaurids under the genus *Metoposaurus* and maintained the subspecies distinction between “*Metoposaurus fraasi jonesi*” and “*Metoposaurus fraasi fraasi*,” the latter comprising specimens now considered non-diagnostic at the species or even genus level (Hunt, 1993). Murry (1986) implicitly considered these subspecies invalid, instead referring to the latter taxon as “*Metoposaurus fraasi*.” Hunt (1993) then published a revision of the Metoposauridae based on his firsthand observation of many of the nominal taxa and referred specimens and established a taxonomic framework that remains largely in use. Hunt (1993) maintained the synonymy of “*Buettneria*” *perfecta* and “*Buettneria*” *howardensis* and included “*Eupelor*” *browni*, “*Metoposaurus*” *maleriensis* (=*Panthasaurus maleriensis*), and some specimens previously referred to *Metoposaurus diagnosticus* under his concept of *Buettneria perfecta*. It was later recognized that the genus *Buettneria* was preoccupied by a bush cricket (*Buettneria maculiceps*), and the replacement name *Koskinonodon* was selected as the earliest diagnostic holotype and by page priority (Mueller, 2007). Later a redescription of the holotype of *A. browni* demonstrated the validity of *A. browni*, considered by Hunt (1993) to be non-diagnostic to the level of species, and established the priority of *A. browni* (Gee, Parker & Marsh, 2020). Some authors still consider *A. browni* non-diagnostic based on personal observations and maintain the use of “*Koskinonodon perfectum*” (Lucas, 2020; Rinehart, Lucas & Heckert, 2024).

**Description**

Sand Creek breaks—. UMMP 7475 was originally designated as the holotype of “*Buettneria perfecta*” (Case, 1922), and most metoposaurid specimens from the Dockum Group and the Chinle Formation that are not readily referable to *Buettnererpeton bakeri* and *Apachesaurus gregorii* have been assigned to this taxon and synonyms therein (Gee, Parker & Marsh, 2020; Hunt, 1993). UMMP 7475 is a very well-preserved skull from the Tecovas Formation of the Dockum Group in Texas (Figs. 34, 35). It is missing both tabular horns and part of the left ‘cheek,’ and in occipital view, it is apparent that it was slightly crushed resulting in a break between the skull roof and the basicranium spanning between the paraquadrate foramina (Fig. 34).

The details of the skull roof are largely in agreement with the initial description by Case (1922). A notable deviation present in some other referred specimens of *Tecovaserpeton perfectum* is a narrow contribution of the lacrimal to the orbital margin on the left side of UMMP 7475, but on the right side, this contact is broad with an elongate posterior process of the lacrimal extending to nearly the midlength of the orbit (Figs. 34A, 34B). The full complement of lateral line sulci is present apart from the transverse occipital sulcus likely due to incompleteness of both tabulars. Case (1922) illustrated the supraorbital sulcus and the infraorbital sulcus overlapping entirely on the maxilla, but this could not be confirmed by firsthand study; the sulci instead remain entirely separate as is typical. The supraorbital sulcus does not form a commissure with the temporal and jugal sulci (*contra* Case, 1922). An accessory jugal sulcus was identified on the right side of the skull roof, deviating from the jugal sulcus just anterior to the jugal-quadratojugal suture.

On the right side of the palate, the maxilla is essentially complete, and it contacts a slender process of the quadratojugal (Figs. 34C, 34D). The palatine-ectopterygoid suture was not previously identified, but it forms a ‘kinked’ suture. The *insula jugalis* is relatively short, not reaching half of the width of the palatal ramus where it inserts between the pterygoid and the ectopterygoid. The tooth bases of UMMP 7475 are preserved in nearly their entirety (Figs. 34C, 34D). There are 14 teeth on the right premaxilla and 12 on the left, and a pair of indentations just lingual to the marginal tooth row of the premaxilla were likely formed by the dentary fangs. Approximately 80 teeth are present on the right maxilla with space for more, potentially numbering more than 90. The transverse vomerine tooth row is asymmetric similar to the premaxillary teeth with nine teeth on the right side and eight on the left. Approximately 20 teeth are present in the parachoanal tooth row with two or three teeth present at the posterior margin of the choana separated from the row. There are ten teeth in the right palatine tooth row, and eight teeth in the left palatine tooth row all larger than the marginal maxillary dentition at the same level. The ectopterygoids bear approximately 30 teeth that gradually decrease in size posteriorly with 27 teeth on the right ectopterygoid and 26 on the left and space for more. Two pairs of fang bases are present, one pair on the vomer and one pair on the palatine. Four of these fang bases bear teeth, but they are distributed asymmetrically with the anterior fang positions bearing the teeth on the left vomer and palatine and the posterior fang positions bearing teeth on the right vomer and palatine.

The displacement of the occiput has resulted in some significant displacement of the vertical process of the exoccipital at about the level of the suture between the exoccipital and the tabular/postparietal (Fig. 35). The posttemporal fenestra is obscured by the displaced vertical process of the exoccipital, but a concave emargination on the dorsal surface of the vertical process likely forms the ventral border of the posttemporal fenestra. An additional indentation that may be a foramen is present just ventral to this emargination. The squamosal is excluded from contact with the quadrate by an extensive ventromedial process of the quadratojugal.

Rotten Hill—. The metoposaurid specimens of the Rotten Hill bonebed were recently described in detail, and as a result, only select specimens are included here to demonstrate some differences in interpretation of the anatomy and to describe the occipital region of the skull not previously published (Lucas et al., 2016). Metoposaurid crania from the Rotten Hill bonebed are invariably crushed inhibiting comparison of some of the features of the occiput and distorting primarily the posterior skull table, but the sutural contacts throughout the skull roof and surface details are well preserved. This is exemplified by WT 3055 (Fig. 36) in which the full complement of lateral line sulci and the dentition are readily identifiable.

A small element in the left external naris of WT 3055 is identified here as the septomaxilla (Figs. 36A, 36B), confirming its presence in at least some specimens referred to *Tecovaserpeton perfectum*. The lacrimal of WT 3055 contributes to the orbital margin, but its contact with this margin is very narrow with the prefrontal and jugal nearly contacting on the left side (Figs. 36A, 36B). A broad contact is otherwise widespread among the Rotten Hill specimens (*e.g*., Figs. 37, 38) including a smaller skull, WT 3011 (figs. 29c, 30c: Lucas et al., 2016). However, the actual contribution of the lacrimal to the orbital margin of WT 3011 is narrower than indicated by the pen markings on the specimen as the lacrimal-prefrontal suture approaches the orbit at an oblique angle (Fig. S3). Most of the Rotten Hill specimens have a parietal-supratemporal suture that is subparallel to the midline as in most other metoposaurid taxa (*e.g*., Figs. 37, 39), but WT 3055 (Fig. 36) has a modest oblique angle while WT 3003 (Fig. 38) has a much greater angle. A greater angle of the parietal-supratemporal suture is one of the apomorphies of *Metoposaurus krasiejowensis*, but in *M. krasiejowensis*, the parietal is so wide that it commonly contacts the postorbital in large specimens (Sulej, 2002, 2007). Lucas et al. (2016) illustrated a parietal-postorbital point contact in WT 3055 resulting from a medial extension of the postorbital, but this extension is instead the broken anterior process of the supratemporal (Fig. 36B). Specimen WT 3067-1 (figs. 29e, 30e: WT 3067 of Lucas et al., 2016) is unique in having a wider parietal than the frontal due to a greater angle of left parietal-supratemporal suture. Whether this variation is symmetric cannot be determined due to extensive deformation and reconstruction on the right side of WT 3067-1.

The lateral line sulci of the Rotten Hill metoposaurids had not previously been described and illustrated in detail, and all cranial lateral line sulci previously identified in metoposaurids were present on all specimens provided the associated cranial elements were preserved. The supraorbital sulcus of WT 3055 follows the typical pathway from the premaxilla-nasal joint around the external naris, lateral onto the maxilla then inflected medially at the level of the lacrimal around the medial margin of the orbit to its termination at about the midlength of the postfrontal (Figs. 36A, 36B). The supraorbital sulcus does not contact the lacrimal on the right side of WT 3055, but there is a very minor overlap on the left side. Damage to the right side of WT 3003 partially obscures the position of the supraorbital sulcus relative to the lacrimal, but on the left side, the supraorbital sulcus is very wide as it curves anterior to the orbit and extends onto the lacrimal (Fig. 38). The infraorbital sulcus follows the typical pathway from the lateral margin of the external naris then lateral to the orbit along the lateral margin. The infraorbital sulcus is only notable in that on the right side of WT 3055 the Z-shaped flexure is positioned anterior to the lacrimal perhaps owing to a less elongate lacrimal, and this is similarly the case in some other Rotten Hill crania (Figs. 36–38), though not all (Fig. 39). The temporal and the jugal sulci join to form a postorbital sulcus with the termination of the temporal sulcus on the supratemporal with a slight lateral curve at the termination (Figs. 36A, 36B). An accessory jugal sulcus departs from the main sulcus near the squamosal-quadratojugal suture, and the jugal sulcus exhibits a marked flexure at the posterior skull roof margin. The transverse occipital sulcus is incomplete and only present on the posterior margin of the tabular.

Much like other metoposaurids, the palates of the Rotten Hill specimens of *T. perfectum* are highly conserved. A small depression between the vomers just anterior to the transverse vomerine tooth row in WT 3055 is similar to the vomerine pit described in *Micropholis* and illustrated in *Mastodonsaurus* (Schoch, 1999; Schoch & Rubidge, 2005), although it is not clear if it is a throughgoing opening. The posterior end of each maxilla is broken, and it is not clear whether the maxilla and the quadratojugal would have contacted. Additional elements of the palate and occiput of WT 3114 (PPHM 1) were not previously figured but are included here to demonstrate rationale for character scores. The quadrate is typical in shape but slightly over prepared, and it is enveloped laterally by the quadratojugal and medially by the pterygoid with a minor contribution of the squamosal on the dorsomedial surface (Figs. 37A, 37C). Metoposaurid dentition is highly conserved, and the Rotten Hill metoposaurids are no exception. The only notable feature here not previously described is only evident in WT 3055, several depressions lingual to the marginal tooth row of the premaxilla and the anterior maxilla are likely pits formed by the anterior dentary teeth (gray circles with dashed outlines: Fig. 36D). The marginal tooth row of WT 3055 bears 13 teeth on the right premaxilla, 14 teeth on the left premaxilla, and at least 63 teeth on the left maxilla with space for 1–2 more teeth on each premaxilla and >10 more teeth on the left maxilla.

While the occipital region of most of the Rotten Hill metoposaurids is crushed and displaced, it is reasonably well preserved in one of the smallest specimens, WT 3011 (Fig. 40). Damage around the foramen magnum precludes identification of a lamellose process. The vertical process of the exoccipital is posteriorly and dorsally displaced on both sides of the occiput, slightly overlapping with the parotic process of the tabular and paroccipital process of the postparietal and obscuring the view of the posttemporal fenestra. A small foramen is present on the right vertical process of the exoccipital just ventral to the dorsal margin of the exoccipital. Like other metoposaurids, the tabular and the postparietal form a blade-like descending flange with the postparietal bearing a semicircular depression. A small flange projects from the parotic process of the tabular, just dorsal to the contact with the vertical process of the exoccipital. The right quadrate ramus is incomplete, but the orientation of the right ‘cheek’ suggests that the angle of inflection of the quadrate ramus of the pterygoid was greater than a ‘gradual slope,’ but this is equivocal. The margins of the paraquadrate foramen are partially preserved and suggest this opening was relatively narrow. No articulated or disarticulated stapes was identified in the collection at PPHM, and it is presumably missing due to taphonomic loss. As a result, any braincase elements not explicitly present are considered unknown due to varying degrees of ossification among taxa.

Unknown locality (MOTT VPL 0000)—. A relatively small (midline length ≈ 35 cm) though morphologically indistinguishable cranium, TTU-P09045, shares many features with the holotype of *Tecovaserpeton perfectum*. The occipital surface of TTU-P09045 is extensively damaged and not described here. The lacrimal contributes to the orbital margin, and the anterior process of the prefrontal is wide though slightly pointed (Figs. 41A, 41B). The more pointed morphology of the anterior end of the prefrontal could be attributed to ontogenetic variation as the prefrontal is more pointed in smaller crania from the Rotten Hill bonebed (figs. 28a, 29c: Lucas et al., 2016), and indeed, it is variable among larger specimens (*e.g*., figs. 33–34: Lucas et al., 2016). The remainder of the skull roof is typical for metoposaurids, but the right tabular lacks a pointed anterior process between the supratemporal and the squamosal similar to *Arganasaurus lyazidi* (Fig. 2). The full complement of cranial lateral line sulci is present on TTU-P09045 including a relatively well-developed accessory jugal sulcus (Figs. 41A, 41B).

The cultriform process of the parasphenoid is subequal in width to the minimal width of the palatal ramus of the pterygoid (Figs. 41C, 41D). The *insula jugalis* is relatively short, and the ectopterygoid does not form a postermedial extension onto the palatal ramus. The tooth positions are relatively well preserved for the palatal dentition, but the marginal dentition is only partially preserved. There are at least 12 teeth on the right premaxilla, but the remaining tooth rows of the maxilla and the left premaxilla are too incomplete to determine exact counts. The transvomerine tooth row on the right vomer bears 10 tooth bases, and only seven tooth cases could be identified on the left vomer with room for additional teeth. The parachoanal tooth rows are obscured by damaged to the medial margins of both choanae, but on the left palatine posterior to the choana, there is a short row of six tooth bases. The marginal tooth row on the left palatine bears 10 tooth bases much larger in diameter than the marginal tooth row of the adjacent maxilla. The ectopterygoid bears the remainder of the palatal tooth row with 25 tooth positions and space for at least 26 that diminish in diameter posteriorly. There is ornamentation on the parasphenoid, but it is restricted to the basal plate.

MOTT VPL 0696—. Green (1954) informally named “*Buettneria calgariensis*” in his PhD dissertation based on TTU-P09044, a large metoposaurid cranium from the Tecovas Formation of west Texas which is at least 55 cm in midline length, though the premaxillae are incomplete (Figs. 42, 43). The right side of the skull is significantly distorted, and the posterior skull table is extensively reconstructed (Fig. 42). Much of this reconstruction is sculpted and painted to appear like the surrounding material for display, hindering identification of the reconstructed portions. Ultraviolet fluorescence was used to discern reconstruction from the original material where possible, but the right antorbital skull roof may have either additional reconstruction that could not be readily discerned or significantly more plastic deformation than the left side_—_especially in the immediate antorbital region (Figs. 42A, 42B).

TTU-P09044 is readily referred to *Tecovaserpeton perfectum* by several features including: a lacrimal contribution to the orbital margin, the full complement of lateral line sulci, a relatively wide and blunt anterior process of the prefrontal, the absence of a posteromedial extension of the ectopterygoid, and a cultriform process subequal in width to the minimal width of the palatal ramus of the pterygoid (Figs. 42A–42D). The sutures of the *insula jugalis* are partially preserved and suggest that it did not occupy more than half of the width of the palatal ramus (Figs. 42C, 42D). Very faint and low ridges of ornamentation are present on the basal plate of the parasphenoid, but whether this faint ornamentation extends onto the cultriform process could not be determined due to damage in this region. The dentition is incompletely preserved throughout the palate hindering tooth counts.

The occiput of TTU-P09044 is partially obscured by putty acting as a support for the heavy elements of the skull (Fig. 43). The exoccipitals are posteriorly and dorsally displaced separating the occipital pillars at the sutures between the vertical process of the exoccipital and both the paroccipital process of the postparietal and the parotic process of the tabular. The dorsal displacement of the exoccipital obscures the posttemporal fenestra from occipital view. Damage to the lateral margin of the parotic process of the tabular prevents the identification of a laterally directed flange. The parotic process of the right tabular is entirely lateral to the preserved portion of the right exoccipital suggesting probable medial displacement of the right exoccipital or significant loss of its lateral margin. The squamosal forms a broad contact with the quadrate and excludes the quadrate from bordering the paraquadrate foramen. The borders of the left paraquadrate foramen are preserved in their entirety, but the foramen is infilled with putty. The left paraquadrate foramen is subequal to the foramen magnum in transverse width, and the right side is too reconstructed to determine.

UCMP V6333—. UCMP 113554 is a partially prepared cranium with a damaged occiput from an unknown stratigraphic level in the Dockum Group of Crosby County, TX. The suture configuration of the skull roof and the palate is broadly consistent with other metoposaurids from North America. The orbits are subcircular like those of some specimens of *Anaschisma browni* (Figs. 20, 22) and unlike the typical oval shape (Fig. 44). The narial-orbital distance is notably shorter than in other specimens of *Tecovaserpeton perfectum*. The lacrimal forms a broad contact with the orbit, and the supraorbital sulcus does not contact the lacrimal. The lateral line sulci of the posterior skull table exhibit an unusual configuration that may be the byproduct of preparation or preservation, but from my observation, these appear to be real. At the anterior extent of the temporal and jugal sulci on the right side a few sets of joined pits appear to represent a discontinuous transverse commissure of these sulci as well as a commissure with the supraorbital sulcus (Fig. 44). Unfortunately, this could not be confirmed on the left side of the skull roof due to damage to the surface of the postfrontal and the postorbital, and the left side lateral line sulci seem otherwise typical. The posterior extent of the right temporal sulcus has an abrupt lateral curvature and apparent expansions of the sulcus but does not appear to contact the squamosal, although there is extensive damage to the surface of the right squamosal. This appearance of the posterior terminus of the right temporal sulcus may be due to damage to the ornamentation artificially joining the sulcus into the adjacent pits. However, this is not mirrored on the left side, and if the temporal sulci were to terminate at the same level, they would appear typical for all non-*Apachesaurus* metoposaurids. An accessory jugal sulcus could not be identified on either side of UCMP 113554, but it could not be ruled out that this may be due to damage to the surface of the skull.

The anterior palate is reasonably well preserved and differs little from other metoposaurids described here (Figs. 44C, 44D). The cultriform process is not noticeably more narrow than other metoposaurids with the exception of the very wide cultriform process of *Paranaschisma*, but it does narrow to a minimum width at about its midlength that is less than the minimal width of the palatal ramus of pterygoid. While much of the basal plate of the parasphenoid is damaged, the region that would typically be ornamented is preserved, and no ornament was apparent. An *insula jugalis* frames the anterior margin of the subtemporal fenestra, but its sutures could not be fully defined due to damage on the palatine ramus of the pterygoid. The borders of the left paraquadrate foramen are preserved and indicate a width subequal to that of the foramen magnum unlike the narrower paraquadrate foramina of small specimens of *Apachesaurus* and more similar in proportion to large-bodied coeval metoposaurids (see Fig. S4). Little else can be discerned in occipital view other than the presence of an oblique crest of the pterygoid, a pterygoid depression, and a blade-like ventral extension of the postparietals—all typical features of the metoposaurid occiput. The occipital pillars surrounding the foramen magnum are heavily damaged, and the occipital condyles are entirely missing. The left stapes may be intact and articulated, but it is almost entirely obscured by matrix.

Holmes Creek—. UMMP 8854 is a large skull roof (midline length ≈ 55 cm) from the Tecovas Fm of Crosby County, TX (Fig. 45). The sutural patterns of the skull roof are consistent with those of specimens of *Tecovaserpeton perfectum*, *Paranaschisma howardensis*, and *Anaschisma browni* including a lacrimal contribution to the orbital margin. Unlike *A. browni*, UMMP 8854 has both an accessory jugal sulcus and a partial transverse occipital sulcus. Unlike most specimens of *P. howardensis*, the anterior end of the right prefrontal is relatively pointed, and the angle of the medial and lateral margins of the prefrontal anterior process is narrow. The partial transverse occipital sulcus on the right side is elongate extending from the end of the tabular horn to nearly the midline on the postparietal. Little can be discerned from the ventral view of the skull roof due to extensive damage and reconstruction. A few tooth bases can be seen on fragments of the maxilla, the ectopterygoid, and possibly the palatine, but none of these elements is complete enough to warrant a tooth count. Additional information on suture morphology of the skull roof such as scarf joints, butt joints, *etc*., is available due to the absence of all the palatal and braincase elements, but these cannot be compared with the vast majority of metoposaurid crania included here.

*Apachesaurus* Hunt, 1993

Diagnosis—. As for species by monotypy.

Type species—. *Apachesaurus gregorii*

*Apachesaurus gregorii* Hunt, 1993

Synonyms—. *Anaschisma* n. sp. Gregory, 1980

*Kalamoiketor pinkleyi* (?) Murry & Long, 1989

“*Anaschisma*” sp. Murry, 1989

“Small metoposaurid” Davidow-Henry, 1989

Figures 46–52

**Figure 46:**
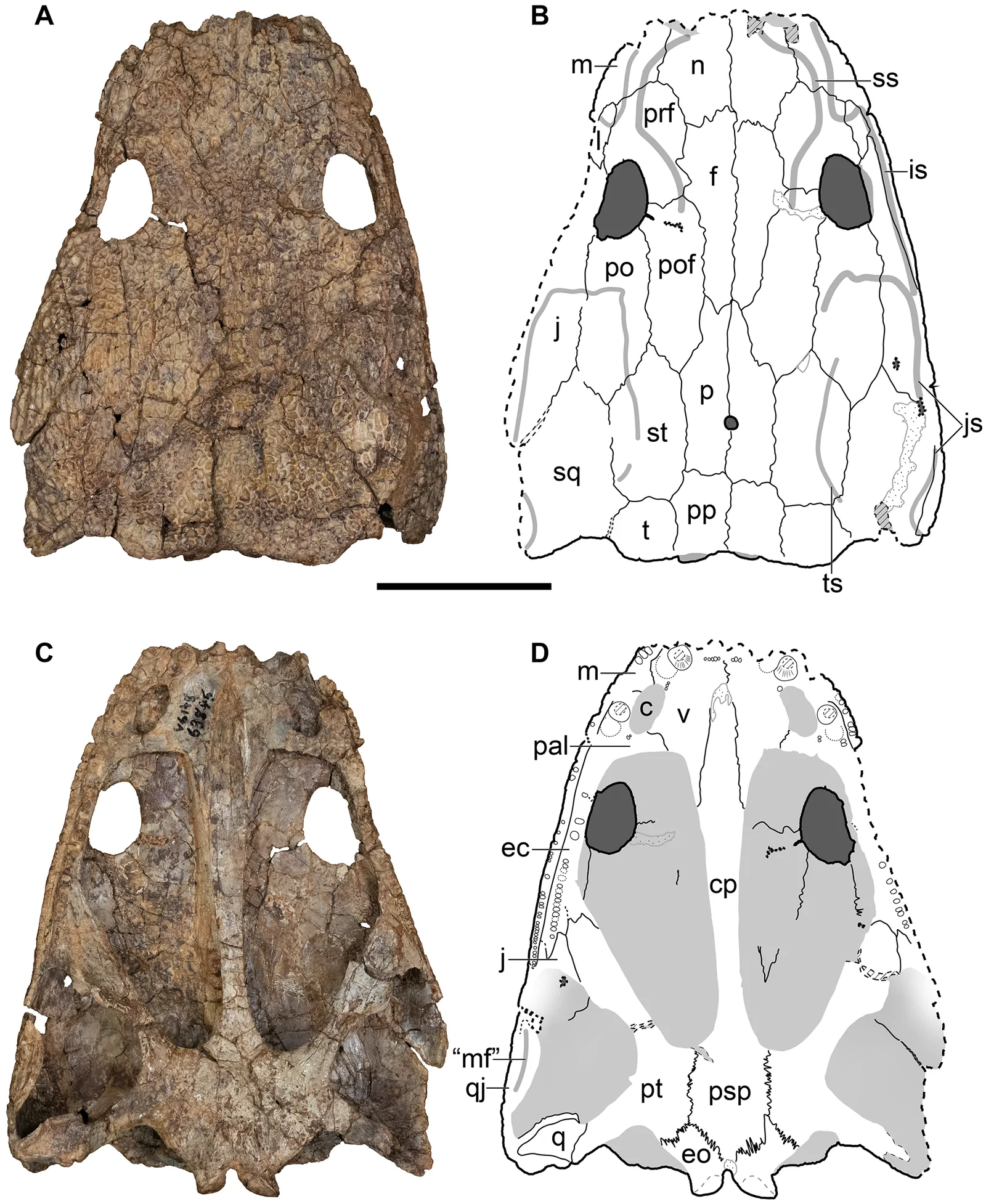
Holotype cranium of *Apachesaurus gregorii*, UCMP 63845, from UCMP V6148 (North Apache Canyon Quarry/Gregory’s Quarry 2) in Quay County, NM. (A) Photograph in dorsal view; (B) interpretive drawing of the same; (C) photograph in ventral view; (D) interpretive drawing of the same. Stippling represents residual matrix; diagonal lines represent broken surfaces; fine dashed gray lines represent topographic details like ridges. Abbreviations: c, choana (internal naris); cp, cultriform process; ec, ectopterygoid; eo, exoccipital; f, frontal; is, infraorbital sulcus; j, jugal; js, jugal sulcus; l, lacrimal; m, maxilla; “mf,” “maxillary furrow;” n, nasal; p, parietal; pal, palatine; po, postorbital; pof, postfrontal; pp, postparietal; prf, prefrontal; psp, parasphenoid; pt, pterygoid; q, quadrate; qj, quadratojugal; sq, squamosal; ss, supraorbital sulcus; st, supratemporal; t, tabular; ts, temporal sulcus; v, vomer. Scale bar equals 5 cm.

**Figure 47:**
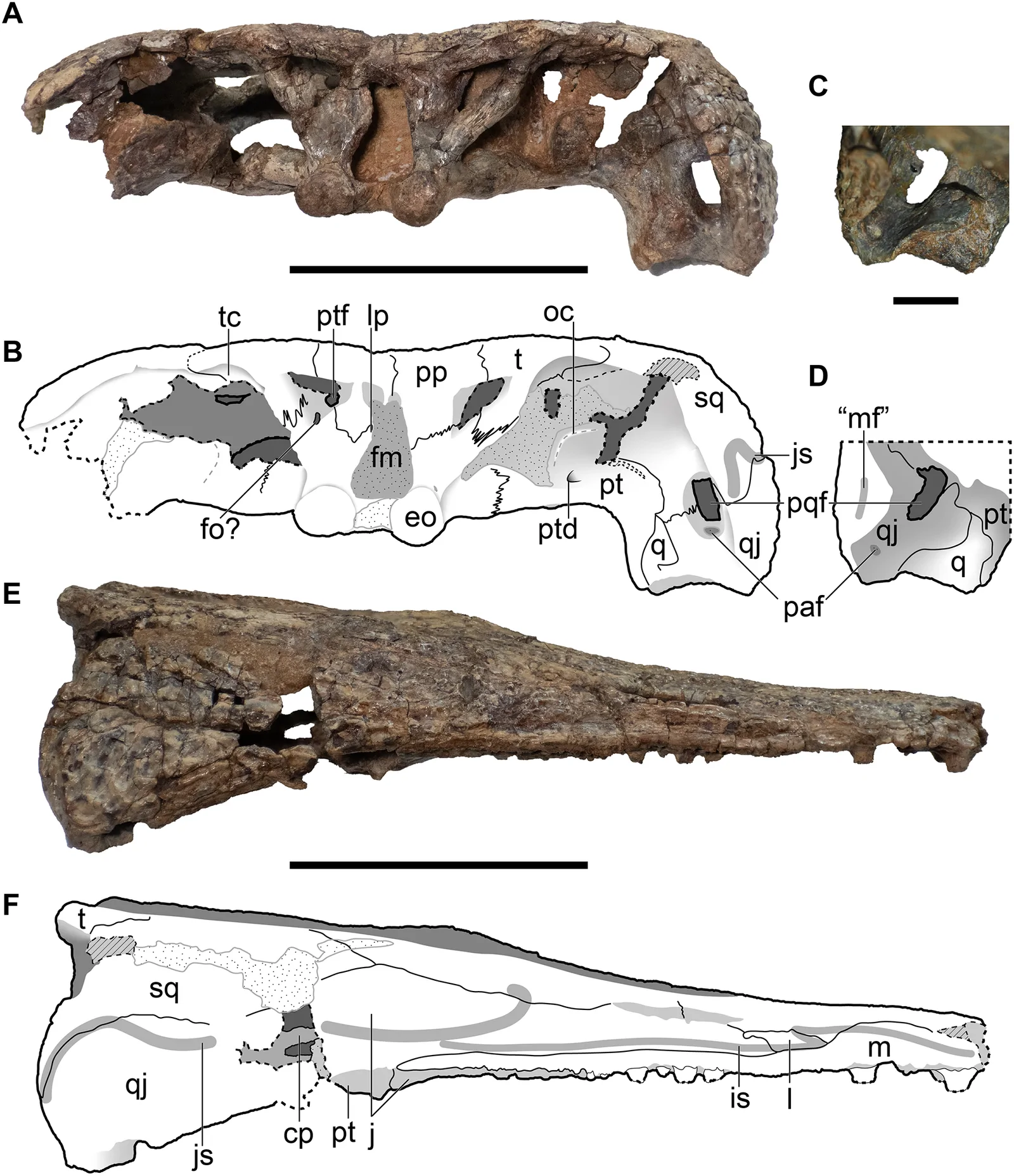
Holotype cranium of *Apachesaurus gregorii*, UCMP 63845, from UCMP V6148 (North Apache Canyon Quarry/Gregory’s Quarry 2) in Quay County, NM. (A) Photograph in occipital view; (B) interpretive drawing of the same; (C) photograph of supraglenoid region in anterior view; (D) interpretive drawing of the same; (E) photograph in right lateral view; (F) interpretive drawing of the same. Stippling represents residual matrix; diagonal lines represent broken surfaces; fine dashed gray lines represent topographic details like ridges. Abbreviations: cp, cultriform process; eo, exoccipital; fm, foramen magnum; fo?, foramen?; is, infraorbital sulcus; j, jugal; js, jugal sulcus; l, lacrimal; lp, lamellose process; m, maxilla; “mf,” “maxillary furrow;” oc, oblique crest of the pterygoid; paf, paraquadrate accessory foramen; pp, postparietal; pqf, paraquadrate foramen; pt, pterygoid; ptd, pterygoid depression; ptf, posttemporal fenestra; q, quadrate; qj, quadratojugal; sq, squamosal; t, tabular; tc, tympanic crest. Scale bars for (A, B) and (E, F) equal 5 cm; scale bar for (C, D) equals 1 cm. Photograph in (C) provided by C. So.

**Figure 48:**
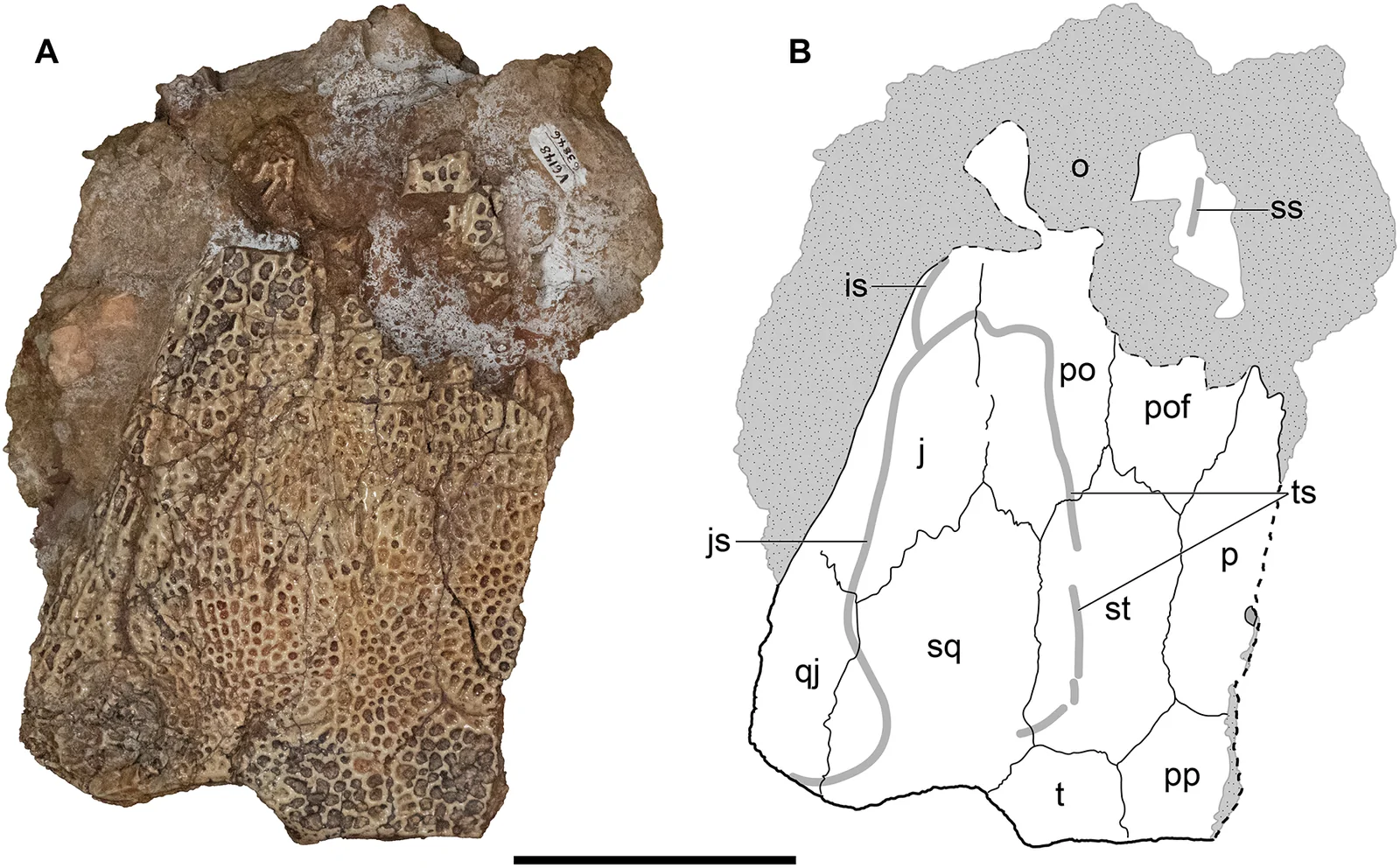
Referred posterior left skull roof of *Apachesaurus gregorii*, UCMP 63846, from UCMP V6148 (North Apache Canyon Quarry/Gregory’s Quarry 2) in Quay County, NM in dorsal view. (A) Photograph; (B) interpretive drawing. Stippling represents residual matrix. Abbreviations: is, infraorbital sulcus; j, jugal; js, jugal sulcus; o, orbit; p, parietal; po, postorbital; pof, postfrontal; pp, postparietal; qj, quadratojugal; sq, squamosal; ss, supraorbital sulcus; st, supratemporal; t, tabular; ts, temporal sulcus. Scale bar equals 5 cm.

**Figure 49:**
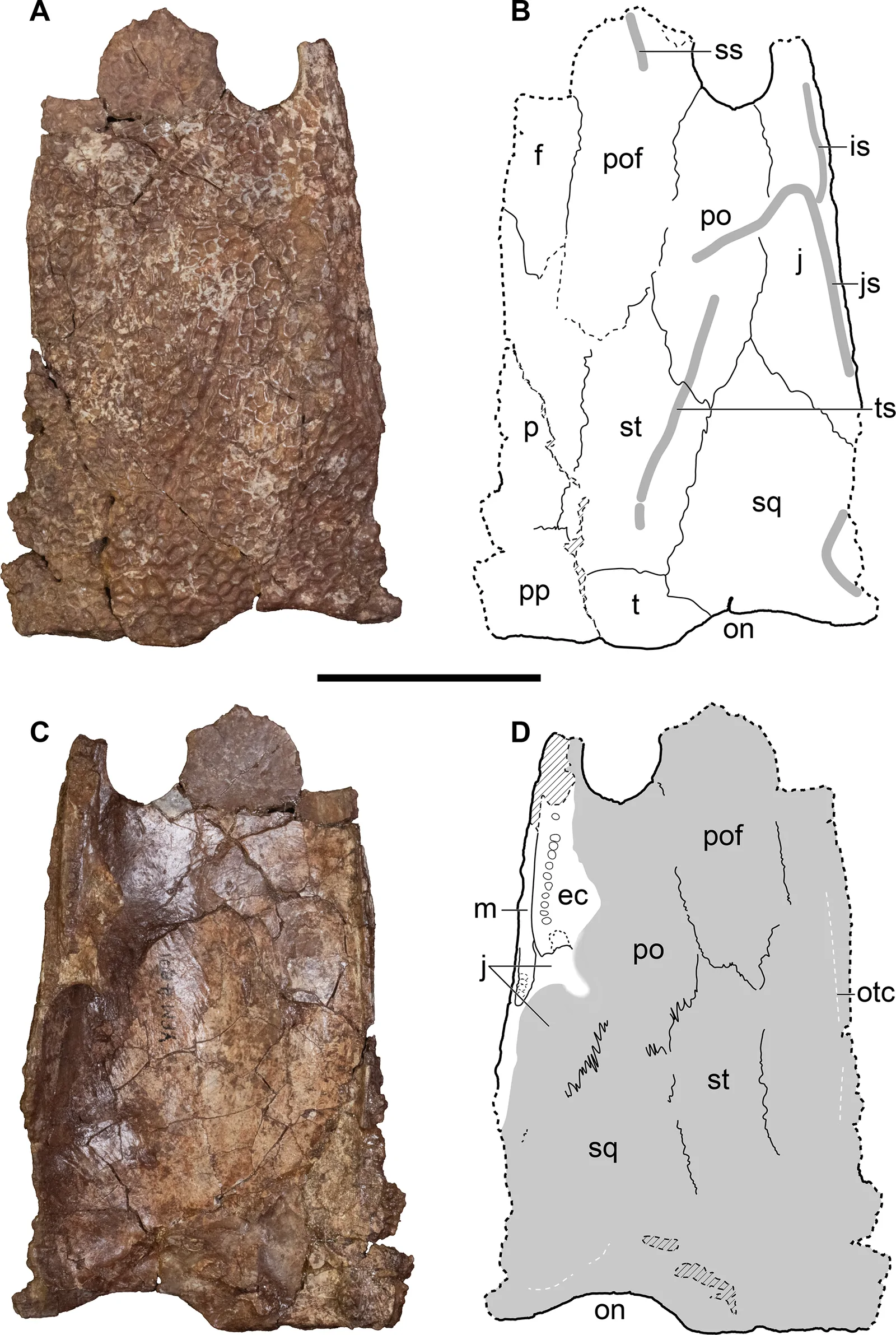
Referred partial right skull roof of *Apachesaurus gregorii*, YPM 4201, from YPM 6649 in Quay County, NM. (A) Photograph in dorsal view; (B) interpretive drawing of the same; (C) photograph in ventral view; (D) interpretive drawing of the same. Diagonal lines represent broken surfaces; fine dashed gray lines represent topographic details like ridges. Abbreviations: ec, ectopterygoid; f, frontal; is, infraorbital sulcus; j, jugal; js, jugal sulcus; m, slot on jugal for maxilla; on, otic notch; otc, orbitotemporal crest; p, parietal; po, postorbital; pof, postfrontal; pp, postparietal; sq, squamosal; ss, supraorbital sulcus; st, supratemporal; t, tabular; ts, temporal sulcus. Scale bar equals 5 cm.

**Figure 50:**
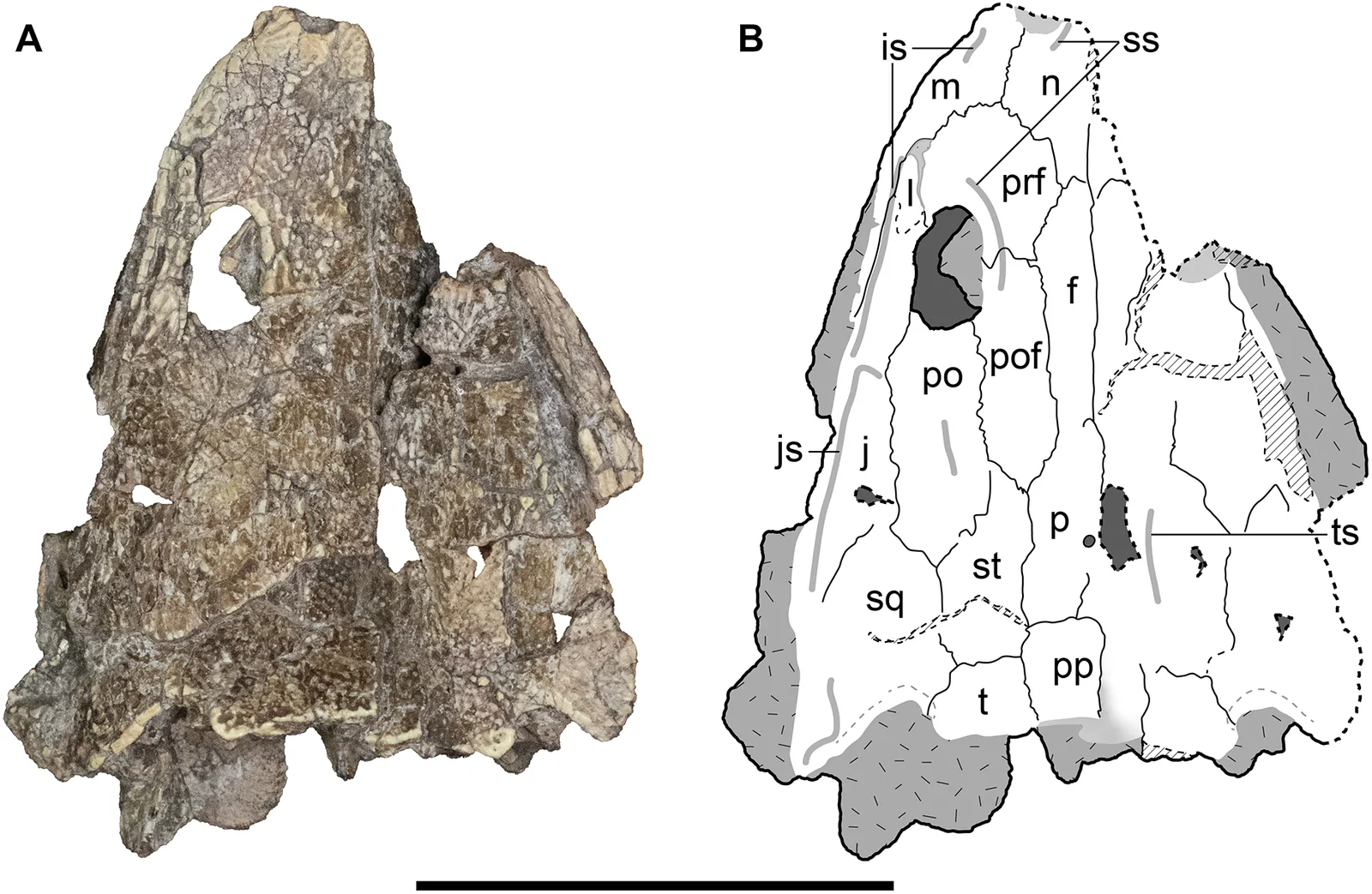
Referred cranium of *Apachesaurus gregorii*, UCMP 171591, in dorsal view from UCMP V82250 (Lacey Point or Inadvertent Hills) in Petrified Forest National Park, Apache County, AZ. (A) Photograph; (B) interpretive drawing. Diagonal lines represent broken surfaces; hatching represents bones other than the skull roof; fine dashed gray lines represent topographic details like ridges. Abbreviations: f, frontal; is, infraorbital sulcus; j, jugal; js, jugal sulcus; l, lacrimal; m, maxilla; n, nasal; p, parietal; po, postorbital; pof, postfrontal; pp, postparietal; prf, prefrontal; sq, squamosal; ss, supraorbital sulcus; st, supratemporal; t, tabular; ts, temporal sulcus. Scale bar equals 5 cm.

**Figure 51:**
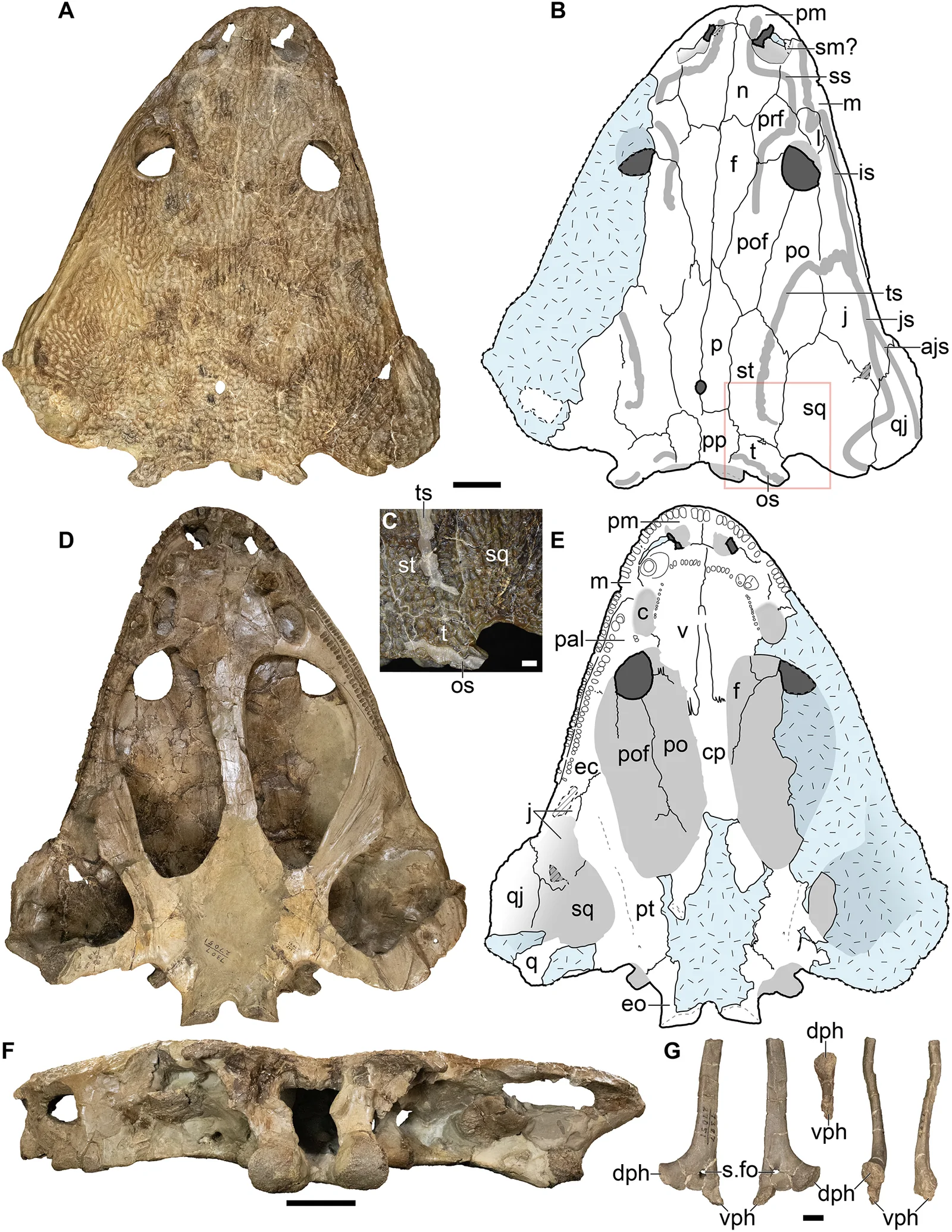
Partial cranium of a putative adult specimen of cf. *Apachesaurus gregorii*, UCMP 27051, from locality UCMP 7307 (Blue Hills 1) in Apache County, AZ. (A) Photograph in dorsal view; (B) interpretive drawing of the same; (C) close-up photograph of right temporal region with highlights showing sutures and lateral line sulci; (D) photograph in ventral view; (E) interpretive drawing of the same; (F) photograph in occipital view; (G) photographs of right stapes in (left to right) posterolateral, anteromedial, proximal, posterodorsal, and anteroventral views. Red square in (B) denotes the position of inset (C). Blue hatching represents reconstruction; diagonal lines represent broken surfaces; fine dashed gray lines represent topographic details like ridges; stippling represents residual matrix. Abbreviations: ajs, accessory jugal sulcus; c, choana (internal naris); cp, cultriform process of the parasphenoid; dph, dorsal proximal head; ec, ectopterygoid; eo, exoccipital; f, frontal; is, infraorbital sulcus; j, jugal; js, jugal sulcus; l, lacrimal; m, maxilla; n, nasal; p, parietal; pal, palatine; pm, premaxilla; po, postorbital; pof, postfrontal; pp, postparietal; prf, prefrontal; pt, pterygoid; q, quadrate; qj, quadratojugal; s.fo, stapedial foramen; sm?, septomaxilla?; sq, squamosal; ss, supraorbital sulcus; st, supratemporal; t, tabular; ts, temporal sulcus; v, vomer; vph, ventral proximal head. Upper scale bar for (A, B), (D, E); scale bar in inset for (C); lower left scale bar for (F); lower right scale bar for (G). Scale bars for (A, B) and (D–F) equal 5 cm; scale bars for (C–G) equal 1 cm.

**Figure 52:**
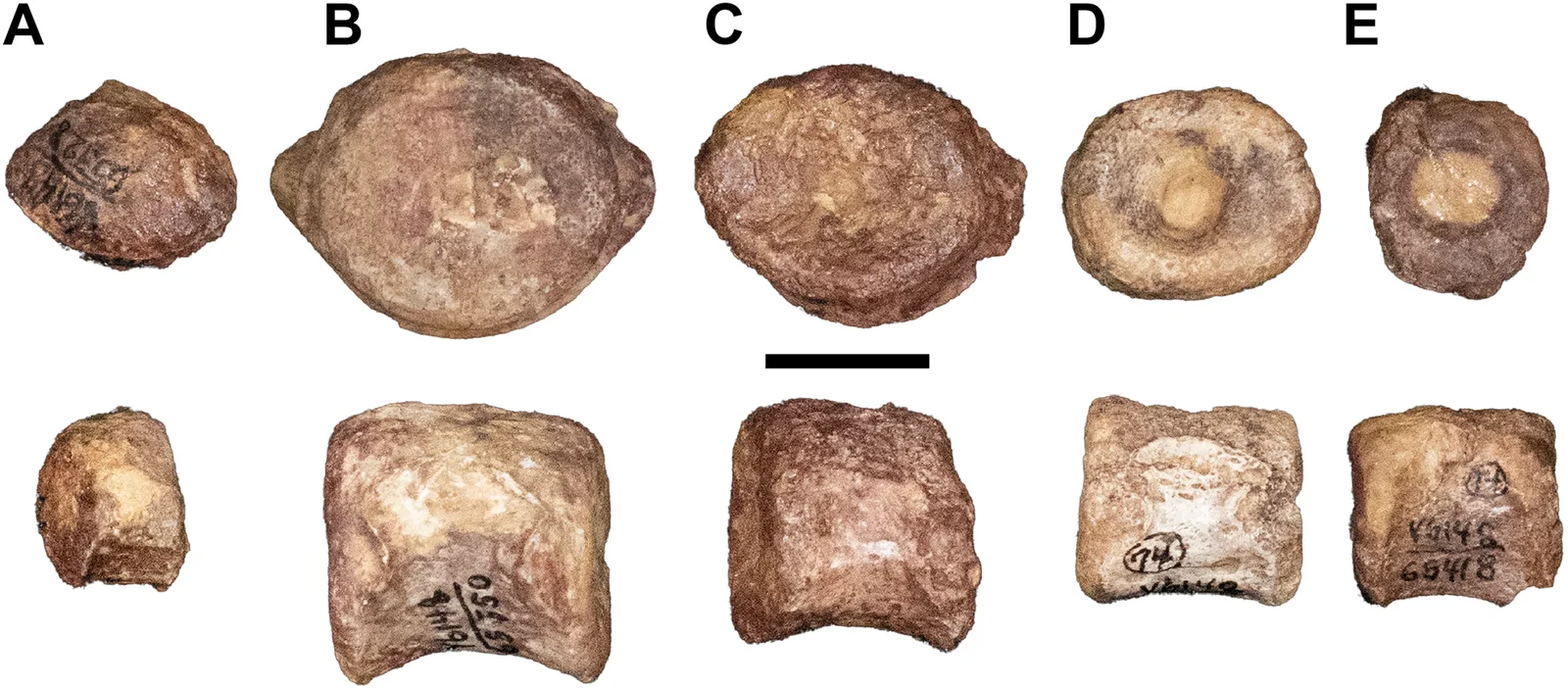
Photographs of referred intercentra of *Apachesaurus gregorii* from UCMP V6148 (Gregory’s Quarry 2) in Quay County, NM. Upper images in anterior view, lower images in left lateral view. (A) UCMP 65328, anterior dorsal; (B) UCMP 65550, presacral; (C) UCMP 65312, presacral; (D) UCMP 65417, presacral; (E) UCMP 65418, caudal. Scale bar equals 1 cm.

Holotype—. UCMP 63845, a nearly complete skull missing the premaxillae, the left quadrate, the left quadratojugal, and much of the left maxilla.

Type locality and horizon—. UCMP V6148 (North Apache Canyon Quarry/Gregory’s Quarry 2) in the Redonda Formation of Quay County, NM.

Referred material—. UCMP 63846, a partial left posterior skull roof; YPM 4201, partial right postorbital skull roof; YPM 4202, partial skull; YPM 4204, partial squamosal; UCMP 171591, partial cranium, mandible, intercentra, interclavicle, and clavicles; UCMP 27051, partial cranium and left hemimandible; UCMP 65313, partial skull; UCMP 63832, partial skull; UCMP 63847, 63848, partial right hemimandibles; UCMP 63849, nearly complete interclavicle; UCMP 63854, partial interclavicle; UCMP 63851, YPM 9628, partial clavicles; UCMP 4212, 4217, ilia; UCMP 65312, 65328, 65417, 65418, 65550, YPM 4222, intercentra.

Emended diagnosis—. Metoposaurid differentiated from all other metoposaurids by an elongated and curved temporal lateral line sulcus that may pass onto the squamosal, quadrate process of the pterygoid bent ventrally at an approximately 90° angle, and a consistently narrow cultriform process throughout ontogeny. Further differentiated from all metoposaurids except *Tecovaserpeton perfectum* by intercentra that form complete cylinders throughout the post-cervical trunk and into the anterior caudal series from early ontogeny and a centrally placed notochordal canal/pit throughout the trunk intercentra.

Remarks—. Sulej (2002) reported *Apachesaurus* from Popo Agie Formation based on Gregory’s (1980) referral of specimens from Quay County, NM to *Anaschisma* sp. The initial taxonomic assignment of UCMP 63845 to *Anaschisma* sp. by Gregory (1980) was due to a mischaracterization of the holotypes of *Anaschisma browni* and “*An. brachygnatha*” (=*An. browni*) as having an underdeveloped tabular horn and shallow otic notch when in reality this region in both specimens of *An. browni* is damaged, and the morphology of the tabular horn cannot be determined (Gee, Parker & Marsh, 2020; Hunt, 1993). A shallow otic notch and poorly developed or “absent” tabular horn has consistently been used to differentiate *Apachesaurus gregorii* from other metoposaurids, especially other North American metoposaurids (Hunt, 1993; Rinehart & Lucas, 2018; Spielmann & Lucas, 2012). Interestingly, widths of the skull measured across the tabular horns exhibit among the strongest positive allometry in the skull roofs of specimens from the Lamy amphibian quarry commonly referred to “*Koskinonodon perfectum*” (figs. 84, 86: Rinehart, Lucas & Heckert, 2024). Notably, positive allometry in the width across the tabular horns could indicate relative increase in width exclusively in the space between the tabular horns or by lengthening of the tabular horns, but this was not explicitly tested by Rinehart, Lucas & Heckert (2024). However, the width of the skull roof between the otic notches was found to have negative allometry, and when taken together with positive allometry in the tabular horn length, would suggest either progressive deepening of the otic notch, lengthening of the tabular horn, or both during ontogeny. Here a poorly developed tabular horn is not considered a diagnostic character but rather an indicator of early ontogeny. These results are consistent with the juvenile characteristics of vertebral specimens referred to *Apachesaurus* (Gee, Parker & Marsh, 2017). A small metoposaurid skull from Post Quarry in Texas (TTU-P9216) is commonly referred to *A. gregorii*, but this specimen is currently missing and could not be reassessed for this study (K Dean-Wallace, 2023, personal communication).

**Description**

UCMP V6148/Gregory’s Quarry—. The cranial osteology of the holotype of *Apachesaurus gregorii* has been described in detail (Spielmann & Lucas, 2012), but a brief redescription is undertaken here to demonstrate differences in interpretation from previous work. The topology of the sutural contacts of the dorsal skull roof is largely in agreement with previous descriptions (Hunt, 1993; Spielmann & Lucas, 2012). The occiput is almost entirely hidden in dorsal view which has previously been considered diagnostic of *Apachesaurus gregorii* (Hunt, 1993; Spielmann & Lucas, 2012), but here, this is considered more likely due to the excellent three-dimensional preservation of UCMP 63845 and common flattening and displacement of the palate/occiput in large metoposaurid skulls (*e.g*., Lucas et al., 2016; Rinehart, Lucas & Heckert, 2024).

The details of the cranial lateral lines are difficult to discern in most small metoposaurid skulls due to shallow sulci, conflation with elongate grooves associated with zones of rapid growth, and the increased potential for overpreparation on small specimens. Contrary to previous descriptions (Hunt, 1993; Rinehart & Lucas, 2018; Spielmann & Lucas, 2012), nearly the full complement of cranial lateral line sulci typically found in metoposaurids is present in UCMP 63845 with the exceptions of the accessory jugal sulcus and the transverse occipital sulcus (Figs. 46A, 46B). The infraorbital sulcus follows the typical pathway seen in other metoposaurids from the lateral external naris, continuing lateral to the orbit while following the maxilla-jugal suture. Contrary to previous descriptions, the infraorbital sulcus exhibits a stepped (nearly Z-shaped) flexure on the lacrimal, not as dramatic as that seen in most other metoposaurids, but also not the simple curvature depicted by previous authors (fig. 12b: Hunt, 1993; fig. 10a: Spielmann & Lucas, 2012). The infraorbital sulcus merges with the jugal sulcus at its posterior extent (Figs. 46B, 47F). An additional sulcus passes near the ventral margin of the quadratojugal that has been curled over such that it is only visible in ventral view (Figs. 46C, 46D). A similar sulcus has been identified in *Almasaurus habbazi* and referred to as the “maxillary furrow” (fig. 80: Dutuit, 1976a). It may be continuous with the mandibular lateral line sulci, but this sulcus is presently unnamed due to uncertainty in its homology. The supraorbital sulcus terminates just posterior to the prefrontal-postfrontal suture, well anterior to the typical termination in larger metoposaurids near the middle of the postfrontal (*e.g*., Figs. 8, 22, 24). The temporal sulcus is present but discontinuous (*contra* Hunt, 1993; Spielmann & Lucas, 2012), and unlike other metoposaurids, it curves beginning at about the midlength of the supratemporal and reaches the supratemporal-squamosal suture (Figs. 46A, 46B). The jugal sulcus reaches the posterior margin of the skull roof, and the curvature of its posterior extent approximately mirrors the squamosal-quadratojugal suture as it does in other metoposaurids (Figs. 46B, 47F). The commissure between the temporal and jugal sulci (forming a combined postorbital sulcus) is primarily transverse and has a weakly sinuous curvature unlike most other metoposaurids. Slight overpreparation or damage to the skull roof of UCMP 63845 may have resulted in some of the differences in interpretation between this study and previous studies, but the lateral line sulcus morphology of the posterior skull roof is confirmed by a referred skull roof, UCMP 63846.

The *fodina vomeralis*, an opening often present between the vomers at the anterior apex of the cultriform process of the parasphenoid, was previously considered absent, but upon reexamination, it is evident that there is matrix infilling this space (Fig. 46). It is still unclear whether a throughgoing foramen or a shallow pit is present in this position. A palatal process of the jugal (*i.e*., the *insula jugalis*) is readily apparent forming the anterior margin of the subtemporal fenestra in the palate (Figs. 46C, 46D); the *insula jugalis* sutures to the maxilla anterolaterally, the ectopterygoid anteriorly, and the pterygoid medially, forming the anterior border of the subtemporal fenestra.

No stapes or supraoccipital were identified in my reexamination of UCMP 63845 (*contra* Spielmann & Lucas, 2012). The structure previously identified as the stapes is instead the tympanic crest of the tabular (see Sulej, 2007). The structure previously identified as the supraoccipital is not clearly separated from the exoccipital by a suture, but instead, the purported sutures are indistinguishable from fractures in this region of the skull (Figs. 47A, 47B). A slight protrusion of the exoccipital medially into the foramen magnum (Figs. 47A, 47B) is almost certainly the lamellose process which may have supported a cartilaginous supraoccipital (Sulej, 2007). The surface of the quadrate of metoposaurids is typically unfinished (cancellous), and it is enveloped by processes of the pterygoid, quadratojugal, and sometimes by a minor contribution of the squamosal. The quadrate is similar in UCMP 63845 with a broad ventral process of the quadratojugal almost completely enveloping the quadrate in occipital view and a short contact between the squamosal and quadrate (Figs. 47A, 47B). The posttemporal fenestra of *A. gregorii* is typically considered triangular in outline (Spielmann & Lucas, 2012), but it is instead a triangular depression surrounding the typical small, round posttemporal fenestra (Figs. 47E, 47F) differing from the polygonal opening in some other stereospondyls (*e.g*., Fernández-Coll et al., 2019). A depression adjacent to the paraquadrate foramen is infilled with matrix was previously identified as an accessory paraquadrate foramen (fig. 11: Spielmann & Lucas, 2012). That identification is upheld and clarified by the presence of a foramen on the internal surface of the quadratojugal that likely communicates with the external opening (Figs. 47C, 47D), though X-ray scanning may be necessary to verify this connection. Gee & Kufner (2022) excluded the paraquadrate accessory foramen previously identified in *Metoposaurus krasiejowensis* from their character matrix due to the appearance of a thin medial wall that may be prone to damage during deposition or preparation (*e.g*., fig. 1d: Sulej, 2007). However, the pathway of the paraquadrate accessory foramen in UCMP 63845 is much longer and surrounded by more substantial bone than a simple strut similar to that described in *Benthosuchus sushkini* (fig. 16: Bystrow & Efremov, 1940). Regardless, the thin lamina forming the medial wall of the paraquadrate accessory foramen could be readily damaged, and the internal opening is often obscured by the ventral process of the quadratojugal in ventral view, so careful examination of this region in other metoposaurids will be necessary in future studies.

UCMP 63846, a referred skull roof from the type locality of *Apachesaurus gregorii*, is slightly larger and nearly indistinguishable from the holotype (Fig. 48). The tabular of UCMP 63846 has a more squared off posterolateral margin similar to the condition considered a “moderately developed” tabular horn in some specimens of *Arganasaurus lyazidi* (Buffa, Jalil & Steyer, 2019; Dutuit, 1976b) unlike the entirely rounded margin in UCMP 63845. The temporal sulcus is present but discontinuous on the supratemporal with some small pits separating it into anterior and posterior segments and the posterior segment further subdivided. The posterior segment curves laterally to reach the supratemporal-squamosal suture as in UCMP 63845 near the juncture with the tabular (Fig. 48). The jugal sulcus exhibits a much more dramatic medial curvature over its posterior extent, not closely mirroring the squamosal-quadratojugal suture.

YPM 4201 is a partial right posterior skull roof and among the largest cranial specimens that have been referred to *Apachesaurus gregorii* (Fig. 49; Hunt, 1993; Spielmann & Lucas, 2012). The general pattern of sutures, the shallow otic notch, and the relatively undeveloped tabular horn are consistent with other specimens of *A. gregorii*. The rounded posterior margin of the tabular is more similar to that seen in the smaller holotype (Fig. 46) than in the referred skull from the type locality (Fig. 48). A short section of the margin of the pineal foramen is tentatively identified, and it is positioned relatively more posterior between the parietals than in the type locality specimens and a small, referred specimen (UCMP 171591, see below). The supraorbital sulcus terminates at about the same level in YPM 4201 as in UCMP 63845 (Figs. 46, 49). The temporal sulcus is discontinuous as in the type locality specimens (Figs. 46A, 46B, 49A, 49B), and the anterior portion of the temporal sulcus is significantly medially displaced compared to the posterior segments, similar to the “accessory temporal sulcus” seen in some trematosaurs (see Appendix 1) though here not considered homologous. Unlike the type specimens, the temporal sulcus of YPM 4201 terminates on the supratemporal, but there is a weak lateral deflection at its posterior extent also seen in other metoposaurids that do not have a temporal sulcus that contacts the squamosal. The only portion of the palate preserved is the portions of the jugal and ectopterygoid that contact the palatal ramus of the pterygoid (Figs. 49C, 49D). The ectopterygoid forms a posteromedial projection, and the *insula jugalis* is short and forms the anterior margin of the subtemporal fenestra. A short segment of the posterior maxilla is preserved with some tooth attachment sites, and the anterior extent of the maxilla is discernable *via* its slotted attachment to the jugal. The marginal tooth row is partially preserved on the ectopterygoid with 11 positions and space for more.

UCMP V82250—. A small metoposaurid skull (Fig. 50) with an associated mandible and some postcrania, UCMP 171591, from the site UCMP V82250 (referred to as Lacey Point quarry (Long & Murry, 1995; Parrish, 1991) or Inadvertent Hills (UCMP collection; Rinehart & Lucas, 2018)) in Petrified Forest National Park, Apache County, AZ was originally reported as UCMP 82/39/37 in an undergraduate thesis and included in several subsequent studies (Davidow-Henry, 1987, 1989; Hunt, 1993; Hunt & Lucas, 1993; Spielmann & Lucas, 2012) including a recent description after additional preparation at the New Mexico Museum of Natural History (NMMNH; Rinehart & Lucas, 2018). This specimen has typically been referred to *Apachesaurus gregorii* due to an undeveloped tabular horn, lacrimal separated from the orbital margin, and anteroposteriorly elongate intercentra (Rinehart & Lucas, 2018). The lacrimal is short and compact much like UCMP 63845 (Figs. 46A, 46B). The anterior contacts of the lacrimal are slightly separated from the maxilla and the prefrontal, and the posterior suture with the jugal is unclear (Fig. 50). While the pineal foramen is located within the posterior half of the parietals, it is situated relatively far forward like in the type locality specimens of *A. gregorii* (Figs. 46, 48). Of the cranial lateral line sulci of UCMP 171591, only a short segment of the jugal sulcus was previously noted (Rinehart & Lucas, 2018). Here each of the cranial lateral line sulci except the accessory jugal sulcus and the transverse occipital sulcus were observed (Fig. 50). All lateral line sulci are incomplete and shallow, and notably more incomplete than other larger juvenile specimens of *A. gregorii*, likely owing to the early ontogenetic stage of UCMP 171591.

UCMP 7307—. Specimen UCMP 27051 from UCMP 7307 (Blue Hills 1) situated in the lower Petrified Forest Member of the Chinle Formation is a partial cranium, stapes (Fig. 51), and mandible (not figured due to extensive reconstruction). UCMP 27051 is referred to *Apachesaurus gregorii* based on the configuration of the lateral line sulci and form of the palate. The suture pattern of the skull roof is broadly consistent with other metoposaurids described here, with only differences noted. A small element likely representing a partial septomaxilla is present on the lateral margin of the right external naris at the juncture between the premaxilla, the maxilla, and the vomer (Figs. 51A, 51B). The parietal is just shorter than the frontal along the midline being almost subequal in length. The pineal foramen is located well posteriorly, much more so than in the type locality and referred specimens of *A. gregorii* (Figs. 46, 48, 50). The right temporal sulcus curves laterally and narrows at its posterior terminus and nearly contacts the squamosal (Figs. 51A, 51C). Whether the temporal sulcus crosses the supratemporal-squamosal suture cannot be determined because a hiatus in ornament demarcates this suture. The left temporal sulcus also exhibits a marked lateral curvature, and it does not reach the squamosal, terminating at a more anterior position on the supratemporal (Figs. 51A, 51B).

The cultriform process is narrow, certainly narrower than other large-bodied metoposaurid specimens described here and narrower than the minimum width of the palatal ramus of the pterygoid (Figs. 51D, 51E). Crushing has resulted in the ‘cheek’ and jaw articulation flaring laterally, possibly artificially imposing a gradual slope on the quadrate ramus of the pterygoid which has been partially reconstructed with plaster/putty (Fig. 51F). If reconstructed, the pterygoid and ‘cheek’ should exhibit a greater inflection, though the degree to which this inflection would be in line with the holotype of *A. gregorii* is unclear (Figs. 47A, 47B). The dentition is partially preserved as tooth bases and four palatal fangs, but the posterior ends of the both the maxilla and the ectopterygoid are not preserved (Figs. 51D, 51E). The right premaxilla bears 11 tooth bases with space for two more teeth. The transverse vomerine tooth row bears seven tooth bases on the right side and six on the left with space for one or two more teeth on both sides. The parachoanal tooth rows are incomplete along the posteromedial margin of the choana on both sides with nine tooth bases preserved on the right side and 11 on the left. A pair of tooth bases is present posterior to the right choana. Approximately 11 tooth bases are present on the right palatine, but the palatine-ectopterygoid suture is estimated. Both palatal fangs are present on the left vomer, but only one is present on the right vomer and the right palatine.

A stapedial foramen is present between the proximal heads of the stapes which is unknown in other metoposaurids (Fig. 51G). The end of the ventral proximal head is finished as it is in a stapes of *Paranaschisma howardensis* (Fig. 31E) and a stapes of *Buettnererpeton bakeri* (fig. 18b: Gee & Kufner, 2022), but the dorsal proximal head is unfinished presumably for the attachment of a cartilaginous cap (see Schoch, 2000). The anterior and transverse crests are indistinct unlike the comparable and presumably adult stapedes of *P. howardensis* (Fig. 31E) and *B. bakeri* (fig. 31: Gee & Kufner, 2022).

Mandible—. No significant differences from previous descriptions of mandibular material from the type locality of *Apachesaurus gregorii* were noted. However, an element identified as a mandible listed under UCMP 171591 from UCMP V82250 was previously referred to *A. gregorii* (fig. 3: Rinehart & Lucas, 2018), but this identification is questionable. The “mandible” entirely lacks sutures (*contra* Rinehart & Lucas, 2018), and the internal structure visible on the broken ends is highly porous and not at all similar to the internal structure of metoposaurid mandibles along the mandibular ramus or posterior to the adductor chamber as originally proposed (see fig. 4: Gruntmejer et al., 2018). An ilium referred to the archosauriform *Vancleavea* was recovered from the same locality (UCMP 165197; not figured), and this unknown element of UCMP 171591 may be one of the ornamented cranial or pectoral girdle elements of *Vancleavea* or a related doswelliid (Dilkes & Sues, 2009; Nesbitt et al., 2009).

Intercentra—. The elongate intercentra of *Apachesaurus gregorii* have long been considered diagnostic for the genus, leading to the assignment of cylindrical intercentra with intersegmental parapophyses to *Apachesaurus* and a long stratigraphic range of this taxon (~20 Ma). Notably, all known elongate intercentra assigned to *Apachesaurus* also possess a wide and typically throughgoing notochordal canal. An anterior dorsal intercentrum, UCMP 65328, identified by a opisthocoelous body and broad parapophysis is present among the referred intercentra from the type locality of *A. gregorii* but was not previously figured (Fig. 52A). UCMP 65328 lacks a notochordal canal or pit, and unlike other intercentra from UCMP V6148, it is strongly opisthocoelous. UCMP 65328 is also notable in being much shorter in length than height (height/length ≈ 1.5), not fitting the diagnosis of elongate intercentra (diameter/length < 0.8) for *A. gregorii* provided in the original description and revisions (Hunt, 1993; Rinehart & Lucas, 2018; Spielmann & Lucas, 2012).

Three of the remaining intercentra are presacral (*sensu* Konietzko-Meier, Bodzioch & Sander, 2012) identified by an anterior and posterior parapophysis (intersegmental) at about the midheight of the centrum (Figs. 52B–52D). These were previously described as generalized dorsal intercentra (Hunt, 1993) and also “cervical” intercentra (Spielmann & Lucas, 2012), but they entirely lack the characteristic angled and singular parapophysis of cervical/postcervical intercentra or a partial diapophysis (see Dutuit, 1976b; Konietzko-Meier, Bodzioch & Sander, 2012; Sulej, 2007). These three intercentra have also been described as amphicoelous (Hunt, 1993) and procoelous (Spielmann & Lucas, 2012), but the vertebral body shape would be better described as either acoelous or weakly amphicoelous with the concavities on the anterior and posterior faces being due entirely to the presence of a notochordal canal or furrow. The caudal intercentrum UCMP 65418 was previously described but not figured; UCMP 65418 forms an elongate cylinder with a prominent and relatively large notochordal canal through the center of the intercentrum (Fig. 52E). The hemal arches were coossified with the intercentrum, but they are broken off at the point where they would narrow and separate from the body of the vertebra.

Additional comments on postcrania—. An ilium from the type locality of *Apachesaurus gregorii* (UCMP 65307, erroneously listed as 65309) was previously referred to *A. gregorii* (figs 26a–b: Spielmann & Lucas, 2012). UCMP 65307 is unlike any metoposaurid or even stereospondyl ilia (*e.g*., fig. 62: Gee & Kufner, 2022; fig. 11: Kufner & Gee, 2021; fig. 27: Spielmann & Lucas, 2012) in having a broad concave acetabulum nearly the length of the base of the ilium, a narrow mediolateral profile lacking a prominent supraacetabular buttress, and a flattened dorsal shaft with a broad proximal expansion (figs. 26a–b: Spielmann & Lucas, 2012). UCMP 65307 instead appears much more similar to ilia of *Vancleavea campi* (see fig. 7: Parker & Barton, 2008; fig. 28: Spielmann & Lucas, 2012).

Metoposauridae indet.

Several alpha taxa have been erected based on material from North America that demonstrate clear metoposaurid affinities but lack diagnostic characters at the species level in the current taxonomic framework. The taxa *Metoposaurus fraasi* Lucas, 1904, *Metoposaurus jonesi* Case, 1920, and *Buettneria major* Branson & Mehl, 1929 are based exclusively on postcranial material and have been regarded as non-diagnostic below the family level for decades (see Hunt, 1993). These clavicles and interclavicles all possess the wide region of ornamentation common among North American metoposaurids. *Kalamoiketor pinkleyi* Branson & Mehl, 1929 is based on a partial skull from the Chinle Formation (MU 554) found about 8 miles northwest of Adamana, AZ. The exact horizon from which MU 554 was recovered is unclear with some previous authors suggesting either the Blue Mesa Member or the lower Petrified Forest Member of the Chinle Fm (Hunt, 1993; Long & Murry, 1995). MU 554 is in severe disrepair (A. Kufner, 2026, personal observations), but with a renewed conservation effort on this specimen, the validity of *K. pinkleyi* may soon be reassessed.

**Figure 53:**
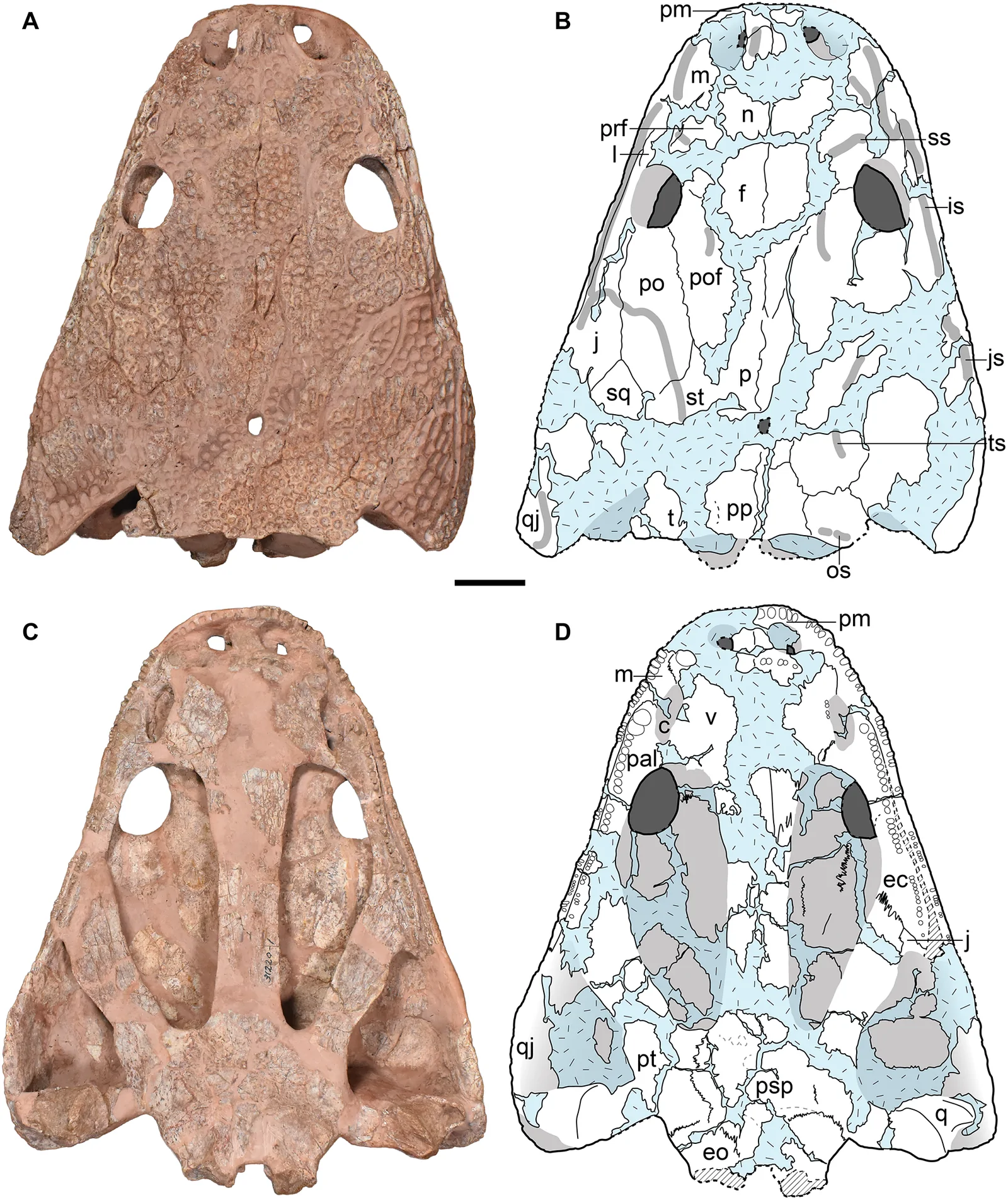
Cranium of Metoposauridae indet., TMM 31220-1, from Otis Chalk Quarry 4 in Howard County, TX. (A) Photograph in dorsal view; (B) interpretive drawing of the same; (C) photograph in ventral view; (D) interpretive drawing of the same. Blue hatching represents reconstruction; fine dashed gray lines represent topographic details like ridges; diagonal lines represent broken surfaces. Abbreviations: c, choana (internal naris); ec, ectopterygoid; eo, exoccipital; f, frontal; is, infraorbital sulcus; j, jugal; js, jugal sulcus; l, lacrimal; m, maxilla; n, nasal; os, transverse occipital sulcus; p, parietal; pal, palatine; pm, premaxilla; po, postorbital; pof, postfrontal; pp, postparietal; prf, prefrontal; psp, parasphenoid; pt, pterygoid; q, quadrate; qj, quadratojugal; sq, squamosal; ss, supraorbital sulcus; st, supratemporal; t, tabular; ts, temporal sulcus; v, vomer. Scale bar equals 5 cm.

Otis Chalk Quarry 4—. Specimen TMM 31220-1 is commonly considered a syntype of *Paranaschisma howardensis*, but due to incompleteness of TMM 31220-1 and the resulting uncertainty in phylogenetic position, it is here referred to Metoposauridae indet. (Fig. 53). In addition to poor preservation, the stratigraphic position of Quarry 4 cannot be reliably correlated with Quarry 3 at present (Long & Murry, 1995; Sawin, 1944), and with the clear differences of metoposaurid taxa between Otis Chalk quarries 2 and 3, even similarity in stratigraphic level is not sufficient to justify referral without clear apomorphies. Some details of the skull roof suggest affinities with either *P. howardensis* or *Tecovaserpeton perfectum* including a lacrimal contribution to the orbital margin, extensive ornamentation on the basal plate of the parasphenoid (possibly though equivocally extending onto the cultriform process), and the presence of a partial transverse occipital sulcus on the tabular. The tabular horn is directed at an angle approximately orthogonal to the midline of TMM 31220-1, a feature shared with only one other metoposaurid specimen described here (see below), but the degree to which this is due to a fracture at the base of the tabular horn is unclear. Differing from the syntypes of *P. howardensis*, TMM 31220-1 lacks a clear posteromedial extension of the ectopterygoid (Figs. 53C, 53D). The *insula jugalis* is also relatively short, a condition similar to some specimens of *T. perfectum* including the holotype (see Figs. 34, 36, 41, 44) but distinct from the large *insula jugalis* of some specimens of *P. howardensis* including the lectotype (see Figs. 24, 26, 30, 32).

**Figure 54:**
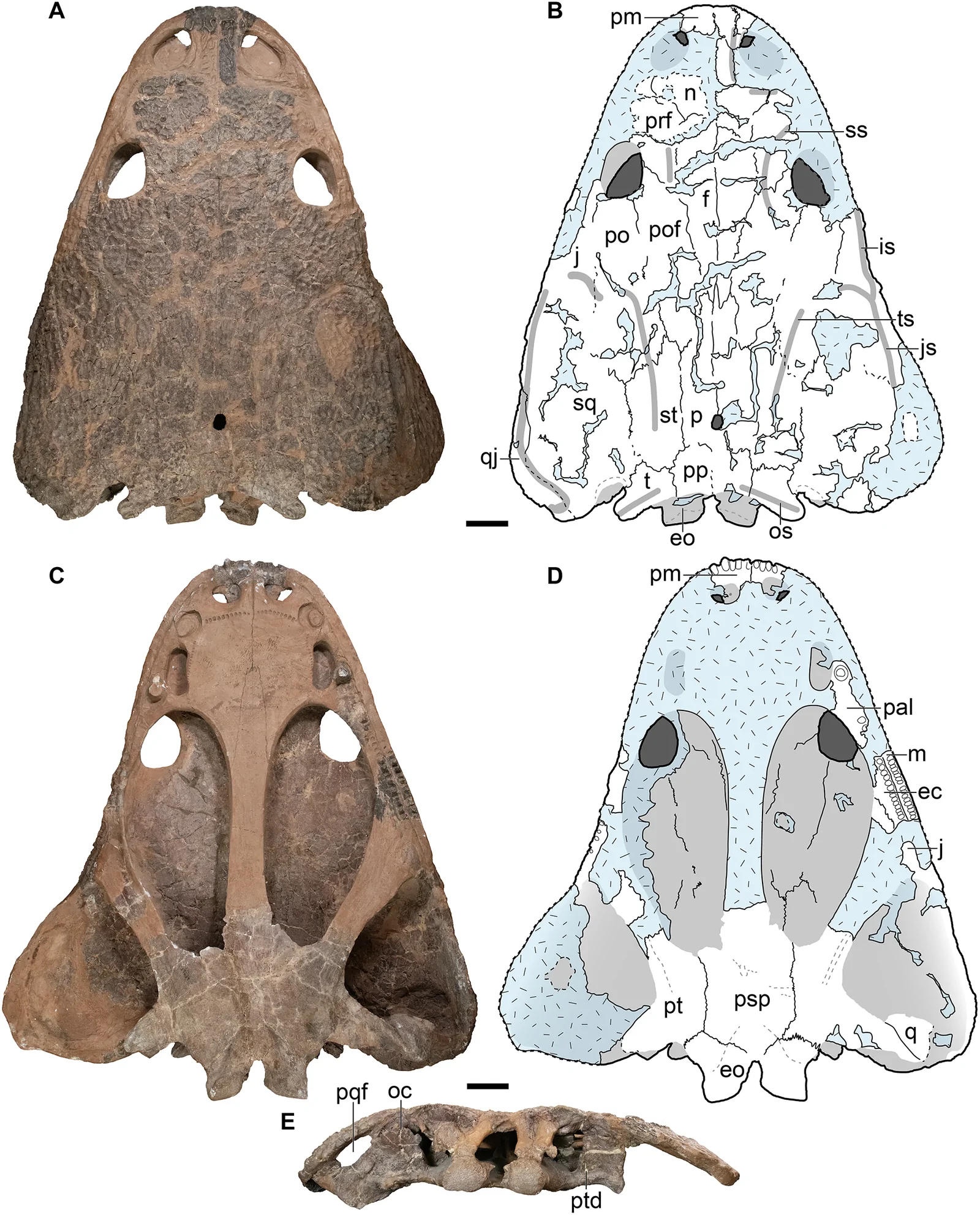
A large, partial cranium of Metoposauridae indet., UCMP 27039, from locality UCMP 7307 (Blue Hills 1) in Apache County, AZ. (A) Photograph in dorsal view; (B) interpretive drawing of the same; (C) photograph in ventral view; (D) interpretive drawing of the same; (E) photograph in occipital view. Blue hatching represents reconstruction; fine dashed gray lines represent topographic details like ridges; stippling represents residual matrix. Abbreviations: ec, ectopterygoid; eo, exoccipital; f, frontal; is, infraorbital sulcus; j, jugal; js, jugal sulcus; m, maxilla; n, nasal; oc, oblique crest of the pterygoid; p, parietal; pal, palatine; pm, premaxilla; po, postorbital; pof, postfrontal; pp, postparietal; pqf, paraquadrate foramen; prf, prefrontal; psp, parasphenoid; pt, pterygoid; ptd, pterygoid depression; q, quadrate; qj, quadratojugal; sq, squamosal; ss, supraorbital sulcus; st, supratemporal; t, tabular; ts, temporal sulcus. Upper scale bar for (A–D); lower scale bar for (E). Scale bars equal 5 cm.

UCMP 7307—. UCMP 27039 is the largest metoposaurid cranium observed in this study (midline length ≈ 60 cm) and has significant regions of reconstruction obscuring much of the morphology (Fig. 54). UCMP 27039 is from the locality Blue Hills 1 in Apache County, AZ (UCMP 7307; erroneously noted as UCMP 7707 (fig. 10b: Long & Murry, 1995)) which also yields a large cranium here referred to *Apachesaurus gregorii* (Fig. 51). Sutures of the skull roof are difficult to discern, perhaps due to preservation, but this could be due, in part, to coossification of cranial elements and partial obliteration of sutures indicating a more advanced ontogenetic stage. Regardless, the damage to the skull roof is so extensive that much of the antorbital skull is reconstructed aside from the immediate parasagittal elements along the right side (Figs. 54A, 54B). A prominent alary process of the premaxilla extends along the medial narial margin. What can be determined of the sutural configuration of the postorbital skull roof is consistent with essentially all other metoposaurids except *Metoposaurus* spp. Surface details of the skull roof such as the extent of the lateral line sulci are estimated due to extensive surficial damage, and the unusual lateral curvature of the temporal sulcus found in UCMP 27051 (Fig. 51) and the extension of the temporal sulcus onto the squamosal in the holotype and a referred skull roof of *A. gregorii* (Figs. 46, 48) could not be corroborated. A partial transverse occipital sulcus is present on the tabular and extending onto the postparietal on the right side (Figs. 54A, 54B).

Most of the palate is reconstructed, but the basicranium is relatively intact (Figs. 54C, 54D). The cultriform process is entirely reconstructed, and significant portions of the palatal rami are as well. The contact between the left pterygoid and the ectopterygoid is preserved demonstrating the absence of a posteromedial extension of the ectopterygoid. Some faint ornamentation formed by a single preserved pit and some radiating ridges can be seen on the basal plate of the parasphenoid, and posterior to this ornament, a pair of ridges extend from the basal plate onto the subotic process of the exoccipital oblique to the midline. Positionally, these ridges are similar to the “muscular crests” (*sensu* Sulej, 2007) found in most metoposaurids and other stereospondyls, but those crests are typically transverse as they extend laterally. The occiput is reasonably well-preserved (Fig. 54E), but significant portions are missing (*e.g*., right quadrate and much of the quadrate ramus of the pterygoid) or reconstructed (*e.g*., the occipital pillars). The width of the paraquadrate foramen is approximately subequal with the width of the foramen magnum, although the latter is partially reconstructed. The paraquadrate foramen is surrounded by the quadratojugal and the squamosal, and the ventral process of the squamosal contacts the quadrate. The oblique crest of the pterygoid, with a depression near its origin from the quadrate ramus, ascends dorsally and slightly posteriorly to just below the level of the tabular horn. The quadrate ramus curves ventrolaterally, but this curvature is less dramatic than that seen in the holotype of *A. gregorii* (Figs. 47A, 47B).

**Figure 55:**
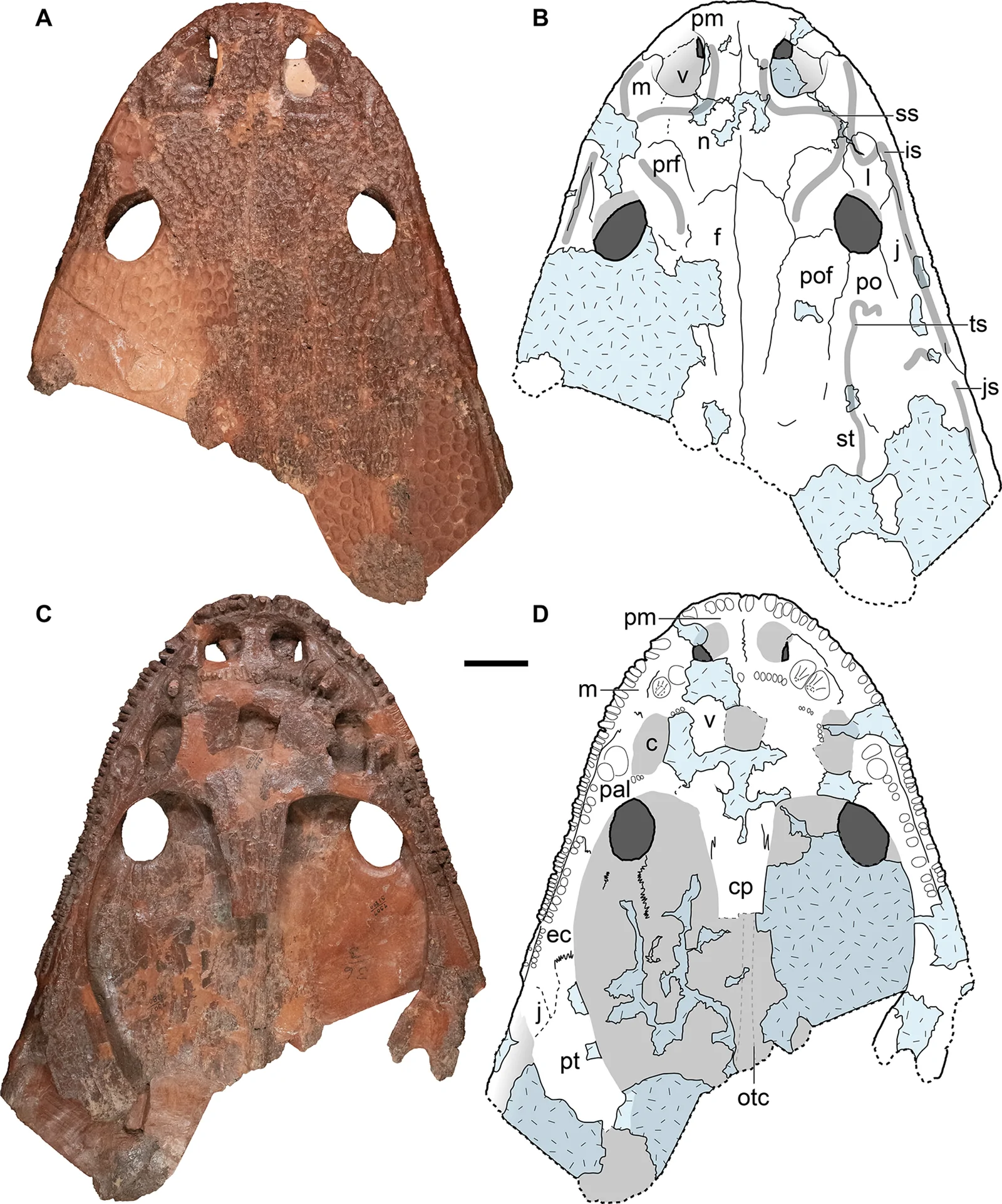
Partial anterior cranium of Metoposauridae indet., UCMP 31800, from locality UCMP 7307 (Blue Hills 1) in Apache County, AZ. (A) Photograph in dorsal view; (B) interpretive drawing of the same; (C) photograph in ventral view; (D) interpretive drawing of the same. Blue hatching represents reconstruction; diagonal lines represent broken surfaces; fine dashed gray lines represent topographic details like ridges. Abbreviations: c, choana (internal naris); cp, cultriform process of the parasphenoid; ec, ectopterygoid; f, frontal; is, infraorbital sulcus; j, jugal; js, jugal sulcus; l, lacrimal; m, maxilla; n, nasal; otc, orbitotemporal crest; pal, palatine; pm, premaxilla; po, postorbital; pof, postfrontal; prf, prefrontal; pt, pterygoid; ss, supraorbital sulcus; st, supratemporal; ts, temporal sulcus; v, vomer. Scale bar equals 5 cm.

UCMP 31800 is a partial anterior cranium encompassing much of the prepineal skull roof and portions of the palate (Fig. 55). UCMP 31800 is also from the Blue Hills 1 locality co-occurring with a large, referred specimen of *Apachesaurus gregorii* (UCMP 27051: Fig. 51). However, the relevant anatomy used to refer UCMP 27051 to *A. gregorii* including the configuration of the temporal sulcus and the relative width cultriform process are not preserved in UCMP 31800. The lacrimal forms a broad contribution to the orbital margin (Figs. 55A, 55B). The relatively broad and blunt anterior process of the prefrontal is similar to specimens of *Paranaschisma howardensis*. The choanae are relatively large more like those of *Tecovaserpeton perfectum* and *A. gregorii* (Figs. 55C, 55D). The cultriform process narrows posteriorly, but it is too fragmentary and constriction of the metoposaurid cultriform process is too variable to determine if this narrowing would be consistent throughout.

**Figure 56:**
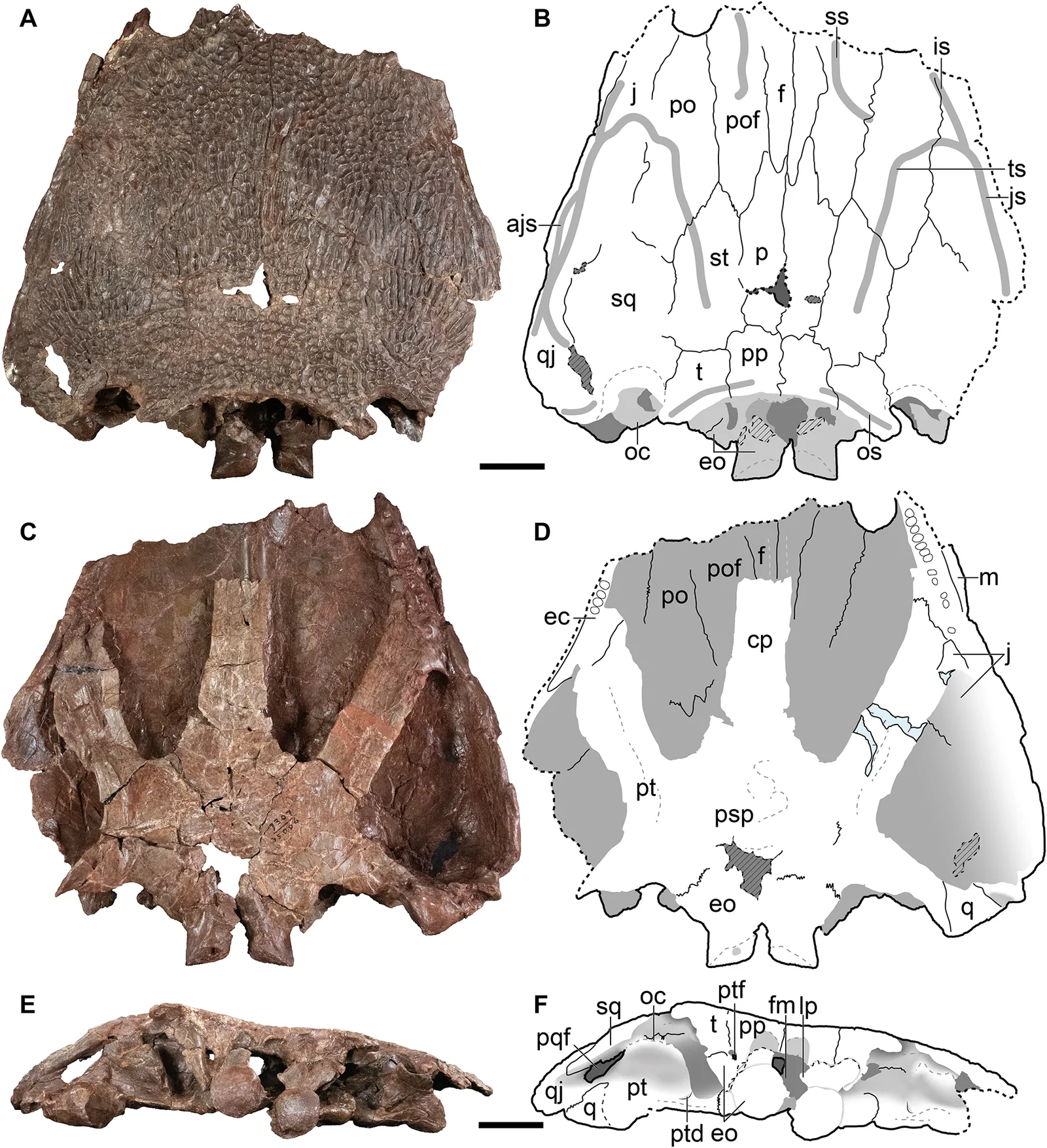
Partial posterior cranium of Metoposauridae indet., UCMP 35006, from locality UCMP 7307 (Blue Hills 1) in Apache County, AZ. (A) Photograph in dorsal view; (B) interpretive drawing of the same; (C) photograph in ventral view; (D) interpretive drawing of the same; (E) photograph in occipital view; (F) interpretive drawing of the same. Blue hatching represents reconstruction; diagonal lines represent broken surfaces; fine dashed gray lines represent topographic details like ridges; stippling represents residual matrix. Abbreviations: ajs, accessory jugal sulcus; cp, cultriform process; eo, exoccipital; f, frontal; fm, foramen magnum; is, infraorbital sulcus; j, jugal; js, jugal sulcus; lp, lamellose process; oc, oblique crest of the pterygoid; os, transverse occipital sulcus; p, parietal; po, postorbital; pof, postfrontal; pp, postparietal; pqf, paraquadrate foramen; psp, parasphenoid; pt, pterygoid; ptd, pterygoid depression; ptf, posttemporal fenestra; q, quadrate; qj, quadratojugal; sq, squamosal; ss, supraorbital sulcus; st, supratemporal; t, tabular; ts, temporal sulcus. Scale bars equal 5 cm.

UCMP 35006 preserves most of the postorbital cranium (Fig. 56). The posterior margins of both orbits are preserved, and the layout of the skull roof elements does not significantly differ from other metoposaurids presented here (Figs. 56A, 56B). The portion of the frontal that is preserved appears relatively narrow. The left parietal-supratemporal suture is subparallel to the midline, but the right parietal-supratemporal suture is slightly angled. The full complement of posterior skull roof lateral line sulci is present including an accessory jugal sulcus and an incomplete transverse occipital sulcus traversing the entire posterior margin of the tabular and part of the postparietal. In ventral view, significant portions of the posterior left palate and the basicranium are preserved (Figs. 56C, 56D). The cultriform process of the parasphenoid is wider than the minimal width of the palatal ramus of the pterygoid unlike UCMP 27051 (Figs. 51D, 51E). The *insula jugalis* is relatively short, and the ectopterygoid does not project onto the palatal ramus (Figs. 56C, 56D) unlike specimens of *P. howardensis*. The sutures around the basal plate of the parasphenoid are difficult to discern from fractures. Some reticulate ornamentation is present on the basal plate, but it does not radiate onto the cultriform process. The ventral and occipital surfaces of the quadrate are slightly worn down from preparation. There is a conspicuous circular indentation on the right occipital condyle of unknown origin. In occipital view, UCMP 35006 has typical features such as a pterygoid depression and tall oblique crest of the pterygoid. A lamellose process is present on the right exoccipital jutting into the foramen magnum as a distinct projection though not as large as that of *P. howardensis*. A small round posttemporal fenestra is visible on the left occipital pillar. The paraquadrate foramen is slightly wider than the foramen magnum, but the latter is distorted by the dorsomedially displaced left exoccipital. The ventral suture between the squamosal and the quadratojugal could not be identified prohibiting identification of contact between the squamosal and the quadrate.

**Figure 57:**
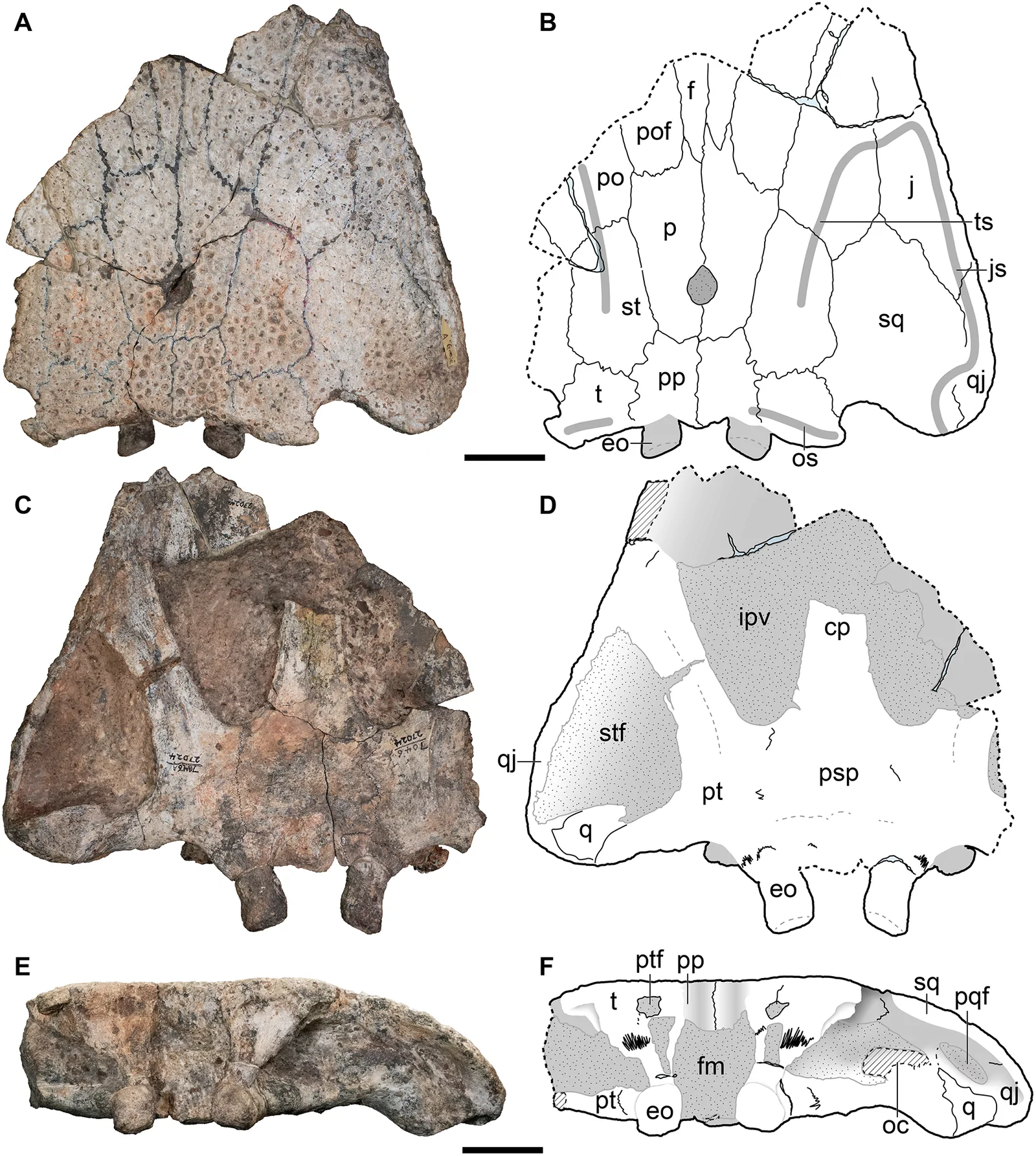
Partial posterior cranium of Metoposauridae indet., UCMP 27024 from UCMP 7046A (Point of Bluff S) in Petrified Forest National Park, AZ. (A) Photograph in dorsal view; (B) interpretive drawing of the same; (C) photograph in ventral view; (D) interpretive drawing of the same; (E) photograph in occipital view; (F) interpretive drawing of the same. Blue hatching represents reconstruction; diagonal lines represent broken surfaces; fine dashed gray lines represent topographic details like ridges; stippling represents residual matrix. Abbreviations: cp, cultriform process; eo, exoccipital; f, frontal; fm, foramen magnum; ipv, interpterygoid vacuity; j, jugal; js, jugal sulcus; oc, oblique crest of the pterygoid (broken); os, transverse occipital sulcus; p, parietal; po, postorbital; pof, postfrontal; pp, postparietal; pqf, paraquadrate foramen; psp, parasphenoid; pt, pterygoid; ptf, posttemporal fenestra; q, quadrate; qj, quadratojugal; sq, squamosal; st, supratemporal; stf, subtemporal fenestra; t, tabular; ts, temporal sulcus. Scale bars equal 5 cm.

UCMP 7046A—. UCMP 27024 is a partial posterior skull roof, palate, and braincase from site UCMP 7046A Adamana 14 (aka Point of Bluff S: Long & Murry, 1995) situated in the lower Petrified Forest Member of the Chinle Formation in Petrified Forest National Park, AZ. The dorsal surface of the skull roof is well-prepared and well-preserved, but there appears to be damage (either from preservation or from preparation) on the ventral surface of the palate inhibiting the assessment of surface-level details such as ornamentation on the basal plate (Fig. 57). Damage to the anterolateral portion of the skull roof prevents the confident identification of an infraorbital sulcus, but the jugal and temporal sulci are present forming a joined postorbital sulcus with no accessory jugal sulcus. The presence of a partial transverse occipital sulcus on the tabular and partially on the postparietal rules out the referral of UCMP 27024 to either *Anaschisma browni* or *Buettnererpeton bakeri*, and the absence of an accessory jugal sulcus precludes referral to *Paranaschisma howardensis* or *Tecovaserpeton perfectum*. This specimen is unusual among North American metoposaurids in the presence of a very wide parietal that forms a broad contact with the postorbital, a state seen in some examples of *Metoposaurus krasiejowensis* (figs. 12, 15: Sulej, 2007) although most of the figured specimens of *M*. *krasiejowensis* have either a point contact or no contact. The postparietals form a prominent bulge on their posterior margin in dorsal view (Figs. 57A, 57B). The apex of the tabular horn is directed approximately laterally unlike the posterolateral inclination of the tabular horn of most metoposaurids. The occipital condyles are very narrow and widely separated, but there is some damage along the medial margin of both condyles making their total mediolateral width uncertain although they appear nearly complete (Fig. 57). The lamellose process of the exoccipital is entirely absent with smooth margins bordering the foramen magnum along its lateral borders (Figs. 57E, 57F). Ventral to the posttemporal fenestra, a matrix-filled fossa extends along much of the length of the vertical process of the exoccipital. A similar fossa bearing a foramen in the ventral part of the fossa is present in *Metoposaurus algarvensis* (fig. 6: Brusatte et al., 2015), however, the fossa in *M. algarvensis* also surrounds the posttemporal fenestra unlike the condition in UCMP 27024. The paraquadrate foramen is small compared to similarly sized metoposaurids, but its relative size is slightly larger than that seen in the holotype of *Apachesaurus gregorii* (Figs. 47A, 47B, 57E, 57F).

Stereospondyli indet.

Several specimens have been referred to the Metoposauridae or alpha taxa therein over the last century including several holotypes now typically considered *nomina dubia*. The earliest known specimen sometimes considered a metoposaurid is AMNH 5661 *Dictyocephalus elegans* Leidy, 1856. *Dictyocephalus elegans* has invariably been considered a *nomen dubium* for decades often not even being considered in taxonomic revisions because it lacks definitive apomorphies of the Metoposauridae (Buffa, Jalil & Steyer, 2019; Gee, Parker & Marsh, 2020; Gee & Kufner, 2022; Hunt, 1993; Zanno et al., 2002; this study), but some earlier authors considered *D. elegans* a valid metoposaurid but distinct from other metoposaurids based on a shallow otic notch and diminutive tabular horn (Colbert & Imbrie, 1956; Davidow-Henry, 1989). Another specimen typically considered non-diagnostic below the family level is AMNH 3927 “*Mastodonsaurus*” *durus* Cope, 1866 later reassigned to *Eupelor durus* (Cope, 1868) from the Phoenixville Tunnel in Pennsylvania. The validity of *Eupelor durus* has fluctuated with some authors considering the genus a valid metoposaurid (Colbert & Imbrie, 1956) and more recent authors considering it non-diagnostic below the family level (Baird, 1986; Hunt, 1993). The type material was restricted to purported “labyrinthodont” cranial material (Cope, 1868), but (Colbert & Imbrie, 1956) could not identify the cranial material to which Cope was referring in their study of the Pennsylvania material. Without published figures or even identification of the type material of *Eupelor durus*, only firsthand observation can verify these claims.

## Results

### Specimen-level phylogeny

**Figure 58:**
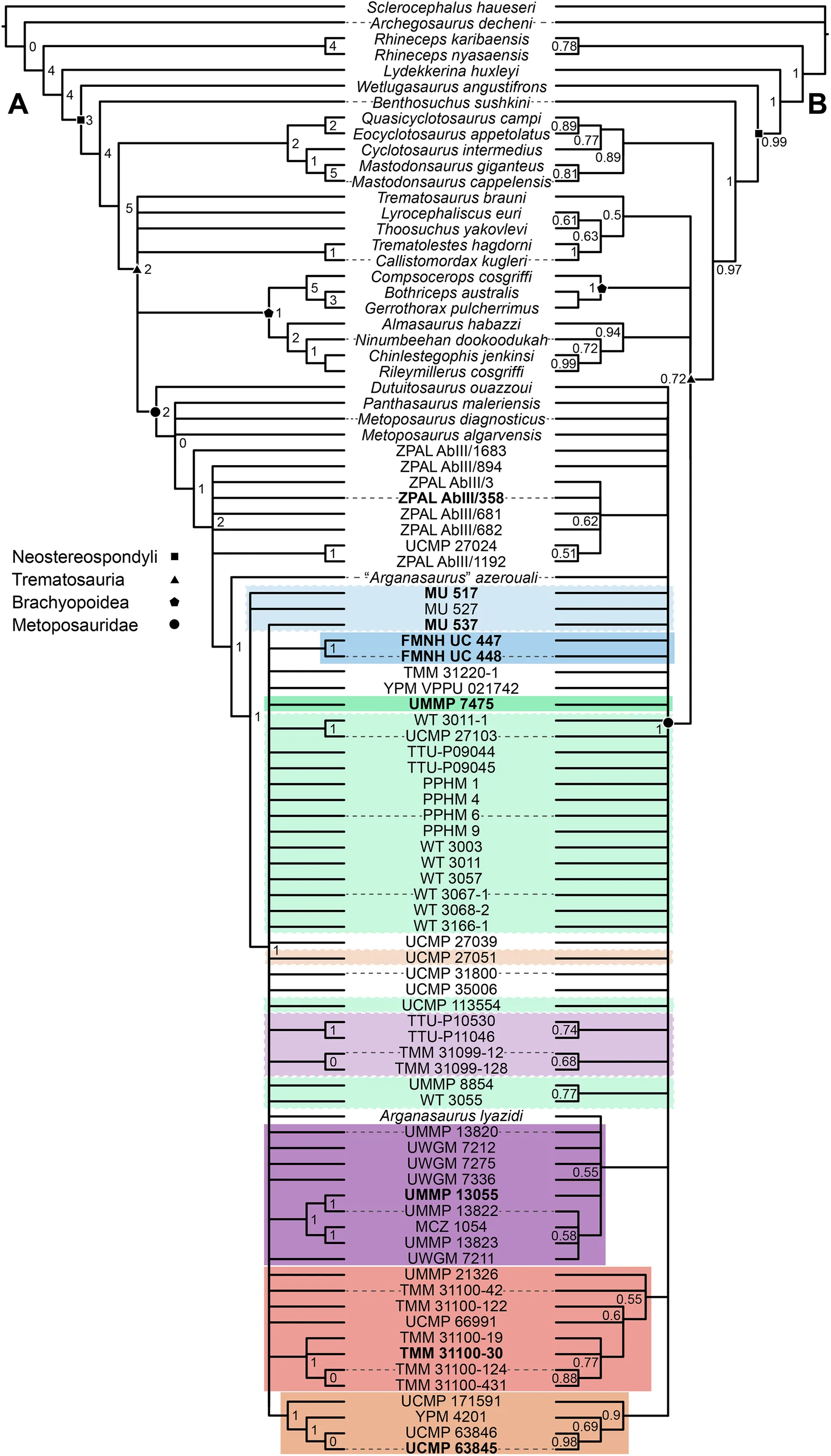
Comparison of trees resulting from the character matrix including all specimens and removing only dubious characters. (A) Strict (Nelson) consensus tree of 1,440 trees (895 steps; CI = 0.195; RI = 0.514); (B) Bayesian summary tree with nodes less than 0.5 posterior probability collapsed. Shapes indicating nodes: square, Neosterospondyli; triangle, Trematosauria; pentagon, Brachyopoidea; circle, Metoposauridae. Colors indicating specific referrals: blue = *Anaschisma browni*; red = *Paranaschisma howardensis*; purple = *Buettnererpeton bakeri*; orange = *Apachesaurus gregorii*; green = *Tecovaserpeton perfectum*; darker shades indicate monophyly or near monophyly including the holotype of a given species and excluding the type or referred material of others; these colors correspond to taxa as indicated in Fig. 1.

Fully bifurcated summary trees from MrBayes with consensus type set to “allcompat” (*i.e*., all clades compatible with the “halfcompat” tree (=majority-rule consensus) and with one another) and complete strict (Nelson) consensus trees from TNT are provided in File S1 (Figs. S5–S8). The topologies of the trees recovered were consistent with that found by Gee & Kufner (2022) with monophyletic Metoposauridae typically recovered in a polytomy with brachyopoids, trematosaurids, and with ‘latiscopids’ recovered in variable positions within this clade (*e.g*., Fig. 58). While resolving higher level relationships of the Stereospondyli and the Metoposauridae was not the impetus for this study, the recovery of tree topologies broadly consistent with previous hypotheses of stereospondyl relationships underlines an emerging consensus (*e.g*., Eltink, Schoch & Langer, 2019; Schoch, 2013; So et al., 2024). A well-supported (posterior probability (PP) ≥ 0.93; Bremer decay index (BDI) ≥ 4) Neostereospondyli was consistently recovered (most inclusive clade containing *Mastodonsaurus giganteus*, *Buettnererpeton bakeri*, and *Trematolestes hagdorni* but not *Lydekkerina huxleyi sensu* Eltink, Schoch & Langer, 2019), and within Stereospondyli, Trematosauria was consistently recovered as a monophyletic clade albeit with unstable relationships of the constituent clades (*i.e*., Trematosauridae, Metoposauridae, and Brachyopoidea). The two pairs of non-metoposaurid congeneric species were consistently recovered as sister taxa in all analyses with good nodal support (Fig. 58, Figs. S5–S8: *Rhineceps nyasaensis* + *R. karibaensis*, *Mastodonsaurus giganteus* + *M. cappelensis*). In all analyses, Metoposauridae was recovered as a monophyletic clade with strong support in the Bayesian analyses and moderate to strong support in the parsimony analyses (PP = 1; BDI ≥ 1).

**Figure 59:**
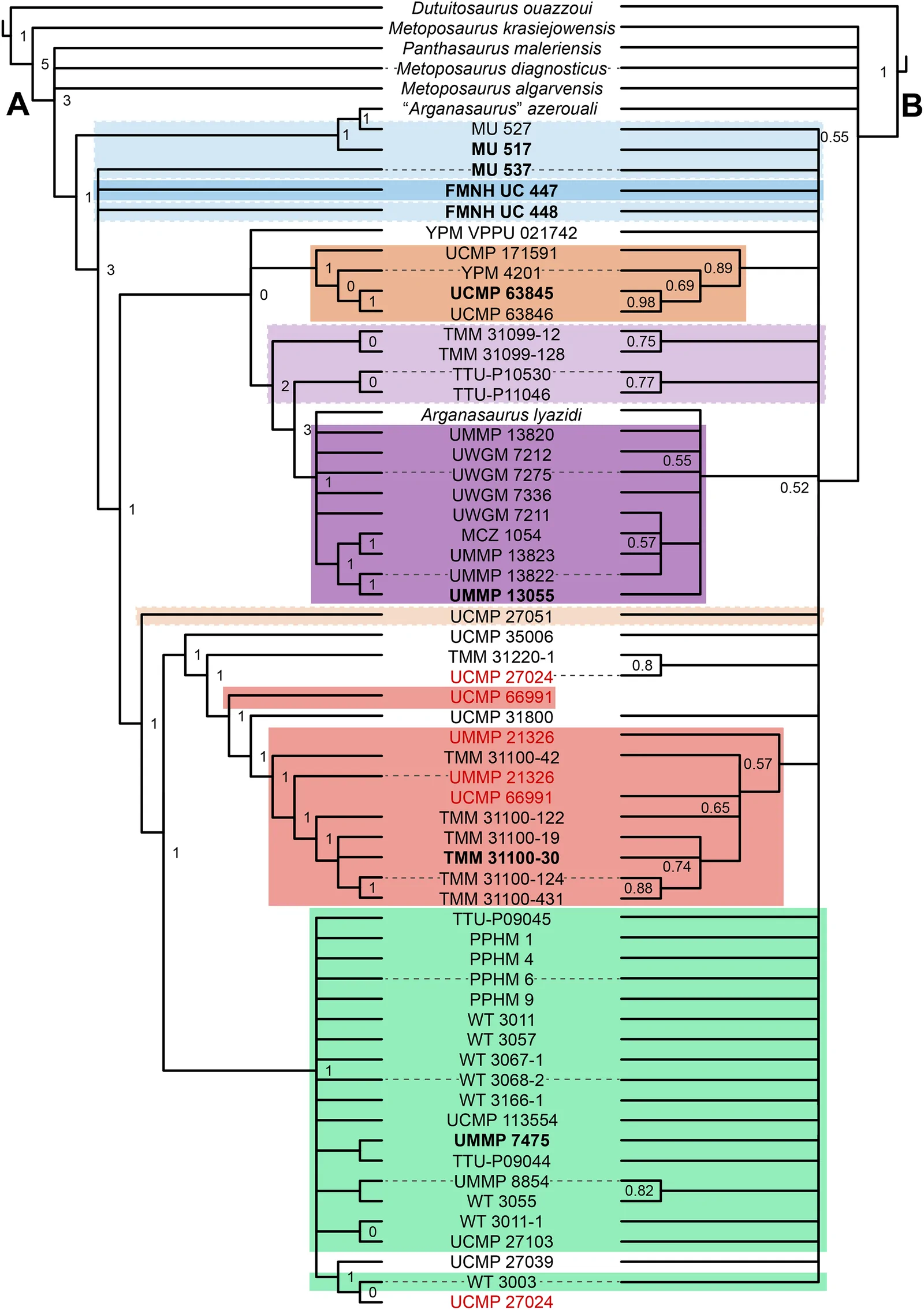
Comparison of trees focused on the Metoposauridae resulting from the character matrix excluding European specimens and removing only dubious characters. (A) Strict (Nelson) consensus tree of 1,281 trees (872 steps; CI = 0.246; RI = 0.624); (B) Bayesian summary tree with nodes less than 0.5 posterior probability collapsed. OTU labels in red have more divergent positions between analyses that could not be aligned by simple transformations to the tree (*i.e*., without changing the topology). Colors correspond to taxa as indicated in Figs. 1 and 58.

**Figure 60:**
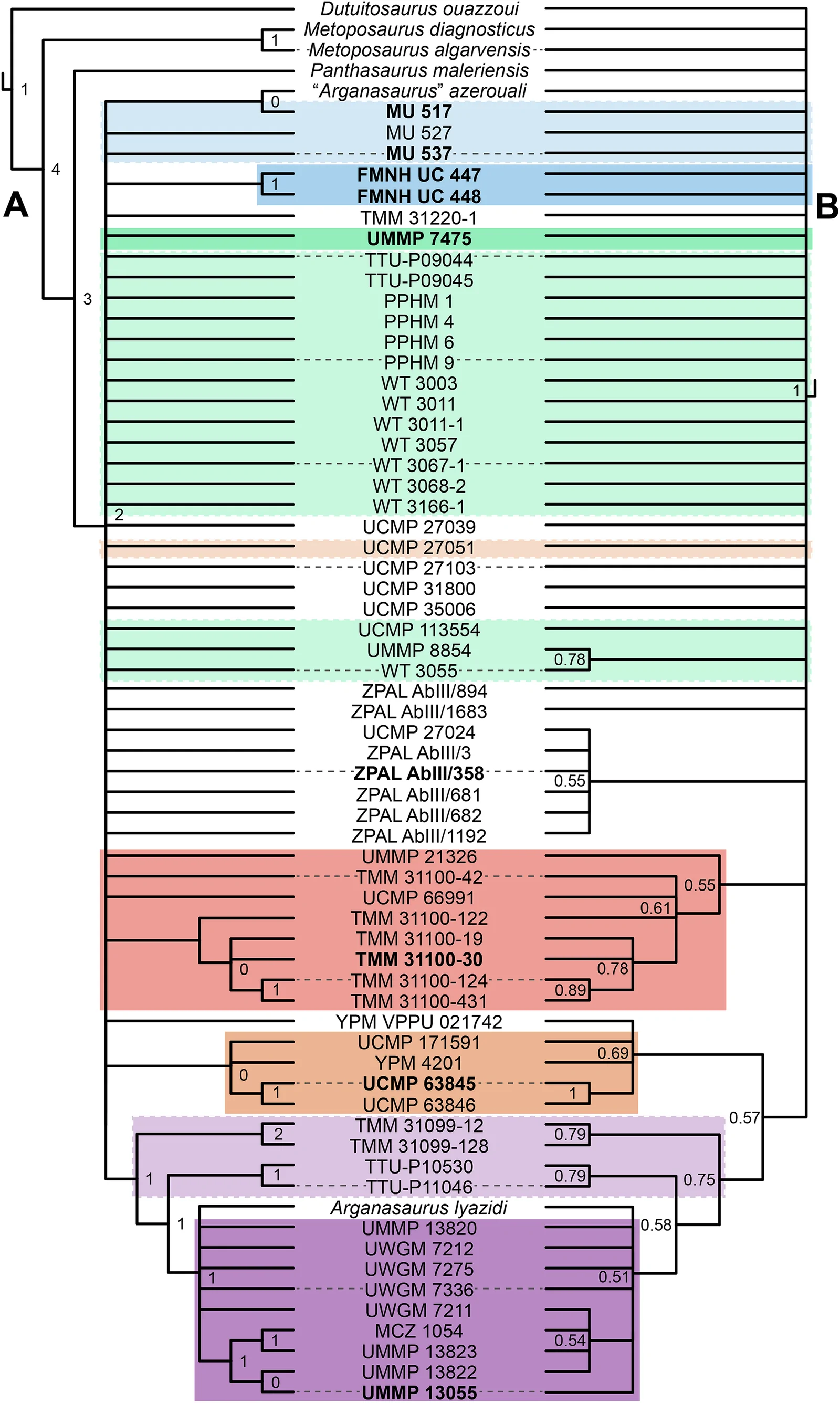
Comparison of trees focused on the Metoposauridae resulting from the character matrix including all specimens and removing dubious and putative ontogenetically variable characters. (A) Strict (Nelson) consensus tree of 831 trees (849 steps; CI = 0.203; RI = 0.533); (B) Bayesian summary tree with nodes less than 0.5 posterior probability collapsed. Colors correspond to taxa as indicated in Figs. 1 and 58.

**Figure 61:**
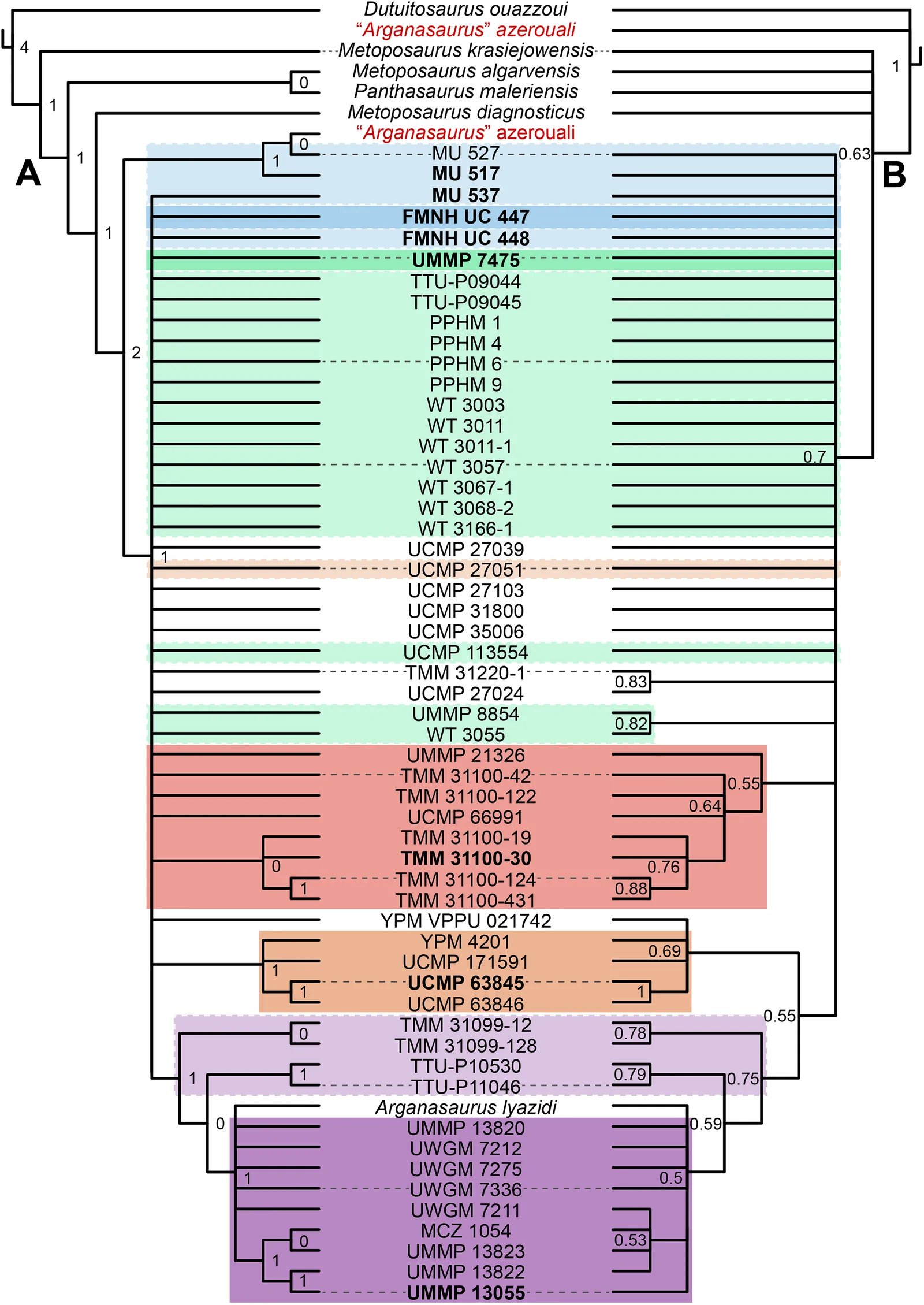
Comparison of trees focused on the Metoposauridae resulting from the character matrix excluding European specimens and removing dubious and putative ontogenetically variable characters. (A) Strict (Nelson) consensus tree of 784 trees (829 steps; CI = 0.23; RI = 0.585); (B) Bayesian summary tree with nodes less than 0.5 posterior probability collapsed. OTU labels in red have more divergent positions between analyses that could not be aligned by simple transformations to the tree (*i.e*., without changing the topology). Colors correspond to taxa as indicated in Figs. 1 and 58.

The internal relationships of metoposaurids have historically been poorly resolved (see Gee & Kufner, 2022), but some patterns have begun to emerge in the present analyses that agree with some previous studies. *Dutuitosaurus* is frequently recovered as the earliest diverging metoposaurid in the present analyses, a finding consistent with one previous computer-assisted phylogenetic analysis of metoposaurids (Buffa, Jalil & Steyer, 2019) and a non-computer-assisted analysis of metoposaurid relationships finding *Dutuitosaurus* sister to a clade of *Metoposaurus* + “*Buettneria*” (Hunt, 1993). In some of the present analyses, most of the metoposaurids found outside North America are recovered in a polytomy outside a clade of North America + Morocco (excluding *Dutuitosaurus*) or in a polytomy of all metoposaurids (Figs. 58–61). In all iterations of this matrix except the analyses including all specimens and ontogenetically variable characters (Fig. 58) and as would often be expected, the Bayesian analyses recovered less resolved internal relationships of the Metoposauridae than the parsimony analyses (see O’Reilly et al., 2016, 2018).

A monophyletic *Metoposaurus* was only recovered in the “allcompat” Bayesian trees including putative ontogenetically variable characters (Figs. S5 and S6). *Panthasaurus maleriensis* from the Indian subcontinent was commonly recovered among *Metoposaurus* spp. as suggested in some previous studies (Buffa, Jalil & Steyer, 2019; Chakravorti & Sengupta, 2019) or in a polytomy of all metoposaurids with no close relationship with *Tecovaserpeton perfectum* differing from some earlier interpretations of *P. maleriensis* (*e.g*., RoyChowdhury, 1965; Sengupta, 2002). “*Arganasaurus*” *azerouali* was never recovered with the type species of the genus, *Arganasaurus lyazidi*, but it was recovered either in a polytomy of all or most metoposaurids or among the specimens of *Anaschisma browni*.

*Buettnererpeton bakeri* and *Ar. lyazidi* were consistently recovered in a well-supported clade with *Ar. lyazidi* typically found in a polytomy with specimens of *B. bakeri* from the type locality in Snyder County, TX EP and NK in Fremont County, WY. The referred specimens from Otis Chalk Quarry 2 and the Boren Quarry were recovered in either a polytomy of North American metoposaurid specimens (Figs. 58, 59B) or in a weakly supported clade with the EP and NK specimens of *B. bakeri* (Figs. 59A, 60, 61). A core clade of *Apachesaurus gregorii* consisting of the small-bodied type locality material and a previously referred juvenile specimen (UCMP 171591) was consistently recovered as monophyletic in all analyses. In some of the analyses, *A. gregorii* was found as the sister taxon of *B. bakeri* forming a clade of North American specimens without a lacrimal contribution to the orbital margin (Figs. 59A, 60B, 61B). UCMP 27051, referred to *A. gregorii* by a distinct lateral curvature at the posterior end of the temporal sulcus toward the squamosal and a very narrow cultriform process, was not recovered with this core clade of *A. gregorii* (see Discussion for further rationale to uphold this referral). In one parsimony analysis excluding specimens of *M. krasiejowensis*, UCMP 27051 was recovered as the sister taxon of a clade consisting of *T. perfectum* and *Paranaschisma howardensis* (Fig. 59A), but otherwise, UCMP 27051 was recovered in a polytomy of North American metoposaurids. A core clade of four specimens from Otis Chalk Quarry 3 including the lectotype of *P*. *howardensis* (TMM 31100-30) was recovered as a reasonably well-supported monophyletic clade in most analyses (PP > 0.7; BDI = 0–1) with additional specimens from Otis Chalk Quarry 3 and UMMP 21326 sometimes forming a grade outside of this core. In the parsimony analysis including ontogenetically variable characters with a species-level OTU of *M. krasiejowensis*, several indeterminate specimens form a grade at the base of the clade of *P. howardensis* specimens (Fig. 59A).

The least well-supported species proposed here are *An. browni* and *T*. *perfectum*. FMNH UC 447 (holotype of *An. browni*) and FMNH UC 448 form a clade in the parsimony analysis including ontogenetically variable characters and specimen-level OTUs of *M. krasiejowensis* exclusive of non-*Anaschisma* OTUs (Fig. 58A). However, a monophyletic clade comprising all specimens of *An. browni* (FMNH UC 447 and 448; MU 517, 527, and 537) was not recovered in any analysis, either forming a successive grade outside of the remainder of North America + *Ar. lyazidi* clade in the parsimony analyses with a species-level OTU of *M. krasiejowensis* (Figs. 59A, 61A) or recovered in a polytomy with all or most metoposaurids (Figs. 58B, 59B, 60, 61B). A close relationship of MU 517 and MU 527 with “*Ar*.” *azerouali* was recovered in some parsimony analyses (Figs. 59A, 60A, 61A), but the position of “*Ar*.” *azerouali* is highly unstable (*e.g*., Fig. 61). Regardless, a close relationship between *An. browni sensu stricto* and previously referred specimens now assigned to *P. howardensis* and *T. perfectum* exclusive of the generally recognized species *A. gregorii* and *B. bakeri* in distinct genera is not supported. The specimens comprising *T. perfectum* are recovered in either a weakly-supported clade in the parsimony analysis removing only dubious characters and with a species-level OTU of *M. krasiejowensis* (Fig. 59A) or in a polytomy with all North American metoposaurids in the Bayesian analyses (Figs. 58B, 59B, 60B, 61B) and the remaining parsimony analyses (Figs. 58A, 60A, 61A).

The Bayesian analyses with specimens of *Metoposaurus krasiejowensis* always recovered a polytomy of all species-level metoposaurid OTUs with some specimen-level OTUs recovered in clades corresponding to their specific referrals, including the specimens of *M. krasiejowensis* (Figs. 58B, 60B). Only two specimens of *M. krasiejowensis* were not recovered in this clade: ZPAL AbIII/1683 and ZPAL AbIII/894. ZPAL AbIII/1683 is one of the smallest skulls described by Sulej (fig. 4: 2007), and ZPAL AbIII/894 (fig. 8, 16d: Sulej, 2007) is slightly larger, but both specimens exhibit at least one reduced angle of the parietal-supratemporal suture relative to the midline unlike the steeper angle in all other specimens of *M. krasiejowensis*. In each of the Bayesian analyses with specimen-level OTUs of *M. krasiejowensis*, UCMP 27024 is recovered in a clade with *M. krasiejowensis*, and like those specimens of *M. krasiejowensis*, UCMP 27024 has a widely angled parietal-supratemporal suture, so wide in fact, that the parietal forms a broad sutured contact with the postorbital as in some specimens of *M. krasiejowensis* (*e.g*., figs. 12, 15: Sulej, 2007). This could be suggestive of either the presence of *M. krasiejowensis* in North America, a heretofore unknown adult variant of coeval metoposaurids (*Apachesaurus gregorii* or *T. perfectum*), or a novel species. Due to the fragmentary nature of the skull, UCMP 27024 is referred to Metoposauridae indet. until additional material can complement the morphology of this specimen.

Removal of putative ontogenetically variable characters resulted in little change to the topology between the trees with different sets of metoposaurid OTUs (Figs. 60, 61). Only two appreciable differences are noted: in both Bayesian and parsimony analyses, removal of ontogenetically variable characters resulted in more specimens included in the *Buettnererpeton bakeri* clade (like the parsimony analysis with the species-level OTU of *M. krasiejowensis*: Fig. 59A) but with slightly lower support in the parsimony analyses. Notably, removal of ontogenetically variable characters did not recover a clade encompassing UCMP 27051 and the core clade of *A. gregorii*.

Some specimens were consistently recovered in clades but are not referred to the species of these clades due to their fragmentary nature. For example, UCMP 27039 was recovered with *T. perfectum* in one parsimony analysis (Fig. 59A) but is too fragmentary for confident referral. Similarly, UCMP 31800, 35006, and TMM 31220-1 were recovered with specimens of *P. howardensis* in the same parsimony analysis (Fig. 59A) but are all too fragmentary to be referred to any species described here. YPM VPPU 021742 has historically been referred to *Buettnererpeton bakeri* (Baird, 1986; Gee & Kufner, 2022), but support for this referral was not found in any analysis likely owing to the incompleteness of the specimen which is known only from a partial mold of the dorsal surface of the cranium. In fact, YPM VPPU 021742 was recovered with specimens of *A. gregorii* in the Bayesian analyses excluding possible ontogenetically variable characters such as the presence of a tabular horn typically used to differentiate this specimen from *A. gregorii* (Figs. 60B, 61B), but here it is considered too incomplete for referral.

## Discussion

### Implications of this specimen-level phylogeny of North American metoposaurids

This is the most comprehensive phylogenetic analysis of metoposaurid specimens to date and suggests that the long-standing synonymy of latest Carnian–Norian-aged North American metoposaurids with a lacrimal contribution to the orbital margin (*Anaschisma browni*, *Paranaschisma howardensis*, and *Tecovaserpeton perfectum*) should be reconsidered. The chronostratigraphically oldest taxon *Buettnererpeton bakeri* is found to be the most geographically widespread metoposaurid even excluding the previously referred partial skull roof mold from Nova Scotia (YPM VPPU 021742). The monophyly of at least some specimens in the species *B. bakeri*, *P. howardensis*, and *Apachesaurus gregorii* is supported in all analyses presented. Each of these taxa can be readily diagnosed exclusively from cranial specimens with apomorphies in the case of *A. gregorii* and *P. howardensis* and with a robust differential diagnosis in the case of *B. bakeri*. The remaining taxa—*An. browni* and *T. perfectum—*are supported by phenetic diagnoses, but monophyly of their constituent specimens is either non-existent in the case of the former or unsupported to weakly supported in the case of the latter (Figs. 58–61). Regardless, there is not a monophyletic grouping recovered in any of the analyses that would both support the synonymy of *An. browni*, *P. howardensis*, and *T. perfectum* and also exclude the genera *Apachesaurus*, *Arganasaurus*, or *Buettnererpeton*.

Sawin (p. 393: 1945) noted the presence of possible specimens of “*Buettneria*” *bakeri* (=*Buettnererpeton bakeri*) from Otis Chalk Quarry 2. In addition, Sawin (1944) and some subsequent authors (*e.g*., Long & Murry, 1995) have suggested that Quarry 2 is lower in section than Quarry 3 where the most abundant remains of *Paranaschisma howardensis* were collected. Most authors have considered TMM 31099-128 (Otis Chalk Quarry 2) to be a juvenile specimen of *Anaschisma browni* or junior synonyms therein (Gee & Kufner, 2022; Gee & Parker, 2020; Hunt, 1993; Martz, 2008). However, Chakravorti and Sengupta (fig. 1: 2019) referred this specimen to “*Koskinonodon*” *bakeri* (=*Buettnererpeton bakeri*) after observation of a cast (AB8948: Natural History Museum of London), erroneously considered to be a cast of UMMP (VP) 13055, the holotype of *B. bakeri* from the Elkins Place bonebed. Several shared features support the referral of the Otis Chalk Quarry 2 material to *B. bakeri*, most notably the lacrimal excluded from the orbital margin, the wide cultriform process, and the presence of a sensory groove on the clavicle. Interestingly, referred specimens of *B. bakeri* from Otis Chalk Quarry 2 and the Boren Quarry were not consistently recovered in a clade with the EP (type locality) and NK specimens (see Figs. 58, 59). While these specimens fall under the phenetic diagnosis of *B. bakeri*, some details of their anatomy not considered in the present analyses such as the downturned premaxilla (shared with *An. browni*) or differences in the undescribed postcranial material may serve to differentiate these specimens from *B. bakeri*.

The close relationship of *Buettnererpeton bakeri* and *Arganasaurus lyazidi* with weak to moderate support (PP ≥ 0.5; BDI ≥ 0) in most analyses suggests that the dispersal of metoposaurids across western Laurasia may have originated in northern Africa or southern Europe. The Irohalene Member (T5) of the Timezgadiouine Formation is typically considered Carnian in age, and the type locality of *Ar. lyazidi* (Site 19 of Dutuit) is near the top of the Irohalene Member (Dutuit, 1976b; Jalil, 1999; Tourani et al., 2023). A modeled depositional age of ~231–229 Ma for the “purple unit” of the Popo Agie Formation was estimated based on radioisotopic ages from the overlying “ochre unit” (Lovelace et al., 2025) constraining the age of referred specimens of *B. bakeri* from the NK locality. Notably, the timing of dispersal may coincide with the Carnian Pluvial Episode (Jalil, 1999; Tourani et al., 2023; Zouheir et al., 2023). The near contemporaneity and similarity of *Ar. lyazidi* and *B. bakeri* suggests the need for a redescription and revision of *Ar. lyazidi* to determine whether these species may be synonymous.

The referral of UCMP 27051 to *Apachesaurus gregorii* was not supported by monophyly in the phylogenetic analyses, but UCMP 27051 shares an apomorphic temporal sulcus extending to the squamosal with the type locality specimens of *A. gregorii*—a character not found in any other metoposaurids or any other stereospondyls included in this study. The phylogenetic placement of UCMP 27051 may be explained by a suite of other features such as the well-developed tabular horn and presence of all cranial lateral line sulci shared by most other large-bodied North American metoposaurids that could be indicative of a more advanced ontogenetic stage. UCMP 27051 is much larger than other specimens of *A. gregorii*, approaching almost three times the midline length of the skull roof of the holotype UCMP 63845. Without unambiguous specimens of *A. gregorii* spanning this size, a description of the acquisition of adult characters in *A. gregorii* remains elusive.

The referral of UCMP 27051 to *Apachesaurus gregorii* suggests an intriguing possibility for the ontogeny of *A. gregorii*. The lacrimal enters the orbital margin of UCMP 27051, unlike all other specimens of *A. gregorii*, leaving a few options to explain this discrepancy: (1) the referral of UCMP 27051 to *A. gregorii* based on other characters was made in error; (2) like *Metoposaurus krasiejowensis*, intraspecific variation in the lacrimal contribution to the orbital margin is present in *A. gregorii*; or (3) remodeling of the circumorbital elements during ontogeny results in a greater or lesser contribution of the lacrimal to the orbital margin when early in ontogeny there is no contact. Without a larger sample of similarly sized specimens with the apomorphies of *A. gregorii*, the second option cannot currently be tested. However, it is not without precedent, for example, the proportion of the orbital margin formed by the lacrimal is variable in *Tecovaserpeton perfectum* (*e.g*., Figs. 36, 37). Indeed, this may also inform option three if the development of the lacrimal proceeded through stages of no contact to narrow contact to broad contact. There is also evidence for extensive remodeling of the preorbital skull roof in *M. krasiejowensis*, and the growth of the dorsal surface of the posterior skull roof elements exhibits an alternating pattern of ridges and grooves in successive ‘generations’ of growth (Gruntmejer, Konietzko-Meier & Bodzioch, 2016). Previous work on the histology of the skull of metoposaurids is limited, and individual elements were sampled to delineate the distribution of remodeling, vascularization, and response to modeled stress regimes (Gruntmejer, Konietzko-Meier & Bodzioch, 2016; Konietzko-Meier et al., 2018). Histological study of the skull roof sutures similar to previous work on the mandible of *M. krasiejowensis* (Gruntmejer et al., 2018; Gruntmejer, Bodzioch & Konietzko-Meier, 2021) may reveal whether the circumorbital region of the skull roof in metoposaurids possessing a lacrimal contribution to the orbital margin exhibits growth patterns that may explain a shift in the position of the lacrimal relative to the orbital margin.

The novel taxonomic arrangement proposed here based on previously named species could indicate a more endemic distribution of metoposaurids in the late Carnian–Norian of North America than previously recognized. However, the diachronous nature of the occurrences of these taxa or poorly resolved correlation of the Chugwater Group and lower Dockum Group leave ample room for competing hypotheses on the origin and evolution of North American metoposaurids. The phylogenetic relationships found in this study and geographic distribution of these taxa offer little evidence for hypotheses regarding their dispersal or persistence across North America be that *via* vicariance from locations such as modern-day Morocco or Europe, long-term anagenesis, cladogenesis, or some combination thereof.

It should also be noted that recovery of ontogenetically ordered clades in cladistic ontogeny is highly sensitive to character and outgroup selection (Napoli, 2026). While a cladistic analysis of the ontogeny (*e.g*., Brochu, 1996; Carr, 2020; Napoli, 2026; Zietlow, 2020) of metoposaurids was not the goal of this study, the failure of some cladistic ontogenies to recover taxonomically valid clades may explain some of the non-monophyletic clades of referred specimens in the present analyses. In addition, the ontogenetic sequence of metoposaurids and variability of morphological characters therein are very poorly constrained. The outgroup taxa (non-metoposaurids) included in this study were scored almost exclusively based on adult specimens with the exceptions of *Benthosuchus sushkini*, *Trematolestes hagdorni*, and *Trematosaurus brauni* for which scores from juvenile specimens were incorporated as polymorphisms of their respective species rather than OTUs separate from the adults.

### Excluded characters

95—. Case (fig. 3: 1922) depicted a possible opisthotic in UMMP 7475, the holotype of “*Buettneria*” *perfecta* (=*Tecovaserpeton perfectum*). The view presented is a ventral view of the skull roof with the palate and basicranium removed, a view which is now inaccessible due to reattachment of the two parts of the specimen (see Fig. 36). However, the element depicted is more likely a damaged parotic process of the tabular with fractures near the connection with the roofing portion of the tabular appearing similar to sutures. Case’s placement of the opisthotic in UMMP 7475 aligns with the position of this element in most non-stereospondyl temnospondyls (*e.g*., figs. 5e–f: Eltink, Schoch & Langer, 2019) in which the opisthotic forms an integral part of the occipital pillar (*sensu* Brusatte et al., 2015), and there is no contact between the vertical process of the exoccipital and the parotic process of the tabular. Due to the contact of the exoccipital and the tabular, the opisthotic (or otic complex when coossified with the prootic or otherwise indistinguishable) is “displaced,” being situated anterior to the occipital pillar at the level of the vertical process of the exoccipital in several stereospondyls (*e.g*., fig. 26: Dutuit, 1976b; figs. 19–20: Schoch, 1999; fig. 12: Witzmann et al., 2012). Despite several additional descriptions of the metoposaurid braincase or basicranial region, no ossified opisthotic has been identified outside of *Dutuitosaurus* (Branson & Mehl, 1929; Case, 1931; Sulej, 2007). Whether the widespread absence of an opisthotic is due to poor or late ossification of braincase elements, taphonomic loss owing to weak attachment, or inaccessibility of this region in many specimens outside of more expensive methods such as X-ray computed tomography, the presence or absence of an opisthotic cannot be properly assessed at present and was excluded from the phylogenetic analyses.

114—. The position of the chorda tympanic foramen (foramen for the chorda tympani nerve or internal mandibular branch of the facial nerve CN VII) has long been recognized as informative for assigning mandibular fragments of temnospondyls to higher taxonomic ranks (Jupp & Warren, 1986). The homology of the chorda tympani nerve of amniotes and the internal mandibular branch of the facial nerve of actinopterygians was originally suggested based on similar developmental origin (Goodrich, 1915) and later supported by similar chemosensory function (Kiyohara, Shiratani & Yamashita, 1985). The internal mandibular branch of the facial nerve (VII) enters the mandible through a foramen that is within or near endochondral ossifications formed by Meckel’s cartilage in the region of the glenoid fossa, and the canal runs between Meckel’s cartilage and the dermatocranial elements on the lingual surface of the mandible potentially forming a groove on Meckel’s cartilage (*e.g*., Igielman et al., 2025). Sulej (2007) proposed an opening between the “retroarticular process” formed by the prearticular and the arcadian process formed by the surangular (*sensu* Jupp & Warren, 1986) to be the chorda tympanic foramen, an interpretation that was followed by some subsequent studies (Gee & Kufner, 2022; Kufner & Gee, 2021). In other temnospondyls, the groove between these processes is the arcadian groove (Jupp & Warren, 1986) which may simply be deeper in some metoposaurids with a large throughgoing canal. Indeed, this opening was identified as the “Meckelian cleft/fissure” (in the original French: “la fente meckelienne”) in the metoposaurid *Dutuitosaurus ouazzoui* (Dutuit, 1976b). Several foramina are present in the glenoid region of well-prepared metoposaurid mandibles (*e.g*., fig. 31: Dutuit, 1976b; fig. 33: Gee & Kufner, 2022), and it is not clear which of these represent the chorda tympanic foramen without access to the internal canal morphology (*e.g*., a canal passing anteriorly through or on the surface of the articular). A large foramen on the anterior region of the articular may be the true chorda tympanic foramen (*e.g*., fig. 35: Gee & Kufner, 2022), but establishing the homology of mandibular features is beyond the scope of this study. Because scores for the chorda tympanic foramen were acquired using this erroneous interpretation, character 114 should be considered unknown in metoposaurids in phylogenetic analyses until homology of the foramina in the posterior mandible of metoposaurids is reassessed.

124—. No metoposaurids are known to have a procoelous centrum. In fact, no temnospondyls are known to have a procoelous centrum. The condition of most “rachitomous” (~non-stereospondyl) temnospondyls is difficult to assess due to an often large notochordal canal compounded with the morphological complexity of multi-part centra (paired pleurocentra and, typically, a single intercentrum). However, the majority of temnospondyl vertebral bodies could be considered amphicoelous with a concavity on the anterior and the posterior face of the centrum as originally interpreted by McHugh (2012). In contrast, the much larger intercentrum coupled with reduced or unossified pleurocentra of stereospondyls that form little of the anteroposterior contact between centra is more straightforward to assess and provides the opportunity for the presence of different vertebral body types. The vertebral bodies of metoposaurids range in shape along the axial column with anterior post-atlantal centra being strongly opisthocoelous (strongly convex anterior face and concave opposing posterior face), and the trunk centra gradually becoming acoelous (essentially flat to weakly amphicoelous) posteriorly (Dutuit, 1976b; Gee & Kufner, 2022; Konietzko-Meier, Bodzioch & Sander, 2012; Sulej, 2007). Previous authors have suggested that *Dutuitosaurus* has a concavity on the anterior face of the third vertebra (Buffa, Jalil & Steyer, 2019), but this condition would be better described as amphicoelous due to a corresponding concavity on the posterior face (fig. 34: Dutuit, 1976b). However, Dutuit’s illustration could also be interpreted as anteriorly convex (as it is here) due to a lack of unambiguous shading indicators for depth which would be more consistent with the condition in other metoposaurids. A study focusing on the morphology of the metoposaurid axial skeleton could further clarify morphological differences or possible regionalization along the axial column.

### Comparison of small-bodied metoposaurid dermal elements

Gee & Kufner (2022) suggested comparisons of metoposaurids with *Apachesaurus gregorii* would be more appropriate with similarly sized specimens, for example, YPM VPPU 021742, a small impression of the dorsal skull roof of a metoposaurid previously referred to *Buettnererpeton bakeri*. However, in the present analyses, YPM VPPU 021742 does not exclusively form a clade with specimens of *B. bakeri*, but in some analyses, it is sister to a core clade of *A. gregorii* which are notably all small-bodied (Figs. 58–61). As a result, YPM VPPU 021742 is considered indeterminate at the genus level, but additional small-bodied cranial and postcranial specimens can now be referred to *B. bakeri* (Figs. 5–7, 11, 14, 16).

The smallest cranial specimens of *B. bakeri* have at minimum a deeper and narrower otic notch than any specimens of *A. gregorii* at similar size or even smaller (Figs. 6, 14), and the smallest specimen of *B. bakeri* has a well-developed tabular horn (TMM 31099-128: Fig. 6). The width of the cultriform process in the smallest specimens of *B. bakeri* is wider than the minimal width of the palatal ramus of the pterygoid in contrast to the holotype of *A. gregorii* in which the minimal width of the cultriform process is slightly less than the palatal ramus of the pterygoid. The paraquadrate foramen can only be evaluated in three small-bodied specimens in the present study, and it seems to be proportionally wider in putative juvenile specimens of *B. bakeri* (Figs. 7, 14) than in *A. gregorii* (Fig. 47) though not to the degree seen in adult specimens (*e.g*., Fig. 10).

The underdevelopment of the cranial lateral line sulci has frequently been invoked to taxonomically differentiate *A. gregorii* and to infer its ecology (see below). While more lateral line sulci were identified and described here (Fig. 46) in UCMP 63845 than in previous descriptions (see Hunt, 1993; Spielmann & Lucas, 2012), nonetheless, the lateral line sulci of the posterior skull roof are discontinuous, a condition also found in referred specimens of *A. gregorii* (Figs. 48–50), and all of these putative juvenile specimens of *A. gregorii* lack an accessory jugal sulcus and a transverse occipital sulcus. The full complement of cranial lateral line sulci found in large specimens of *B. bakeri* is also present in TMM 31099-128, but like the holotype of *A. gregorii*, the sulci are narrow and shallow, though the degree to which sulcus depth and width scale with skull size is not directly assessed here. Rinehart and Lucas (fig. 4d: 2018) measured the lateral line sulcus depth and width at several points across the skull roof of specimens of *Apachesaurus* and “*Koskinonodon perfectum*” (one skull from Metoposauridae indet. (UCMP 27039) and four skulls from the Lamy amphibian quarry Metoposauridae incertae sedis) and suggested that the width of the jugal sulcus at the posterior margin of the squamosal scales isometrically with skull length, but the other measurements were not discussed. Like *A. gregorii*, the supraorbital sulcus of Otis Chalk Quarry 2 specimens passes medial to the lacrimal (Figs. 5, 6), but it is not nearly as widely separated as it is in UCMP 63845 (Fig. 46). However, there is considerable variation in this character among the large-bodied specimens of *B. bakeri* (Figs. 3, 8, 9, 12) suggesting limited utility for taxonomic differentiation or ontogenetic assessment.

Some features of the dermal pectoral girdle elements, namely the clavicle and interclavicle, have been suggested to be of taxonomic utility or ontogenetically variable in *Apachesaurus gregorii*. Rinehart & Lucas (2018) suggested the development of the posterior process of the interclavicle may be indicative of early ontogeny in *A. gregorii*, and a similar condition is found in multiple specimens of *B. bakeri* but in a range of sizes (Figs. 17, 18: figs. 55–56: Gee & Kufner, 2022). Spielmann & Lucas (2012) suggested that the curvature of the ‘anterior margin’ of the clavicle may be useful in differentiating *A. gregorii* from other metoposaurids, and this is likely referring to the combination of a curved lateral margin and possibly including the clavicular contact which appears to be highly variable among metoposaurids. Significant variation in the morphology of the anterior end of the clavicle is typical among specimens of *B. bakeri*, *Anaschisma browni*, and *T. perfectum* with some specimens more rounded and others more pointed with no clear trend related to body size (Figs. 17, 19; figs. 52–53: Gee & Kufner, 2022; fig. 8: Kufner & Gee, 2021; figs. 53–61: Lucas et al., 2016). While some similar trends such as widening of the paraquadrate foramen in adults or underdevelopment of the lateral line sulci in juveniles appear to be present in both *A. gregorii* and *B. bakeri*, the dissimilarities between the crania of similarly sized juveniles of *B. bakeri* and *A. gregorii* suggest that there is a fundamental difference in the ontogenetic trajectory of these taxa. Further investigation into some of the hypothesized ontogenetic variability in the postcranial skeleton may yield taxonomically and phylogenetically informative results.

### Lateral line sulci as indicators of ontogeny and ecology

The presence and/or morphology of cranial lateral line sulci are commonly used to infer ecology or to score character states for phylogenetic inference in temnospondyls (Hunt, 1993; Rinehart & Lucas, 2018; Schoch, 2013; So et al., 2024; Sulej, 2007). These inferences are sound at the surface level because the lateral line system only functions in water due, at least in part, to the exposed gel of the cupula encasing the fragile cilia of each sensory organ (Coombs, Janssen & Webb, 1988; Coombs et al., 2014; Mogdans, 2019). Therefore, the presence of lateral line sulci implies a predominantly aquatic lifestyle, and this is largely in agreement with inferred ecology of stereospondylomorphs and some non-stereospondylomorph temnospondyls based on gill architecture (Schoch & Witzmann, 2011). However, caution should be exercised in these interpretations because developmental studies of actinopterygian fishes suggest that the pattern of osteological expression of cranial lateral lines could be attributed to ontogeny (Northcutt, 1989; Pastana, Bockmann & Datovo, 2020; Webb, 2014).

Development of the lateral line system is most well understood in actinopterygians largely due to extensive work on the model zebrafish *Danio rerio* (Coombs et al., 2014). The sensory organs of the lateral line system are called neuromasts, and there are two types: superficial neuromasts and canal neuromasts (Coombs et al., 2014). Superficial neuromasts are plesiomorphic to extant vertebrates, but canal neuromasts are ancestral to gnathostomes and frequently lost in some derived lineages (Northcutt, 1989; Webb, 2014). Only canal neuromasts directly interact with dermatocranial elements during development thus leaving an osteological record that can be examined in the fossil record. The pit line organs of extant amphibians and South American and African lungfishes are composed entirely of superficial neuromasts and embedded in dermal grooves that do not interact with the dermatocranium thus necessitating observation of other extant models (Northcutt, 1989; Northcutt, Catania & Criley, 1994; Webb, 2014).

In general, canal morphogenesis in actinopterygians begins near the end of the larval period (Fuiman, Higgs & Poling, 2004) with canal neuromasts initially embedded in the epithelium, followed by invagination of the epithelium to form a groove, then ossification of the walls of the epithelium, followed by formation of an epithelium roofing the canal, and finally ossification of the roof of the canal (Pastana, Bockmann & Datovo, 2020; Tarby & Webb, 2003; Webb, 2014). Fully integrated cranial lateral line canals were also present plesiomorphically in the dermatocranium of all major extant lineages of sarcopterygians—Actinistia, Dipnoi, and Tetrapodomorpha—including the extant Australian lungfish and the coelacanths suggesting that a similar developmental sequence was present in the common ancestor of Osteichthyes (Hensel & Balon, 2001; Webb, 2014). Temnospondyls appear to lack integrated cranial lateral line canals ancestrally, although some authors have suggested these canals were present in early temnospondyls likely due to early proposed affinities of the colosteids—now generally accepted as a clade of stem tetrapods—to the Temnospondyli (Bolt & Lombard, 2010; Romer, 1969; Schoch, 2009; Smithson, 1982). Most non-stereospondyl temnospondyls only retained cranial lateral line sulci through a “larval” stage much like modern amphibians, but stereospondyls commonly retained the sulci into their neotenic adult forms (Schoch, 2009, 2010, 2014). The presence of lateral line sulci and absence of canals suggest a truncation in the normal developmental routine of canal morphogenesis ancestral to temnospondyls.

Little is known of the earliest stages in “canal” morphogenesis in stereospondyls, but an early juvenile of the trematosaurid *Trematolestes hagdorni* has discontinuous lateral line sulci surrounding the orbit while larger specimens have continuous sulci throughout the skull roof (fig. 10: Schoch & Mujal, 2022). In addition, while most specimens including juveniles of the neostereospondyl *Benthosuchus sushkini* have most cranial lateral line sulci, the transverse occipital sulcus is only found in a large referred adult specimen (fig. 62: Bystrow & Efremov, 1940). Interestingly, the largest adults of *T. hagdorni* may lose the transverse occipital sulcus (Schoch & Mujal, 2022). The discontinuity or absence of some cranial lateral line sulci may indicate an early ontogenetic stage for the small crania of *Apachesaurus gregorii* (Figs. 46, 48–50). Temnospondyls also exhibit phylogenetic variability in the expression of the lateral line system. The transverse occipital sulcus is rarely present in temnospondyls with some authors suggesting it is only present in trematosauroid stereospondyls (*e.g*., Schoch, 2019). Partial transverse occipital sulci have been reported in rhytidosteids (Maganuco, Pasini & Auditore, 2014; Warren & Black, 1985), and a complete transverse occipital sulcus has been reported in the plagiosaurid *Gerrothorax pustuloglomeratus* (Hellrung, 2003) which suggests that the transverse occipital sulcus may be plesiomorphic to a more inclusive concept of Trematosauria (*e.g*., Maganuco, Pasini & Auditore, 2014). When the transverse occipital sulcus is present in trematosaurs, it is typically discontinuous with the other lateral line sulci of the postorbital skull roof (Schoch & Milner, 2000).

The morphology of cranial lateral line sulci may be informative for other aspects of ecology outside of a predominantly aquatic or “not necessarily aquatic” life history. Widened lateral line canals are associated with sedentary feeding strategies especially in low light environments (Coombs, Janssen & Webb, 1988; Webb, 2014). Lateral line canals act to filter background noise of water flow from biologically relevant frequencies, and as a result, fishes that live in turbulent or fast-moving waters or are fast swimmers tend to have a well-developed lateral line canal system while closely related fishes living in slow moving waters or that are relatively sedentary tend to have relatively underdeveloped lateral line canals (Engelmann et al., 2000; Mogdans, 2019). However, canal neuromast morphology is also highly diverse which can influence function, so the width of canals/sulci or additions of accessory sulci alone may not be sufficient to demonstrate specific ecological modes (Aggarwal et al., 2026). In addition, minor excursions onto land followed by desiccation of the superficial and/or canal neuromasts could have occurred because damaged neuromasts can regenerate (Coffin et al., 2014). However, these events were likely rare because repeated desiccation and subsequent necrosis of the neuromasts could increase chances for infection and reduce chances for survival.

The underdevelopment of cranial lateral line sulci in *A. gregorii* commonly used to suggest a “less aquatic” or “more terrestrial” ecology in *Apachesaurus* than other metoposaurids (*e.g*., Hunt, 1993; Rinehart & Lucas, 2018) is not sufficient to demonstrate terrestriality or even less reliance on water in *Apachesaurus*. An alternative hypothesis is that these incomplete and shallow sulci represent an early ontogenetic stage prior to the connection of the sulcus segments, analogous or perhaps homologous with stages in actinopterygian canal morphogenesis. The absence of accessory sulci or the transverse occipital sulcus in small-bodied specimens of *A. gregorii* could be attributed to the relatively calm lacustrine system inhabited by the Redonda Formation metoposaurids (Hester & Lucas, 2001; Lehman & Chatterjee, 2005; Martz, 2008), and the presence of these sulci in a putative adult (UCMP 27051) may suggest ontogenetic niche partitioning as previously proposed (Rinehart & Lucas, 2018). Rather than occupying more terrestrial habitats, juveniles of *Apachesaurus* may have occupied lower flow regime habitats while adults may have occupied higher flow regimes. This observation may also be extended to other North American metoposaurids such as *Anaschisma browni* that exhibits a simplified but fairly widened system of cranial lateral lines and is found exclusively in the “ochre unit” of the Popo Agie Formation, often interpreted as a large lacustrine system or even a persistent lake (Deckman, Lovelace & Holland, 2024; High & Picard, 1965).

### Vertebral ossifications relative to the notochord

Small intercentra of referred specimens of *Buettnererpeton bakeri* (Fig. 16) are notable in a few features, (1) trunk intercentra are proportionally more similar to large-bodied metoposaurids in being anteroposteriorly shorter than both height and width, (2) trunk intercentra are dorsally open forming a ventrally rounded “heart” shape in transverse section, (3) trunk intercentra lack a notochordal pit (Fig. 16). This suggests the position of the notochord relative to the trunk intercentra was dorsally placed as opposed to the central position of the notochordal canal in the intercentra of *Apachesaurus gregorii* (Fig. 52) and similar examples known throughout much of the Dockum Group and the Chinle Formation (Gee, Parker & Marsh, 2017; Gee & Parker, 2020; Hunt, 1993; Lucas et al., 2016; Lucas, 2020; Rinehart & Lucas, 2018; Spielmann & Lucas, 2012). This is supported by a dorsally placed notochordal pit in the intercentra from the type locality of *B. bakeri* (figs. 38–42: Gee & Kufner, 2022). In addition, referred specimens of *Tecovaserpeton perfectum*, while often missing the notochordal pit, in rare cases retain this pit, and it is centrally placed much like traditional small, elongate *Apachesaurus*-type intercentra. However, the central placement of the notochordal pit only appears consistent in the anterior trunk with slight dorsal displacement in the posterior trunk (A. Kufner, 2023, personal observations; figs. 41–42: Lucas et al., 2016).

One challenge with assessing the notochordal canal/pit from the literature and in museum collections is the paucity of figured and/or well-prepared intercentra from metoposaurid taxa other than *A. gregorii*, *B. bakeri*, *T. perfectum* and *Metoposaurus krasiejowensis* (Gee & Kufner, 2022; Lucas et al., 2016; Rinehart & Lucas, 2018; Spielmann & Lucas, 2012; Sulej, 2007). For example, of the 313 intercentra referred to *Paranaschisma howardensis* from Otis Chalk Quarry 3 that were observed, less than 50 were sufficiently prepared to view most of the surface, and those that were prepared often had the unfinished anterior and posterior faces ground down inhibiting identification of the notochordal pit. For intercentra of *P. howardensis* in which the notochordal pit could be identified, it was more dorsally placed than those of *A. gregorii* and *T. perfectum*, but similar to *A. gregorii* and *T. perfectum*, the anterior caudal intercentra form dorsally closed cylinders with the notochordal pit just dorsal to the center of the body of the intercentrum.

In addition to potential taxonomic differences, the position of intercentra relative to the notochord differs along the axial column with notochordal pits/canals generally appearing more dorsally placed in the anterior trunk and more ventral in the posterior trunk (A. Kufner, 2023, personal observations). As a result, dorsal closure of the intercentrum may be useful for determining the degree of osteological maturity if axial position can be determined based on a combination of transverse and sagittal profiles as well as parapophysis morphology. In addition to the position of the notochord, the smallest intercentra referred to *B. bakeri* (Fig. 16H) are slightly more elongate than the larger intercentra, although not to the degree considered diagnostic for *A. gregorii* (Hunt, 1993). Elongation of the intercentra may also be influenced by the position of the intercentrum in the axial column since the only anterior dorsal intercentrum from UCMP V6148 is about half the length of posterior trunk and caudal intercentra with similar height (Fig. 52). Further investigation into axial variation and allometry of metoposaurids is needed.

### On the ontogenetic status of the holotype of *Apachesaurus gregorii*

Hunt (1993) used four criteria to argue that the holotype of *Apachesaurus gregorii* (UCMP 63845) and other referred specimens represent adults: (1) juveniles of other species appear more similar to the adults than *Apachesaurus* does to any other species; (2) elongate intercentra considered diagnostic of the genus *Apachesaurus* co-occur with short metoposaurid intercentra; (3) only small-bodied specimens of *Apachesaurus* were present in the collections at NMMNH at the time of Hunt’s study; and (4) the placement of the pineal foramen far posterior relative to the orbits.

On criterion 1: Hunt used the examples of *Dutuitosaurus ouazzoui* and “*Buettneria*” *perfecta* (=*Tecovaserpeton perfectum*, *Paranaschisma howardensis*, *Anaschisma browni* (in part), and *Buettnererpeton bakeri* (in part)) citing block MNHN XIII/38/65 (pl. xxxiv: Dutuit, 1976b) and, erroneously, the holotype of “*Arganasaurus*” *azerouali* (pl. xxxv: Dutuit, 1976b) for *Dutuitosaurus*, and TMM 31099-128 (Figs. 6, 7; figs. 8a–b: Hunt, 1993) for “*Buettneria*” as evidence for similarity in overall form of the juveniles and adults of these taxa. There is another juvenile specimen of *Dutuitosaurus* included in the photographic plates of Dutuit (pl. xxxii: 1976b) that lends credence to Hunt’s argument. In recognition of the differing taxonomic referral of TMM 31099-128 of this study relative to Hunt’s referral, comparisons are drawn between TMM 31099-128 and adult specimens of *B. bakeri* because Hunt’s argument still holds. I fully agree that juvenile specimens of both *D. ouazzoui* and *B. bakeri* appear more similar in overall cranial morphology with the adults of their respective species. For example, juvenile specimens of both *D. ouazzoui* and *B. bakeri* have a relatively wide cultriform process of the parasphenoid, indeed, wider than the minimal width of the palatal ramus of the pterygoid in contrast to UCMP 63845. The juvenile specimens of both species also have a more developed tabular horn at similar or even slightly smaller size compared to UCMP 63845. This pattern of development has also been observed in other trematosaurs and stereospondyls more broadly (Bystrow & Efremov, 1940; Schoch, 2009; Schoch & Mujal, 2022; Warren & Hutchinson, 1988; Witzmann, Scholz & Ruta, 2009).

On criterion 2: As previously noted, the axial position of intercentra may play a large role in the relative proportions of the vertebral body (*e.g*., Figs. 16, 52). An evaluation of intercentrum morphometrics requires strict criteria for the assignment of each intercentrum to axial position and a robust taxonomic framework independent from the dimensions of intercentra. While the present study provides a more robust taxonomic framework, a morphometric analysis of intercentra is beyond the scope of this study.

On criterion 3: While I cannot verify the specimens present at the time at NMMNH from the Bull Canyon and Redonda formations alluded to by Hunt (p. 85: 1993), I have no reason to believe this to be false. The more pertinent issue is that many of the criteria for assigning fragmentary metoposaurid material to *Apachesaurus* may instead be indicative of juvenile metoposaurids more broadly and not taxonomically informative. Indeed, Hunt suggests on the same page that *Apachesaurus* retains many juvenile stereospondyl characteristics: a high, convex skull profile in occipital view, shallow otic notches, and small zones of intensive growth (fewer elongate ridges and grooves, more reticulate ornamentation)—excluding presumed taxonomically informative characters such as the undeveloped tabular horn frequently used to assign fragmentary small cranial material to *Apachesaurus* (*e.g*., Spielmann & Lucas, 2012).

On criterion 4: The position of the pineal foramen in UCMP 63845 and referred small-bodied specimens of *Apachesaurus* is more posteriorly placed between the parietals than in many stereospondyls, but that is typical for metoposaurids and more broadly for Trematosauria (Schoch & Milner, 2000, 2014). In large-bodied metoposaurids, the placement of the pineal foramen in the posterior ¼ or less of the parietal is typical, but in small-bodied specimens of *A. gregorii*, the pineal foramen is positioned in the posterior ⅓ or even slightly anterior. In the context of other metoposaurids and trematosaurs, this position of the pineal foramen would be interpreted as appearing more juvenile based on Hunt’s original references to capitosaurs (Warren & Hutchinson, 1988) and the neostereospondyl (although sometimes early-diverging trematosaur) *Benthosuchus sushkini* (Bystrow & Efremov, 1940). However, more recent studies on trematosaurids would be more appropriate for comparison (Schoch, 2019; Schoch & Mujal, 2022). For example, in the smallest specimen of *Trematolestes hagdorni*, the pineal foramen is positioned at about the midlength of the parietals, and in larger specimens, the pineal foramen is more posterior (figs. 3, 5, 10: Schoch & Mujal, 2022). This would indicate that the position of the pineal foramen in small-bodied specimens of *A. gregorii* appears like another juvenile trematosaur.

Altogether, these observations suggest that *Apachesaurus* underwent a shift in developmental routine relative to the ancestral metoposaurid ontogenetic sequence, but the underlying description of this shift in developmental timing depends entirely on whether these small-bodied specimens of *Apachesaurus* are interpreted as adults or juveniles. The combination of achieving adult resemblance at small body size and slow development thereafter resulting in gigantism in stereospondyls seems to result from deceleration (*i.e*., neoteny, slowed developmental sequence: Alberch et al., 1979) of the ancestral ontogenetic sequence of temnospondyls (Dobreva, Camacho & Abzhanov, 2022; Schoch, 2009, 2010; Witzmann, Scholz & Ruta, 2009), and this appears to be the case for both *Dutuitosaurus ouazzoui* and *Buettnererpeton bakeri*. Hunt (1993; p. 85) suggested that progenesis (*i.e*., hypomorphosis, early termination of a developmental routine: Alberch et al., 1979) describes the pattern of retention of juvenile characters of *A. gregorii* which achieved somatic maturity at smaller body size than the presumed ancestral adult size of metoposaurids. Presumably, this would be hypomorphosis of the previously discussed features of the cranium followed by a gradual increase in body size, but to a much smaller body size than most other metoposaurids. An alternative to this, and the preferred interpretation here, based on the identification of a putative large-bodied adult (UCMP 27051) would be that *A. gregorii* exhibited post-displacement of the ancestral developmental sequence of metoposaurids resulting in the retention of “larval-like” characters into larger body size and subsequent development of adult characters in the typical ontogenetic sequence of stereospondyls. In other words, this may be an example of the post-displacement of “metamorphosis.” Another alternative explanation could be acceleration in early growth rate decoupled from the acquisition of adult characters, but the absence of large regions of intensive growth in the skull roof of small-bodied *A. gregorii* implies this is unlikely. Further investigation into heterochronic shifts in metoposaurids and *A. gregorii*, in particular, will require more well-established growth series of metoposaurids, including early juvenile forms of other species.

### On ecological interpretations of *Apachesaurus gregorii*

Hunt (1993) proposed a “less aquatic” ecology for *Apachesaurus gregorii* relative to other metoposaurids based on a relatively poorly developed cranial lateral line system and the formation of a “distinct acetabulum” in addition to its association with an “anomalously rich” terrestrial fauna citing the presence of dinosaurs, rauisuchians, and sphenosuchians at sites that yield referred material of *Apachesaurus*. This “less aquatic” interpretation of *A. gregorii* has been maintained by subsequent authors (Lucas et al., 2016; Rinehart & Lucas, 2018; Sulej, 2007) adding well-developed, elongate intercentra (presumably to stiffen the trunk in a laterally undulating walk), and robust rib facets (parapophyses; presumably for ventilation of the lungs).

The underdevelopment of the cranial lateral line sulci could be entirely due to ontogeny or even ontogenetic niche partitioning into different water flow regimes (see above). Stiffening of the trunk is widespread among terrestrial temnospondyls, but this is typically achieved through extra-vertebral ossifications (*e.g*., Dilkes, 2009). Indeed, elongation of the axial column is often associated with more aquatic amphibians (Schoch, 2009; Urošević et al., 2016), and the axial skeleton of *A. gregorii* is too incomplete to determine whether a concomitant reduction in vertebral number was present in *A. gregorii*. The “distinct acetabulum” of the ilium (interpreted here as the development of the supraacetabular buttress as well as an anterior ridge) used to suggest that *A. gregorii* was more terrestrial (figs. 14b–c: Hunt, 1993) can also be found in some large-bodied, fully aquatic metoposaurids (fig. 62: Gee & Kufner, 2022; fig. 67: Lucas et al., 2016; figs. 58–59: Sulej, 2007). Terrestrial and aquatic morphologies in the appendicular skeleton of temnospondyls are well characterized (*e.g*., Pawley, 2007; Pawley & Warren, 2005, 2006). Additional features of the appendicular skeleton of *A. gregorii* in common with other metoposaurids and many other aquatic stereospondyls include both a proportionally large pectoral girdle which would restrict forelimb range of motion and also a poorly ossified pelvis (only the ilium and the ischium are ossified, and they are not coossified) with short sacral rib attachments (Rinehart & Lucas, 2018; Spielmann & Lucas, 2012). Unfortunately, the paucity of referred forelimb and hindlimb material of *A. gregorii* inhibits the identification of more ecologically informative morphology such as ossified epiphyses or well-developed muscle attachment sites that could better support terrestrial locomotion.

### How many metoposaurids is too many metoposaurids?

The number of metoposaurid species in North America has fluctuated since the first description of putative metoposaurid material from the Newark Supergroup (Leidy, 1856). Early workers often invoked very minor differences when erecting novel taxa (*e.g*., Branson, 1905; Branson & Mehl, 1929; Case, 1920; Lucas, 1904), differences that can now be considered within the range of intraspecific variation based on descriptions of abundant material from bonebeds (*e.g*., Gee & Kufner, 2022; Lucas et al., 2016; Sulej, 2007). Prior to Colbert & Imbrie (1956) revision, 13 species of North American metoposaurid were named, but in their revision, they identified two species and two subspecies under one of these species: *Eupelor browni*, *Eupelor fraasi fraasi*, and *Eupelor fraasi jonesi*. Subsequent revisions and descriptions of metoposaurids (*e.g*., Gee & Jasinski, 2021; Gee & Kufner, 2022; Hunt, 1993; Kufner & Gee, 2021; Lucas et al., 2016; Rinehart, Lucas & Heckert, 2024) have typically recognized three North American species (or junior synonyms therein): *Anaschisma browni*, *Buettnererpeton bakeri*, and *Apachesaurus gregorii*. A strong emphasis has been placed on the position of the lacrimal for differentiating *An. browni* and *B. bakeri*, but other diagnostic features of *A. gregorii* have been used in its diagnosis. Some workers insist that due to the separation of the lacrimal from the orbital margin in *B. bakeri* that it should be placed in the genus *Metoposaurus* despite postcranial morphology suggesting more similarities with *An. browni* and *A. gregorii* (*e.g*., wide regions of reticulate ornamentation on the clavicle) and the fact that most *Metoposaurus* specimens have a lacrimal entering the orbital margin (Lucas, 2020; Rinehart, Lucas & Heckert, 2024). This emphasis on using a single character for uniting numerous specimens under a single taxon ignores the totality of evidence demonstrated here in the phylogenetic analyses.

These three species in their previous conception spanned different geographic and chronostratigraphic ranges (see Fig. 1). Recent radioisotopic ages from the Popo Agie Fm coupled with depositional modeling place an age of ~231–229 Ma on occurrences of *B. bakeri* from the NK locality in the “purple unit,” and occurrences of *An. browni* from the “ochre unit” of the Popo Agie Fm range from ~229–227 Ma (Lovelace et al., 2025).

There is little age constraint on the formations of the Dockum Group outside of vertebrate biostratigraphy (see Cornet, 1993), but most occurrences of *Paranaschisma howardensis* are restricted to the Otischalkian holochronozone of west Texas with the only occurrences from the Colorado City Member (probably equivalent to the lower Cooper Canyon Formation and the lower Tecovas Formation; see Martz, 2008; Martz & Parker, 2017) and likely the Tecovas Formation, low in the Dockum Group. According to catalog information at UMMNH, UMMP 21326 is from Cerita de la Cruz Creek (often referred to as Sierrita de la Cruz Creek or informally as Sweetly Cruize), and from this places UMMP 21326 in the vicinity of the Rotten Hill bonebed (Lucas et al., 2016). The precise stratigraphic position of UMMP 21326 is unclear, but based on outcrops of the Dockum Group in Potter County, TX, it is almost certainly from the Tecovas Formation (Lehman & Chatterjee, 2005). Differences in preservation from the Rotten Hill material also suggest a different preservational environment (A. Kufner, 2022–23, personal observations). The presence of leptosuchomorph phytosaurs from the same locality number in the UMMNH collection may suggest an Adamanian age for UMMP 21326 (Martz & Parker, 2017), but this locality number was applied for numerous collections along this creek. With estimates for the Otischalkian–Adamanian boundary placed in the lower Tecovas Fm (Martz & Parker, 2017), the occurrence of UMMP 21326 may range from the upper Otischalkian to the lower Adamanian.

The age of the oldest occurrences of *Tecovaserpeton perfectum* in this study are from the (upper?) Tecovas Formation of the Dockum Group, and the age of the Tecovas Formation is poorly resolved possibly ranging from the latest Carnian into the middle–late Norian (Martz & Parker, 2017). However, the age of the upper Tecovas Formation can be constrained to the Adamanian holochronozone due to the occurrence of leptosuchomorph phytosaurs (Lovelace et al., 2024; Lucas et al., 2016; Martz & Parker, 2017). The youngest tentatively referred specimen of *T. perfectum* (UCMP 27103) is from the lower Petrified Forest Member of the Chinle Formation, and the age of the lower Petrified Forest Member is estimated between 214–210 Ma (Rasmussen et al., 2021). Additional material previously referred to “*Koskinonodon perfectum*” from the Lamy amphibian quarry in the Garita Creek Formation (*e.g*., Rinehart, Lucas & Heckert, 2024) could not be reliably referred to an existing metoposaurid species and is thus considered Metoposauridae incertae sedis. However, the Garita Creek Formation is generally considered to span the Adamanian holochronozone and would offer no additional information on the age range of *T. perfectum* if a referral of the Lamy material to *T. perfectum* could be justified.

Former estimates of the chronostratigraphic range of *A. gregorii* relied on the referral of elongate intercentra resulting in a range of >20 Ma (Gee & Kufner, 2022). In the current taxonomic framework, the oldest occurrences of *A. gregorii* are known from the lower Petrified Forest Member of the Chinle Fm (~214 Ma), and youngest occurrences are from the Redonda Formation which is generally regarded as Rhaetian in age (~208.5–200 Ma; but Spielmann & Lucas (2012) suggest the Redonda Fm may span the Norian–Rhaetian boundary) and spanning the Revueltian–Apachean holochronozones (Lovelace et al., 2024; Martz & Parker, 2017).

Extant amphibians may be informative for assessing expected geographic ranges of fossil amphibians. Recent advances in the mitogenomics of the Chinese giant salamander species complex (*Andrias* spp.) suggest widespread cryptic speciation with at least five species recognized over a geographic area similar in size to the Upper Triassic metoposaurid-bearing outcrops of the western United States (Marr et al., 2024; Turvey et al., 2019; Yan et al., 2018). Whether osteology can differentiate these species has not been tested, but this could serve as an informative comparison between the biological and morphological species concepts. Regardless of relatedness or details of ecological similarity, giant salamanders and metoposaurids almost certainly exhibited similar limits in dispersal potential being restricted to waterways or short forays onto land to reach a nearby body of water. With the presence of geographical barriers to dispersal such as the Ancestral Front Range and the Ancestral Uncompaghre Highlands during the early Late Triassic (see Lovelace et al., 2024), reproductive isolation of the northern (Chugwater) and southern (Dockum) North American metoposaurid populations was possible if not likely.

Even with the splitting of *An. browni* into three monospecific genera, the number of metoposaurid species in North America may still be underestimated with some recent authors suggesting the presence of a novel species from the Lot’s Wife beds in the Sonsela Member of the Chinle Formation at Petrified Forest National Park (Gee, Soler & Parker, 2016). Indeed, UCMP 27024 (Fig. 57) exhibits unique morphology among North American metoposaurids that may suggest a close relationship or convergence with European metoposaurids. A heuristic approach (*e.g*., Wells et al., 2022) like the present study including further description and reevaluation will be necessary to assess the diversity of metoposaurids in the Chinle Fm and the upper Dockum Group.

### A case for monospecific genera

The decision to define two novel genera (*Paranaschisma* and *Tecovaserpeton*) in this study to accommodate each of the five recognized species in its own genus will without doubt be met with skepticism from some authors who have long viewed the metoposaurids with a lacrimal contribution to the orbital margin in North America as synonymous. However, modern workers are increasingly questioning historical precedents for generic synonymy of fossil vertebrates especially with an increasing number of specimen-level phylogenetic studies (Fitch et al., 2023; Gee & Kufner, 2022; Taylor, 2026; Tschopp, Mateus & Benson, 2015). This novel taxonomic framework is based on not only specimen-level phylogenetic hypotheses that are subject to refinement or even outright rejection by the broader paleontological community but also on phenetic diagnoses that demonstrate additional morphological differences between previously defined holotypes of metoposaurid species that can be applied to additional referred specimens. The nodal support in all phylogenetic analyses is low for clades within the Metoposauridae, a finding consistent with previous phylogenetic analyses of metoposaurids (Buffa, Jalil & Steyer, 2019; Chakravorti & Sengupta, 2019; Gee, Parker & Marsh, 2020; Gee & Kufner, 2022), but the recovery of several monophyletic clades formed by specimens including previously designated holotypes implies some level of differentiation between these specimens is possible. Assigning any of these species to a preexisting genus would imply a close phylogenetic relationship between the species, a hypothesis that is supported in some analyses and breaks down in others.

For example, the holotype of *Tecovaserpeton perfectum* (UMMP 7475) is equally closely related to *Apachesaurus gregorii*, *Anaschisma browni*, and *Paranaschisma howardensis* in all the Bayesian analyses which in one interpretation could synonymize all of these taxa with *An. browni*. However, UMMP 7475 is also equally closely related to a clade consisting of *Buettnererpeton bakeri* and *Arganasaurus lyazidi* which can be differentiated from *An. browni* with phenetic diagnoses. Should all North American species be synonymized under *An. browni*? Another phylogenetic history of North American metoposaurids is recovered in one of the parsimony analyses in which specimens of *B. bakeri* + *A. gregorii* form a clade with specimens of *T. perfectum* + *P. howardensis* + UCMP 27051, a referred adult specimen of *A. gregorii*, to the exclusion of *An. browni* (Fig. 59A). A recent revision of “*Arganasaurus*” *azerouali* (Buffa, Jalil & Steyer, 2019) suggested a sister relationship with *Ar. lyazidi*—a relationship not recovered here, casting doubt on the generic referral of the species originally assigned to “*Metoposaurus*” *azerouali* (Dutuit, 1976b). Indeed, *Metoposaurus* is never recovered as a monophyletic clade in any of the present analyses, so should *Metoposaurus* be restricted to the type species *Metoposaurus diagnosticus*, or should all metoposaurids be placed under the genus *Metoposaurus* as separate species as previously proposed (RoyChowdhury, 1965)?

Phylogenetic hypotheses supporting a taxonomic framework are subject to change with character and OTU inclusion or exclusion, and novel generic assignments based on these new hypotheses serve only to add confusion in taxonomic history and current accepted use. There is a fundamental flaw underlying the approach of incorporating two or more extinct morphologically defined species into a genus: the relatedness of any two or more fossil specimens is a hypothesis based on often inadequate material and inadequate character or taxon sampling. It has become increasingly apparent that it is essential to maintain taxonomic stability to promote clarity and consistency when comparing specimens. Assigning each of these species to its own genus is the most taxonomically stable approach and avoids the propagation of increasingly lengthy and confusing synonymy lists.

## Conclusions

Through a combination of specimen-level phylogenetic analyses and reevaluation of phenetic diagnoses, five metoposaurid species were recognized in the Upper Triassic of North America. These species were each placed in their own genus following the establishment of two novel genera to accommodate two species previously referred to *Anaschisma browni*. This taxonomic framework is intended to improve stability in the Metoposauridae by providing a naming scheme agnostic to the relatedness of the individual species. Differences in the cranial lateral line sulci in North American metoposaurids can be attributed to both ontogeny and ecology, but disentangling the influences of both will require additional sampling and assessment. Juvenile specimens of *Buettnererpeton bakeri* were identified and described as such, revealing a conserved developmental trajectory with other Triassic stereospondyls. A large-bodied adult of *Apachesaurus gregorii* was identified and suggests a shift in the early development of *A. gregorii* relative to the ancestral metoposaurid condition that may have resulted either in a prolonged “larval” state or an accelerated increase in body size prior to acquiring adult characteristics which will require further description of late Norian–Rhaetian (Revueltian–Apachean) metoposaurid specimens to evaluate. The shift in development of *A. gregorii* is tentatively related to ontogenetic niche partitioning. The increasing ability to evaluate large numbers of specimens to test species delimitations will necessitate periodic revision to fossil taxa and taxonomic schemes to ensure the validity of taxa widely used for paleobiological inferences.

## Supplemental Information

10.7717/peerj.21720/supp-1 Supplemental Information 1 Character list, changes, and supplemental rib histology description.

10.7717/peerj.21720/supp-2 Supplemental Information 2 Character matrix.

10.7717/peerj.21720/supp-3 Supplemental Information 3 Bayes blocks used in Bayesian phylogenetic analyses.

## Institutional Abbreviations

FMNH UC: Field Museum of Natural History (University of Chicago collection), Chicago, IL
MCZ: Museum of Comparative Zoology, Harvard University, Cambridge, MA
MNHN: Muséum national d'Histoire naturelle, Paris, France
MOTT VPL: Museum of Texas Tech Vertebrate Paleontology Locality, Lubbock, TX
MU: University of Missouri, Columbia, MO
PPHM (WT): Panhandle Plains Historical Museum (West Texas), Canyon, TX
TMM: Texas Memorial Museum, Austin, TX
TTU-P: Texas Tech University (Paleontology collection), Lubbock, TX
UCMP: University of California Museum of Paleontology, Berkeley, CA
UMMP (=UMMNH): University of Michigan Museum of Paleontology, Ann Arbor, MI
UWGM: University of Wisconsin Geology Museum, Madison, WI
YPM (VPPU): Yale Peabody Museum, Ithaca, NY
ZPAL: Institute of Paleobiology, Polish Academy of Sciences, Warsaw, Poland

## Additional Information and Declarations

### Data Availability

The following information was supplied regarding data availability:

The data and code are available in the Supplemental Files.

### New Species Registration

The following information was supplied regarding the registration of a newly described species:

Publication LSID: urn:lsid:zoobank.org:pub:CF860616-7B8B-4815-90E7-1973FC686972.

Paranaschisma gen. nov. genus LSID: urn:lsid:zoobank.org:act:D1EB4464-096F-433D-8559-750491195662.

Paranaschisma howardensis species LSID: urn:lsid:zoobank.org:act:2780A989-5AD5-4C94-8F20-EB0E661D5247.

Tecovaserpeton gen. nov. genus LSID: urn:lsid:zoobank.org:act:984AF4FC-9CEB-4CF9-BAE8-B376E8774B4C.

Tecovaserpeton perfectum species LSID: urn:lsid:zoobank.org:act:3328F3B8-C953-4211-BCE0-C400A14ED38B.
