# Supplementary material for: Specimen-level phylogenetic analysis and taxonomic revision of North American metoposaurids: a case for monospecific genera in vertebrate paleontology
Source: PeerJ. 2026 Sep 23;14:e21720. doi: 10.7717/peerj.21720 (PMC13615736; doi:10.7717/peerj.21720)
Supplement: Supplementary file 1 [file peerj-14-21720-s001.docx]

**Supplementary File S1**

**for**

**Specimen-level phylogenetic analysis and taxonomic revision of North American metoposaurids: A case for monospecific genera in vertebrate paleontology**

Aaron M. Kufner

**Table of contents**

**Character list**

…………………………………………………………………… 1–16

**Included taxa**

Table S1………………………………………………………… 17–18

**Character state changes**

…………………………………………………………………… 19–32

**Notes on non-metoposaurids**

…………………………………………………………………… 33–34

**Instructions for analyzing the character matrix**

…………………………………………………………………… 34

**Rib histology methods, results, and interpretation**

…………………………………………………………………… 35

**References**

…………………………………………………………………… 36–44

**Supplemental figures and captions**

…………………………………………………………………… 45–56

**Additional Supplemental Files**

**Appendix 2: Character matrix including all characters, specimen-level OTUs, and species-level OTUs in NEXUS format**

**Appendix 3: Bayes blocks used in Bayesian inference with Appendix 2**

**Character list (Based on Gee and Kufner 2022).**

**Reference character matrix abbreviations: Pardo, Huttenlocker, and Small, 2017 (PHS); Chakravorti and Sengupta, 2018 (CS); Buffa, Jalil, and Steyer, 2019 (BJS); Gee, Parker, and Marsh, 2020 (GPM); Gee and Kufner, 2022 (GK). Characters marked with (*) may be influenced by ontogeny in metoposaurids; characters marked with (**) are considered problematic. This is further elaborated in the main text.**

1. **Skull (proportions).** longer than wide (0); wider than long (1). [PHS – 215]
2. **Preorbital-postorbital (length ratio).** ratio of preorbital to postorbital length < 1 when measured along midline (0); ratio between 1 and 2 (1); ratio >2 (2). [GK – 2; modified from GPM – 4; adapted from Schoch, 2013] – ordered
3. **Orbit location.** medial, framed by wide jugals laterally (0); lateral emplacement, framed by very slender jugals (1). [GPM – 11; adapted from Schoch, 2013]
4. **Orbits in interpterygoid fenestrae.** posterior (0); second anterior quarter of interpterygoid fenestrae (1); anterior quarter of interpterygoid fenestrae (2). [BJS – 30] – ordered
5. **Ornamentation (prepineal region).** elongate grooves present (0); absent (1). [Schoch, 2013 – 6]
6. **Lateral line sulci.** throughout skull roof (0); confined to circumorbital region (1); absent (2). [adapted from Schoch, 2013 – 34] – unordered
7. **Number of premaxillary teeth.** > 15 (0); 10-15 (1); < 10 (2). [PHS – 242] – ordered
8. **Number of maxillary teeth.** < 30 (0); 30-50 (1) >50 (2). [GK – 8; modified from PHS – 243] – ordered
9. **Premaxilla (alary process).** absent, premaxilla and nasal share a transversely oriented suture (0); present, forming a posterior indentation along the medial narial margin (1); present, forming a posterior triangular process fully separated from the naris (2). [GPM – 5; adapted from Milner, 1993; Schoch, 2013] – unordered
10. **Premaxilla (mandibular apertures).** openings in ornamented portion of premaxilla for symphyseal fangs absent (0); present (1). [modified from GK – 10]
    a. The wording of this character was modified to reflect the original intent and specify that the apertures pierce the ornamented roof portion of the premaxilla and form an additional hole separate from the narial fossa. Most stereospondyls have apertures that pierce through the juncture of the premaxilla and vomer (anterior palatal vacuity), but these are typically located within the narial fossa or do not pierce the skull roof if located more medially.
11. **Maxilla-orbital margin.** contact absent (0); contact present (1). [BJS – 6; adapted from McHugh, 2012]
12. **Nasal (width).** nasal longer than wide (0); as wide as long (1). [Schoch, 2013 – 16]
13. **Lacrimal.** present (0); absent (1). [Schoch, 2013 – 21]
14. **Lacrimal-nasal.** contact absent (0); contact present (1). [BJS – 7]
15. **Lacrimal (length).** compact, less than half of the distance between the naris and the orbit (0); elongate, at least 50% of the distance between the naris and the orbit (1). [GK – 15; modified from BJS – 8; adapted from Schoch and Milner, 2000]
16. **Lacrimal-orbit contact.** absent (0); present (1). [modified from GK – 16; BJS – 9; adapted from McHugh, 2012]
    a. The character-states were changed to reflect a poor distinction between broad and narrow contact of the lacrimal to the orbital margin. For example, both *Trematolestes* and *Callistomordax* were coded as having a broad contact because the entire width of the small lacrimal contributed to the orbital margin. However, in some specimens of *Metoposaurus krasiejowensis* which were coded as a narrow contact by GK, the proportion of the orbital margin made up of the lacrimal is approximately equal to that seen in *Trematolestes* and *Callistomordax*. For *Metoposaurus diagnosticus*, this character has been coded as narrow (BJS) and broad (GK) adding to the ambiguity in the distinction between narrow and broad contact.
    b. To capture some of the variation in lacrimal position relative to the orbit independent of contact, character 141 was added.
17. **Lacrimal-naris.** contact present (0); contact absent (1). [BJS – 10; adapted from Hunt 1993]
18. **Palatine (laterally exposed palatine, LEP).** absent (0); present (1). [Schoch, 2013 – 127]
19. **Prefrontal (anterior end).** pointed (0); blunt (1). [modified from GPM – 16; adapted from Schoch, 2013]
    a. **Original character: *Prefrontal (anterior end).*** *pointed (0); wide and blunt (1). [GPM – 16; adapted from Schoch, 2013]*
    b. This character was modified to reflect solely whether the shape of the anterior end of the prefrontal is pointed or blunt. An additional character was added to describe the width of the lateral and medial margins (ch. 142). This allows for the coding of character-states like blunt and narrow or pointed and wide anterior prefrontal ends.
20. **Prefrontal-maxilla contact.** absent (0); present anteriorly with an intervening element posteriorly (1); present with no intervening element (2). [modified from Schoch, 2013 – 45; Englehorn et al., 2008 – 5] – unordered
    a. **Original character: *Prefrontal (anterior contacts)****. nasal and lacrimal (0); nasal and maxilla (1); lacrimal and maxilla (2). [GK – 20, BJS – 11; adapted from McHugh, 2012]*. This character is one of three replacing BJS – 11 (GK – 20) which was modified from McHugh (2012) – 23 and Laurin & Reisz (1997) – 26.
    b. The prefrontal in temnospondyls tapers anteriorly in most taxa or is at least at its most narrow width. To avoid the issues that arise when a novel combination of contacts is found or if the prefrontal only contacts a single bone anteriorly (e.g., *Ninumbeehan dookoodukah*), this character was split into two additional characters involving the constituent bones that contact one another in the preorbital region (ch. 20 and ch. 143). In addition, one of the original states (state 0: nasal and lacrimal) was partially dependent on ch. 14 (presence or absence of a nasal-lacrimal contact). Typically, the intervening element between the maxilla and prefrontal is the lacrimal, but in some taxa it is a lateral exposure of the palatine (e.g., Pardo et al., 2017). It should be noted that in trematopids and some colosteids (a widely used outgroup for temnospondyl phylogenetic analyses), the prefrontal forms the posteromedial margin of the external naris or even contacts the septomaxilla (Laurin & Reisz, 1997; Bolt & Lombard, 2010), but these conditions were excluded here due to a lack of taxa that bear them in the current study.
21. **Prefrontal, frontal (anterior extent).** prefrontal terminating anterior to the anterior terminus of the frontal (0); terminating at same level as terminus (1); terminating posterior to terminus (2). – ordered [adapted from Schoch, 2013 – 39]
22. **Prefrontal-postfrontal.** sutured (0); separated by frontal (1). [Schoch, 2013 – 42]
23. **Prefrontal-jugal contact.** absent (0); sutured (1); point contact (2). [BJS – 12; adapted from McHugh, 2012] – unordered
    a. State 2 was added to differentiate the point contact present in taxa such as *Ninumbeehan* and *Almasaurus* from the more typical elongate suture. It is however unclear if these contact types represent a morphocline.
24. **Frontal.** paired (0); fused (1) [GPM – 101; adapted from Huttenlocker et al., 2013]
25. **Frontal-nasal (suture).** anterior to the orbit margin (0); level with margin (1); posterior to margin (2). [adapted from Schoch, 2013 – 32] – ordered
26. **Frontal, nasal (length).** frontal as long or longer than nasal (0); shorter (1). [Schoch, 2013 – 31]
27. **Postfrontal-prefrontal or -frontal suture (position).** anterior to the mid-length of the orbit (0); approximately at the mid-length of the orbit (1); well posterior to the mid-length of the orbit (2). [modified from GK – 27] – ordered
    a. **Original character: *Postfrontal contribution to orbital margin.*** *broad (0); reduced (1). [BJS – 13]*
    b. This character was modified to remove ambiguity in the terms “broad” or “reduced” when referring to the contribution of the postfrontal to the orbital margin. This character only assesses the position of the anterior suture of the postfrontal. Character 145 assesses the position of the lateral/posterolateral suture.
28. **Postorbital (lateral process).** not wider than orbit (0); with substantial lateral process projecting into jugal (1). [Schoch, 2013 – 49]
29. **Postorbital, postfrontal.** shorter than supratemporal and parietal (0); as long or longer (1). [GPM 20; adapted from Schoch, 2013]
30. **Jugal (anterior margin).** posterior to the anterior orbital margin (0); level with anterior orbital margin (1); anterior to anterior orbital margin (2). [BJS – 14; adapted from McHugh, 2012] – ordered
31. **Parietal (width relative to frontal).** greater (0); less than or equal to (1). [PHS – 312]
32. **Parietal (length).** shorter than frontal along midline (0); as long or longer (1). [GK – 32].
33. **Parietal-supratemporal (suture).** straight (0); angled (1). [BJS – 15]
34. **Parietal-postorbital.** contact absent (0); present (1). [PHS – 232]
35. **Parietal-tabular.** absent (0); present (1). [PHS – 234]
36. **Pineal foramen (position).** within anterior half of the parietals (0); within posterior half of parietals (1). [GK – 36]
37. **Supratemporal (proportions).** longer than wide (0); quadrangular (1). [Schoch, 2013 – 53]
38. **Squamosal (shape).** pentagonal (0); sub-triangular (1). [GK – 38]
39. **Squamosal (falciform crest).** posterior rim of squamosal straight (0); with convex projection, referred to as falciform crest (1). [GPM – 26; adapted from Schoch, 2013]
40. **Squamosal-tabular (dorsal).** separated by supratemporal (0); sutured (1). [GPM – 25 ; adapted from Schoch, 2013]
41. **Quadratojugal-maxilla.** contact present (0); contact absent (1). [GPM – 28; adapted from Schoch, 2013]
42. **Quadrate-squamosal.** contact present (0); excluded by the quadratojugal (1). [BJS – 32; adapted from McHugh, 2012]
43. **Otic notch.** enclosed otic notch (0); semicircular embayment ( 1); straight transverse posterior skull margin without embayment between cheek and table ( 2). [adapted from Schoch, 2013 – 51] – ordered
44. **Otic notch (position).** lateral, expanding along entire cheek to form continuous unornamented area up to quadrate (0); slit-like (1); small and rounded, confined to dorsomedial part of squamosal (2). [GPM – 21; adapted from Schoch 2013] – unordered
45. **Postparietal (proportions).** longer than wide (0); wider than long (1). [adapted from PHS – 237]
46. ***Tabular (horn).** well-developed (0); moderately developed (1); or absent (2). [modified from GK – 46; adapted from McHugh, 2012; Schoch, 2013] – ordered
    a. These states are here considered ordered due to their existence along a morphocline.
47. **Tabular (laterally directed parotic flange).** (0) absent, (1) present. [GK – 47]
48. **Postparietal (occipital flange).** in occipital view, postparietals discontinuous across dorsal part of the osseous foramen magnum (i.e., descending flange absent) (0); with a lamina extending ventrally into the dorsal portion of the osseous foramen magnum (i.e., descending flange present) (1). [modified from GPM – 30; Schoch, 2008a – 29, Schoch, 2013 – 72]
    a. **Original character: *Occipital flange****. descending flange of occipital portion of postparietals forming a bulge (0); long smooth blades as long as the dermal portion of the postparietal (1). [GPM – 30; Schoch, 2008a – 29, Schoch, 2013 – 72]*
    b. The original wording of this character has previously sparked confusion (e.g., Gee & Kufner, 2022) and here has been modified to retain the original intent while specifying that the unornamented part of the postparietal simply must exhibit a ventral flange that is not necessarily as long as the dorsal part of the postparietal. This ventral or descending flange extends beyond the main body of the postparietal and may be difficult to identify if significant sediment matrix remains within the brain case.
49. **Paraquadrate foramina (borders).** entirely within quadratojugal (0); framed by quadrate and quadratojugal (1); framed by squamosal and quadratojugal (2); framed by quadrate, squamosal, and quadratojugal (3). [GK – 49] – unordered
50. ***Paraquadrate foramina.** absent (0); small circular opening with maximum diameter less than that of the exoccipital condyle (1); large oval opening with maximum diameter greater than that of the exoccipital condyle (2). [GK – 50; modified from BJS – 37 and GPM – 102; adapted from McHugh, 2012, and Hunt 1993] – ordered
    a. While not quantitatively demonstrated here, it does appear that there is some allometry in the size of the paraquadrate foramen in small members of a given metoposaurid species relative to the skull size. Ontogenetic influence for this character should be considered in future studies.
51. **Posttemporal fenestrae (size).** as wide or wider than maximum width of foramen magnum (0); narrower (1). [GK – 51; modified from BJS – 39; adapted from McHugh, 2012]
52. **Posttemporal fenestrae (shape).** circular or oval (0); polygonal (1); circular opening recessed in polygonal fossa [NEW] (2). [GK – 52] – unordered
    a. Appendix 1 of GK lists this character with circular (0) and oval (1) as separate states, however, their matrix (Appendix 2) combines states 0 and 1 into the state “circular or oval (0)”. Here the latter is used due to an unclear distinction in circular and oval posttemporal fenestrae.
    b. State 2 was added to account for the condition found in *Apachesaurus* and in *Dutuitosaurus* in which the posttemporal fenestra itself is a small circular opening, but it is recessed within a subtriangular fossa. This has previously been considered a polygonal opening (e.g., Spielmann and Lucas, 2012; Buffa et al., 2019), but the condition is different from other stereospondyls that have a large polygonal opening such as *Angusaurus* (Fernández-Coll et al., 2019). Because these states do not lie along an unambiguous morphocline, they are treated as unordered. Similarly in *Metoposaurus algarvensis*, a fossa surrounds the posttemporal fenestra, but this fossa is a dorsoventrally elongate oval and not clearly subtriangular. Future authors may consider assessing this character more thoroughly.
53. **Foramen magnum (proportions).** dorsal portion dorsoventrally deeper (0); ventral portion dorsoventrally deeper (1); both portions equal in dorsoventral depth (2). [BJS – 41] – unordered
54. **Posteriorly projecting occiput.** absent (0); occiput partially exposed in dorsal view (1); occiput fully exposed, with foramen magnum largely visible in dorsal view. [BJS – 31; adapted from Hunt 1993] – ordered
55. **Skull (level of jaw articulation).** quadrate trochlea posterior to tabular horns (0); at one level or anterior (1). [GPM – 29; Schoch, 2013]
56. **Quadrate and occipital condyles.** quadrate condyles posterior to occipital ones (0); or at same level (1); or well anterior (2). [GPM – 31; adapted from Yates & Warren, 2000; Schoch, 2013] – ordered
57. **Infraorbital sulcus.** straight or gently curved (0); step-like (1); Z-shaped (2). [GK – 57; modified from BJS – 16; adapted from McHugh 2012] – unordered
58. **Supraorbital sulcus.** sulcus passes medial to lacrimal (0); sulcus enters lacrimal (1). [BJS – 17; adapted from McHugh, 2012]
59. **Temporal sulcus.** terminates anterior to the supratemporal (0); terminates on the supratemporal (1); passes onto the tabular (2); passes onto the squamosal (3). [modified from GK – 59; CS – 7] – unordered
    a. **Original character: *Postorbital sulcus.*** *does not terminate on the supratemporal (0); terminates on the supratemporal (1).*
    b. The name of this character was changed to specify that this is referring to the temporal part of the postorbital sulcus. State 0 was added to differentiate taxa in which the temporal sulcus terminates anterior to the supratemporal (e.g., *Sclerocephalus*). State 3 was added to differentiate the condition in specimens of *Apachesaurus* in which the temporal sulcus curves laterally and passes onto the squamosal rather than the typical path onto the tabular. The states were reorganized, and the wording of state 2 was modified to specify that the sulcus continues onto the tabular.
60. **Jugal sulcus-posterior skull margin contact.** absent (0); present (1). [BJS – 18]
    a. This was changed from “postorbital” to “jugal sulcus” to reflect the part of the postorbital sulcus intended.
61. **Postorbital-infraorbital sulci.** sulci remain distinctly separated posteriorly (0); sulci merge together on the jugal (1). [CS – 8]
    a. In all metoposaurid specimens scored here with sufficient preservation, the infraorbital sulcus merged with the jugal sulcus (postorbital sulcus, in part). This differs from some recent interpretations by GK, but see main text for further discussion.
62. **Palate structure.** in occipital view, pterygoids either sloping continuously ventrolaterally or flat horizontal (0); vertically curved ventrally at right angle with basicranium (1). [Schoch, 2013]
63. **Choana (shape).** oval (0); circular (1). [adapted from Schoch, 2013 – 101]
64. **Maxilla-choana.** forming most of the lateral border of choanae (0); lateral processes of vomer and palatine approach one another so as to reduce the maxillary contribution (1); vomer-palatine suture lateral to the choanae excludes maxilla entirely (2). [Yates & Warren, 2000 – 76] – ordered
65. **Choana, interpterygoid vacuity.** posterior margin of choana is anterior to the anterior margin of the vacuity (0); posterior margin of choana is at or posterior to the anterior margin of the vacuity (1). [GK – 65]
66. **Anterior palatal opening(s).** vomer and premaxilla with continuous suture and no fossa(e) for fangs (0); paired fossae for symphyseal fangs (1); single perforation for symphyseal fangs (2); paired perforations for symphyseal fangs (3). [modified from GK –66; adapted from Schoch, 2013 – 91 & 92] – unordered
    a. Added state for the paired fossae found in some non-stereospondyl stereospondylomorphs (e.g., *Archegosaurus decheni*, fig. 17: Witzmann, 2006) and reworded the first state to specify a lack of fossae/perforations.
67. **Palatal tusks (cross-section).** round or oval (0); laterally compressed and keeled at least on one side (1). [Schoch, 2013 – 80]
68. **Maxilla-vomer.** absent (0); present (1). [BJS – 20; adapted from McHugh, 2012]
69. **Vomer (denticles).** present (0) ; absent (1). [GK – 69; modified from GPM – 35; adapted from Schoch, 2013]
70. **Vomer (transvomerine tooth row).** absent (0); present and transverse (1); V-shaped (2). [GPM – 36; adapted from Schoch, 2013] – unordered
71. **Vomer (parachoanal tooth row).** absent (0); present (1). [BJS – 21; adapted from McHugh, 2012]
72. **Palatine (denticles).** present (0); absent (1). [PHS – 252]
73. **Ectopterygoid (denticles).** present (0); absent (1). [GK – 73]
74. **Ectopterygoid (fangs).** present (0); absent (1). [GPM – 38; Schoch, 2013 – 88]
75. **Jugal (ventral process).** no ventral outgrowth (0); short ventral outgrowth (insula jugalis) framing subtemporal window (1); insula jugalis occupies greater than 50% of the width of the palatal ramus. [modified from CS – 17] – ordered
    a. During the course of this study, it became clear that some metoposaurid specimens have a large *insula jugalis* that occupies over half of the attachment of the palatal ramus to the lateral palate.
76. **Palatine, ectopterygoid (suture).** straight or very slightly convex (0); sinuous or markedly convex (1). [GK – 76; modified from Schoch, 2013 – 125]
77. **Pterygoid (denticles).** present (0); absent (1). [PHS – 249]
78. **Pterygoid (anterior sutures).** Contacts both vomer and palatine (0); only contacts palatine (1); does not contact vomer or palatine (2). [adapted from Schoch, 2013 – 97] – ordered
79. **Pterygoid-exoccipital.** contact absent (0); present (1). [Schoch, 2013 – 118]
80. **Pterygoid, ectopterygoid.** palatine ramus exclusively formed by pterygoid (0); with posteromedial projection of ectopterygoid. [Schoch, 2013 – 123]
81. **Pterygoid (posterolateral flange).** absent (0); present (1). [BJS – 23]
82. **Pterygoid (palatine ramus).** medial margin is curved, concave medially (0); margin is straight (1); margin is curved, convex medially (2). [GK – 82; modified from BJS – 24] – ordered
83. **Pterygoid (oblique crest/otic ridge).** absent (0); present (1). [GK – 83; modified from BJS – 42; adapted from McHugh, 2012]
84. **Parasphenoid-pterygoid suture (orientation).** suture of the parasphenoid is not oblique (curved/straight) to the long axis of the skull (0); oblique to the long axis of the skull (1). [modified from GK – 84]
    a. Modified wording from GK to refer to the orientation of the basicranial suture.
85. **Basicranium (suture).** Suture much shorter than basal plate, reaching at best 40% its length (0); suture between 40% and 67% as long as the basal plate (1); suture almost as long as basal plate (2). [adapted from Schoch, 2013 – 107] – ordered
86. ***Parasphenoid (ornamentation).** absent (0); confined to basal plate (1); extends on the cultriform process (2). [BJS – 25] – ordered
    a. The states of this character may be ontogenetically influenced. No small metoposaurid specimens in this study exhibited any ornament on the basal plate. Similarly, the greatest extent of palatal ornament seems to be associated with larger individuals, but a broader taxonomic sample will be necessary to properly assess this.
87. **Parasphenoid (central depression).** depression on ventral surface of basal plate is absent (0); depression is present (1). [BJS – 26; adapted from McHugh, 2012]
88. **Parasphenoid (shagreen).** tooth patches present (0); teeth entirely absent (1). [GPM – 37; adapted from Yates & Warren, 2000; Schoch, 2013]
89. **Parasphenoid (plate, proportions).** longer than wide (0); equant or slightly wider than long (1); much wider than long (2). [adapted from Schoch, 2013 – 110] – ordered
90. **Parasphenoid (cultriform process, structure).** ventrally flat (0); with ridge emplaced on broader base (1); knife-edged and keel-shaped (2). [GK – 90; modified from BJS – 29; adapted from McHugh, 2012] – ordered
91. **Parasphenoid (cultriform process, relative width).** narrower than the minimal width of the palatal ramus of the pterygoid (0); subequal (1); broader (2). [modified from GK – 91; modified from BJS – 27; adapted from McHugh, 2012] – ordered
    a. The wording of this character was modified and a state was added to clarify how to assess the width of the cultriform process and remove any ambiguity. The minimum width of the cultriform process and the palatal ramus were used in this assessment. As a result of this modification, all OTUs were reviewed.
92. **Parasphenoid (cultriform process, post-vomerine width).** uniform width between base and posteriormost contact with vomers (0); continuously narrowing from base (1); continuously broadening from base (2); constricted in medial third (3); constricted in posterior third (4). [GK – 92; modified from BJS – 28; adapted from McHugh, 2012] – unordered
    a. This character appears entirely randomly distributed with no consistency of states within putative populations known from bonebeds. When included in preliminary analyses, Bayesian runs would not converge after over 100,000,000 iterations. As a result, this character was excluded from subsequent tests.
93. **Parasphenoid (cultriform process, anterior extent in ventral view).** terminates at or posterior to the level of the anterior margin of the interpterygoid vacuity (0); terminates anterior to this level (1). [modified from GK – 93]
94. **Quadrate trochlea.** medial bulge only slightly larger than lateral one (0); much larger (1). [adapted from Schoch, 2013 – 135]
95. ****Opisthotic.** ossified (0); unossified (1). [GK – 95; modified from BJS – 43]
    a. This character is considered dubious due to misidentification of the opisthotic that is further elaborated in the main text.
96. **Sphenethmoid.** ossified (0); unossified (1). [GK – 96; modified from CS – 54; Anderson et al., 2008 – 114]
97. **Dentition (marginal).** heterogeneous, varying sizes and distances (0); homogeneous, small teeth, equidistant (1). [GPM – 32; adapted from Schoch & Milner, 2000; Schoch, 2013]
98. **Symphyseal tooth row.** (0) absent, (1) present. [modified from GK – 98; GPM – 66; adapted from Schoch, 2013]
    a. Removed attribution of the symphyseal row of teeth to the adsymphyseal element which has been uncontroversially identified in some tetrapodomorphs (e.g., Bolt & Lombard, 2001) and has been proposed for some temnospondyls (e.g., Kligman et al., 2023).
99. **Middle coronoid.** denticles only (0); teeth only (1); edentulous (2). [GK –99; modified from BJS – 44] – unordered
100. **Anterior coronoid.** denticles only (0); teeth only (1); edentulous (2). [GK –100; modified from BJS – 45] – unordered
101. **Posterior coronoid.** denticles only (0); teeth only (1); edentulous (2). [GK – 101; modified from BJS – 46] – unordered
102. **Prearticular (anterior extent).** midpoint of middle coronoid (0); not anterior to posterior coronoid (1). [BJS – 47; adapted from McHugh, 2012]
103. **Prearticular (hamate process).** absent (0); present and short (1); present and tall (2). [GK –103; modified from BJS – 48; adapted from McHugh, 2012, and Schoch, 2013]
104. **Preglenoid process.** labial side of surangular with straight dorsal margin anterior to glenoid (0); with dorsally convex margin (1). [adapted from Schoch, 2013 – 144]
105. **Meckelian window (length).** small round or oval opening (0); elongate window shorter than the adductor fossa (1); as long or longer than adductor fossa (2). [GPM – 65; Schoch, 2013] – ordered
106. **Postsplenial (contribution to Meckelian window).** present (0); absent (1). [modified from GK –106; adapted from Warren and Marsicano 2000; McHugh, 2012]
     a. **Original character: *Meckelian window (borders)****. (0) bounded by the prearticular, postsplenial, and angular; (1) bounded by the prearticular and postsplenial; (2) bounded by the prearticular and angular; (3) bounded by the prearticular, postsplenial, middle coronoid and angular; (4) entirely enclosed by the postsplenial. [Adapted from Warren and Marsicano 2000; McHugh, 2012]. – unordered*
        i. This character was split so that the contribution of each element is considered separately. This can allow for states not considered in the original conception of this character such as the contribution of the posterior coronoid to the Meckelian window commonly seen in metoposaurids. Further characters pertaining to each potential contributing element were appended to the end of this list and the matrix. In the current dataset, all taxa have a contribution of the prearticular to the Meckelian window and thus its inclusion would be parsimony non-informative, so this character was omitted. However, this may not be the case in a broader taxonomic sample.
107. **Splenial contribution to symphysis.** absent (0); present (1). [BJS – 49]
108. **Splenial-coronoids.** splenial contacts multiple coronoids dorsally (0); splenial contacts only first coronoid dorsally (1). [PHS – 310]
109. **Angular (extent in lateral view).** posterior tooth row (0); middle of tooth row (1). [PHS – 270]
110. **Mandibular anterior ventral margin.** straight (0); concave (1). [BJS – 51]
111. **Mandibular tapering in height anteriorly.** absent (0); present (1). [BJS – 52]
112. **Postglenoid area.** short boss (0); distinct process (1); or extended, longer than glenoid facet (2). [Damiani, 2001; Schoch, 2008a] – ordered
113. **Glenoid (position).** above level of dorsal surface of dentary (0); at same level (1); below (2). [GK – 113; modified from Damiani, 2001; Schoch, 2008a] – ordered
114. ****Chorda tympanic foramen.** located on the suture between the articular and the prearticular (0); located on the prearticular alone (1); foramen absent (2); located on the suture between the surangular and the prearticular (3). [Yates & Warren, 2000 – 100] – unordered
     a. See main text for reasoning behind the problematic nature of this character.
115. **Presacral count.** more than 28 vertebrae (0); 23-28 (1); < 23 (2). [GK – 115; modified from Schoch, 2013 – 158] – ordered
116. **Atlas**. occipital surfaces separated (0); occipital surfaces contacting (1). [GPM – 110]
117. **Axial neural arch**. absent (0); present (1). [BJS – 53; adapted from Hunt 1993]
118. **Intercentrum (shape).** presacral intercentra form simple wedges (0); incomplete cylinders in which presacral intercentra are open dorsally but do not taper in lateral view as in typical wedges [NEW] (1); dorsally closed discs (2). [modified from GK – 118; adapted from Schoch, 2013; Yates & Warren 2000] – ordered
     a. **Original character: *Intercentrum (shape)****. presacral intercentra form simple wedges (0); dorsally closed discs (1); dorsally closed and elongated cylinders (2). (Yates & Warren 2000). [GPM – 74; adapted from Schoch, 2013] – ordered*
     b. The states of this character were modified to capture nuance in intercentrum shape within metoposaurids and to remove the replication of intercentrum length already taken into account in ch. 120.
119. **Trunk intercentrum (notochordal canal).** throughgoing and wider than intercentrum is high (0); thoroughgoing and narrower than intercentrum is high (1); reduced to surficial perforation or entirely closed (2). [GK – 119; modified from Schoch, 2013 – 164] – ordered
     a. In this study, the minimum width of the dorsal groove on intercentra that lack complete closure was used to assess relative diameter compared to the centrum.
120. **Trunk intercentrum (ventral surface).** ventral surface shorter than wide in ventral view, giving transversely rectangular outline (0); as long as wide, quadrangular (1); longer than wide [NEW] (2). [modified from Schoch, 2013 – 165] – ordered
     a. Modified phrasing to specify that this should be applied to intercentra from the trunk (i.e., anterior dorsal, mid-dorsal, and presacral intercentra of Konietzko-Meier et al., 2012).
     b. Added state 2 to account for anteroposteriorly elongate intercentra such as those of some plagiosaurids.
121. **Intercentrum (anterior surface).** always concave (0); or convex at least in some presacral centra (1). [Schoch, 2013 – 166]
122. **Ossified pleurocentra in posterior trunk vertebrae.** present (0); absent (1). [BJS – 54; adapted from Hunt, 1993]
123. **Neural spine (height).** low throughout vertebral column (0); dorsally extended in posterior portion of trunk or tail (1). [BJS – 56; adapted from Schoch 2008a]
124. ****Procoelous third vertebra.** absent (0); present (1). *Dutuitosaurus ouazzoui* is the only metoposaurid with a procoelous third vertebra. [BJS – 58; adapted from McHugh, 2012]
     a. This character is currently parsimony uninformative, but there are differences in vertebral body types in temnospondyls that may be useful in future studies with further clarification.
125. **Parapophysis.** segmental (0); intersegmental (1). [GPM – 78; adapted from Warren & Snell, 1991; Schoch, 2013]
126. **Ribs (uncinate processes).** absent (0); uncinate plate (1); uncinate process (2). [GK – 126; modified from BJS – 60; adapted from McHugh, 2012]
127. ***Clavicle (contact).** absent (0); reduced to anterior part of pectoral girdle (1); prolonged contact (2). [BJS – 61; adapted from McHugh, 2012] – ordered
     a. Clavicular contact is often ambiguous if no articulated specimens are known. One proposed way to assess this condition is the anteromedial margin of the clavicle that can be either “straight” (prolonged contact) or “curved or…serrated” (no contact). States 1 and 2 lie along a continuum, but all scores were checked to remain internally consistent.
128. **Clavicle (ornamentation).** reticulate ornamentation confined to small region with broader arc of radiating grooves (0); reticulate ornamentation forms large portion of posterolateral surface with narrower arc of radiating grooves (1). [GPM: 106; adapted from Hunt, 1993]
129. **Clavicle (sensory groove).** absent (0); present along posterolateral margin of ventral surface (1). [GPM – 105]
130. **Interclavicle (proportions).** as long as it is wide (0); 1.3 times as long as wide (1); more than twice as long as wide (2). [Schoch, 2013 – 182] – ordered
131. **Interclavicle (relative size of reticulate ornamentation).** large, equal to or greater than half the total width of the ornamented surface (0); small, less than half the total width of the ornamented surface (1). [GPM – 107]
132. **Interclavicle (anterior termination of ornamented region).** rounded (0); tapering to a triangular point (1). [GPM – 108]
133. **Humerus (supinator).** discrete projection offset from ectepicondyle (0); process largely confluent with ectepicondyle (1); process absent (2). [GK – 133; modified from GPM – 88; Yates & Warren 2000]
134. **Humerus (torsion).** strong, 70-90° (0); weak, well below 60° (1). [GPM – 90; adapted from Witzmann & Schoch, 2006a; Schoch, 2013]
135. **Ilium (shaft, orientation).** posterodorsally inclined (0); vertically oriented (1). [Schoch, 2013 – 201]
136. **Ilium (shaft, length).** twice the length of the puboischial articulation of the base or greater (0); less than that (1). [modified from GPM – 92; adapted from Schoch, 2013 – 198]
     a. **Original character: *Ilium (shaft, length)****. shaft of variable length but laterally flattened (0); very long and slender (1). [GPM – 92; adapted from Schoch, 2013 – 198]*
     b. This character was split from GK (136 → 136, 164) to distinguish between the cross-section of the ilium shaft and the length of the shaft.
137. **Ilium (anterior margin of dorsal shaft).** straight (0); concave (1); convex with flexure at the mid-height (2). [modified from BJS – 63 and GPM – 109; adapted from Schoch 2008a]
138. **Pubis.** unossified (0); ossified (1). [Schoch, 2013 – 203]
139. **Femur (trochanter).** internal trochanter present as discrete process offset from shaft (0); continuously confluent with shaft (1). [GPM – 95; adapted from Schoch, 2013]

New Characters

140. **Orbit (orientation).** dorsally oriented (0); between dorsally and laterally oriented (1); laterally oriented (2). [NEW] – ordered
141. **Lacrimal (posterior terminus).** well anterior to the orbit (0); level or just anterior to the orbital margin (1); posterior to the orbital margin (2); well posterior, at about the mid length of the orbit (3). [NEW]
142. **Prefrontal (anterior end, angle).** <90 degrees (0); ≥90 degrees (1). [NEW]
     a. This character is meant to complement ch. 20 which describes the appearance of the anterior end of the prefrontal as pointed or blunt, but in some metoposaurids especially those with more anteriorly placed orbits, the medial and lateral sutures can express a wider angle independent of the pointed or blunt shape of the anterior process of the element.
143. **Prefrontal-nasal contact.** present (0); absent (1). [NEW]
     a. **Original character: *20.* *Prefrontal (anterior contacts)****. nasal and lacrimal (0); nasal and maxilla (1); lacrimal and maxilla (2). [GK – 20, BJS – 11; adapted from McHugh, 2012]*. This character is one of three replacing BJS – 11 (GK – 20) which was modified from McHugh (2012) – 23 and Laurin & Reisz (1997) – 26.
     b. See notes on ch. 20.
144. **Frontal (width).** tapering anteriorly (0); uniform width throughout length (1); tapering posteriorly (2). [NEW] – unordered
     a. Posterior and anterior tapering of the frontal are not mutually exclusive as some trematosaurs have frontals that are widest in the center and taper both anteriorly and posteriorly. Metoposaurids tend to have frontals that are the widest at the anterior end then taper posteriorly.
145. **Postfrontal-postorbital suture (orientation).** steeply inclined toward the sagittal plane, intersecting with sagittal plane on the skull roof (0); shallowly inclined toward the sagittal plane, intersecting with sagittal plane less than the skull length posterior to the skull roof (1); parasagittal, intersecting more than skull length posterior to skull roof (2); intersecting anteriorly to the orbits (3). [modified from GK – 27] – unordered
     a. This character was measured on specimens when possible. To determine this character, fit a straight line from the point at which the postorbital and postfrontal contact the orbital margin to their posterior-most contact.
146. **Postparietals (posterior bulge).** in dorsal view, postparietals have a smooth and concave posterior margin (0); postparietals form a posteriorly directed bulge (1). [NEW]
147. **Tabular horn (long axis orientation).** Posterior (0); posterolateral (1); lateral (2). – ordered
148. **Supratympanic flange.** simple rounded or sharp edged posterodorsal margin of the skull roof around the otic notch (0); smooth ‘gutter’ bordered above-and below by sharp ridges around the margin of the otic notch (1); smooth ‘gutter’ only visible in dorsal view on the squamosal (2). [modified from Yates and Warren, 2000 – 22; Berman et al., 1985 – 3; see also Schoch, 2013 – 55]
     a. State 1 of the original character was split to reflect variation in metoposaurids in which the smooth unornamented flange surrounding the otic notch is only present on the squamosal or if it extends onto the tabular.
149. **Accessory jugal sulcus**. absent (0); present (1). [NEW]
     a. A sulcus emerging from the jugal sulcus (here identified as an accessory jugal sulcus) is widely present among specimens of *Buettnererpeton bakeri* as well as two specimens of *Metoposaurus algarvensis* (FCT-UNL 600 and 601, figs. 2–3: Brusatte et al., 2015). During the study, several specimens of “*Buettneria*” *howardensis* and some previously referred to *Anaschisma browni* from Norian-aged strata were also found to have an accessory jugal sulcus (see main text).
150. **Accessory temporal sulcus.** absent (0); present (1). [NEW]
     a. Also referred to as the vertical portion of the suborbital part of the infraorbital sulcus of Säve-Söderbergh (1936). Note that this is not referring to the commissure between the temporal and supraorbital sulci but a sulcus that continues posteriorly alongside the temporal sulcus and arises from it.
151. **Transverse occipital lateral line sulcus**. present (0); present but discontinuous (1); absent (2). [modified from Schoch, 2019]
     a. The transverse occipital sulcus is sometimes considered to only be present in trematosauroids among stereospondyls (p. 49: Schoch, 2019). However, some capitosaurs and many metoposaurids have an incomplete occipital lateral line sulcus on the tabular (nearly complete transverse commissure in some metoposaurid specimens). Indeed, some plagiosaurids appear to have a transverse occipital sulcus as well (Hellrung, 2003).
152. **Exoccipital (lamellose process).** present, offset from occipital pillar, extending into foramen magnum (0); present, as a raised ridge confluent with the occipital pillar (1); absent (2). [NEW; adapted from Sulej, 2007] – ordered
153. **Exoccipital condyle:foramen magnum (maximum transverse width).** relatively narrow occipital condyle <0.95 (0); subequal 0.95–1.05 (1); relatively wide occipital condyle >1.05 (2). [NEW] – ordered
154. **Fodina vomeralis.** absent (0); present (1); present as fissure between the vomers (2). [NEW] – unordered
155. **Angular (contribution to Meckelian window).** present (0); absent (1). [Modified from GK –106; adapted from Warren and Marsicano 2000; McHugh, 2012]
     a. **Original character: *106. Meckelian window (borders)****. (0) bounded by the prearticular, postsplenial, and angular; (1) bounded by the prearticular and postsplenial; (2) bounded by the prearticular and angular; (3) bounded by the prearticular, postsplenial, middle coronoid and angular; (4) entirely enclosed by the postsplenial. [Adapted from Warren and Marsicano 2000; McHugh, 2012]. – unordered*
     b. See notes on ch. 106.
156. **Middle coronoid (contribution to Meckelian window).** absent (0); present (1). [Modified from GK –106; adapted from Warren and Marsicano 2000; McHugh, 2012]
     a. **Original character: *106. Meckelian window (borders)****. (0) bounded by the prearticular, postsplenial, and angular; (1) bounded by the prearticular and postsplenial; (2) bounded by the prearticular and angular; (3) bounded by the prearticular, postsplenial, middle coronoid and angular; (4) entirely enclosed by the postsplenial. [Adapted from Warren and Marsicano 2000; McHugh, 2012]. – unordered*
     b. See notes on ch. 106.
157. **Posterior coronoid (contribution to Meckelian window).** absent (0); present (1). [Modified from GK –106; adapted from Warren and Marsicano 2000; McHugh, 2012]
     a. **Original character: *106. Meckelian window (borders)****. (0) bounded by the prearticular, postsplenial, and angular; (1) bounded by the prearticular and postsplenial; (2) bounded by the prearticular and angular; (3) bounded by the prearticular, postsplenial, middle coronoid and angular; (4) entirely enclosed by the postsplenial. [Adapted from Warren and Marsicano 2000; McHugh, 2012]. – unordered*
     b. See notes on ch. 106.
158. **Stapes (quadrate process).** absent (0); present (1). [Schoch, 2013 – 150]
159. **Stapes (stapedial foramen).** present as an enclosed foramen between the proximal processes (0); present as an open notch between the proximal processes (1); absent, the space between the processes is imperforate and forms a continuous ridge (2). [modified from Yates and Warren, 2000 – 38]
     a. present as a large, throughgoing foramen proximal to the neck (0); present as a throughgoing foramen on the stylus (1); only a small, imperforate nutrient foramen (2); entirely absent (3).
        i. State 0 was modified to specify the position and size of the stapedial foramen. States 1 and 2 were added to distinguish variation found in metoposaurids and also across stereospondyls more generally.
160. **Stapes (proximal end).** distinct notch between the proximal heads (footplate and ventral process) (0); continuous sharp ridge between the proximal heads (1). [modified from Schoch, 2013 – 151]
161. **Notochord (position in trunk).** dorsally placed resulting in a dorsally open canal or a pit dorsal to the mid-height of the intercentrum (0); centrally placed (1). [NEW; adapted from Warren and Snell, 1991]
     a. In many large North American metoposaurid specimens, the notochordal pit is absent, but histological sectioning may reveal the position of the notochord. Notably, in large metoposaurid intercentra that overlap in stratigraphic range with *Apachesaurus*-type intercentra and that retain a notochordal pit and also all referred *Apachesaurus* intercentra, the notochord is centrally located.
     b. For intercentra in which the notochordal canal is exceptionally wide (e.g., *Sclerocephalus* or *Benthosuchus*), OTUs were scored as inapplicable (-).
162. **Dorsal notochordal incision on caudal intercentra.** open dorsally, forming wedge-or U-shaped anterior profile (0); closed, forming circular anterior profile (1). [GPM – 112]
     a. **Original character excluded from GK: *Caudal intercentra deeply notched.*** *present (0); absent (1). Dutuitosaurus ouazzoui is the only metoposaurid with deeply notched caudal intercentra. [BJS – 57; adapted from Hunt 1993]*
     b. GK considered this character unreliable given the paucity in well figured caudal intercentra from taxa for which complete caudal series are unknown. From my observations of hundreds of isolated intercentra of North American metoposaurids and comparisons with well defined criteria for determining axial position (Sulej, 2007; Konietzko-Meier et al., 2012), this character is included due to variability in North American metoposaurids.
163. **Clavicle (position).** posterior margin of clavicle located posterior to ossification center of interclavicle (center of region of reticulate ornamentation) (0); at the same level or anterior (1). [NEW; adapted from Schoch, 2008a]
     a. The ossification center of the interclavicle is almost always posterior to the posterior margin of the clavicle in metoposaurids. The shape and posterior extent of the clavicle was used by Schoch (2008a) to unite *Callistomordax* with metoposaurids, however the posterior extent of the clavicle in *Callistomordax* is much greater than in metoposaurids and more consistent with other stereospondyls.
164. **Clavicle (trough)**. absent (0); present (1). [NEW; adapted from Sulej, 2007]
     a. A trough along the lateral margin of the ascending plate of the clavicle is only known in metoposaurids (Sulej, 2007) and *Eocyclotosaurus appetolatus* (Rinehart & Lucas, 2016).
165. ***Clavicle (lateral margin).** straight (0); curved (1). [NEW; adapted from Spielmann and Lucas, 2012]
     a. Spielmann and Lucas (2012) suggested the curvature of the lateral margin of the clavicle may be diagnostic for *Apachesaurus gregorii*, however, small specimens referred to *Buettnererpeton bakeri* (Figs. S2G–H) also have this condition suggesting it may be ontogenetically variable.
166. **Ilium (shaft, cross-sectional profile).** laterally flattened/oval for the majority of its length (0); circular (1). [NEW]
     a. This character was split from GK (136) to distinguish between the cross-section of the ilium shaft and the length of the shaft.
     b. There is a small ilium from the Nobby Knob locality (*B. bakeri*) with a laterally flattened cross-section of the dorsal shaft, however, this specimen was not considered in the scoring of NK specimens due to significantly smaller size than any of the skulls used as OTUs. This likely indicates some ontogenetic variability in the ilium shaft of *B. bakeri*.
167. **Ilium (shaft flexure).** if flexure is present, it is pronounced (0); weak (1). [NEW]
     a. This character was added to reflect the known variation of metoposaurids: a specimen from the Popo Agie Fm and specimens from the Otis Chalk quarries have a weak flexure of the dorsal shaft. For any OTU that lacks a flexure of the ilium shaft, this character was scored as inapplicable (-).
168. **Ilium (shaft, mediolateral deflection).** shaft is essentially straight proximo-dorsally (0); shaft is deflected medially (1). [NEW]
     a. Ilia from the Rotten Hill bonebed have two distinct forms. In lateral view, the dorsal shaft of the ilium is straight, but in anterior view, the shaft of some ilia curves resulting in a base and acetabulum that are medially deflected. In others, there is no deflection. A brief survey of this unique feature in the Rotten Hill collection suggests that these morphotypes are represented approximately 1:1 and thus could be the result of sexual dimorphism, but a more rigorous survey of postcranial variability is needed.

**Included taxa.**

Supplemental Table 1. Sources used for scoring of character matrices.

| **Taxon** | **Reference(s)** |
| --- | --- |
| *Almasaurus habbazi* | Dutuit (1976a); pers. comm. C. So, 2025 |
| *Anaschisma browni* | Gee et al. (2020); Kufner & Gee (2021); this study |
| *Apachesaurus gregorii* | Spielmann & Lucas (2012); this study |
| *Archegosaurus decheni* | Witzmann (2005); Witzmann & Schoch (2006) |
| “*Arganasaurus*” *azerouali* | Buffa et al. (2019) |
| *Arganasaurus lyazidi* | Dutuit (1976); Hunt (1993); Buffa et al. (2019); pers. comm. D. Germain, 2025 |
| *Buettnererpeton bakeri* | Gee & Kufner (2022); this study |
| *Benthosuchus sushkini* | Bystrow and Efremov (1940) |
| *Bothriceps australis* | Warren et al. (2011) |
| *Callistomordax kugleri* | Schoch (2008a) |
| *Chinlestegophis jenkinsi* | Pardo et al. (2017) |
| *Compsocerops cosgriffi* | Sengupta (1995) |
| *Cyclotosaurus intermedius* | Sulej & Majer (2005) |
| *Dutuitosaurus ouazzoui* | Dutuit (1976b) |
| *Eocyclotosaurus appetolatus* | Rinehart & Lucas (2016) |
| *Gerrothorax pulcherrimus* | Hellrung (2003), Jenkins et al. (2008), Witzmann et al. (2012) |
| *Lydekkerina huxleyi* | Pawley & Warren (2005), Hewison (2007, 2008), Jeannot et al. (2006) |
| *Lyrocephaliscus euri* | Säve-Söderbergh (1936); Mazin & Janvier (1983) |
| *Mastodonsaurus cappelensis* | Schoch et al. (2023) |
| *Mastodonsaurus giganteus* | Schoch (1999) |
| *Metoposaurus algarvensis* | Brusatte et al. (2015) |
| *Metoposaurus diagnosticus* | Fraas (1889), Sulej (2002) |
| *Metoposaurus krasiejowensis* | Sulej (2002, 2007) |
| *Ninumbeehan dookoodukah* | So et al. (2024); pers. obsv. |
| *Panthasaurus maleriensis* | RoyChowhury (1965); Sengupta (2002); Chakravorti & Sengupta (2018) |
| *Quasicyclotosaurus campi* | Schoch (2000) |
| *Rhineceps karibaensis* | Steyer & Sidor (2025) |
| *Rhineceps nyasaensis* | Watson (1962); Marsicano et al. (2017) |
| *Rileymillerus cosgriffi* | Bolt & Chatterjee (2000); pers. obsv. |
| *Sclerocephalus haeuseri* | Schoch & Witzmann (2009) |
| *Thoosuchus yakovlevi* | Damiani & Yates (2003); Shishkin et al. (2023) |
| *Trematolestes hagdorni* | Schoch (2006); Schoch & Mujal (2022) |
| *Trematosaurus brauni* | Schoch (2019) |
| *Wetlugasaurus angustifrons* | Schoch & Milner (2000); Schoch (2008b) |

**Character state changes.**

11. - → 0, Several species were coded as “not applicable” by GK for character 11 (maxilla contribution to the orbital margin). These were adjusted to “no contact” or “unknown” where relevant since all sampled taxa have both a maxilla and an orbit. It should be noted that the orbit is separated from the maxilla by wide jugals in all of the taxa coded as inapplicable, and future work may need to reassess the wording of this character when looking at broader stereospondyl relationships.

*Almasaurus habazzi*

8. 0/1 → 1, There are just under 50 teeth in the maxilla of *Almasaurus* (C. So, pers. comm., 2025).

23. 0 → 2(new), The prefrontal and jugal meet at a weak point contact on the ventral margin of the orbit. This contact is displaced in the distorted skulls of *Almasaurus*, but compares favorably with the morphology found in *Ninumbeehan dookoodukah* (C. So, pers. comm., 2025).

99. 0,1 → 1, A row of teeth is clearly visible from the posterior to the middle coronoid on a referred mandible of *Almasaurus* (C. So, pers. comm., 2025).

100. 0,1 → ?, It is unclear whether the tooth-like structures illustrated by Dutuit (fig. 94: 1976a) are anchored to the mandible or not.

101. 0,1 → 1, A row of teeth is clearly visible from the posterior to the middle coronoid on a referred mandible of *Almasaurus* (C. So, pers. comm., 2025).

123. 0 → ?, Neural arches from the posterior trunk and tail of *Almasaurus* are unknown (Dutuit, 1976a).

Specimens of *Anaschisma browni*

FMNH UC 448

34. 0,1 → 0, Gee et al. (2020:fig. 3b) interpreted FMNH UC 448 as having a contact between the parietal and the postorbital. This is unusual but not unique among specimens previously referred to *Anaschisma browni* (e.g., Lucas et al., 2016:fig. 30a). The interpreted lateral extension of the parietal crosses a segment of reconstruction, and the purported suture is confluent with a crack that extends through the rest of the postorbital. Here, it is considered more likely that this suture was lost in whatever damaged fragment needed to be reconstructed and thus the other side of the specimen was used to score this character.

86. ? → 1, Faint ornamentation can be seen on the parasphenoid basal plate and cultriform process with raking light.

MU 537

48. 1 → ?, Most of the dorsal portion of the postparietals is missing.

*Buettnererpeton bakeri* (at the species-level)

19. 0 → 0,1, From personal observation of many specimens referable to *B. bakeri*, the morphology of the anterior end of the prefrontal is variable.

30. 1 → 0,1, The anterior margin of the jugal is most commonly level with the anterior orbital margin, however, in one skull from the type locality the jugal terminates level on the left side and anteriorly on the right side (fig. 9: Gee & Kufner, 2022). This character is also variable among additional referred specimens in the current study.

38. 0 → 1, All specimens of *B. bakeri* have a “subtriangular” squamosal in which the squamosal and the quadratojugal suture follows a continuous curve rather than being “kinked” at the squamosal-jugal-quadratojugal joint.

41. 1 → 0,1, In one specimen in which the maxilla is complete (UMMP 13823), the maxilla and quadratojugal meet at a point contact. However, in the largest known nearly complete skull of *B. bakeri* (TTU-P11047), the maxilla and quadratojugal are separated by the jugal despite the maxilla being complete.

58. 0 → 0,1, Several specimens in the current study referable to *B. bakeri* exhibit variability in this character.

76. 0 → 1, For all specimens in which the ectopterygoid-palatine suture is visible and complete, a kinked suture is apparent.

114. 1 → 2, A small foramen that may be the chorda tympani foramen (see discussion in main text) can be seen on the articular in one specimen from the Elkins Place bone bed (fig. 35: Gee & Kufner, 2022).

116. 0 → 1, The only atlas from the Elkins Place bone bed has occipital surfaces that are incompletely rimmed and appear more like atlas intercentra from referred specimens of *Anaschisma* which had been coded by GK as contacting. This is distinct from atlantes of *Metoposaurus krasiejowensis* that have fully rimmed occipital surfaces.

117. 1 → 0, The only putative axis from the type locality of *B. bakeri* lacks a fused neural arch.

124. 1 → 0, All putative cervical or postcervical intercentra from the type locality of *B. bakeri* are opisthocoelous.

126. 2 → 1, Most ribs lack an uncinate plate or process, but the few that do have this feature in the anterior trunk have a plate and not the distinct triangular process present in taxa such as *Eryops*.

131. 0 → 0,1, Some interclavicles from the Elkins Place bone bed have a small region of reticulate ornamentation on the ventral side of the interclavicle (figs. 55b, 56a, d: Gee & Kufner, 2022), but most have a relatively large region of reticulate ornament.

Specimens of *Buettnererpeton bakeri*

UMMP 13820

48. 1 → 0, In dorsal view, the postparietals form a bulge on their posterior margin.

*Apachesaurus gregorii*

19. 0 → 1, The morphology of the anterior end of the prefrontal could only be reevaluated in two specimens of *Ap. gregorii* (the holotype and a referred specimen), and it is blunt in both.

30. 1 → 0,1, The states of this character are variable within the holotype of *Ap. gregorii* (UCMP 63845) with the left side jugal being level with and the right side terminating well anterior to the orbital margin.

31. 0 → 1, The infraorbital sulcus of UCMP 63845 forms a stepped but approximately Z-shaped flexure (pers. obsv.).

49. 1 → 2, Previous work (e.g., fig. 11: Spielmann and Lucas, 2012) and the current study confirm the paraquadrate foramen is surrounded exclusively by the squamosal and quadratojugal in UCMP 63845. This cannot be assessed for other specimens.

52. 1 → 2, The holotype of *Apachesaurus gregorii* exhibits a large subtriangular fossa on either side of the occiput. On the left side, a sheet of bone with a small circular hole is present recessed within this fossa, and here, this hole is considered the posttemporal fenestra. On the right side, this sheet of bone is missing, and this is due to damage based on broken margins surrounding the opening. A posttemporal fenestra recessed within a subtriangular fossa is also present in *Dutuitosaurus* (fig. 7: Dutuit, 1976b), and it is not clear if there could be ontogenetic influence in the appearance of this character.

61. 0 → 1, In all specimens of *Apachesaurus* evaluated here for which this character could be scored, the infraorbital sulcus merges with the jugal sulcus.

92. 2 → 0, The width of the cultriform process changes only very gradually. This character is intended to differentiate specimens that have a clear constriction in the cultriform process.

94. 0 → 1, The quadrate of *Apachesaurus* does not differ appreciably from that of other metoposaurids.

121. 0 → 1, An anterior dorsal intercentrum from the type locality of *Apachesaurus* is opisthocoelous.

127. ? → 0, The only complete referred clavicle of *Apachesaurus* (fig. 7a: Rinehart and Lucas, 2018) has a flat medial edge indicative of a prolonged contact of the clavicles.

“*Arganasaurus*” *azerouali*

33. ? → 1, The left side of the holotype is relatively undistorted and has an angled parietal-supratemporal suture (fig. 1: Buffa et al., 2019).

42. 1 → ?, The figure of the holotype of *Ar. azerouali* (fig. 4: Buffa et al., 2019) shows an unusual arrangement of the quadrate where it is exceptionally large, uncharacteristic for most temnospondyls outside of some dissorophoids. Sutures of the quadrate, squamosal, and quadratojugal are difficult to discern in the accompanying photo, so whether the squamosal contacts the quadrate is currently considered unknown.

57. ? → 0, Buffa and colleagues (pp. 6–7: 2019) note that the infraorbital sulcus forms a gentle curve across the lacrimal of the referred specimen MNHN.F.TAL 17 (fig. 2c, therein).

*Arganasaurus lyazidi*

5. ? → 1, Elongate grooves are present in the prepineal region of a number of *Ar. lyazidi* specimens (pers. comm. D. Germain, 2025).

9. 0 → 0,1, While some specimens of *Ar. lyazidi* lack an alary process of the premaxilla, at least one specimen (MNHN-F-ALM216) has an alary process along the medial narial margin.

14. - → 0, In this new interpretation of a much shorter (but still elongate) lacrimal that does not reach the narial margin, the lacrimal is precluded from contact with the nasal by a prefrontal-maxilla contact.

17. 0 → 1, The extremely elongate lacrimal of previous interpretations appears to be due to a fracture in the maxilla that extends through the thinnest portion in the infraorbital sulcus.

20. 0 → 1, See specimen MNHN-F-ALM216.

58. ? → 1, See specimen MNHN-F-ALM216.

61. 0 → 0,1, Several examples of *Ar. lyazidi* have an infraorbital sulcus that terminates anterior to the postorbital sulcus, but these sulci join together in at least one specimen (Dutuit, 1976b).

64. ? → 1, See specimen MNHN-F-ALM216.

68. ? → 1, See specimen MNHN-F-ALM216.

71. ? → 0, See specimen MNHN-F-ALM216.

75. ? → 1, See specimen MNHN-F-ALM216.

77. ? → 1, The surface of the pterygoid of specimen MNHN-F-ALM216 is well-preserved and does not show evidence of the grinding present in the anterior part of the palate that precludes identification of much of the dentition. This is taken to mean that the lack of denticles suggests true absence which is the case in all other metoposaurids.

78. ? → 2, See specimen MNHN-F-ALM216.

80. ? → 0, See specimen MNHN-F-ALM216.

81. 0,1 → 1, Preparation removed much of the detail of the palate of most specimens of *Ar. lyazidi*, and the posterolateral flange of the pterygoid seems to have been damaged frequently during preparation. In those specimens with less damage, it is present.

86. ? → 1, See specimen MNHN-F-ALM216.

93. ? → 1, See specimen MNHN-F-ALM216.

*Benthosuchus suskini*

127. ? → 0, Bystrow and Efremov (fig. 48: 1940) reconstructed the clavicles with no contact, and the lack of a flat edge at the anterior end supports this interpretation.

137. 2 → 1,2, See fig. 53 of Bystrow and Efremov (1940).

*Bothriceps australis*

7. 1/2 → ?, None of the specimens of *Bothriceps* are sufficiently well preserved to accurately determine tooth counts (see Watson, 1956; Warren et al., 2011).

48. 1 → 0, See figs. 2c, 4c of Warren et al. (2011).

52. 0,1 → 1, The posttemporal fenestra is considered polygonal in shape here due to a distinct overhang formed by the postparietal and tabular that gives the dorsal margin a straighter or incised edge (fig. 2c: Warren et al., 2011).

118. 0,2 → 2, All intercentra of *Bothriceps* referred by Warren et al. (fig. 7: 2011) form discs.

125. ? → 0, The parapophysis is shared between the intercentrum and pleurocentrum and not by a pair of sequential intercentra, so this a segmental condition (Warren et al., 2011).

*Callistomordax kugleri*

118. 0,2 → 0, This character was originally scored as polymorphic due to the presence of a notochordal canal in anterior trunk centra, however, this canal is formed by the co-ossification of the paired pleurocentra and intercentrum (fig. 8: Schoch, 2008a). So rather than the entire element being a disc formed exclusively by the intercentrum, it is a compound element and all post-axial intercentra in *C. kugleri* form wedges in lateral view.

*Chinlestegophis jenkinsi*

4. 1/2 → ?, The anterior margin of the interpterygoid vacuity is not preserved (Pardo et al., 2017).

113. 1/2 → ?, The glenoid region of the mandible of *Chinlestegophis* is not preserved (Pardo et al., 2017).

*Cyclotosaurus intermedius*

52. 0,1 → 1, The posttemporal fenestra appears like an elongate triangle (fig. 4: Sulej & Majer, 2005).

127. 1 → ?, The anterior contact of the clavicle is not clear from the figures or the description of Sulej and Majer (2005). The only figured clavicle (fig. 6f–g: Sulej and Majer, 2005) is missing the anteromedial edge where the clavicles would contact. The pectoral girdle reconstruction (fig. 8e: Sulej and Majer, 2005) was based on an isolated interclavicle (ZPAL Ab III 887/1) which does not reveal the shape of the anteromedial contacts of the clavicles.

*Dutuitosaurus ouazzoui*

52. 0 → 2, *Dutuitosaurus* exhibits a condition also seen in *Apachesaurus* in which the posttemporal fenestra is small and subcircular but recessed within a subtriangular fossa (fig. 7: Dutuit, 1976b).

68. 0 → 1, The interpretation of the palate by Dutuit (1976b) shows a similar constriction of the maxilla by the vomer and palatine where it contributes to the choana.

118. 3 → 1, Dutuit (1976b) illustrated intercentra with a dorsal notch and interpreted this notch to be the notochordal canal. This is the generally accepted condition for most Triassic temnospondyls (Warren and Snell, 1991). Thus the trunk intercentra are not dorsally closed but have an open notochordal canal.

119. 2 → 1, With the reinterpreted notochordal canal located at the apex of trunk intercentra and no clear evidence of a notochordal pit, this shows that the notochordal canal passed through the intercentra. However, this may not be equivalent to the throughgoing notochordal canal of *Apachesaurus*.

122. ? → 0, Pleurocentra known from the posterior trunk of *Dutuitosaurus* have previously been interpreted as pathological (e.g., Sulej, 2007; Gee and Kufner, 2022). However with this new (revived: see Dutuit, 1976b) interpretation of the notochord passing through the dorsal apex of the intercentra, the presence of pleurocentra surrounding the dorsal side of the notochord seems to indicate ossifications consistent with earlier diverging stereospondyls.

*Eocyclotosaurus appetolatus*

96. ? → 1, Of the many skulls of *E. appetolatus*, none preserves an ossified sphenethmoid despite this being a common occurrence among other capitosaurs (Rinehart and Lucas, 2016). I consider it reasonable to infer that this element was unossified.

*Lydekkerina huxleyi*

100. 0/1 → ?, The anterior coronoid is not sufficiently well preserved in any of the references to determine the state of this character.

*Lyrocephaliscus euri*

8. 1/2 → 1, See Säve-Söderbergh (fig. 5: 1936).

*Mastodonsaurus giganteus*

119. 2 → 1,2, The anterior trunk intercentra of *Mastodonsaurus* have a notochordal pit in the dorsal half of the intercentrum, but posterior trunk intercentra have a dorsally open notochordal canal.

*Metoposaurus algarvensis*

48. 1 → 0, The only occipital region sufficiently figured exhibits very shallow postparietals with no apparent descending lamina (fig. 6a, b: Brusatte et al., 2015).

119. 2 → ?, The figures of *M. algarvensis* lack sufficient detail to address this character, and it is not addressed in the text (Brusatte et al., 2015).

*Metoposaurus krasiejowensis*

9. 0,1 → 0,1,2, One specimen(fig. 12: Sulej, 2007) has an alary process separated from the medial narial margin on the left side.

42. 1 → 0,1, Sulej (2007) noted the contact of the quadrate and squamosal in at least some specimens and reconstructed both states in his composite figure (fig. 1d, therein).

47. 0 → 1, In fig. 16a of Sulej (2007) this flange is present, but in the rest, it is either absent or uncertain, here considered uncertain.

57. 1 → 2, all specimens exhibit a Z-shaped flexure.

*Panthasaurus maleriensis*

116. 1 → 0,1, Two atlantes of *P. maleriensis* have been figured, one showing clear separation of the occipital surfaces (fig. 28: RoyChowdhury, 1965) and one showing the surfaces contacting (fig. 10f: Sengupta, 2002).

117. ? → 1, A partial neural arch is fused to the axis intercentrum (fig. 10a: Sengupta, 2002).

124. ? → 0, The third vertebra is preserved, and as in all metoposaurids except for *Apachesaurus*, the anterior trunk intercentra are opisthocoelous (fig. 10b: Sengupta, 2002).

Metoposauridae indet.

YPM VPPU 021742

9. ? → 1, This specimen was interpreted by GK as indeterminate for the alary process of the premaxilla, however, a line of exposed sediment extends from the medial margin of the naris to the supraorbital canal that elsewhere in this specimen is interpreted as evidence of sutures. Furthermore, this morphology of the premaxilla-nasal suture is consistent with other metoposaurids including a referred specimen of *B. bakeri* (UMMP 13820).

60. ? → 1, The posterior extent of the jugal sulcus (of the postorbital sulcus) can be seen in a cast of this specimen TMM 41742-1 in which it reaches the posterior skull roof margin.

*Quasicyclotosaurus campi*

85. 0/2 → ?, The basicranial suture is not sufficiently well preserved to determine its state (fig. 7: Schoch, 2000b).

*Rhineceps nyasaensis*

7. 1/2 → 2, Marsicano et al. (fig. 7: 2017) show the left premaxilla with nine tooth bases and the right with seven.

*Rileymillerus cosgriffi*

2. 0,1 → ?, The preorbital region is incomplete, so at best, the preorbital-postorbital length ratio is estimated at >0.9 but is currently unknown.

6. 1 → ?, *Rileymillerus* was initially described as lacking lateral line sulci. However, from personal examination of TTU-P9168, it appears that the surface is sufficiently damaged to make this assessment questionable. In particular, the circumorbital region is highly damaged, and a crack along the posterior margin of the orbit follows the course that may be expected of the postorbital/jugal sulcus of temnospondyls.

19. 1 → 0, The prefrontal of *Rileymillerus* was initially interpreted as forked with an indentation in the anterior end (fig. 2: Bolt and Chatterjee, 2000). From personal examination of the specimen, the prefrontal is probably better considered to be pointed with an angle between the margins of slightly less than 90°. There was putty infilling abundant cracks along the medial margin of the prefrontal.

20. - → 1, The initial interpretation of Bolt and Chatterjee (2000) of the preorbital element as a wide nasal is here upheld due to lack of evidence that this element is the lacrimal (as suggested by Kligman et al., 2023). As a result, the lacrimal is later scored as absent.

26. 0 → ?, The nasal is incomplete, so this character is scored as unknown. The initial score by GK is likely but cannot be verified.

31–33. ? → 1, 1, 0, The parietal margins can be reasonably inferred from personal observation of TTU-P9168 to the extent which they are necessary for these characters despite the reconstruction given by Bolt and Chatterjee (fig. 2-1: 2000).

39. 0 → 1, A convex projection that starts on the posterior margin of the squamosal and continues on the quadratojugal is present on TTU-P9168. This projection is directed slightly ventrally making it difficult to see in dorsal profile, and it is unclear if this deflection is due to true morphology or distortion.

41. 1 → ?, The presence of an *insula jugalis* is a poor predictor of a maxilla-quadratojugal contact because the maxilla is known to underlap the jugal and sometimes reach the quadratojugal (e.g., fig. 16: Gee and Kufner, 2022).

45. ? → 1, The anterior margins of the postparietals is clear, and laterally, any breakup of the bone that obscures the postparietal-tabular suture suggests relatively wide postparietals, at least wider than long.

48. ? → 0, TTU-P9168 has a simple bulge of the postparietal in occipital view and not the more elongated process (blade) like metoposaurids.

49. ? → 0, Bolt and Chatterjee (p. 675: 2000) note the presence of a small foramen that they interpreted as between the squamosal and quadratojugal. They also considered this foramen too small to be the paraquadrate foramen. I consider this foramen to be homologous to the paraquadrate foramen of other stereospondyls (e.g., *Almasaurus*: Dutuit, 1976a). The suture between the squamosal and quadratojugal also appears to be slightly different from that figured by Bolt and Chatterjee (fig. 3: 2000). A posterior hook-like process of the squamosal forms a much more narrow scarf joint between the squamosal and quadratojugal than initial interpretations suggest (figs. 2-5: Bolt and Chatterjee, 2000) resulting in a point contact of the quadrate and the squamosal. As a result, this foramen is completely enclosed in the quadratojugal, which is a fairly common morphology among stereospondyls (e.g., *Benthosuchus*: Bystrow & Efremov, 1940; *Mastodonsaurus*: Schoch, 1999; *Compsocerops*: Sengupta, 2002).

50. ? → 1, The reinterpreted paraquadrate foramen is much smaller than the other foramina of the occiput.

52. 1 → 0, Although the dorsal and ventrolateral margins are slightly flatter, the posttemporal fenestrae form elongate ovals unlike the more triangular fenestrae of many other stereospondyls (e.g., *Angusaurus*: Fernández-Coll et al., 2019).

56. 1→ 2, Bolt and Chatterjee (2000) noted that TTU-P9168 is distorted with the left side dorsally and medially displaced. When comparing only the right side elements of the suspensorium, the quadrate is clearly anterior to the occipital condyle.

57–61. - → ?, All characters of lateral-line sulcus morphology were coded as unknown rather than inapplicable to reflect that extensive damage to the surface of TTU-P9168 precludes identification of lateral line sulci. There is a groove that approximates the posterior margin of the orbit that may be a lateral line sulcus, but without an extensive revision of this taxon, it is better to consider this character dubious than to directly contradict the initial description.

70. 2 → ?, Bolt and Chatterjee (2000) described “an irregular row that begins medial to the anterior fang position and continues near the intervomerine suture”, however, on my inspection there is no clear evidence of this tooth row on the left vomer such as broken tooth bases. It may be that this tooth row was present and abraded through the difficult preparation process or from molding and casting, so I consider this character state unknown.

73–74. 1 → ?, The ectopterygoid of TTU-P9168 is not visible. Although it is likely that denticles were absent, it cannot be verified.

76. 0 → ?, The complete extent of the ectopterygoid-palatine suture is not visible in ventral view.

113. 0 → 1, Bolt and Chatterjee (2000) state that the glenoid facet is at the same level as the dentary. This was confirmed in my own study of TTU-P9168.

*Sclerocephalus haeuseri*

76. 0/1 → 0,1, At least one specimen of *S. haeuseri* has a kinked palatine-ectopterygoid suture (fig. 6a, e: Schoch & Witzmann, 2009). Others are illustrated with straight sutures (e.g., fig. 4: Boy, 1988).

*Trematolestes hagdorni*

8. 1/2 → 1,2, Modified from uncertain to polymorphic, but this polymorphism may be related to ontogeny, see Schoch and Mujal (fig. 7: 2022).

21. 0,2 → 0,1, All figured specimens have a prefrontal and frontal that terminate at nearly the same level, but in some specimens, the terminus of the prefrontal is located anterior to that of the frontal (Schoch and Mujal, 2022).

41. 0 → ?, The text of Schoch (2006) does not address the contact or lack thereof of the maxilla and quadratojugal. In fig. 4d of Schoch (2006) a point contact is reconstructed, but it is not clear that this is known in any of the specimens.

48. 0 → 0,1, See Schoch and Mujal (figs. 7d, e: 2022).

54. 1/2 → 1, See reconstructions of Schoch and Mujal (2022).

93. 0/1 → 0, The vomers close around the cultriform process at the level of the anterior margin of the interpterygoid vacuity, but there is an opening anterior to this for the fodina vomeralis (Schoch, 2006; Schoch & Mujal, 2022).

*Trematosaurus brauni*

41. 1 → 0, the only specimen in which a posterior suture of the maxilla has been identified in palatal view (GG 22.188; Schoch, 2019:fig. 7) has a point contact of the maxilla and quadratojugal.

54. 1,2 → 2, See Schoch (fig. 5a: 2019).

105. 1/2 → ?, Schoch (2019) noted that the lingual side of the mandible of *Trematosaurus* is very poorly known and did not figure it.

**Notes on non-metoposaurids.**

*Almasaurus habazzi*

151. Some specimens of *Almasaurus* have a series of joined pits along the posterior margin of the postparietals and tabulars here interpreted as a transverse occipital sulcus (pl. 60a: Dutuit, 1976a) but others do not (pl. 62b, 68d: Dutuit, 1976a). A transverse occipital sulcus is present in the putative sister taxon of *Almasaurus*, *Ninumbeehan dookoodukah* (So et al., 2024).

*Benthosuchus suskini*

151. Bystrow and Efremov (fig. 24: 1940) illustrated the transverse occipital sulcus, but it was not shown in any of the stippled drawings of the dermal skull roof. This may be an interpretation that this lateral line sulcus was present just posterior to the skull roof margin or that skulls that were not illustrated showed evidence of this sulcus. Here it is considered absent.

*Callistomordax kugleri*

41. The text and figure of this feature contradict one another (fig 1B, p. 88: Schoch, 2008a). The text description was used to score this character because it is definitively stated that the maxilla ends prior to the posterior termination of the ectopterygoid and cannot contact the quadratojugal.

*Cyclotosaurus intermedius*

151. The transverse occipital sulcus appears similar to the condition in *E. appetolatus* with a partial sulcus along the posterior margin of the otic notch (fig. 2: Sulej and Majer, 2005).

*Eocyclotosaurus appetolatus*

151. Some specimens of *E. appetolatus* appear to have partial transverse occipital sulci along the posterior margin of the otic notch (e.g., fig. 23: Rinehart and Lucas, 2016) while others do not (e.g., fig. 20: Rinehart and Lucas, 2016).

159. The notochordal canal of *E. appetolatus* ends about 5 mm above the center of the intercentrum.

*Lyrocephaliscus euri*

151. The transverse occipital sulcus is only expressed as a partial sulcus on the tabular (fig. 1, pl. 2.1: Sӓve-Soderbergh, 1936; fig. 2a: Mazin and Janvier, 1983).

*Trematolestes hagdorni*

161. Only the smallest specimen has an ossification center of the interclavicle posterior to the posterior margin of the clavicle.

*Wetlugasaurus angustifrons*

57. Schoch (2008b) considered the lacrimal flexure of the infraorbital sulcus to be Z-shaped, however, it does not have the same dramatic curvature seen in most metoposaurids or capitosaurs, so here is considered a simple curve.

**Instructions for analyzing the character matrix.**

Supplementary file S2 has all characters and OTUs used in this study in Nexus format. For each analysis, select characters or OTUs were removed from the matrix in a character matrix editor such as Mesquite (Maddison & Maddison, 2026) and corresponding changes in the numbering of ordered characters were made manually in the Bayes blocks which are provided in File S3. For the analyses incorporating all specimens, the species-level OTU of *Metoposaurus krasiejowensis* was removed, and for the analyses on only North American specimens (those observed firsthand), all of the specimens of *M. krasiejowensis* (beginning with ZPAL) were removed, and the species-level OTU was used instead. For the analyses removing only dubious characters (those marked with “**” above), only characters 95, 114, and 124 were removed, and for the analyses removing putative ontogenetically variable characters (those marked with “*” above), characters 46, 50, 86, 127, and 165 (of the original matrix numbering) were also removed. Each file was exported to the appropriate file format, and the Bayes blocks were appended to the Nexus files used in Bayesian inference.

**Histology.**

**Methods**

A preliminary investigation of the osteohistology of ribs referred to *Buettnererpeton bakeri* from Nobby Knob in Fremont Cty, WY allows assessment of relative age distribution at this site. Ribs were chosen due to their abundance and because ribs of at least some stereospondyls can act as a reasonably reliable record for growth comparable to the femur (McHugh, 2014). Each of the ribs can be assigned to type E/F based on the criteria of Sulej (2007). Each rib was photographed in standard anatomical orientations prior to measuring. Thin sections of the ribs were prepared using standard hard-tissue histological sampling techniques (Padian and Lamm, 2013). Photo micrographs of the slides were captured on a Nikon Eclipse E600 Pol confocal microscope in plane-polarized (PPL) and cross-polarized light (XPL) to differentiate calcite infill from bone mineral and bone mineral orientations. Each rib was cut near the mid-diaphysis.

**Results and interpretation**

In contrast with previously sampled metoposaurid femora (Steyer et al. 2004), the largest sampled ribs exhibit a large medullary cavity. The large medullary cavity of UWGM 7046 almost entirely displaced the cortical bone (Fig. S1C), probably due to a more distally positioned cut. The smallest rib (UWGM 7048, in part) exhibits the smallest medullary cavity that lacks indication of perimedullar erosion (Fig. S1A). Cortical bone is asymmetrically distributed, especially in the larger specimens (Figs. S1B, D–E). No lines of arrested growth (LAGs) could be identified in either of the UWGM 7048 sections (Fig. S1A), but they are present in all of the ribs except UWGM 7046 (likely owing to the position of the cut) (Figs. S1B, D–E). There are three LAGs and small erosional cavities within the cortex of UWGM 7051 (Fig. S1B). Structures interpreted as erosional cavities line the medullary cavity of UWGM 7046 (Fig. S1C). There are at least four LAGs in the cortex of UWGM 7049, and two LAGs can be identified internal to a muscular crest that follow the external morphology of this crest (Fig. S1D). Internal to the LAGs of the muscular crest of UWGM 7049, there are larger erosional cavities. The medullary cavity of UWGM 7055 is much smaller than the other large ribs, likely owing to the more proximal position of the cut. UWGM 7055 is incomplete and thus ensuring a consistent cut position was more difficult. There are at least five and maybe six LAGs on one side of UWGM 7055 and four on the other (Fig. S1E). UWGM 7055 also exhibits some secondary osteons adjacent to the medullary cavity and closely spaced LAGs in the external cortex that are interpreted as an external fundamental system (EFS). The EFS indicates that UWGM 7055 was approaching somatic maturity (Griffin et al., 2021).

The ribs sampled here were cut near mid-length, but these cuts exhibit significant variation in microanatomy suggesting a need to either serially section metoposaurid ribs to test for consistent histological features or section exclusively the proximal ribs following McHugh (2014). The largest ribs have more features associated with remodeling, including perimedullar erosional cavities, an expanded medullary cavity, and putative intracortical secondary osteons. With all of the described features taken together and as would be expected, the smallest rib is likely from a very early juvenile, and the remaining ribs exhibit more relatively advanced ontogenetic stages corresponding to increasing size.

**References.**

Anderson, J. S., Reisz, R. R., Scott, D., Fröbisch, N. B., & Sumida, S. S. (2008). A stem batrachian from the Early Permian of Texas and the origin of frogs and salamanders. *Nature*, *453*(7194), 515–518. <https://doi.org/10.1038/nature06865>

Berman, D. S., Reisz, R. R., & Eberth, D. A. (1985). *Ecolsonia cutlerensis*, an Early Permian dissorophid amphibian from the Cutler Formation of north-central New Mexico. *New Mexico Bureau of Mines & Mineral Resources Bulletin*, *191*.

Bolt, J. R., & Chatterjee, S. (2000). A new temnospondyl amphibian from the Late Triassic of Texas. *Journal of Paleontology*, *74*(4), 670–683.

Bolt, J. R., & Lombard, R. E. (2001). The mandible of the primitive tetrapod *Greererpeton*, and the early evolution of the tetrapod lower jaw. *Journal of Paleontology*, *75*(5), 1016–1042.

Bolt, J. R., & Lombard, R. E. (2010). *Deltaherpeton hiemstrae*, a new colosteid tetrapod from the Mississippian of Iowa. *Journal of Paleontology*, *84*(6), 1135–1151. <https://doi.org/10.1666/10-020.1>

Boy, J. A. (1988). Über einige Vertreter der Eryopoidea (Amphibia: Temnospondyli) aus dem europäischen Rotliegend (? Höchstes Karbon – Perm) 1. *Sclerocephalus*. *Paläontologische Zeitschrift*, *62*(1/2), 107–132.

Brusatte, S. L., Butler, R. J., Mateus, O., & Steyer, J. S. (2015). A new species of *Metoposaurus* from the Late Triassic of Portugal and comments on the systematics and biogeography of metoposaurid temnospondyls. *Journal of Vertebrate Paleontology*, *35*(3), 1–23. <https://doi.org/10.1080/02724634.2014.912988>

Buffa, V., Jalil, N. E., & Steyer, J. S. (2019). Redescription of *Arganasaurus* (*Metoposaurus*) *azerouali* (Dutuit) comb. nov. From the Upper Triassic of the Argana Basin (Morocco), and the first phylogenetic analysis of the Metoposauridae (Amphibia, Temnospondyli). *Papers in Palaeontology*, 1–19. <https://doi.org/10.1002/spp2.1259>

Bystrow, A. P., & Efremov, J. A. (1940). *Benthosuchus sushkini* Efremov – a labyrinthodont from the Eotriassic of the Sharzenga River. *Trudy Paleontologicheskogo Instituta*, *10*, 1–152.

Chakravorti, S., & Sengupta, D. P. (2018). Taxonomy, morphometry and morphospace of cranial bones of *Panthasaurus* gen. nov. *maleriensis* from the Late Triassic of India. *Journal of Iberian Geology*. <https://doi.org/10.1007/s41513-018-0083-1>

Damiani, R. J. (2001). A systematic revision and phylogenetic analysis of Triassic mastodonsaurids (Temnospondyli: Stereospondyli). *Zoological Journal of the Linnean Society*, *133*, 379–482.

Damiani, R. J., & Yates, A. M. (2003). The Triassic amphibian *Thoosuchus yakovlevi* and the relationships of the Trematosauroidea (Temnospondyli: Stereospondyli). *Records of the Australian Museum*, *55*(3), 331–342. <https://doi.org/10.3853/j.0067-1975.55.2003.1388>

Dutuit, J.-M. (1976a). Cinequiéme parite description d’*Almasaurus habbazi*. *Sciences de La Terre*, *36*(Sciences de la terre), 183–244.

Dutuit, J.-M. (1976b). Description des premiers stegocephales recueillis dans le couloir d’Argana (atlas occidental): Mémoires du Muséum National d’Histoire Naturelle. *Sciences de La Terre*, *36*(C), 1–253.

Englehorn, J., Small, B. J., & Huttenlocker, A. (2008). A redescription of *Acroplous vorax* (Temnospondyli: Dvinosauria) based on new specimens from the Early Permian of Nebraska and Kansas, U.S.A. *Journal of Vertebrate Paleontology*, *28*(2), 291–305. [https://doi.org/10.1671/0272-4634(2008)28[291:AROAVT]2.0.CO;2](https://doi.org/10.1671/0272-4634(2008)28%5B291:AROAVT%5D2.0.CO;2)

Fernández-Coll, M., Arbez, T., Bernardini, F., & Fortuny, J. (2019). Cranial anatomy of the Early Triassic trematosaurine *Angusaurus* (Temnospondyli: Stereospondyli): 3D endocranial insights and phylogenetic implications. Journal of Iberian Geology, 45(2), 269–286. <https://doi.org/10.1007/s41513-018-0064-4>

Gee, B. M., & Kufner, A. M. (2022). Revision of the Late Triassic metoposaurid “*Metoposaurus*” *bakeri* (Amphibia: Temnospondyli) from Texas, USA and a phylogenetic analysis of the Metoposauridae. *PeerJ*, *10*, e14065. <https://doi.org/10.7717/peerj.14065>

Gee, B. M., Parker, W. G., & Marsh, A. D. (2020). Redescription of *Anaschisma* (Temnospondyli: Metoposauridae) from the Late Triassic of Wyoming and the phylogeny of the Metoposauridae. *Journal of Systematic Palaeontology*, *18*(3), 233–258. <https://doi.org/10.1080/14772019.2019.1602855>

Griffin, C. T., Stocker, M. R., Colleary, C., Stefanic, C. M., Lessner, E. J., Riegler, M., Formoso, K., Koeller, K., & Nesbitt, S. J. (2021). Assessing ontogenetic maturity in extinct saurian reptiles. *Biological Reviews*, *96*(2), 470–525. <https://doi.org/10.1111/brv.12666>

Hellrung, V. H. (2003). *Gerrothorax pustuloglomeratus*, ein Temnospondyle (Amphibia) mit knöcherner Branchialkammer aus dem Unteren Keuper von Kupferzell (Süddeutschland). *Stuttgarter Beiträge zur Naturkunde Serie B (Geologie und Paläontologie)*, *330*.

Hewison, R. H. (2007). The skull and mandible of the stereospondyl *Lydekkerina huxleyi*, (Tetrapoda: Temnospondyli) from the Lower Triassic of South Africa, and a reappraisal of the family Lydekkerinidae, its origin, taxonomic relationships and phylogenetic importance. *Journal of Temnospondyl Palaeontology*, *1*(1), 1–80.

Hewison, R. H. (2008). The sacral region, pelvis and hind limb of the stereospondyl *Lydekkerina huxleyi* (Tetrapoda: Temnospondyli) from the Lower Triassic of South Africa. *Journal of Temnospondyl Palaeontology*, *2*(1), 1–26.

Jeannot, A. M., Damiani, R., & Rubidge, B. S. (2006). Cranial anatomy of the Early Triassic stereospondyl *Lydekkerina huxleyi* (Tetrapoda: Temnospondyli) and the taxonomy of South African lydekkerinids. *Journal of Vertebrate Paleontology*, *26*(4), 822–838. <https://doi.org/10.1671/0272-4634(2006)26%255B822:CAOTET%255D2.0.CO;2>

Jenkins, F. A., Shubin, N. H., Gatesy, S. M., & Warren, A. (2008). *Gerrothorax pulcherrimus* from the Upper Triassic Fleming Fjord Formation of East Greenland and a reassessment of head lifting in temnospondyl feeding. *Journal of Vertebrate Paleontology*, *28*(4), 935–950. <https://doi.org/10.1671/0272-4634-28.4.935>

Kligman, B. T., Gee, B. M., Marsh, A. D., Nesbitt, S. J., Smith, M. E., Parker, W. G., & Stocker, M. R. (2023). Triassic stem caecilian supports dissorophoid origin of living amphibians. *Nature*, *614*(7946), 102–107. <https://doi.org/10.1038/s41586-022-05646-5>

Konietzko-Meier, D., Bodzioch, A., & Sander, P. M. (2012). Histological characteristics of the vertebral intercentra of *Metoposaurus diagnosticus* (Temnospondyli) from the Upper Triassic of Krasiejów (Upper Silesia, Poland). *Earth and Environmental Science Transactions of the Royal Society of Edinburgh*, *103*(3–4), 237–250. <https://doi.org/10.1017/S1755691013000273>

Laurin, M., & Reisz, R. R. (1997). A new perspective on tetrapod phylogeny. In S. S. Sumida & K. L. M. Martin (Eds.), *Amniote origins—Completing the transition to land*. Academic Press.

Lucas, S. G., Rinehart, L. F., Heckert, A. B., Hunt, A. P., & Spielmann, J. A. (2016). Rotten Hill: A Late Triassic bonebed in the Texas Panhandle, USA. *New Mexico Museum of Natural History & Science*, *72*.

Maddison, W. P. and D.R. Maddison. (2026). Mesquite: a modular system for evolutionary analysis. Version 4.03. <https://www.mesquiteproject.org>

Marsicano, C. A., Latimer, E., Rubidge, B., & Smith, R. M. H. (2017). The Rhinesuchidae and early history of the Stereospondyli (Amphibia: Temnospondyli) at the end of the Palaeozoic. *Zoological Journal of the Linnean Society*. <https://doi.org/10.1093/zoolinnean/zlw032>

Mazin, J.-M., & Janvier, P. (1983). l’Anatomie de *Lyrocephaliscus euri* (Wiman), trématosaure du Trias Inférieur du Spitsberg: Arriére-crâne, squelette axial et ceinture scapulaire. *Palaeovertebrata*, *13*(1–2), 13–31.

McHugh, J. B. (2012). Temnospondyl ontogeny and phylogeny, a window into terrestrial ecosystems during the Permian-Triassic mass extinction. In *Iowa Research Online*. <http://ir.uiowa.edu/etd/2942>

McHugh, J. B. (2014). Paleohistology and histovariability of the Permian stereospondyl *Rhinesuchus*. *Journal of Vertebrate Paleontology*, *34*(1), 59–68. <https://doi.org/10.1080/02724634.2013.787429>

Padian, K., & Lamm, E.-T. (Eds.). (2013). *Bone Histology of Fossil Tetrapods: Advancing Methods, Analysis, and Interpretation*. University of California Press.

Pardo, J. D., Small, B. J., & Huttenlocker, A. K. (2017). Stem caecilian from the Triassic of Colorado sheds light on the origins of Lissamphibia. *Proceedings of the National Academy of Sciences*, *114*(27), E5389–E5395. <https://doi.org/10.1073/pnas.1706752114>

Pawley, K., & Warren, A. (2005). A terrestrial stereospondyl from the Lower Triassic of South Africa: The postcranial skeleton of *Lydekkerina huxleyi* (Amphibia: Temnospondyli). *Palaeontology*, *48*(2), 281–298. <https://doi.org/10.1111/j.1475-4983.2005.00446.x>

Rinehart, L. F., & Lucas, S. G. (2016). *Eocyclotosaurus appetolatus*, a Middle Triassic amphibian: Osteology, life history, and paleobiology. *New Mexico Museum of Natural History & Science Bulletin*, *70*, 1–117.

RoyChowdhury, T. K. (1965). A new metoposaurid amphibian from the Upper Triassic Maleri Formation of Central India. *Philosophical Transactions of the Royal Society B Biological Sciences*, *250*(761), 1–52. <https://doi.org/10.1098/rstb.1965.0019>

Säve-Söderbergh, G. (1936). On the morphology of Triassic stegocephalians from Spitsbergen, and the interpretation of the endocranium in the Labyrinthodontia. *Kunglik Svenska Vetenskapsakademiens Handlingar*, *16*(1), 1–181.

Schoch, R. R. (1999). Comparative osteology of *Mastodonsaurus giganteus* (Jaeger, 1828) from the Middle Triassic (Lettenkeuper: Longobardian) of Germany (Baden-Württemberg, Bayern, Thüringen). *Stuttgarter Beiträge Zur Naturkunde, Serie B*, *278*(September 2017), 1–175.

Schoch, R. R. (2000). The status and osteology of two new cyclotosaurid amphibians from the Upper Moenkopi Formation of Arizona (Amphibia: Temnospondyli; Middle Triassic). *Neues Jahrbuch Für Geologie Und Paläontologie - Abhandlungen*, *216*(3), 387–411. <https://doi.org/10.1127/njgpa/216/2000/387>

Schoch, R. R. (2006). A complete trematosaurid amphibian from the Middle Triassic of Germany. *Journal of Vertebrate Paleontology*, *26*(1), 29–43. [https://doi.org/10.1671/0272-4634(2006)26[29:actaft]2.0.co;2](https://doi.org/10.1671/0272-4634(2006)26%5B29:actaft%5D2.0.co;2)

Schoch, R. R. (2008a). A new stereospondyl from the German Middle Triassic, and the origin of the Metoposauridae. *Zoological Journal of the Linnean Society*, *152*(1), 79–113. <https://doi.org/10.1111/j.1096-3642.2007.00363.x>

Schoch, R. R. (2008b). The Capitosauria (Amphibia): Characters, phylogeny, and stratigraphy. *Palaeodiversity*, *1*, 189–226.

Schoch, R. R. (2013). The evolution of major temnospondyl clades: An inclusive phylogenetic analysis. *Journal of Systematic Palaeontology*, *11*(6), 673–705. <https://doi.org/10.1080/14772019.2012.699006>

Schoch, R. R. (2019). Osteology of the temnospondyl *Trematosaurus brauni* Burmeister, 1849 from the Middle Buntsandstein of Bernburg, Germany. *Palaeodiversity*, *12*(1), 41. <https://doi.org/10.18476/pale.v12.a4>

Schoch, R. R., & Milner, A. R. (2000). *Stereospondyli* (P. Wellnhofer, Ed.; Vol. 3B). Pfeil.

Schoch, R. R., & Mujal, E. (2022). Ontogeny and adult osteology of the Middle Triassic temnospondyl *Trematolestes hagdorni*. *Neues Jahrbuch Für Geologie Und Paläontologie - Abhandlungen*, *306*(3), 265–286. <https://doi.org/10.1127/njgpa/2022/1106>

Schoch, R. R., & Witzmann, F. (2009). Osteology and relationships of the temnospondyl genus *Sclerocephalus*. *Zoological Journal of the Linnean Society*, *157*(1), 135–168. <https://doi.org/10.1111/j.1096-3642.2009.00535.x>

Schoch, R. R., Milner, A., Witzmann, F., & Mujal, E. (2023). A revision of *Mastodonsaurus* from the Anisian of Germany, and the evolutionary history of mastodonsaurid temnospondyls. *Neues Jahrbuch Für Geologie Und Paläontologie - Abhandlungen*, *309*(2), 123–140. <https://doi.org/10.1127/njgpa/2023/1154>

Sengupta, D. P. (1995). Chigutisaurid temnospondyls from the Late Triassic of India and a review of the family Chigutisauridae. *Palaeontology*, *38*(2), 331–339.

Sengupta, D. P. (2002). Indian metoposaurid amphibians revised. *Paleontological Research*, *6*(1), 41–65.

Shishkin, M. A., Novikov, I. V., Sennikov, A. G., Golubev, V. K., & Morkovin, B. I. (2023). CHAPTER I. ANNOTATED SYSTEMATIC CATALOG OF TRIASSIC TETRAPODS OF RUSSIA. *Paleontological Journal*, *57*(12), 1363–1468. <https://doi.org/10.1134/S003103012312002X>

Spielmann, J. A., & Lucas, S. G. (2012). Tetrapod fauna of the Upper Triassic Redonda Formation, east-central New Mexico: The characteristic assemblage of the Apachean land-vertebrate faunachron. *New Mexico Museum of Natural History and Science Bulletin*, *55*, 1–119.

So, C., Kufner, A. M., Pardo, J. D., Edwards, C. L., Price, B. R., Bevitt, J. J., LeClair-Diaz, A., St. Clair, L., Mann, J., Teran, R., & Lovelace, D. M. (2024). Fossil amphibian offers insights into the interplay between monsoons and amphibian evolution in palaeoequatorial Late Triassic systems. *Proceedings of the Royal Society B: Biological Sciences*, *291*(2033), 20241041. <https://doi.org/10.1098/rspb.2024.1041>

Steyer, J.-S., Laurin, M., Castanet, J., & De Ricqlès, A. (2004). First histological and skeletochronological data on temnospondyl growth: Palaeoecological and palaeoclimatological implications. *Palaeogeography, Palaeoclimatology, Palaeoecology*, *206*(3–4), 193–201. <https://doi.org/10.1016/j.palaeo.2004.01.003>

Steyer, J.-S., & Sidor, C. A. (2025). The first Paleozoic temnospondyl from Zambia: A new species of *Rhineceps* from the Permian Madumabisa Mudstone Formation, Mid-Zambezi Basin. *Journal of Vertebrate Paleontology*, *45*(sup1), 2451312. <https://doi.org/10.1080/02724634.2025.2451312>

Sulej, T. (2007). Osteology, variability, and evolution of *Metoposaurus*, a temnospondyl from the Late Triassic of Poland. *Palaeontologia Polonica*, *64*, 29–139.

Sulej, T., & Majer, D. (2005). The temnospondyl amphibian *Cyclotosaurus* from the Upper Triassic of Poland. *Palaeontology*, *48*(1), 157–170. <https://doi.org/10.1111/j.1475-4983.2004.00430.x>

Warren, A., & Snell, N. (1991). The postcranial skeleton of mesozoic temnospondyl amphibians: A review. *Alcheringa*, *15*(1), 43–64. <https://doi.org/10.1080/03115519108619009>

Warren, A., Rozefelds, A. C., & Bull, S. (2011). Tupilakosaur-like vertebrae in *Bothriceps australis*, an Australian brachyopid stereospondyl. *Journal of Vertebrate Paleontology*, *31*(4), 738–753. <https://doi.org/10.1080/02724634.2011.590563>

Watson, D. M. S. (1956). The brachyopid labyrinthodonts. *Bulletin of the British Museum (Natural History) Geology*, *2*(8), 315–392.

Witzmann, F. (2005). Cranial morphology and ontogeny of the Permo-Carboniferous temnospondyl *Archegosaurus decheni* Goldfuss, 1847 from the Saar–Nahe Basin, Germany. *Transactions of the Royal Society of Edinburgh: Earth Sciences*, *96*(2), 131–162. <https://doi.org/10.1017/S0263593300001279>

Witzmann, F., & Schoch, R. R. (2006). The postcranium of *Archegosaurus decheni*, and a phylogenetic analysis of temnospondyl postcrania. *Palaeontology*, *49*(6), 1211–1235. <https://doi.org/10.1111/j.1475-4983.2006.00593.x>

Witzmann, F., Schoch, R. R., Hilger, A., & Kardjilov, N. (2012). Braincase, palatoquadrate and ear region of the plagiosaurid *Gerrothorax pulcherrimus* from the Middle Triassic of Germany. *Palaeontology*, *55*(1), 31–50. <https://doi.org/10.1111/j.1475-4983.2011.01116.x>

Yates, A. M., & Warren, A. A. (2000). The phylogeny of the ‘higher’ temnospondyls (Vertebrata: Choanata) and its implications for the monophyly and origins of the Stereospondyli. *Zoological Journal of the Linnean Society*, *128*(1), 77–121. <https://doi.org/10.1111/j.1096-3642.2000.tb00650.x>

**Supplemental Figures.**

**Figure S1. Thin sections (A–E, left XPL, right PPL) and photographs in dorsal view (A’–E’) of representative ribs referred to *Buettnererpeton bakeri* from Nobby Knob in Fremont County, WY.** (A) UWGM 7048 (in part), 1 and 2 correspond to separate cuts indicated on A’; (B) UWGM 7051; (C) UWGM 7046; (D) UWGM 7049; (E) UWGM 7055 (in part), including a closeup of the putative EFS (inset). White arrows indicate lines of arrested growth (LAGs). Abbreviations: EC, erosional cavity; MC, medullary cavity; SO secondary osteons. Left scale bars for A–E equal 500 µm; right scale bars for A’–E’ equal 1 cm; scale bar for closeup of E equals 125 µm.

**Figure S2. Photographs of select ilia of *Buettnererpeton bakeri* from Nobby Knob in Fremont County, WY.** (A) UWGM 7047, left ilium in left lateral view; (B) UWGM 8276, left ilium in left lateral view; (C) UWGM 7375, right ilium in right lateral view (reflected for comparison). Anterior is to the left. Scale bar equals 1 cm.

**Figure S3. Closeup photograph of the left antorbital region of WT 3011 (*Tecovaserpeton perfectum*) from Rotten Hill in Potter County, TX.** White arrows indicate the positions where the sutures of the lacrimal intersect with the orbital margin. Note the difference between the pen marking and the lacrimal-prefrontal suture (upper arrow). Scale not provided.

**Figure S4. Cranium of cf. *Tecovaserpeton* *perfectum*, UCMP 113554, in occipital view from UCMP V6333 (Davidson Creek) in Crosby Cty, TX.** Note that this specimen was lit from below. Abbreviations: fm, foramen magnum; oc, oblique crest of the pterygoid; pqf, paraquadrate foramen; ptd, pterygoid depression. Scale bar is approximately equal to 5 cm.

**Figure S5. Phylogenetic tree output removing only dubious characters and all specimens included.** (A) strict (Nelson) consensus tree of 1440 trees (895 steps; CI=0.195; RI=0.514); (B) Bayesian summary tree with all nodes fully bifurcated (sumt=allcompat). All branches set to equal length. Shapes indicating nodes: square, Neosterospondyli; triangle, Trematosauria; pentagon, Brachyopoidea; circle, Metoposauridae.

**Figure S6. Phylogenetic tree output removing only dubious characters and only North American specimens included.** (A) strict (Nelson) consensus tree of 1281 trees (872 steps; CI=0.246; RI=0.624 1019 trees); (B) Bayesian summary tree with all nodes fully bifurcated (sumt=allcompat). All branches set to equal length. Shapes indicating nodes: square, Neosterospondyli; triangle, Trematosauria; pentagon, Brachyopoidea; circle, Metoposauridae.

**Figure S7. Phylogenetic tree output removing both dubious and putative ontogenetically variable characters and all specimens included.** (A) strict (Nelson) consensus tree of 831 trees (849 steps; CI=0.203; RI=0.533); (B) Bayesian summary tree with all nodes fully bifurcated (sumt=allcompat). All branches set to equal length. Shapes indicating nodes: square, Neosterospondyli; triangle, Trematosauria; pentagon, Brachyopoidea; circle, Metoposauridae.

**Figure S8. Phylogenetic tree output removing dubious and putative ontogenetically variable characters and only North American specimens included.** (A) strict (Nelson) consensus tree of 784 trees (829 steps; CI=0.23; RI=0.585); (B) Bayesian summary tree with all nodes fully bifurcated (sumt=allcompat). All branches set to equal length. Shapes indicating nodes: square, Neosterospondyli; triangle, Trematosauria; pentagon, Brachyopoidea; circle, Metoposauridae.
